# Mapping the Landscape of Magnetic Field Effects on Neural Regeneration and Repair: A Combined Systematic Review, Mathematical Model, and Meta-Analysis

**DOI:** 10.1155/2023/5038317

**Published:** 2023-09-21

**Authors:** Meghan McGraw, Gabrielle Gilmer, Juliana Bergmann, Vishnu Seshan, Kai Wang, David Pekker, Michel Modo, Fabrisia Ambrosio

**Affiliations:** ^1^Discovery Center for Musculoskeletal Recovery, Schoen Adams Research Institute at Spaulding Rehabilitation Hospital, Boston, MA, USA; ^2^Department of Physical Medicine & Rehabilitation, Spaulding Rehabilitation Hospital, Boston, MA, USA; ^3^Medical Scientist Training Program, School of Medicine, University of Pittsburgh, Pittsburgh, PA, USA; ^4^Cellular and Molecular Pathology Graduate Program, University of Pittsburgh, Pittsburgh, PA, USA; ^5^Department of Biological Sciences in the Dietrich School of Arts & Sciences, University of Pittsburgh, Pittsburgh, PA, USA; ^6^Institute of Quantum Science and Technology, Department of Physics and Astronomy, University of Calgary, Calgary, AB, Canada; ^7^Hotchkiss Brain Institute, Cumming School of Medicine, University of Calgary, Calgary, AB, Canada; ^8^Department of Physical Medicine & Rehabilitation, Harvard Medical School, Boston, MA, USA; ^9^Department of Physics & Astronomy, University of Pittsburgh, Pittsburgh, PA, USA; ^10^McGowan Institute for Regenerative Medicine, University of Pittsburgh, Pittsburgh, PA, USA; ^11^Department of Bioengineering, University of Pittsburgh, Pittsburgh, PA, USA; ^12^Department of Radiology, University of Pittsburgh, Pittsburgh, PA, USA

## Abstract

Magnetic field exposure is a well-established diagnostic tool. However, its use as a therapeutic in regenerative medicine is relatively new. To better understand how magnetic fields affect neural repair *in vitro*, we started by performing a systematic review of publications that studied neural repair responses to magnetic fields. The 38 included articles were highly heterogeneous, representing 13 cell types, magnetic field magnitudes of 0.0002–10,000 mT with frequencies of 0–150 Hz, and exposure times ranging from one hour to several weeks. Mathematical modeling based on data from the included manuscripts revealed higher magnetic field magnitudes enhance neural progenitor cell (NPC) viability. Finally, for those regenerative processes not influenced by magnitude, frequency, or time, we integrated the data by meta-analyses. Results revealed that magnetic field exposure increases NPC proliferation while decreasing astrocytic differentiation. Collectively, our approach identified neural repair processes that may be most responsive to magnetic field exposure.

## 1. Introduction

Magnetic fields are a ubiquitous part of life, exerting their effects at all scales, from subatomic particles to the universe itself [1, 2]. In recent years, considerable interest has turned towards the therapeutic application of exogenous magnetic fields for the treatment of a multitude of diseases affecting neural tissues. Magnetic field stimulation is appealing since it is noninvasive and does not require anesthesia. Clinically, transcranial magnetic stimulation (TMS) is applied for the treatment of a variety of psychiatric disorders and applied magnetic fields have also been explored for cerebral ischemic stroke [3, 4]. The varying magnetic fields are thought to provide neuroprotective (i.e., rescue of apoptotic cells) effects, through putative mechanisms such as modulation of calcium and nitric oxide concentrations, free radical production, inhibition of apoptosis, induced angiogenesis, manipulation of cellular electrical activity, and reduced edema/inflammation [4–11]. However, in most cases, dosing schedules of magnetic field intensity and exposure lack standardization. Similarly, the impacts of magnetic field exposure on specific pathologies such as Alzheimer's disease (AD) are poorly understood. Some case-controlled and cohort studies report an increased risk of AD with static (no oscillations) magnetic field exposure (∼10 uT), while dynamic (oscillations) magnetic fields (10 mT, 100 Hz) have been suggested to increase brain activity in AD animal models [12–14]. These latter effects have been hypothesized to result from the breakdown of *β*-amyloid plaques [15].

Several factors contribute to both the uncertain benefit-risk ratio of magnetic field exposure and questions regarding the optimal dosing schedule. Magnetic field properties (magnitude, frequency, and pulsing), timing schedules (constant or fixed interval), species, system level (cell, tissue, organism), outcome of interest (proliferation, reproduction, cell health, and organism behavior), sensitivity of neuronal subpopulations to magnetic fields (e.g., neurons that make up the cortex may respond differently than neurons that make up the hippocampus), and many other characteristics are highly variable across the literature. Such study heterogeneity precludes definition of targeted and specific protocols. Through a combined approach, including a systematic review of the literature, mathematical modeling, and application of meta-analytical tools, here, we summarize the literature aimed to study how magnetic field stimulation affects neural repair. We focused on *in vitro* studies with the goal of synthesizing our mechanistic understanding of magnetic field effects on neural repair, as it is challenging to evaluate mechanistic cell-based effects *in vivo* due to complex nervous system interactions. In addition, we compiled quantifiable repair characteristics (differentiation, proliferation, viability, and maturation) as compared to controls, as well as magnetic field parameters (magnitude, frequency, and timing of exposure). With this data, we performed mathematical modeling and meta-analyses to characterize magnetic field effects on neural repair. Collectively, our work identifies magnetic field parameters that may be most effective in stimulating magneto-sensitive cellular repair processes and eliciting regeneration.

## 2. Materials and Methods

### 2.1. Systematic Review

We conducted a systematic literature search using the Preferred Reporting Items for Systematic Reviews and Meta-Analyses (PRISMA) statement and protocol (Supplementary Material S1), the Meta-Analysis of Observational Studies in Epidemiology (MOOSE) checklist (Supplementary Material S2), the Cochrane Handbook for Systematic Reviews of Interventions, and the practical guide for meta-analyses in animal studies [16–20]. We set our PECO (*P*opulation, *E*xposure, *C*omparison, and *O*utcome) inclusion criteria to identify publications that studied mammalian neural cells *in vitro* (population) exposed to magnetic fields (exposure). Control conditions consisted of an unexposed (besides the geomagnetic field) matched neural cell (comparison), while the outcome variable of interest was a metric of cellular repair or regenerative processes (outcome). Details regarding publication search, full-text screening, and information extraction are provided in the Supplementary Methods (available here).

Throughout this manuscript we use the term “neural” to refer to nervous system cells generally (e.g., astrocytes) and “neuronal” to refer to neurons specifically. Neural progenitor cells (NPCs) refer collectively to neural stem cells, neural progenitor cells, and induced pluripotent stem cells if they were maintained as stem cells throughout the experiment. Specific outcome variables were categorized into the following regenerative processes: viability, proliferation, differentiation, and maturation. “Viability” was defined as a metric that measured cell survival. “Proliferation” was defined as a metric that measured increases in cell numbers or signs of an active cell cycle (i.e., mitosis). “Differentiation” was defined as a metric that tracks the transformation from stem/progenitor cell to a specific mature cell type. “Maturation” was defined as a metric that quantified cellular size. Some dependent variables were included under multiple categories if that metric was used to categorize different repair processes. For example, Glial Cell Line-Derived Neurotrophic Factor (GDNF) is known to be associated with increased viability and differentiation [21, 22], and it was therefore included in both sets of mathematical models. Graphical data were converted to numerical data via a digital ruler. Intrarater reliability for graphical to numerical data conversion, calculated as the reviewer measuring the same data in sessions ten months apart (*n* = 5), was determined to be “excellent” (intraclass correlation coefficient [1, 2]: 0.9999 95% CI: [0.9846, 1.000]).

### 2.2. Rigor and Reproducibility Assessment

We scored rigor and reproducibility using the ARRIVE guidelines 2.0 [23]. Detailed information regarding the scoring strategy, interpretation, and statistics are described in the Supplementary Methods (available here).

### 2.3. Mathematical Models

We tested a variety of mathematical models to evaluate how different magnitudes, frequencies, percent exposure times, and total exposure times (magnetic field parameters and independent variables) affected viability, proliferation, differentiation, and maturation of neural cell populations (repair and regenerative processes, dependent variables). Magnitude was defined as the strength or amplitude of the magnetic field (mT), and frequency was defined as the number of cycles of magnetic field per unit time (Hz). The percentage of time exposed represented the proportion of experiment time that cells were exposed to a magnetic field relative to the total time lapsed until assessments were made. For example, if cells were exposed to a magnetic field for 1 hour, but a total of 10 hours passed from the start of the experiment to data collection, the percent of time exposed would be 0.1.

The mathematical model described below was run if there were at least 6 different values of the independent variable (e.g., magnitudes of 0.1, 1, 2, 5, 10, and 100 mT versus all datapoints investigating 100 mT). All simulations were completed in Python Jupyter Notebook (version 6.3.0) and followed recommendations for best practices in mathematical modeling [24, 25]. Data were first visualized for an understanding of what type of mathematical model (e.g., linear and logarithmic) would best represent the relationship. For all investigated relationships included in this review, linear or polynomial were determined to be most representative. As such, for each independent-dependent variable combination, initial settings for the mathematical model (linear and polynomial) and initial coefficient estimates were generated via the *numpy.polyfit* function (Python 1.23.0).

Simulations were set up as follows:

(1) $$\begin{array}{c} SMD\left(x\right)=ax^{2}+bx+c\text{,} \end{array}$$

where *x* is one of the independent variables (e.g., magnetic field magnitude); SMD(*x*) is the standardized mean difference between the magnetic field exposure group and the control group from an individual study for a repair/regenerative process dependent variable category; *a* and *b* are the coefficients predicted by the simulation; and *c* was set to 0. Therefore, an SMD of zero would indicate there is no difference between the magnetic field exposure and control group, a positive SMD would indicate the dependent variable is greater in the magnetic field exposure group compared to the control group, and a negative SMD would indicate that a dependent variable is lower in the magnetic field exposure group compared to the control group.

In our analysis, we fitted the coefficients of equation (1) by minimizing the root mean squared error (RMSE). To accommodate the fact that some exposure settings appeared in multiple different studies, we ran 20,000 different fits. In each fit, if inputs were the same across different studies, one was randomly selected. For instance, if study A used a magnitude of 10 mT and study B also used a magnitude of 10 mT, whether the SMD for study A or study B was input into the model was randomly selected. To ensure that we were adequately sampling identical inputs, we tracked how the conclusions drawn from the analysis varied with the number of separate fits. We observed that 200 separate fits were sufficient to fix the values of *a* and *b*. Finally, as stated above, we assumed that an external magnetic field exposure of zero (magnitude, frequency, and time) would be equivalent to the control and input this value as an SMD of 0.

The null hypotheses for our simulations were that there is no relationship between our independent variables (magnitude, frequency, and exposure time) and repair processes (viability, proliferation, differentiation, and maturation). In our models, this would present as coefficients (i.e., *a*, *b*) equal to zero. To evaluate this statistically, we defined alpha *a priori* as 0.05. The *p* values of our simulations were quantified as the number of values greater than or less than zero divided by the total number of completed simulations. For example, if the average value of *b* was 9.82 and three out of 100 completed fits produced a negative value of *b*, our *p* value would be 0.03 and this finding would be considered statistically significant. The original code for all simulations is shown in Supplementary Material S3. Fine details for the mathematical model are available in the Supplementary Methods (available here).

### 2.4. Statistical Analysis: Categorical Meta-Analyses

Since a continuous exposure meta-analysis model is not as sensitive to binary hypotheses [26], we performed categorical meta-analysis for the combinations of dependent (e.g., oligodendrocytic differentiation, and astrocytic differentiation) and independent variables (magnetic field amplitude, frequency, and exposure time) for which we did not find a continuous relation. In each case the control group had no external magnetic field exposure besides the Earth's magnetic field, and the experimental group included all cases with additional external magnetic field exposure. SMD and pooled standard deviations (SD_pool_) of outcome measures were calculated using the DerSimonian–Laird method [20]. All meta-analyses were performed using SPSS Statistics for Windows (version 29.0, IBM Corp., Armonk, NY, USA).

## 3. Results

### 3.1. Systematic Review Revealed Heterogeneity in Magnetic Field Parameters, Cell Types, and Outcome Measures for Neural Repair

**Figure 1:**
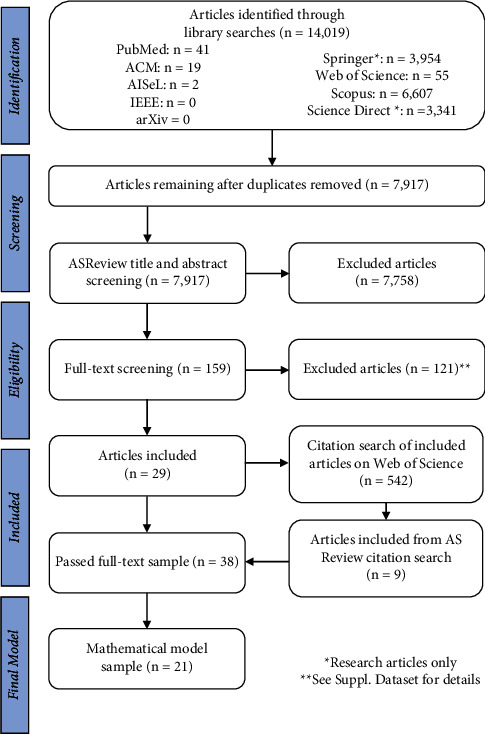
Systematic review workflow. Systematic search of databases, abstract, title, and full-text screening as well as a citation search resulted in 38 unique articles included in this review. Details on reason for exclusion at the full-text level and included article details are available in Tables S2 and S3. Of the 38 articles included, 21 had overlapping data between the investigated independent and dependent variables and were therefore included in subsequent mathematical models and meta-analyses.

From our systematic review, we identified 7,917 articles that evaluated magnetic field exposure effects on *in vitro* neural cell repair processes. Title and abstract screening via ASReview, which is a machine learning algorithm for large review screening, resulted in 159 articles eligible for full-text screening (Table S2). After screening and citation search, 38 articles were included (Figure 1 and Table S3) [27–63]. The most frequent reason for exclusion was whole animal magnetic field exposure (Table S2).

**Figure 2:**
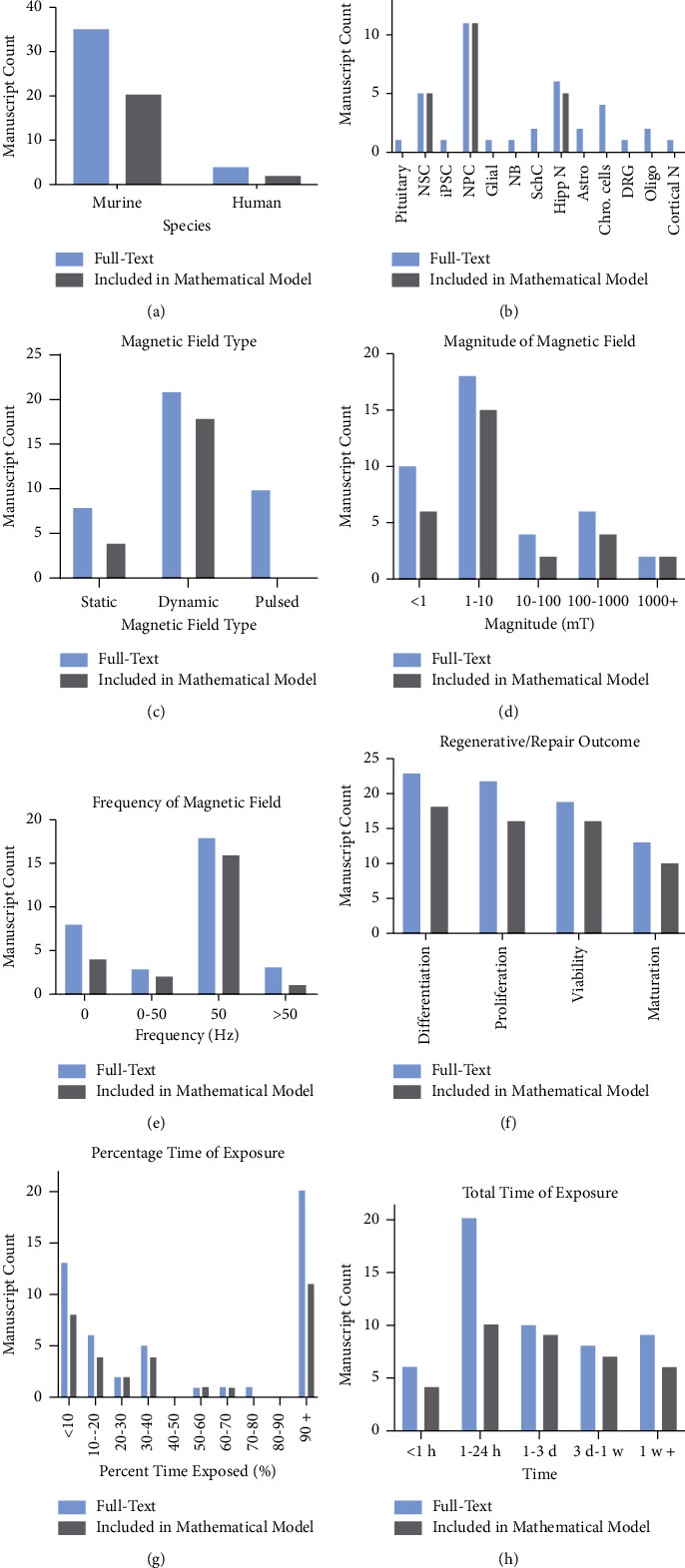
Publications aimed at studying neural repair as a function of magnetic field exposure offer a wide range of diversity in both magnetic field parameters and cell types under study. Manuscripts were counted based on if the included data fell into certain categorical variable classification. In the case of where a manuscript had data falling into multiple categories, the manuscript was counted in every category. Full-text refers to all data within the “passed full-text sample” category (*n* = 38), while mathematical model sample refers to any manuscript where at least one datapoint was included in the mathematical model (*n* = 21). See Table S3 for exclusion criteria. (a) Species of cells. (b) Cell type. NSC = neural stem cells; iPSC = induced pluripotent stem cells; NPCs = neural progenitor cells; NB = neuroblastoma; SchCs = Schwann cells; Hipp N = hippocampal neurons; Astro = astrocytes; Chro cells = chromaffin cells; DRG = dorsal root ganglia; Oligo = oligodendrocytes; Cortical N = cortical neurons. (c) Type of magnetic field. (d) Magnitude of magnetic field. (e) Frequency of magnetic field. (f) Metric of repair/regeneration studied. (g) Percent of time exposed (time exposed to magnetic field over total time of experiment and outcome measurement). (h) Total time of magnetic field exposure h = hours, d = days, and w = weeks.

Of included studies, 35 used murine cells and 4 used human cells (1 study used both; Figure 2(a)). Neural progenitor cells (*n* = 11) were the most common cell type used (Figure 2(b)). When considering magnetic field parameters, dynamic (*n* = 21, Figure 2(c)), low magnitude (1–10 mT, *n* = 30, Figure 2(d)), and 50 Hz frequency (*n* = 17, Figure 2(e)) fields were the most frequently used. For outcome variables, most studies examined how magnetic field exposure affected differentiation and proliferation (Figure 2(f)). The total time of magnetic field exposure was variable, ranging from less than one hour to over one week (Figure 2(h)). The percent time of exposure demonstrated a bimodal distribution, with one cluster of studies focusing on exposure <10% of the total experiment length (e.g., one short burst at the beginning of the experiment) while the other studies evaluated exposures that spanned nearly the entire length of the experiment (>90%) (Figure 2(g)). These findings reveal a high degree of heterogeneity in studies aimed at understanding how magnetic field exposure impacts neural cellular repair processes.

### 3.2. Included Studies Lacked Information on “Inclusion and Exclusion Criteria,” “Protocol Registration,” and “Data Access”

**Figure 3:**
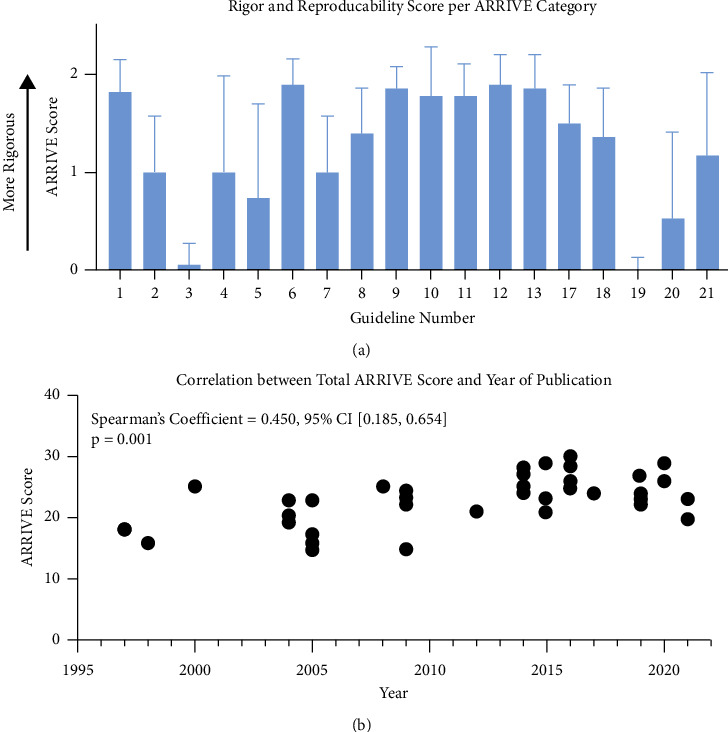
Rigor and reproducibility in studies aimed at understanding magnetic field impacts on neural repair is increasing across time. (a) ARRIVE score based on individual category, with 2 being sufficiently addressed and 0 being insufficiently addressed. 1 = study design; 2 = sample size; 3 = inclusion and exclusion criteria; 4 = randomization; 5 = blinding; 6 = outcome measures; 7 = statistical methods; 8 = experimental animals; 9 = experimental procedures; 10 = results; 11 = abstract; 12 = background; 13 = objectives; 17 = interpretation/scientific implications; 18 = generalizability/translation; 19 = protocol registration; 20 = data access; 21 = declaration of interests. Values are reported as mean ± standard deviation. Guidelines 14–16 were not used due to excluding *in vivo* studies. (b) Relationship between total ARRIVE score and publication year. Each dot represents an included manuscript.

To assess rigor and reproducibility (R&R), we scored each manuscript by ranking ARRIVE guideline categories. The maximum overall score, indicating higher rigor, is 36, and the lowest score, indicating no rigor, is zero. Included manuscripts had an average R&R of 23 ± 4 (Figure 3 and Table S4). When examining the Essential 10, “Study Design,” “Outcome Measures,” “Experimental Procedures,” and “Results” were generally “sufficiently reported.” On the other hand, “Inclusion and Exclusion Criteria” was “insufficiently reported.” Of the additional guidelines, “Abstract,” “Background,” and “Objectives” were “sufficiently reported,” while “Protocol Registration” and “Data Access” were scored as “insufficiently reported” across all studies (Figure 3(a)). We also evaluated the correlation between the ARRIVE score and publication year (Figure 3(b)). We found a moderate, positive correlation, suggesting that R&R is slowly improving over time. Additional description of the qualitative factors driving these findings is presented in the Supplementary Results (available here).

### 3.3. Mathematical Modeling Revealed Magnetic Field Magnitude Has a Stable, Positive Relationship with Neural Progenitor Cell (NPC) Viability

**Figure 4:**
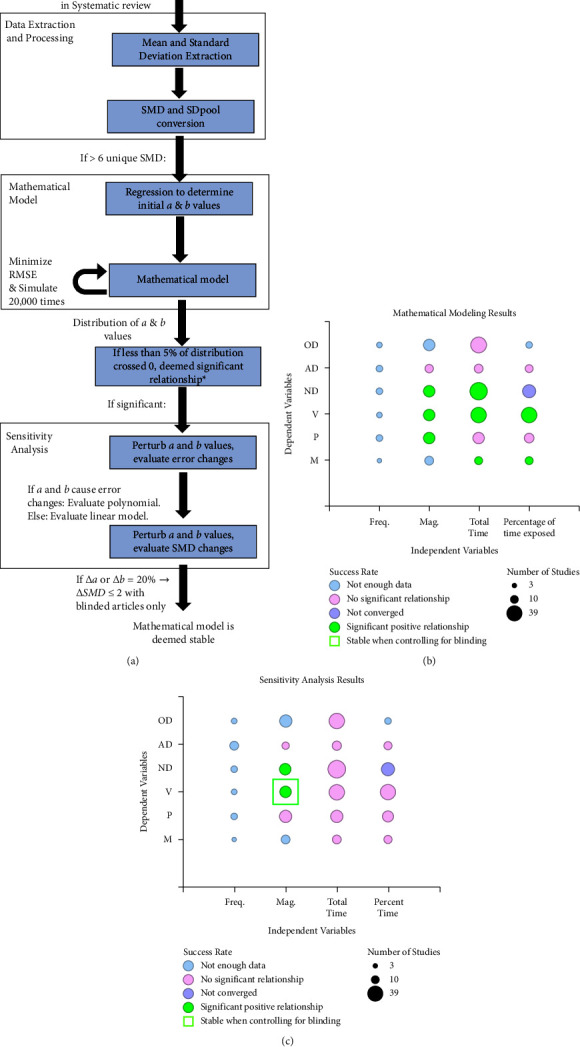
Mathematical modeling and sensitivity analyses reveal NPC viability has a robust relationship with magnitude of magnetic field exposure. (a) Mathematical modeling and sensitivity analyses workflow. SMD = standardized mean difference; RMSE = root mean squared error; SD_pool_ = pooled standard deviation. ^*∗*^For example, if the average value of *a* was 3 and only 3% of simulated *a* values were negative, we would say with confidence that there is a relationship between the independent and dependent variables. (b) Results from mathematical modeling. OD = oligodendrocytic differentiation; AD = astrocytic differentiation; ND = neuronal differentiation; V = neural stem/progenitor cell (NPC) viability; P = NPC proliferation; M = NPC maturation; Percent time = percentage of time exposed. Of note, no negative relationships were observed in our modeling results. (c) Sensitivity analysis results.

The included studies offered varying ranges of properties and exposure schedules of magnetic fields as well as a variety of metrics to quantify repair and regenerative processes. In categorical (standard) meta-analyses, conditions can be divided into two groups: experiment and control. However, the exposure schedules in the dataset we analyzed were not categorical but, instead, were continuous. Therefore, for the first pass through the dataset, we tested a series of mathematical models, similar in structure to continuous exposure meta-analyses, [26] in order to (1) extract the continuous relationship between exposure and regenerative processes and (2) derive information that cannot be extracted from individual studies. Specifically, our models simulated the effects of magnetic field parameters (magnitude, frequency, total time of exposure, percentage of time exposed) on neural repair metrics (viability, proliferation, differentiation, and maturation). Studies that did not contain neural repair outcome measures or that used pulsed magnetic fields were excluded to simplify the model input. Twenty-one articles met these guidelines and were included in subsequent analyses (the modeling workflow is displayed in Figure 4(a); the model input is presented in Table S5).

After quantification, six distinct dependent variables were tested: neuronal, astrocytic, and oligodendrocytic differentiation, as well as neural progenitor cell (NPC) maturation, proliferation, and viability. Considering the independent variables (magnitude, frequency, total time exposed, and percentage of time exposed), a total of 24 combinations were possible for simulation. However, nine combinations did not have enough data to run a simulation (Figure 4(b)). Thus, 15 models were simulated (Figure 4(a) and Tables S6 and S7).

Based on our models, astrocytic and oligodendrocytic differentiation did not have a significant relationship with magnitude, frequency, total time exposed, or percentage of time exposed to the magnetic field (Figure 4(b)). Changes in time-based variables were positively correlated with NPC maturation, while NPC proliferation was positively correlated with increasing magnitude. NPC viability had significant, positive correlations with respect to magnitude, total time exposed, and percentage of time exposed. Lastly, neuronal differentiation was positively correlated with magnitude and total time exposed. Taken together, these simulations suggest that magnetic field exposure may exert positive effects on NPC maturation, proliferation, differentiation, and viability.

To assess the robustness of our simulations, we performed sensitivity analyses. First, we analyzed how changes in *a* and *b* values affected the root mean squared error (RMSE). For most cellular repair processes, we found that both *a* and *b* affected the RMSE. Therefore, we only modeled polynomial relationships for the entirety of the sensitivity analyses. For all considered combinations, RMSE effects were stable. However, unlike the statistically significant relationships observed in fitting to mathematical models, sensitivity analyses revealed a lack of robustness across many of the observed relationships, as quantified by a change in SMD >2 (Figure 4(c)). Models assessing the relationship between NPC viability and neuronal differentiation with respect to magnitude were stable. The total time exposed models generally showed moderate instability (SMD of 2–10 for both *a* and *b*), while the percent time models showed extreme instability (SMD of ∼10^5^ for *a* and ∼10^3^ for *b*).

Using the previous data from our R&R analysis, we repeated the mathematical models incorporating only those that explicitly blinded their experiments (i.e., a score of “2” in the 5^th^ ARRIVE Guideline Category, “Blinding”; *n* = 6). Of note, astrocytic differentiation and NPC maturation simulations could not be run because there was not enough data. Neuronal differentiation, oligodendrocytic differentiation, and NPC proliferation only had enough independent variables for “total time exposed,” while NPC viability had enough data for both “total time exposed” and “magnitude” simulations. Oligodendrocytic differentiation and NPC proliferation did not have a relationship with time exposed to the magnetic field. Changes in total time exposed were positively correlated with neuronal differentiation and NPC viability, while changes in magnitude were positively correlated with NPC viability. However, sensitivity analyses revealed that only the relationships between NPC viability with respect to total time exposed and magnitude were stable. Our findings indicate there is a positive relationship between magnetic field magnitude and NPC viability (Figure 4(c); raw data shown in Figure S1).

### 3.4. Meta-Analyses Reveal That NPC Proliferation Is Increased, Whereas Astrocytic Differentiation Is Decreased by Magnetic Field Exposure

Based on our mathematical modeling results, oligodendrocytic and astrocytic differentiation as well as NPC proliferation were not modulated by changes in magnitude, frequency, or time of exposure to a magnetic field. Because there were no apparent effects of these heterogeneous exposure parameters, we next compiled these datasets to further characterize whether there were any effects of magnetic field exposure via categorical meta-analysis.

**Figure 5:**
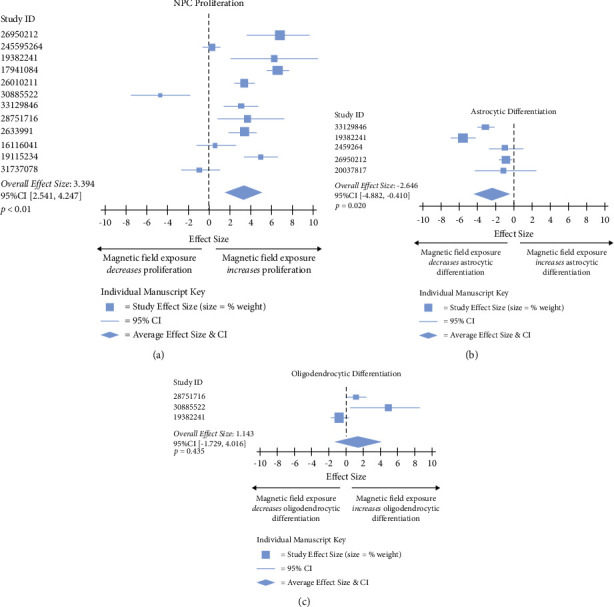
Meta-analyses revealed that magnetic field exposure increases NPC proliferation and decreases astrocytic differentiation. (a) Meta-analysis assessing neural stem/progenitor cell (NPC) proliferation from included studies. (b) Meta-analysis assessing astrocytic differentiation from included studies. (c) Meta-analysis assessing oligodendrocytic differentiation.

Categorical meta-analysis revealed that NPC proliferation was increased with magnetic field exposure (Figure 5(a)). Conversely, astrocytic differentiation was decreased (Figure 5(b)). Lastly, oligodendrocytic differentiation was not impacted, although this analysis is likely underpowered, as there were only three independent studies included (Figure 5(c)). Collectively, these findings suggest that magnetic field exposure may amplify the regenerative activity of some cell types (NPCs) while attenuating others (astrocytes).

## 4. Discussion

Magnetic field stimulation represents a promising noninvasive regenerative therapeutic strategy. However, more information is needed to understand the physiological versus pathological effects of magnetic fields on cell responses as we seek to define more targeted and specific protocols in the clinic. To improve our understanding of magnetic field impacts on neural regeneration, we cataloged all published studies aimed at understanding the impact of magnetic field exposure on neural repair *in vitro* through a literature review. Drawing from the data synthesized in our review, mathematical models revealed that increasing magnetic field magnitude increases NPC viability. Our meta-analyses further revealed that magnetic field stimulation increases NPC proliferation while decreasing astrocytic differentiation.

In considering the magnetic field parameters tested (Figures 2(d) and 2(e)), we found that the included studies primarily attempted to simulate everyday exposures. For example, 50 Hz fields are reflective of those emitted by cell phones and power lines, and there are some human studies indicating detrimental effects from exposure [64–66]. Specific parameters such as time of exposure lacked a clear rationale. When we sought to understand the wide range of exposure times investigated (Figures 2(g) and 2(h)), we found that authors rarely reported how and why exposure schedules were chosen. Future work would benefit from a more clearly presented exposure schedule rationale and/or systematic evaluation of how magnetic field parameters impact repair/regenerative outcomes of interest.

In 2010, the ARRIVE Guidelines were first published to address the rigor and reproducibility crisis in the biological sciences [67]. Based on the positive correlation seen between publication year and ARRIVE Score, progress is clearly being made (Figure 3(b)). However, in the body of literature aimed at understanding magnetic field effects on neural repair and regeneration, there are still many areas needing improvement, including declaration of inclusion and exclusion criteria and *a priori* protocol registration. Given the high heterogeneity of study parameters tested (i.e., magnetic field specifications) and the associated knowledge gap regarding ideal exposure conditions, increased attention to these parameters is essential.

While meta-analysis is standard in systematic reviews, here, we applied mathematical modeling to better incorporate the heterogeneity present within the included studies. Our mathematical modeling results revealed that NPC viability improved with increasing magnetic field magnitude. This finding is consistent with several independent studies and cell types [28, 44, 68–71]. Since neurons themselves lack a robust ability to regenerate, maintaining NPC viability is critically important to neural repair [72]. Our findings suggest one mechanism of therapeutic benefit provided by magnetic fields is the protection of NPC. There is some evidence suggesting these effects may be modulated by magnetic field and voltage-gated ion channel interactions, leading to cell membrane stabilization [73, 74]. Others have suggested this improved viability may be modulated by cellular magnetoelectric materials that facilitate temperature control [75, 76]. Our findings here suggest that examination of these mechanisms to both optimize the therapeutic potential and better understand how magnetic field stimulation affects NPC viability is a promising direction for future research.

Our mathematical modeling suggested there were no effects of magnetic field magnitude, frequency, or exposure time on oligodendrocytic differentiation, astrocytic differentiation, or NPC proliferation. Thus, we evaluated the impact of magnetic stimulation, independent of magnetic field parameters, on repair features by performing categorical meta-analyses. Our meta-analyses indicated that magnetic field exposure may increase NPC proliferation while decreasing astrocytic differentiation. Astrocytes play a critical role in neural repair/regeneration by activating NPC and creating an environment that allows for neuronal maturation and proliferation [77]. However, overactive astrocytes can lead to glial scarring and serve as a detriment to repair [78]. In other studies evaluating the effect of magnetic fields on astrocytes, responses to magnetic fields appeared to be dependent both on endogenous cell features (i.e., the cell cycle phase), as well as exogenous exposure features (e.g., whether the plane of applied magnetic field was parallel versus perpendicular to the plane of the monolayer) [79]. The differential response among varying cellular repair processes to magnetic field exposure may explain the inconsistencies in the benefit-to-risk ratio. Most likely, there is a “goldilocks effect,” in which a yet-to-be-identified ideal magnetic field exposure increases NPC viability and proliferation, while preserving the appropriate level of astrocyte activity [80]. As such, future studies are needed to systematically evaluate how these three parameters of magnetic field exposure—magnitude, frequency, and time—modulate regenerative/repair cascades.

We note that none of the studies controlled for the effects of Earth's static magnetic field (∼50 *μ*T), and all the studies reported magnetic field amplitude but not vector data. The lack of vectorial magnetic field data makes our mathematical model insensitive to magnetic field effects that occur when amplitudes are on the order of Earth's magnetic field or lower. We hypothesize that biological systems that evolved in ∼50 *μ*T Earth magnetic field would be sensitive in the 1–100 *μ*T range. As such, it is important for the next generation of experiments to carefully compensate for the Earth's magnetic field.

Although our analyses here add to a growing body of literature assessing the therapeutic potential of magnetic fields, they have limitations. First, all the included studies were performed using *in vitro* monocultures. There was a diversity of cell types, species, outcome measures, and exposures included in this review. More specifically, the variability in the selected times and schedule of exposure made integration of the protocols challenging. In addition, interest in pulsed electromagnetic fields is increasing due to cell phone emissions, but due to the fact that only a single magnetic field magnitude could be input into our model, it was impossible to consider these potential effects [81]. Given that our review focused on *in vitro* systems, we were also not able to take into account how variations of subpopulation electrical activity may affect magnetic field efficacy. It is also possible that there were impacts of recency here that were not fully elucidated (e.g., 1 hour exposure then sample collection 24 hours later versus 1 hour exposure and immediate sample collection). Given this variability, our models cannot be taken as an exact representation of existing relationships, but rather, a general, binary trend. It is also worth noting that most simulations and meta-analyses contained a relatively low number of studies. Therefore, our analyses were generally underpowered. The original data input was usually clustered around a few points (e.g., frequency was centered around 0 and 50 Hz). This observation further highlights the need for more studies to evaluate uncommon parameters such that the therapeutic window of magnetic fields can be better delineated. Given these considerations, our analyses provide guidance to improve the design and reporting of magnetic field studies for neural cell repair and regeneration, as well as mechanistic understanding.

Magnetic field exposure is and will continue to be a part of everyday life, and the potential for a noninvasive therapeutic will likely increase with our understanding of magnetic field effects on repair processes. Future steps should include increased emphasis on systematic evaluation of magnetic field exposure that impacts NPC viability and proliferation and astrocytic differentiation in original research studies as well as the underlying mechanisms dictating these phenotypes. By increasing our understanding of the nuances of magnetic field effects on neural regeneration, we will move closer towards robust and safe therapies for the patients they aim to treat.

## Data Availability

All data analyses are included in this manuscript and its associated supplement. The original code is available in Supplementary Material S3 and is on the below GitHub link. In addition, an Excel version of all supplemental tables has been uploaded to GitHub. Since this is a review, the original data input into the models and meta-analyses are available in the original publications as cited in this work https://github.com/gggilmer/NeuralRegenMagFieldReview?search=1 [82].

## Supplementary Materials

Supplementary Materials Supplementary Material S1: PRISMA checklist. Supplementary Material S2: MOOSE Checklist. Supplementary Material S3: original code used for simulations. Figure S1: mathematical modeling and sensitivity analyses reveal NPC viability has a robust relationship with magnitude of magnetic field exposure. Raw SMD data of viability versus magnitude. These data are used in Figure 4(c). Table S1: PECO inclusion criteria and search terms. List of population, exposure, comparison, and outcomes used to define our inclusion criteria and the exact search terms used to identify articles for screening. Table S2: full-text screening results and reason for exclusion. At the full-text screening level when articles were excluded, the reason was documented and reported here. The most common reason for exclusion was full animal exposure. Table S3: descriptions of included text outcome variables: study title (manuscript title), cell type, magnitude (mT), field type, frequency (Hz), and outcome variable. Table S4: ARRIVE Guideline Scoring on a per article basis. *X*-axis: the numbers correspond to the ARRIVE guideline ranking on a scale from 0 to 2. Since there were two reviewers, if the ARRIVE guideline ranking varied by only 1, the rankings were averaged. If different by 2 (i.e., one reviewer chose 0 and one chose 2), the article was discussed until a consensus was reached. Table S5: article counts used for simulations. *X*-axis: number of manuscripts, number of datapoints, average number of datapoints per manuscript (rounded to nearest integer), standard deviation (rounded to nearest integer), median, number of unique independent variable (e.g., if the dataset is 0, 0, 1, 2, there are 3 unique independent variable points). *Y*-axis: maturation, proliferation, viability, neuro/astrocyte/oligodendrocyte differentiation. Table S6: variable definition for simulations. *X*-axis: class (regenerative dependent variables), up/downregulate (an increase in a cellular marker result in an increase of a regenerative dependent variable), variable (specific cellular markers/characteristics), description (of the specific cellular markers), applicable studies (number of manuscripts that utilize a specific variable). ^*∗*^ = characteristic is included in two categories (regenerative dependent variable). ^*∗∗*^ = characteristic is included in three categories (regenerative dependent variable). Table S7: inputs for quadratic and linear simulations *a* and *b* guesses. Column titles: variable name (categorical dependent variable), linear/polynomial (fit of raw data), independent variable (magnetic field parameter), maximum RMSE over 10–200% B (constant A) (largest value obtained of the RMSE by varying one coefficient (A or B) from 10 to 200%), maximum RMSE over 10–200% A (constant B). Monoatomic refers to a polynomial that is increasing *or* decreasing. Polynomial refers to a polynomial that increases and decreases.
