# Supplementary material for: Mapping the Landscape of Magnetic Field Effects on Neural Regeneration and Repair: A Combined Systematic Review, Mathematical Model, and Meta-Analysis
Source: J Tissue Eng Regen Med. 2023 Sep 21;2023:5038317. doi: 10.1155/2023/5038317 (PMC11918650; doi:10.1155/2023/5038317)
Supplement: Supplementary Materials — Supplementary Material S1: PRISMA checklist. Supplementary Material S2: MOOSE Checklist. Supplementary Material S3: original code used for simulations. Figure S1: mathematical modeling and sensitivity analyses reveal NPC viability has a robust relationship with magnitude of magnetic field exposure. Raw SMD data of viability versus magnitude. These data are used in Figure 4(c). Table S1: PECO inclusion criteria and search terms. List of population, exposure, comparison, and outcomes used to define our inclusion criteria and the exact search terms used to identify articles for screening. Table S2: full-text screening results and reason for exclusion. At the full-text screening level when articles were excluded, the reason was documented and reported here. The most common reason for exclusion was full animal exposure. Table S3: descriptions of included text outcome variables: study title (manuscript title), cell type, magnitude (mT), field type, frequency (Hz), and outcome variable. Table S4: ARRIVE Guideline Scoring on a per article basis. X-axis: the numbers correspond to the ARRIVE guideline ranking on a scale from 0 to 2. Since there were two reviewers, if the ARRIVE guideline ranking varied by only 1, the rankings were averaged. If different by 2 (i.e., one reviewer chose 0 and one chose 2), the article was discussed until a consensus was reached. Table S5: article counts used for simulations. X-axis: number of manuscripts, number of datapoints, average number of datapoints per manuscript (rounded to nearest integer), standard deviation (rounded to nearest integer), median, number of unique independent variable (e.g., if the dataset is 0, 0, 1, 2, there are 3 unique independent variable points). Y-axis: maturation, proliferation, viability, neuro/astrocyte/oligodendrocyte differentiation. Table S6: variable definition for simulations. X-axis: class (regenerative dependent variables), up/downregulate (an increase in a cellular marker result in an increase of a rege [file 5038317.f1.zip › suppMatS3_feb28_2023.pdf]

# Code for "Mapping the landscape of magnetic field effects on neural repair: a systematic review, mathematical model, and meta-analysis"

## Written by: Meghan McGraw and Gabrielle Gilmer

# Imports

## Import packages into Python

In [1]:
```
import numpy as np
%matplotlib inline
import matplotlib.pyplot as plt
from scipy.integrate import odeint
from scipy.optimize import minimize
import pandas as pd
import seaborn as sns
import os
import random
import statsmodels.api as sm
import csv
```

# 3D Plot

## Input: Excel/ CSV data file

## Output: 3D plot of SMD versus 2 independent variables

## Return value: matrix of mag, freq, SMD, SDpool, characteristic, cell_type

## This function generates a 3D plot of the SMD and two independent variables for a given regenerative characteristic.

In [2]:
```
def plot_raw_data(file):
    #  Import data from CSV
    dat = pd.read_csv(file, index_col = None)
    dataMat = pd.DataFrame(dat, columns= ['Title', 'CellCategory', 'SpecificOutcomeVari

    # sort -- in order for the ODE solver to run the data on a certain independent vari
    #  in ascending/ descending order based on the independent variable
    if choice == 'magnitude':
        dataMat = dataMat.sort_values('Magnitudge')
        x = dataMat.Magnitudge
```

```
        xl = 'Magnitude (mT)'
    elif choice == 'frequency':
        dataMat = dataMat.sort_values('Freq')
        xl = 'Frequency (Hz)'
        x= dataMat.Freq
    elif choice == 'total_time':
        dataMat = dataMat.sort_values('TotalTimeExposed')
        x= dataMat.TotalTimeExposed
        xl = 'Total Time Exposed (hours)'
    else:
        dataMat = dataMat.sort_values('pertimeExposed')
        x = dataMat.pertimeExposed
        xl = 'Percent Time Exposed (%)'

    # split up into different columns
    Title = dataMat.Title
    mag = dataMat.Magnitudge
    freq = dataMat.Freq
    time = dataMat.TotalTimeExposed
    percent = dataMat.pertimeExposed
    SMD = dataMat.SMD
    SDpool = dataMat.Sdpool

    # Plot raw data -- 3D plots -- generate all possible 2 independent variable combina
    # SMD as a function of 2 independent variables

    choices = ['magnitude', 'frequency', 'total_time', 'percent']
    choices.remove(choice)

    for c in choices:
        fig = plt.figure(figsize=(9, 9))
        plt.rcParams.update({'font.size': 14})
        ax = plt.axes(projection='3d')

        if c == 'magnitude':
            y = mag
            yl = 'Magnitude (mT)'
        elif c == 'frequency':
            y = freq
            yl = 'Frequency (Hz)'
        elif c == 'total_time':
            y = time
            yl = 'Total Time Exposed (hours)'
        else:
            y = percent
            yl = 'Percent Time Exposed (%)'

        zline = SMD
        xline = x
        yline = y
        ax.plot3D(xline, yline, zline, 'ro')
        ax.set_xlabel(xl)
        ax.set_ylabel(yl)

        ax.set_zlabel('SMD')

        #add title
        plt.title(name + '_' + choice + '_' + c + " Raw Data")

        plt.savefig('Final_data/3D_Plot/' + name + '_' + choice + '_' + c + '3D_Plot.pn
```

```
    #return pertinent data
    return [mag, freq, time, percent, SMD, SDpool]
```

# Linear/nonlinear regression model

## Input: x_value (independent variable values of magnitude, frequency, total time exposed, or percent time exposed), y_value (SMD), fit

## Return: slope and y-intercept (linear) or polynomial and linear coefficients (polynomial)

## This function uses numpy polyfit to generate first guess slope and y-intercept (linear) or the polynomial and linear coefficients (polynomial) for the mathematical modeling

In [3]:

```
def regression(x_value, y_value, fit):
    x_value = np.array(x_value.tolist())
    y_value = np.array(y_value.tolist())
    fig5, ax5 = plt.subplots()
    ax5.plot(x_value, y_value,'ro',label='Data for ' + name)

    if fit == 'Polynomial':
        p = np.polyfit(x_value, y_value, 2)
    else:
        p = np.polyfit(x_value, y_value, 1)
    ax5.plot(x_value, np.polyval(p, x_value),color='blue',label='Best Fit for ' + name)
    plt.title('A = ' + str(round(p[0], 7)) + " B = " + str(round(p[1], 7)))

    ax5.legend()

    fig5.savefig('Final_data/Regression/' + name + "_" + choice + "_" + fit +\
            '_Regression.png', dpi=300, bbox_inches='tight');

    #a and b values returned
    print("a:")
    print(p[0])
    print("b:")
    print(p[1])
    return p
```

# Auxillary Functions

## Stepwise differential equation solver and root-mean-square error functions

In [4]:

```
# set up model
def dSMDdFq(SMD, fq, a, b):
    dSMDdFq = a*fq + b
```

```
    return dSMDdFq

#root mean square error

# define RMSE
# steps
def RMSE(params):
    a, b = params;
    ODE_output = odeint(dSMDdFq, SMD_0, steps, args = (a,b));
    err = ExpData -ODE_output
    SSE = np.sum(err**2)
    RMSE = np.sqrt(SSE)
    return RMSE
```

# Run model function

## Input: mag, freq, time, percent, SMD, SDpool, fit (linear/polynomial)

## Returns a and b matrix, fit

## Produces 4 plots after running 10,000 trials to guess the a and b values of the data, showing the distribution of a, b, and RMSE values across trials

In [5]:

```
def run_model(mag, freq, time, percent, SMD, SDpool, fit):

    print(name, choice, fit)
    # Defines the size of the vector
    sizeSMD = len(SMD)
    # Defines the index size
    index = sizeSMD
    # Sets initial value
    global SMD_0
    SMD_0 = 0

    # Pulls dosage values to evaluate
    # evaluate based on which independent variable is chosen
    global steps
    if choice == 'frequency':
        steps = freq
    elif choice == 'magnitude':
        steps = mag
    elif choice == 'total_time':
        steps= time
    else:
        steps= percent


    # Sets initial guesses based on trendline model from Excel
    if fit == "Polynomial":
        p0 = [a_guess_poly, b_guess_poly]
    else:
        p0 = [a_guess_linear, b_guess_linear]
```

```
    # sets counter equal to zero
    f = 0

    # Number of values to be generated for each data point
    nVal = 100
    # Generates a vector of the different values of a, b, and RMSE generated by the sim
    a = np.zeros(num_trials+1)
    b = np.zeros(num_trials+1)
    RMSE_val = np.zeros(num_trials+1)

    # generates a figure for the simulations to be plotted on
    fig1, ax1 = plt.subplots()

    plt.rcParams.update({'font.size': 14});

    # labeling purposes
    if choice == 'frequency':
        s = "Frequency (Hz)"
    elif choice == 'magnitude':
        s = "Magnitude (mT)"
    elif choice == 'total_time':
        s = 'Total Time Exposed (hours)'
    else:
        s = 'Percent Time Exposed (%)'

    ax1.set_xlabel(s)


    ax1.set_ylabel("SMD of " + name + ' (' + fit + ")")

    # Simulates using different initial conditions
    for j in np.arange(1, num_trials,1):
        # Generates ExpData vector
        global ExpData
        ExpData = np.arange(0,index,1)
        # Sets iteration markers
        m = 0
        for x in np.arange(0,index,1):
            # Generates a normal distribution based on the SMD and SD from that specifi
            RandGen = np.random.normal(SMD[m], SDpool[m], nVal)
            # Randomly pulls a value from that normal distirbution to input into model
            randCho = random.choice(RandGen)
            # Assigns random value into experimental data
            ExpData[m] = randCho
            # Iterates m
            m = m + 1


        # Minimizes RMSE uses initial data
        sol = minimize(RMSE, p0);
        # Enters generated value into the vector to track all possible a and b values f
        if fit == "Polynomial":
            a_temp = sol.x[0]
        else:
            a_temp = 0
        b_temp = sol.x[1]
        # Generates model data based on a and b values
        modelData = odeint(dSMDdFq, SMD_0, steps, args = (a_temp,b_temp));
        # Stores a, b, and RMSE value
        a[f] = a_temp;
```

```
        b[f] = b_temp;
        RMSE_val[f] = sol.fun
        # Increases counter
        f = f+1
        # Plots simulation
        ax1.plot(steps, modelData, 'b-')

    #saves models as a figure to folder
    fig1.savefig('Final_data/Simulation_Model/' + name + "_" + choice + "_" + fit + '_S


    # Plots generated a values
    fig2, ax2 = plt.subplots()
    ax2.hist(a, bins = 50);
    plt.rcParams.update({'font.size': 14});
    plt.title("Distribution of a values " + name + ", " + choice + ' (' + fit + ")")
    ax2.set_xlabel("a")
    ax2.set_ylabel("Counts")

    fig2.savefig('Final_data/A_Value/' + name + "_" + choice + "_" + fit + '_SimAValue.

    # Plots generated b values
    fig3, ax3 = plt.subplots()
    ax3.hist(b,bins = 50);
    plt.rcParams.update({'font.size': 14});
    plt.title("Distribution of b values " + name + ", " + choice + ' (' + fit + ")")
    ax3.set_xlabel("b")
    ax3.set_ylabel("Counts")

    fig3.savefig('Final_data/B_Value/' + name + "_" + choice + "_" + fit + '_SimBValue.

    # Plots generated RMSE values
    fig4, ax4 = plt.subplots()
    ax4.hist(RMSE_val,bins = 50);
    plt.rcParams.update({'font.size': 14});
    plt.title("Distribution of RMSE values " + name + ", " + choice + ' (' + fit + ")")
    ax4.set_xlabel("RMSE")
    ax4.set_ylabel("Counts")

    fig4.savefig('Final_data/RMSE_Value/' + name + "_" + choice + "_" + fit + '_SimRMSE

    with open('Final_data/A_matrix/' + name + "_" + choice + "_" + fit +'.csv', 'w', ne
      csv_out = csv.writer(csv_1)
      csv_out.writerows([a[index]] for index in range(0, len(a)))

    with open('Final_data/B_matrix/' + name + "_" + choice + "_" + fit +'.csv', 'w', ne
      csv_out = csv.writer(csv_2)
      csv_out.writerows([b[index]] for index in range(0, len(b)))

    return [a, b, fit]
```

# P-value function

## Input: a, b, title (characteristics of dataset from filename), fit

## Output: values and probabilities of a and b

# Calculates the probability of the a and b values actually being 0 from the simulation-produced a and b values

In [6]:

```
def calc_p_value(a, b, title, fit):
    print(title)
    # Print the average value for both paramters
    a_avg = sum(a.sum())/len(a)
    b_avg = sum(b.sum())/len(b)

    print('The average value for the a parameter is %.4f.' % a_avg)
    print('The average value for the b parameter is %.4f.' % b_avg)

    # Calculate the p value for a
    f = 0
    n = 0
    sizeA = len(a)-1
    for j in np.arange(1,sizeA,1):
        if a.iloc[f][0] >= 0:
            n = n +1
        f = f+1

    a_pvalue = (sizeA-n)/sizeA

    #modification--for value at 0, probability is 1
    #if negative, the value needs to be inverted
    if a_avg<0:
        a_pvalue = 1 - a_pvalue

    if a_avg == 0:
        a_pvalue = 1

    # Calculate p value for b
    g = 0
    m= 0
    sizeB = len(b)-1
    for k in np.arange(1,sizeB,1):
        if b.iloc[g][0] >= 0:
            m = m+1
        g = g+1

    #modification
    b_pvalue = (sizeB - m)/sizeB

    #modification
    if b_avg<0:
        b_pvalue = 1 - b_pvalue

    if b_avg == 0:
        b_pvalue = 1


    # Print both p values
    print('The p-value for the a parameter is %.4f.' % a_pvalue)
    print('The p-value for the b parameter is %.4f.' % b_pvalue)


    #make 2D list, exported as CSV file containing the a, b, and p values
    lst=[['', 'average value', 'p-value'], ['a', a_avg, a_pvalue], ['b', b_avg, b_pvalu
    #print(lst)
```

```
    with open('Final_data/p_values/' + title + '_' + fit +'.csv', 'w', newline='') as c
        csvWriter = csv.writer(csv_1,delimiter=',')
        csvWriter.writerows(lst)
    return [a_pvalue, b_pvalue, a_avg, b_avg]
```

# Call functions appropriately

## plot_raw_data(), regression(), run_model()

## Iterate through all datasheets and independent variables to produce plots and calculations aforementioned

In [7]:

```
names = ['Astrocyte', 'Neuron', 'Oligodendrocyte', 'Differentiation', 'Growth', 'Viabil
cho = ['magnitude', 'frequency', 'total_time','percent']


#iterate through each category
for name in names:
    # iterate through each independent variables
    for c in cho:
        file_name = name
        num_trials = 20000
        choice = c

        #generates 3D plot
        file = 'Data/' + file_name+".csv"

        #generates 3D plot
        x = plot_raw_data(file)

        #guess a, b, generates a, b, and RMSE plots

        #find a and b guess values, generates regression coefficients and plot (poly)

        if choice == 'frequency':
            r = regression(x[1], x[4], 'Polynomial')
        elif choice == 'magnitude':
            r= regression(x[0], x[4], 'Polynomial')
        elif choice == 'total_time':
            r= regression(x[2], x[4], 'Polynomial')
        elif choice == 'percent':
            r= regression(x[3], x[4], 'Polynomial')
        a_guess_poly = r[0]
        b_guess_poly = r[1]

        y = run_model(x[0], x[1], x[2], x[3], x[4], x[5], 'Polynomial')


        #guess a, b, generates a, b, and RMSE plots

        #find a and b guess values, generates regression coefficients and plot (linear)
        if choice == 'frequency':
            r = regression(x[1], x[4], 'Linear')
        elif choice == 'magnitude':
            r= regression(x[0], x[4], 'Linear')
```

```
        elif choice == 'total_time':
            r= regression(x[2], x[4], 'Linear')
        elif choice == 'percent':
            r= regression(x[3], x[4], 'Linear')
        a_guess_poly = r[0]
        a_guess_linear = r[0]
        b_guess_linear = r[1]

        #guess a, b, generates a, b, and RMSE plots
        z = run_model(x[0], x[1], x[2], x[3], x[4], x[5], 'Linear')
```

```
a:
6.117853903605512e-05
b:
-0.0014733007441670443
Proliferation frequency Polynomial
a:
0.007104961274877871
b:
1.412203843346335
Proliferation frequency Linear
a:
-2.8741712924567723e-05
b:
0.01009976707969968
Proliferation total_time Polynomial
<ipython-input-5-b8bd61c82b24>:130: RuntimeWarning: More than 20 figures have been opene
d. Figures created through the pyplot interface (`matplotlib.pyplot.figure`) are retaine
d until explicitly closed and may consume too much memory. (To control this warning, see
the rcParam `figure.max_open_warning`).
  fig4, ax4 = plt.subplots()
a:
-0.00018515520667151397
b:
1.7415373362685553
Proliferation total_time Linear
a:
8.893052299099923
b:
-9.122849327889188
Proliferation percent Polynomial
a:
0.6573715007115367
b:
1.4082848489172644
Proliferation percent Linear
```

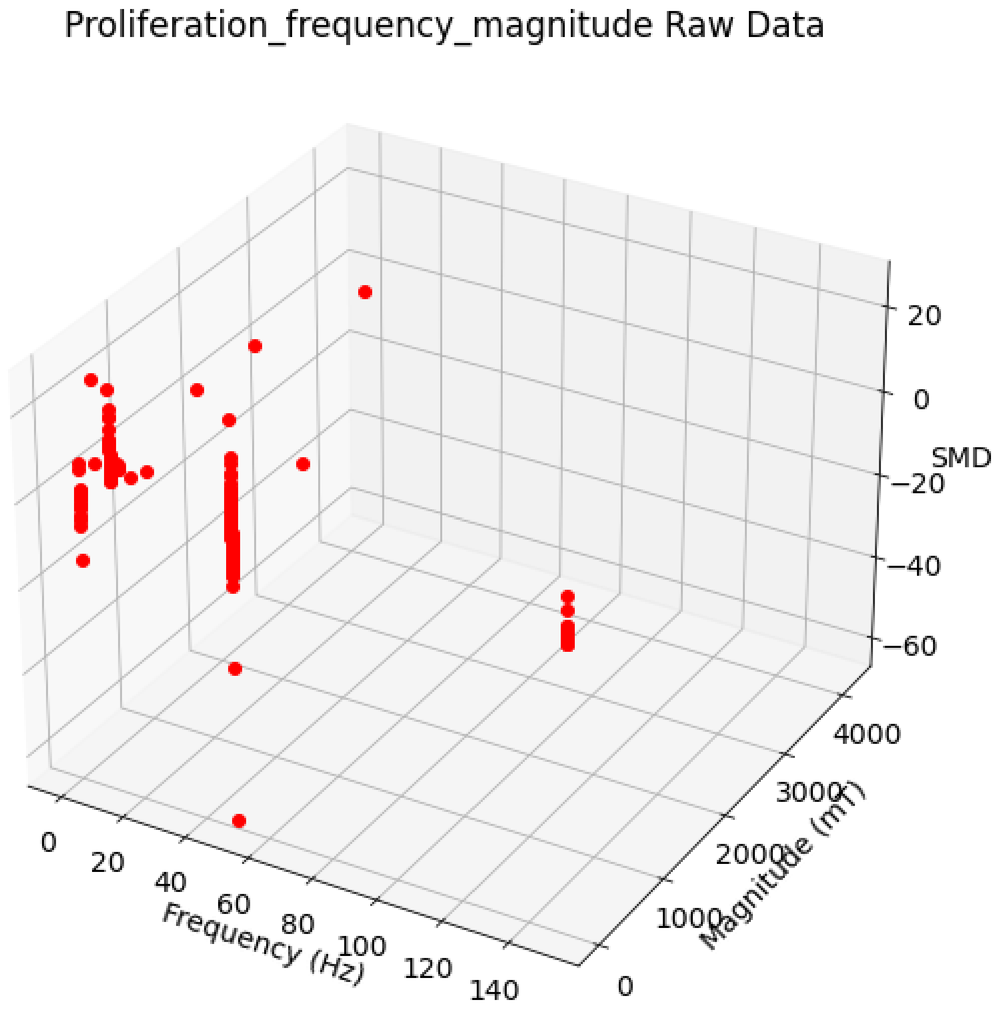
Proliferation_frequency_magnitude Raw Data
Frequency (Hz)
0
20
40
60
80
100
120
140
Magnitude (mT)
0
1000
2000
3000
4000
SMD
20
0
−20
−40
−60

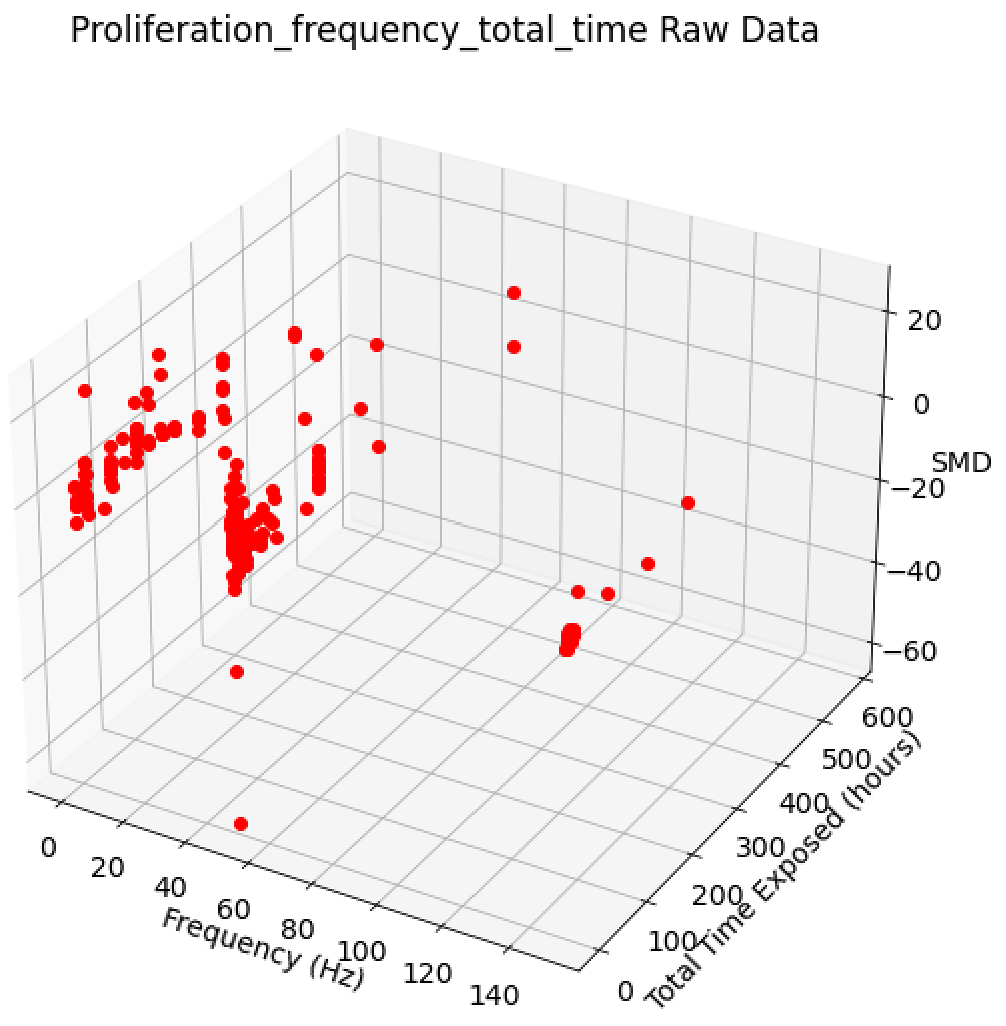
Proliferation_frequency_total_time Raw Data
20
0
SMD
−20
−40
−60
600
500
400
300
200
100
0
Total Time Exposed (hours)
0
20
40
60
80
100
120
140
Frequency (Hz)

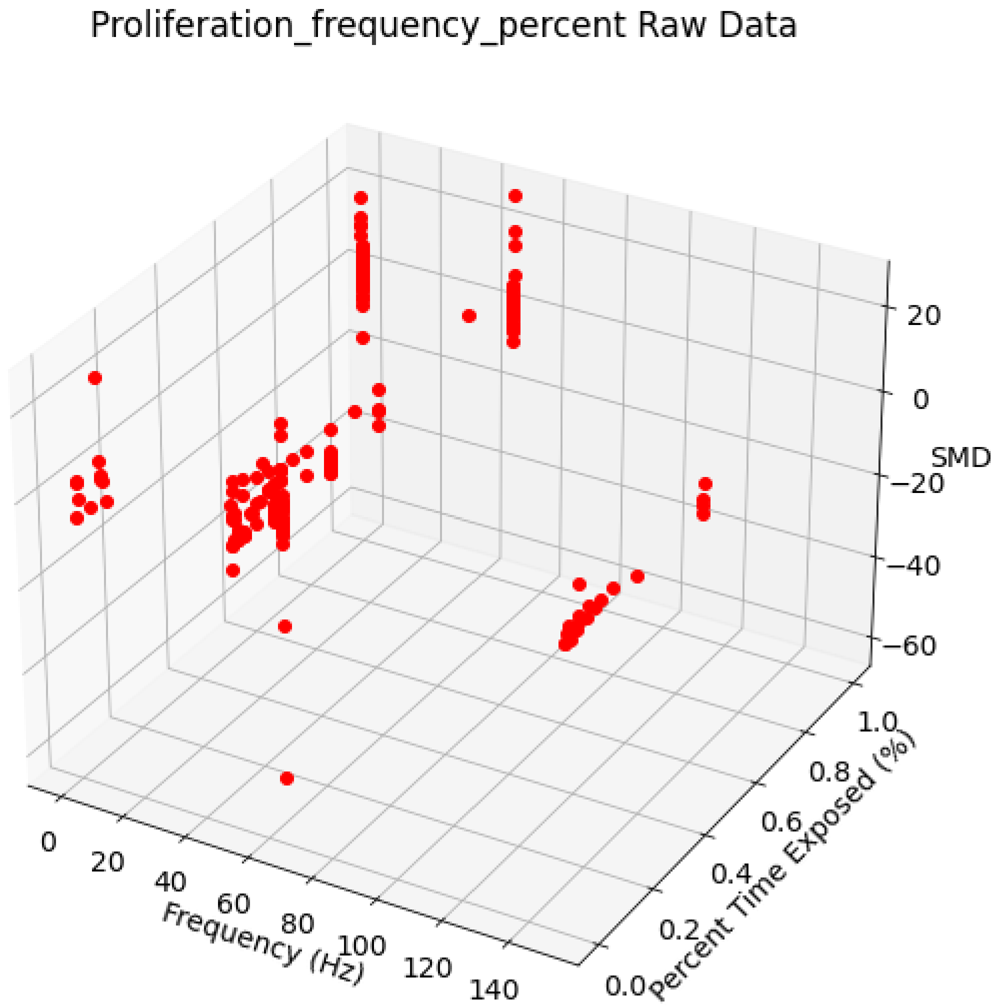
Proliferation_frequency_percent Raw Data
20
0
SMD
−20
−40
−60
1.0
0.8
0.6
0.4
0.2
0.0
Percent Time Exposed (%)
0
20
40
60
80
100
120
140
Frequency (Hz)

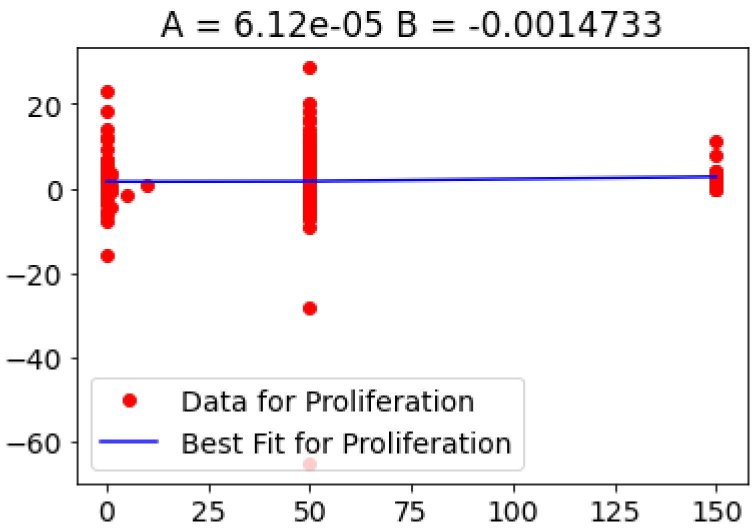
A = 6.12e-05 B = -0.0014733
20
0
−20
−40
−60
0
25
50
75
100
125
150
Data for Proliferation
Best Fit for Proliferation

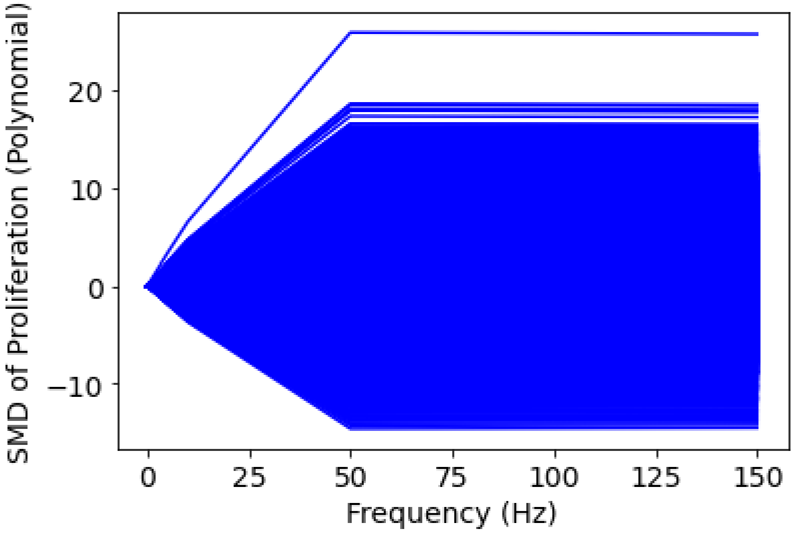
SMD of Proliferation (Polynomial)
Frequency (Hz)
20
10
0
−10
0
25
50
75
100
125
150

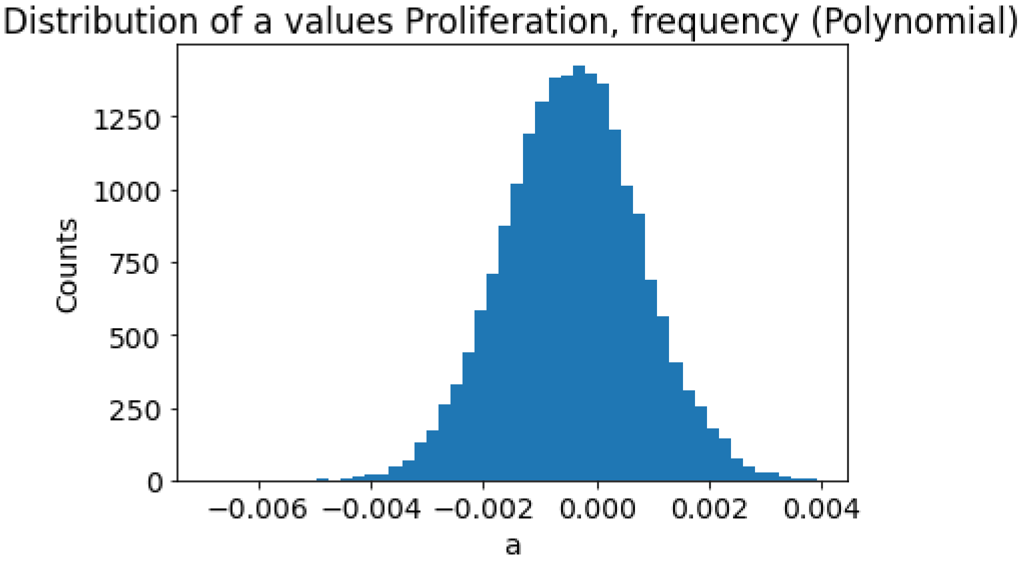
Distribution of a values Proliferation, frequency (Polynomial)
Counts
a
1250
1000
750
500
250
0
−0.006
−0.004
−0.002
0.000
0.002
0.004

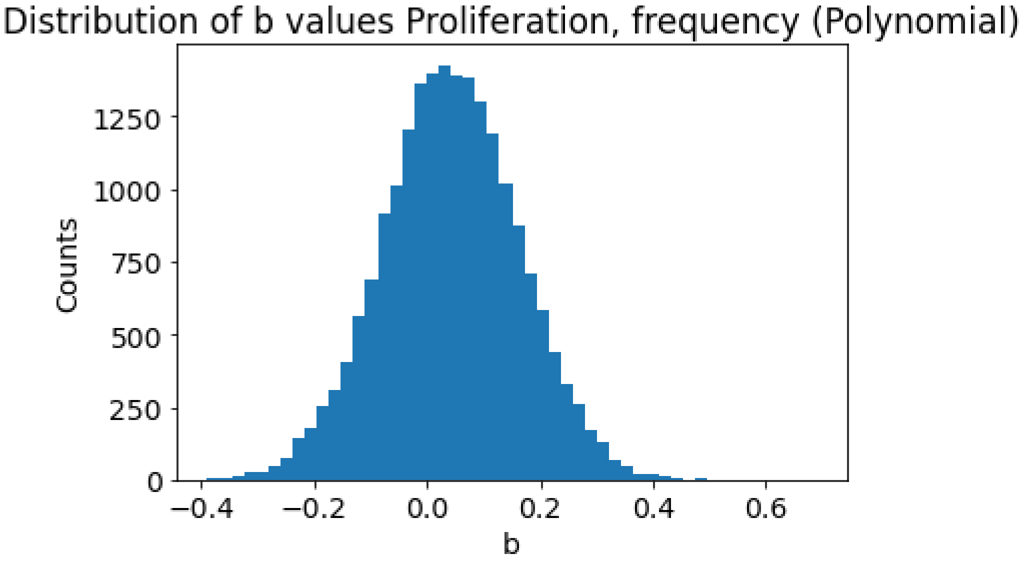
Distribution of b values Proliferation, frequency (Polynomial)
Counts
b
1250
1000
750
500
250
0
−0.4
−0.2
0.0
0.2
0.4
0.6

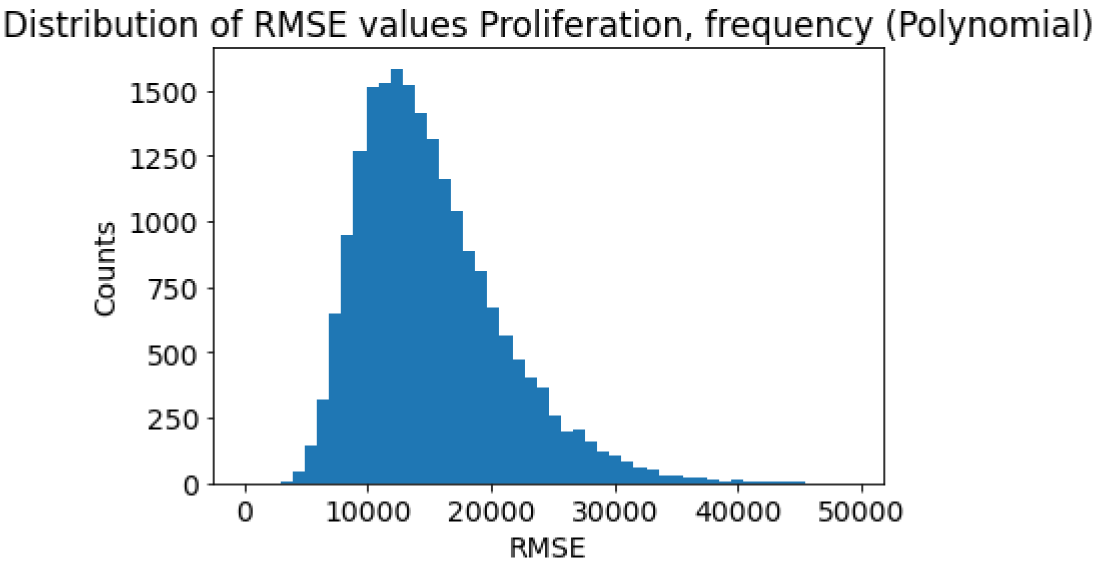
Distribution of RMSE values Proliferation, frequency (Polynomial)
Counts
RMSE
0
250
500
750
1000
1250
1500
0
10000
20000
30000
40000
50000

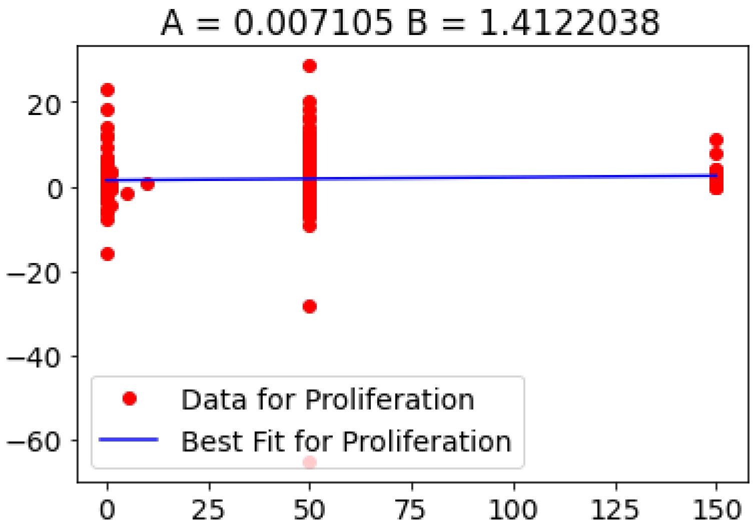
A = 0.007105 B = 1.4122038
Data for Proliferation
Best Fit for Proliferation
20
0
−20
−40
−60
0
25
50
75
100
125
150

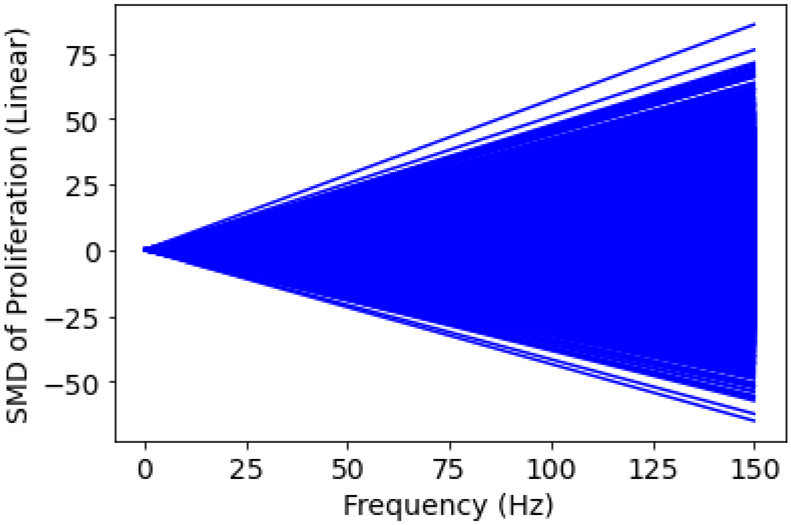
SMD of Proliferation (Linear)
Frequency (Hz)
75
50
25
0
−25
−50
0
25
50
75
100
125
150

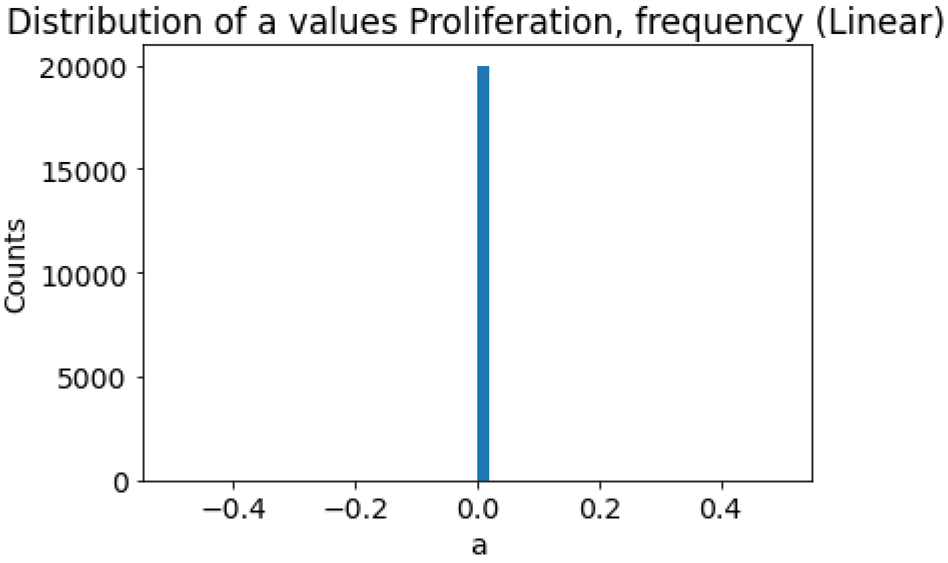
Distribution of a values Proliferation, frequency (Linear)
Counts
20000
15000
10000
5000
0
−0.4
−0.2
0.0
0.2
0.4
a

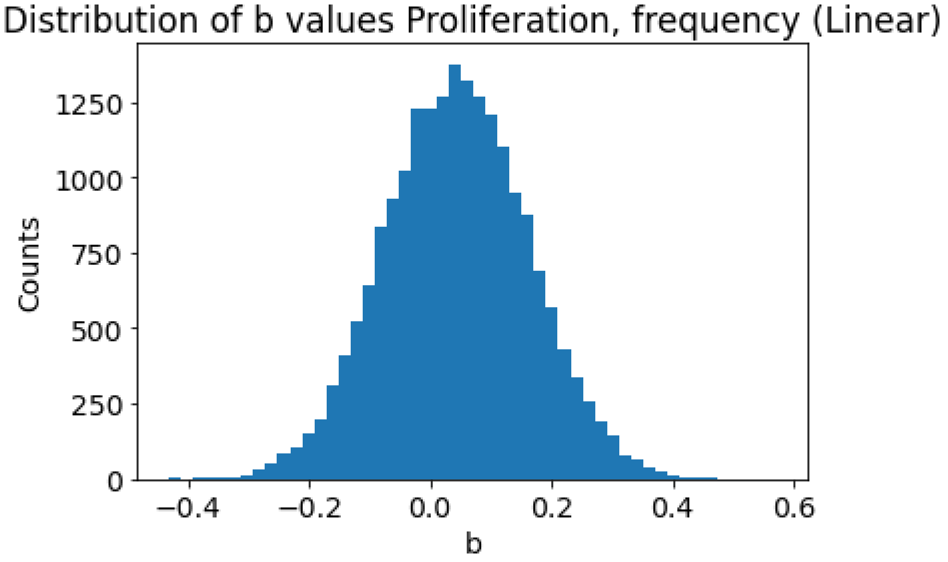
Distribution of b values Proliferation, frequency (Linear)
Counts
1250
1000
750
500
250
0
−0.4
−0.2
0.0
0.2
0.4
0.6
b

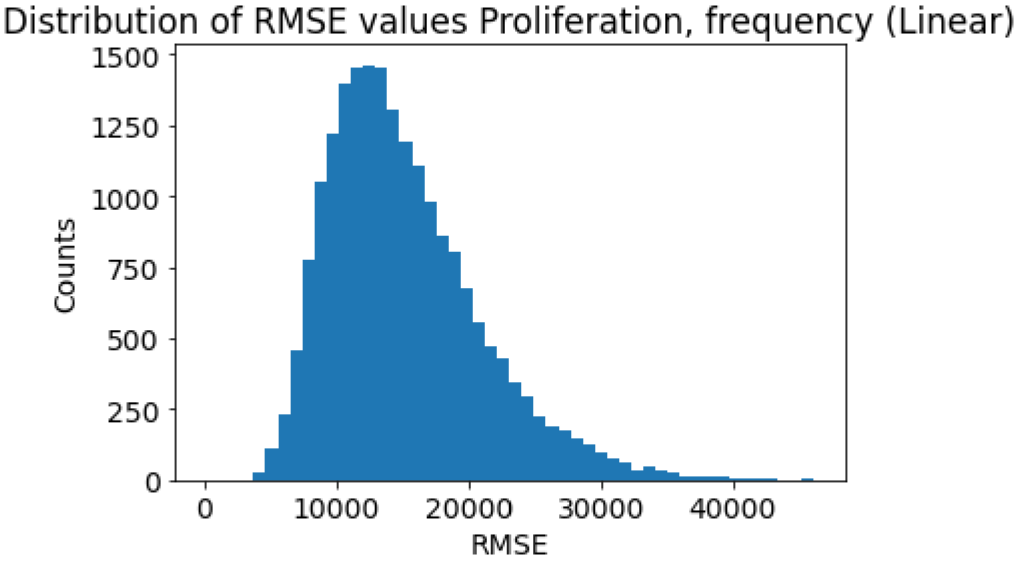
Distribution of RMSE values Proliferation, frequency (Linear)
Counts
1500
1250
1000
750
500
250
0
0
10000
20000
30000
40000
RMSE

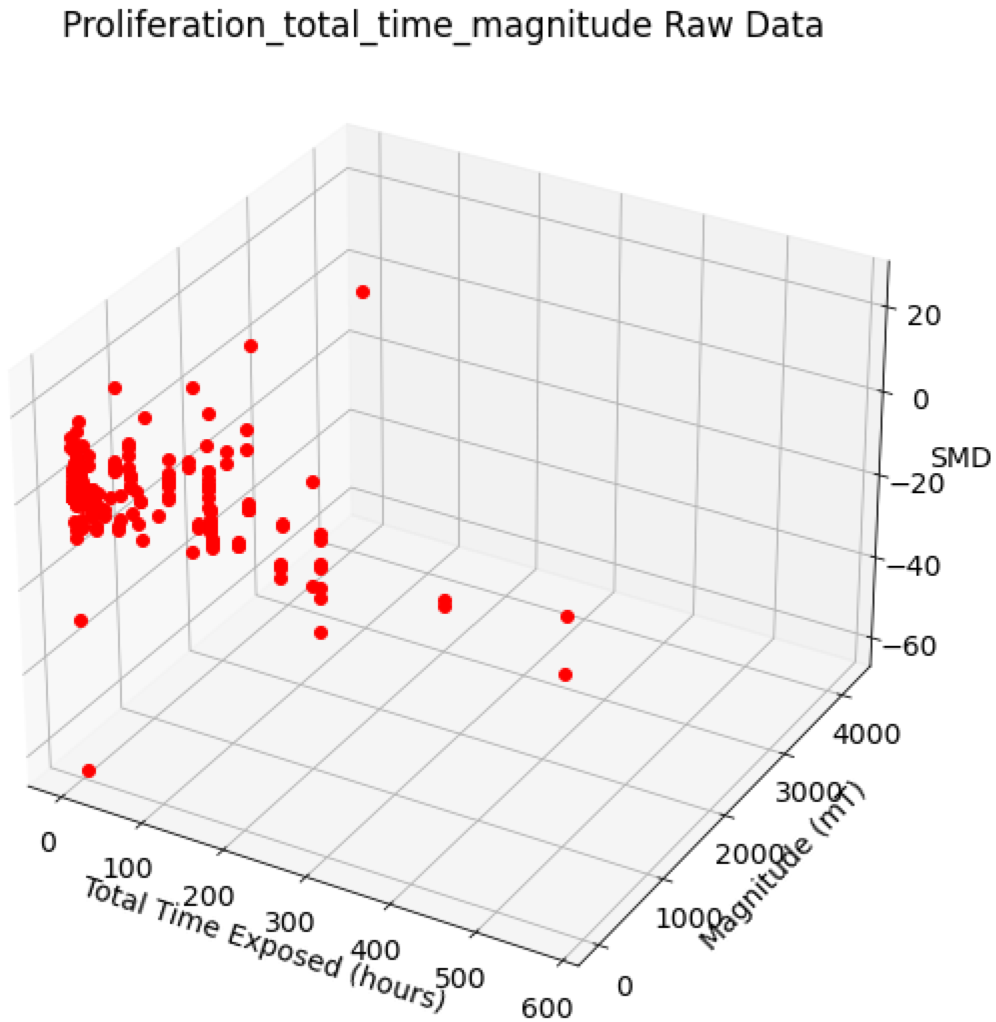
Proliferation_total_time_magnitude Raw Data
SMD
20
0
−20
−40
−60
Total Time Exposed (hours)
0
100
200
300
400
500
600
Magnitude (mT)
0
1000
2000
3000
4000

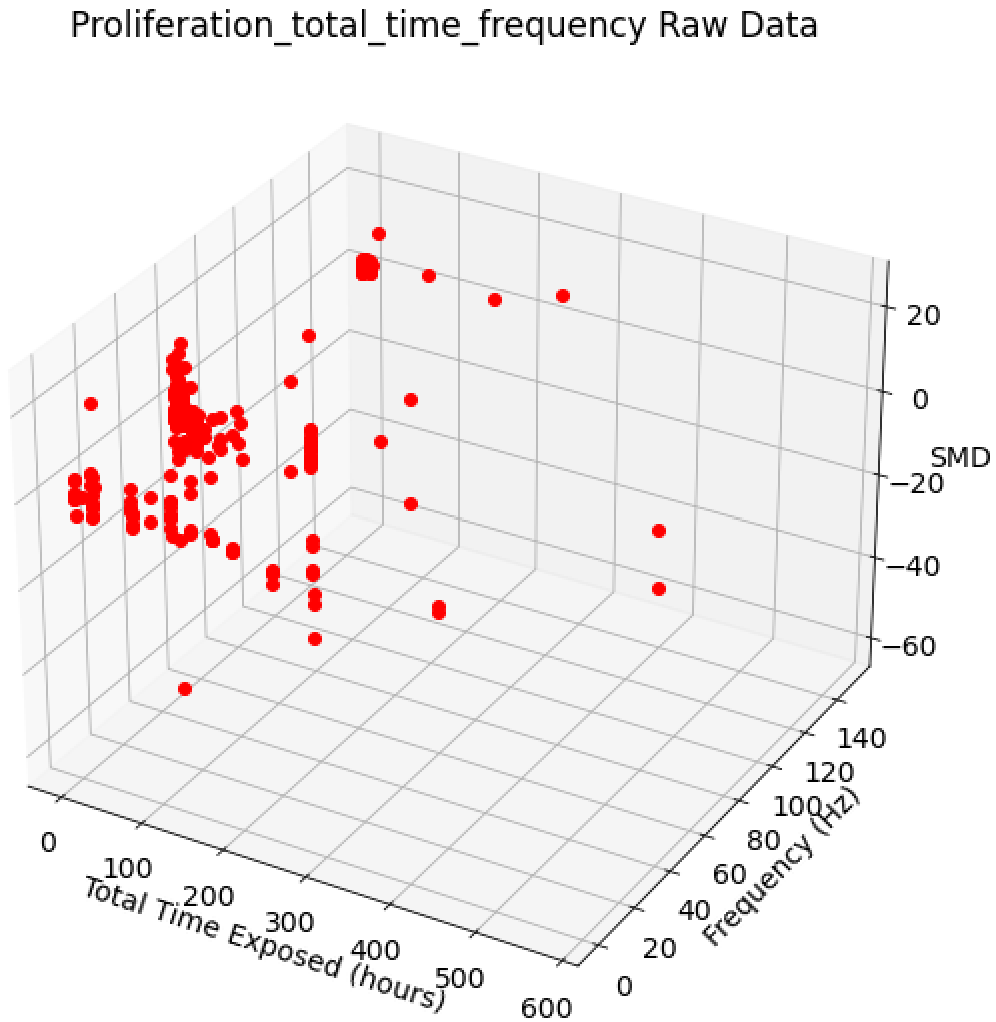
Proliferation_total_time_frequency Raw Data
SMD
20
0
−20
−40
−60
Total Time Exposed (hours)
0
100
200
300
400
500
600
Frequency (Hz)
0
20
40
60
80
100
120
140

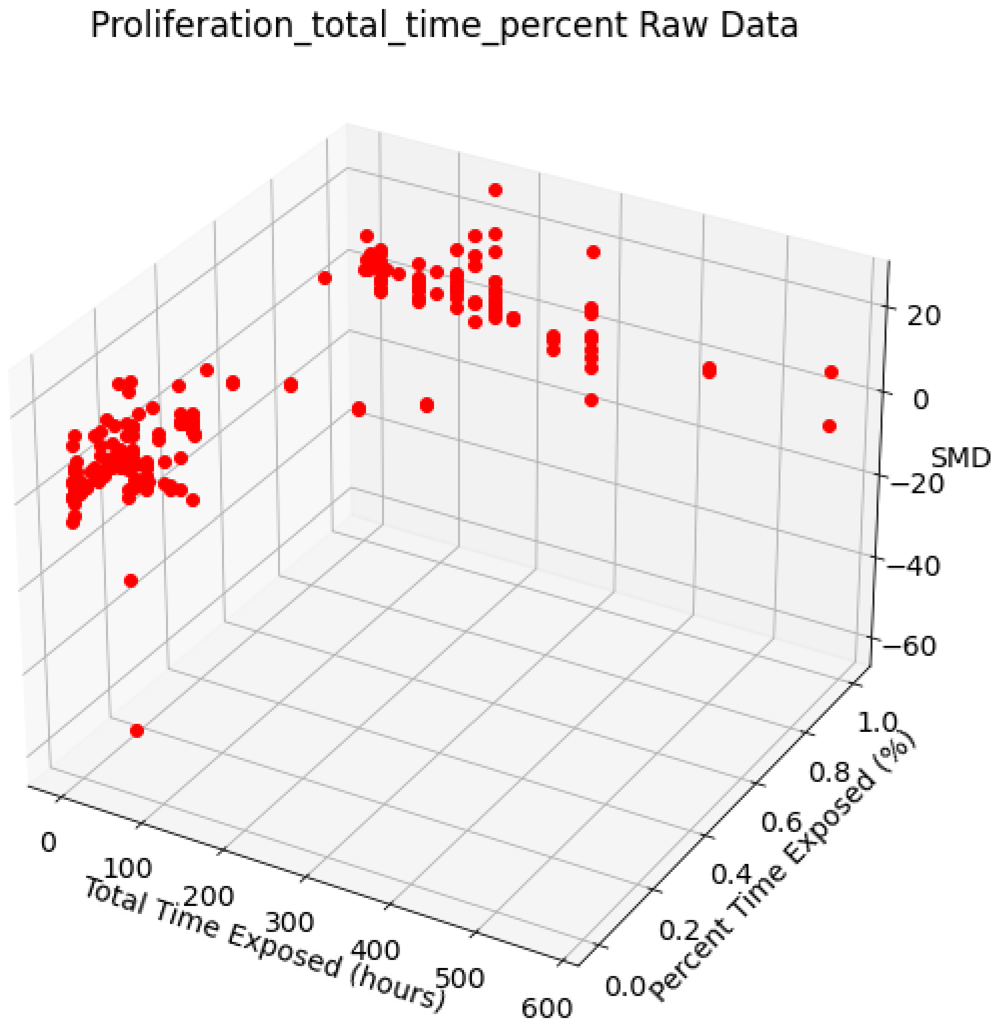
Proliferation_total_time_percent Raw Data
Total Time Exposed (hours)
Percent Time Exposed (%)
SMD
0
100
200
300
400
500
600
0.0
0.2
0.4
0.6
0.8
1.0
20
0
−20
−40
−60

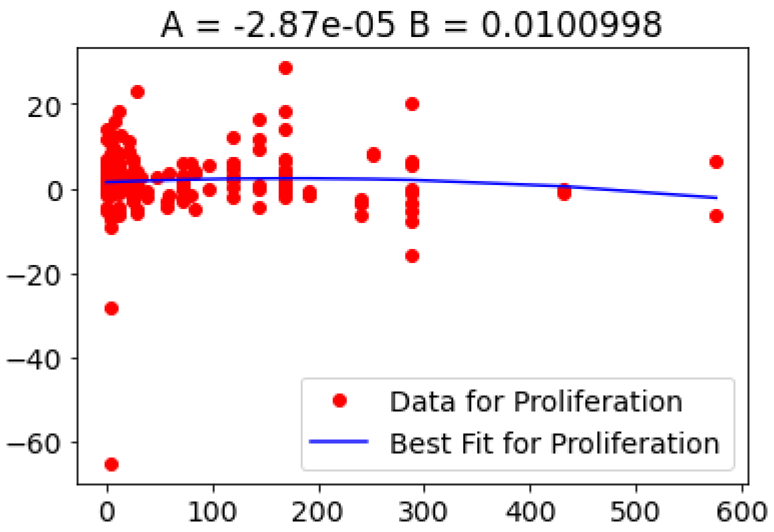
A = -2.87e-05 B = 0.0100998
Data for Proliferation
Best Fit for Proliferation
20
0
−20
−40
−60
0
100
200
300
400
500
600

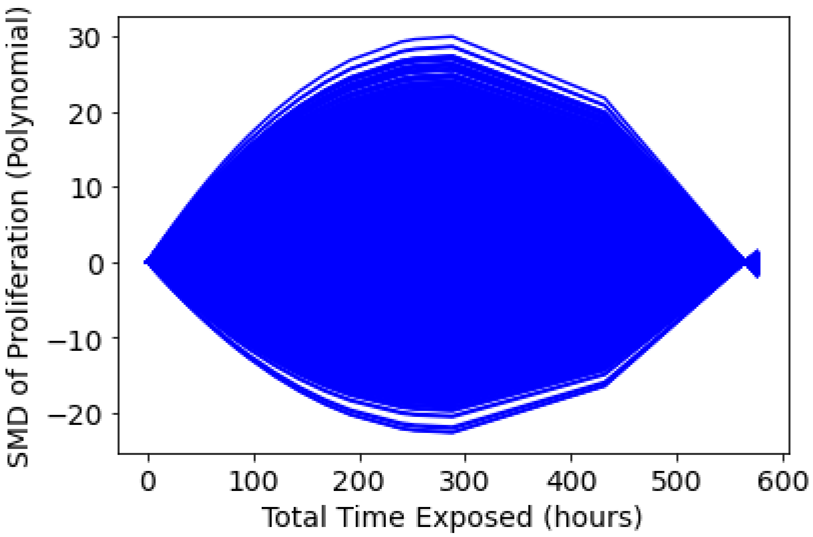
SMD of Proliferation (Polynomial)
Total Time Exposed (hours)

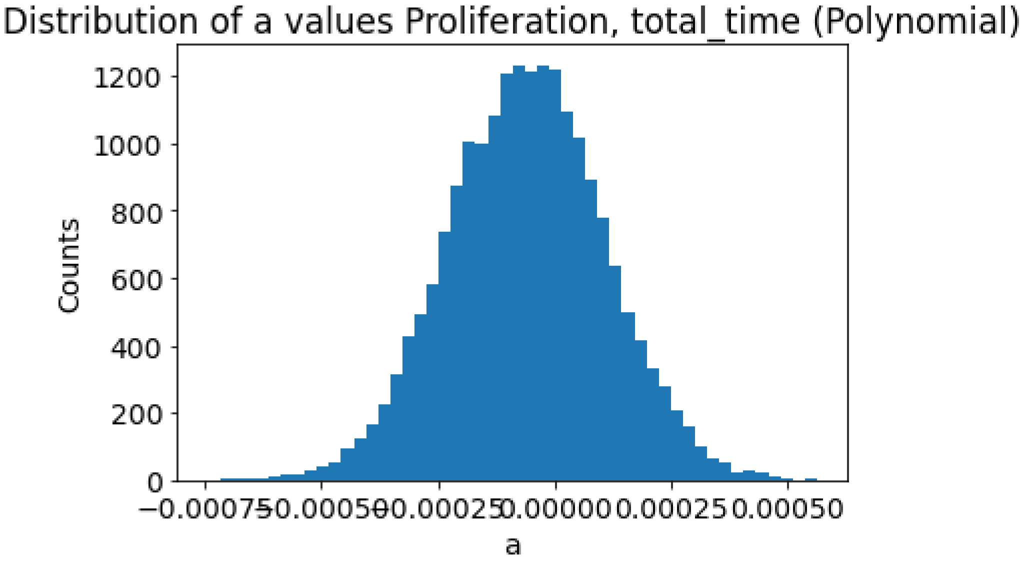
Distribution of a values Proliferation, total_time (Polynomial)
Counts
a

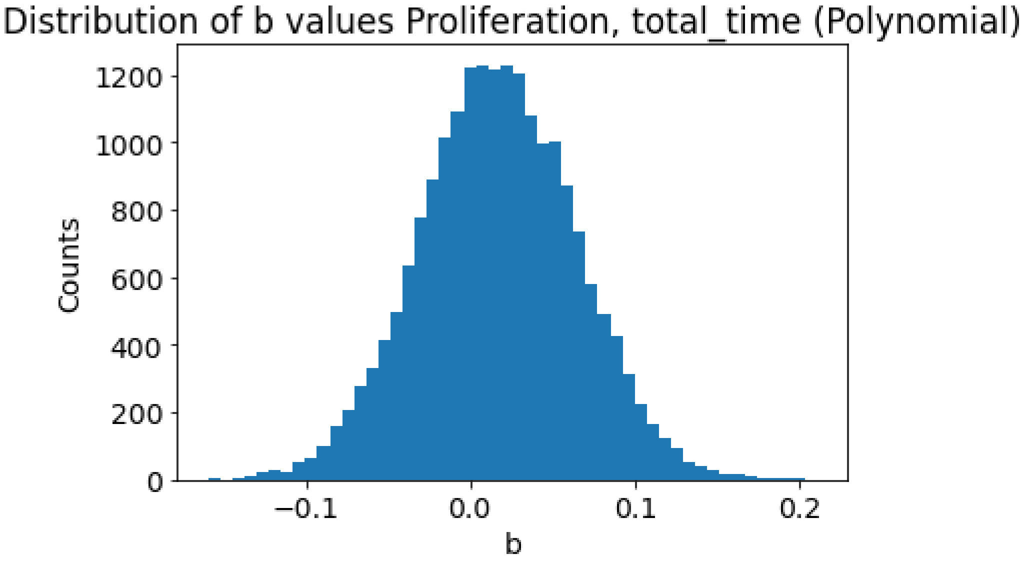
Distribution of b values Proliferation, total_time (Polynomial)
Counts
b

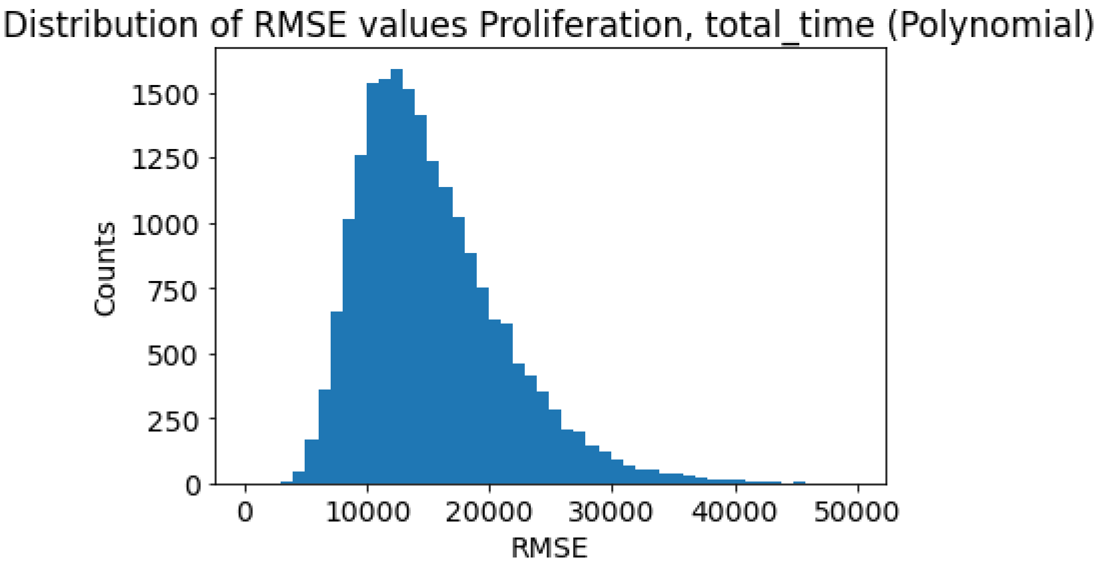
Distribution of RMSE values Proliferation, total_time (Polynomial)
Counts
RMSE
0
250
500
750
1000
1250
1500
0
10000
20000
30000
40000
50000

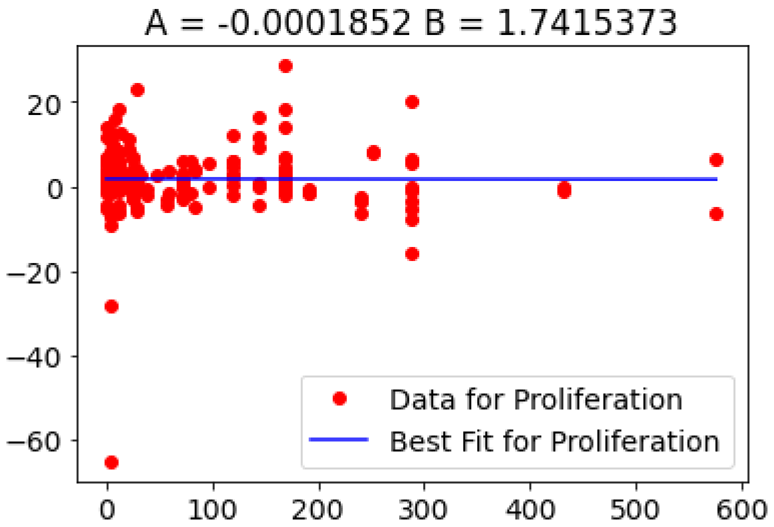
A = -0.0001852 B = 1.7415373
Data for Proliferation
Best Fit for Proliferation
20
0
−20
−40
−60
0
100
200
300
400
500
600

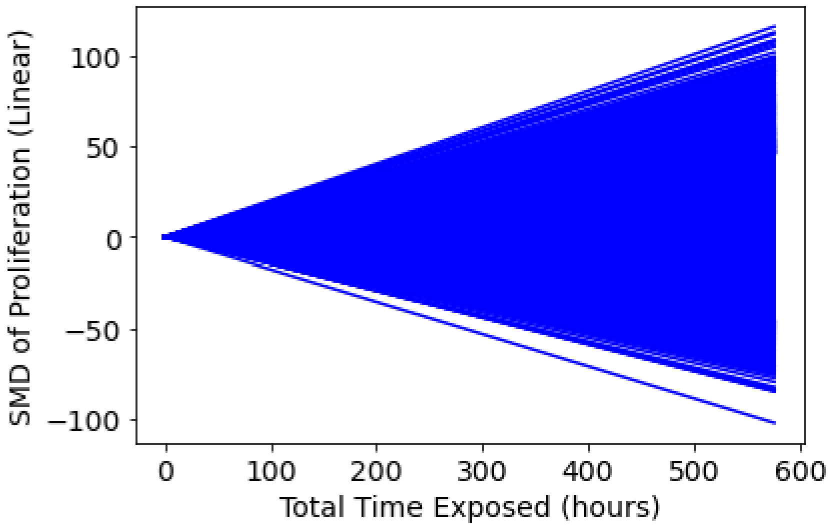
SMD of Proliferation (Linear)
Total Time Exposed (hours)
100
50
0
−50
−100
0
100
200
300
400
500
600

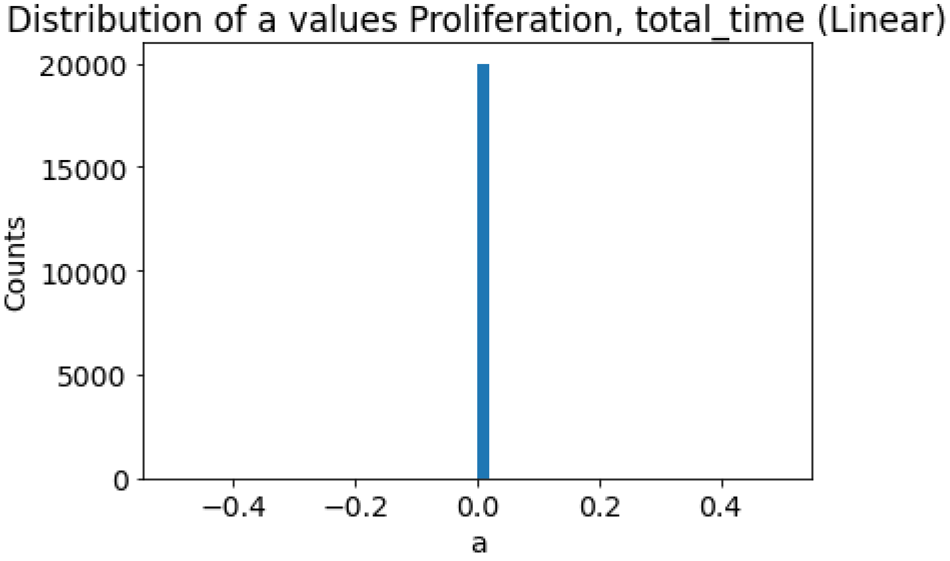
Distribution of a values Proliferation, total_time (Linear)
Counts
a
20000
15000
10000
5000
0
−0.4
−0.2
0.0
0.2
0.4

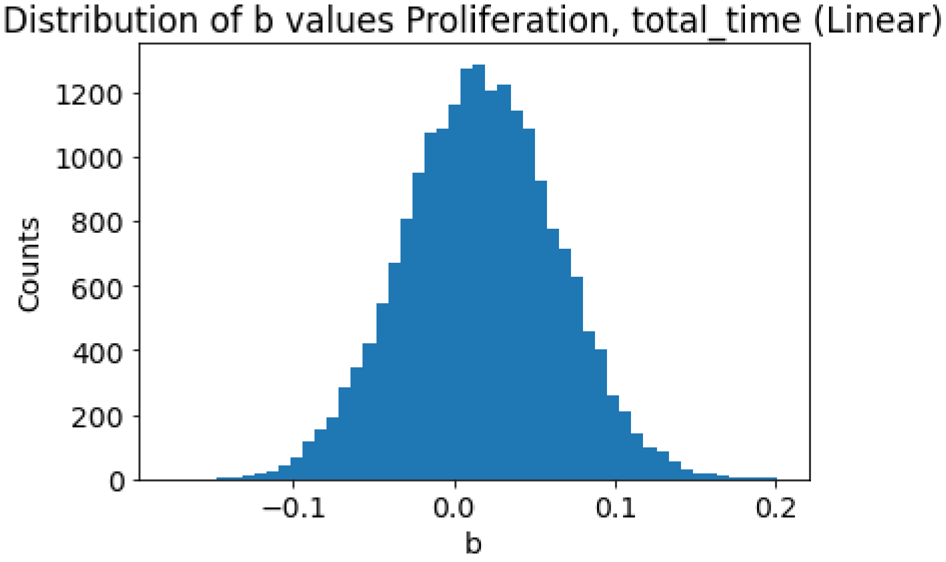
Distribution of b values Proliferation, total_time (Linear)
Counts
b
1200
1000
800
600
400
200
0
−0.1
0.0
0.1
0.2

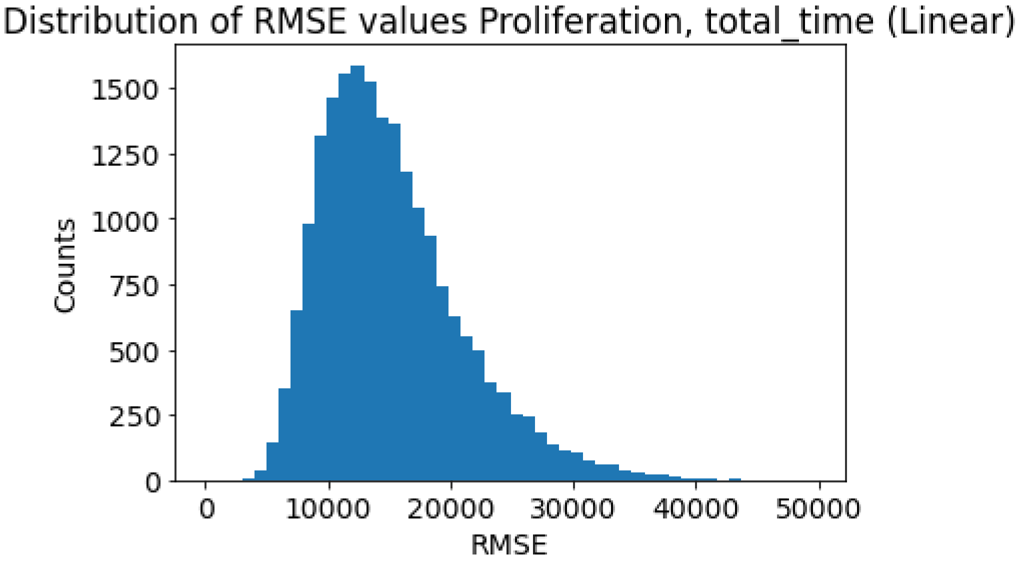
Distribution of RMSE values Proliferation, total_time (Linear)
Counts
RMSE
1500
1250
1000
750
500
250
0
0
10000
20000
30000
40000
50000

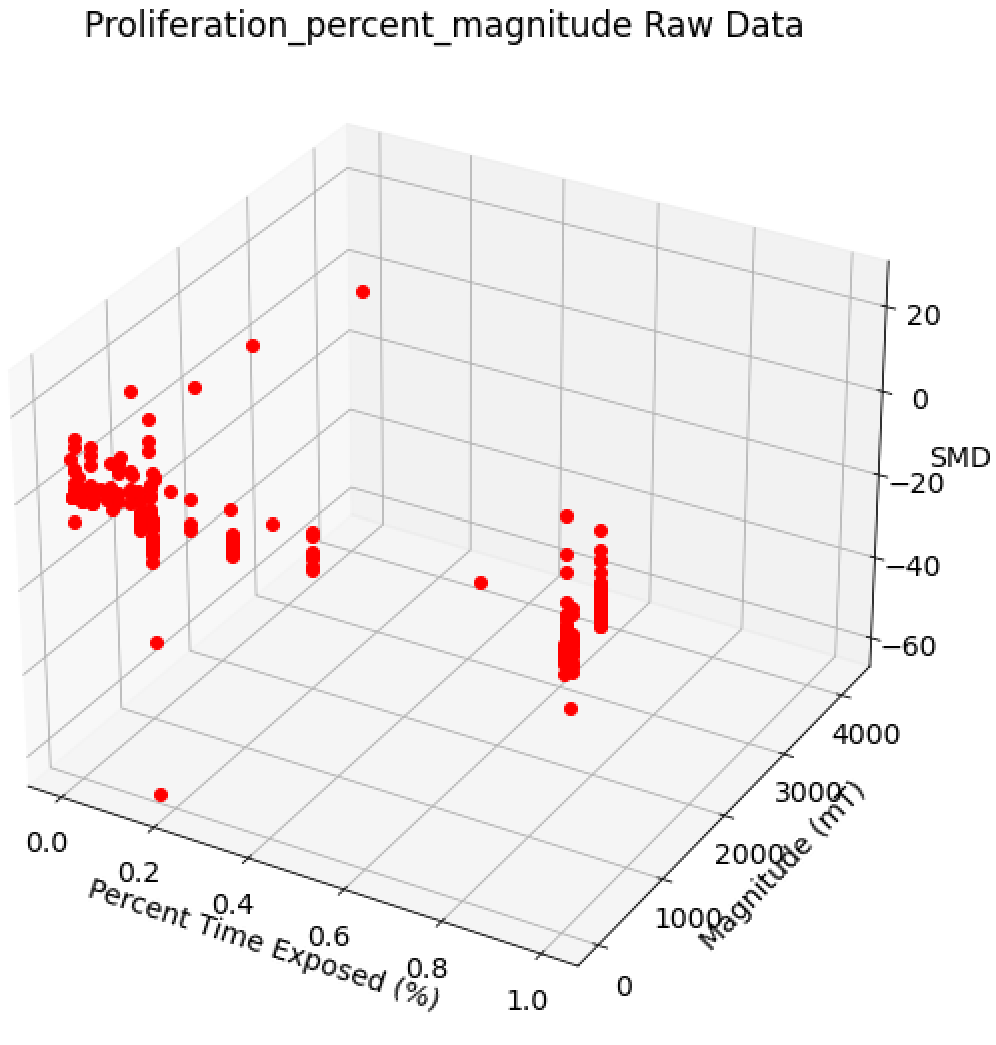
Proliferation_percent_magnitude Raw Data
SMD
20
0
−20
−40
−60
0.0
0.2
0.4
0.6
0.8
1.0
Percent Time Exposed (%)
0
1000
2000
3000
4000
Magnitude (mT)

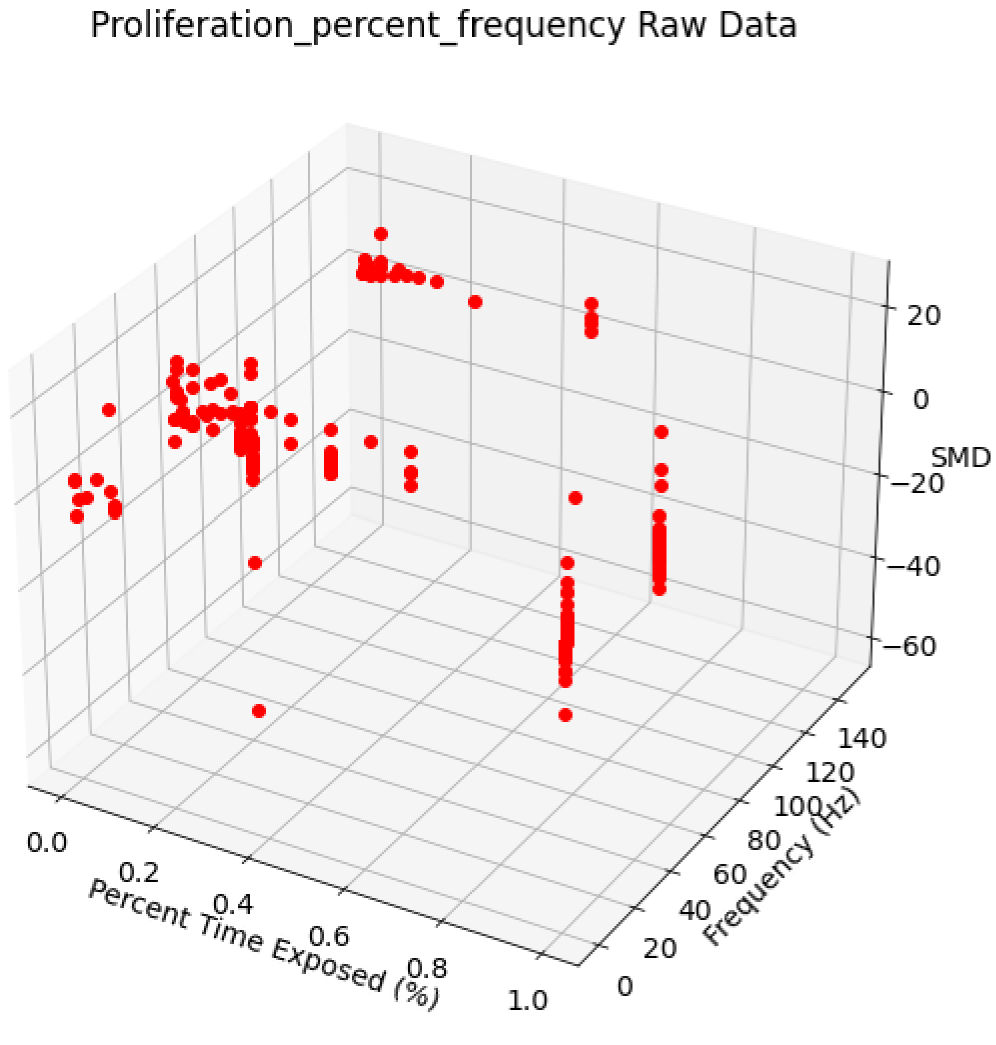
Proliferation_percent_frequency Raw Data
SMD
20
0
−20
−40
−60
0.0
0.2
0.4
0.6
0.8
1.0
Percent Time Exposed (%)
0
20
40
60
80
100
120
140
Frequency (Hz)

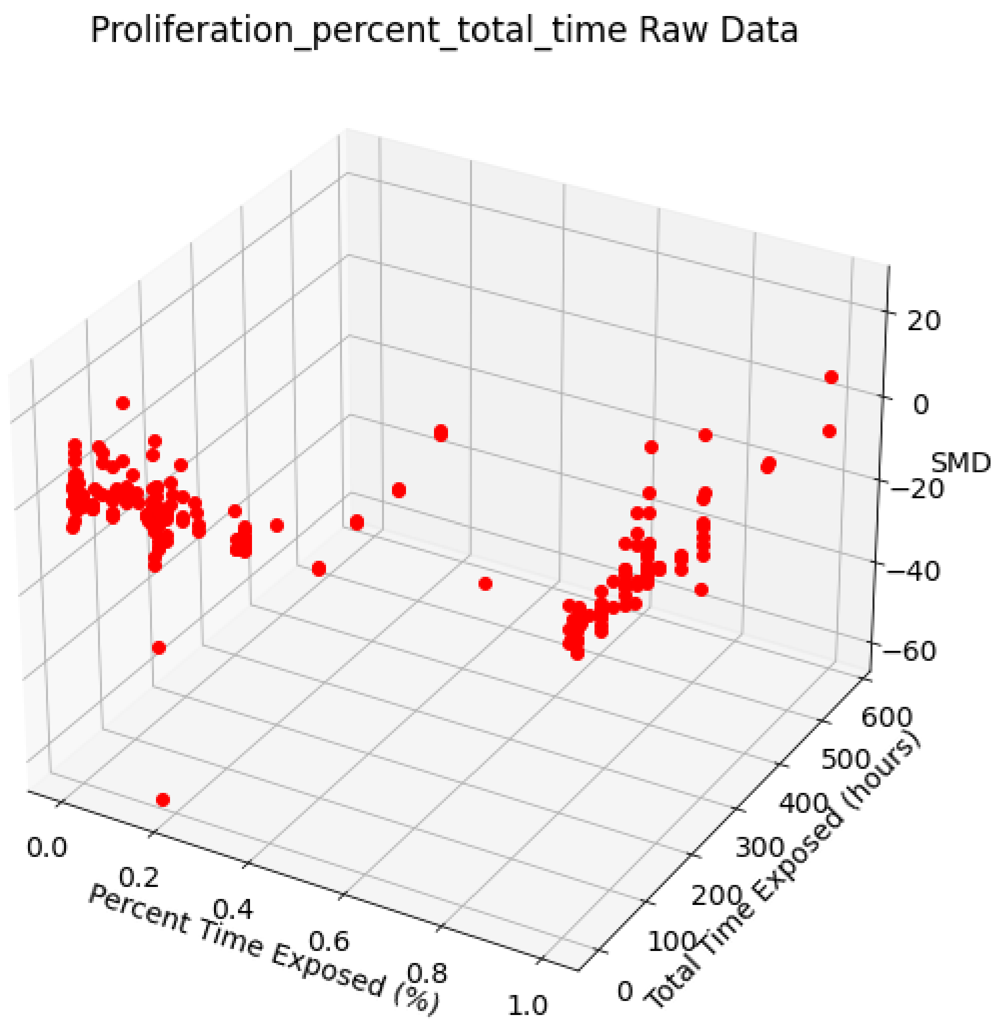
Proliferation_percent_total_time Raw Data
SMD
20
0
−20
−40
−60
Percent Time Exposed (%)
0.0
0.2
0.4
0.6
0.8
1.0
Total Time Exposed (hours)
0
100
200
300
400
500
600

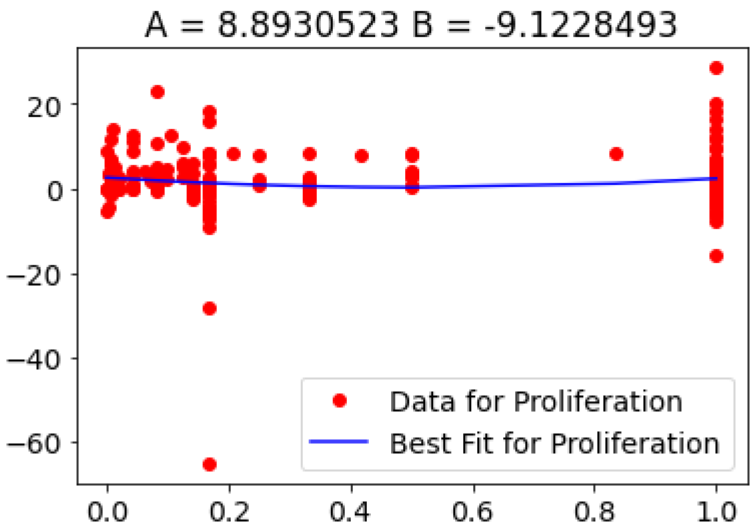
A = 8.8930523 B = -9.1228493
Data for Proliferation
Best Fit for Proliferation
20
0
−20
−40
−60
0.0
0.2
0.4
0.6
0.8
1.0

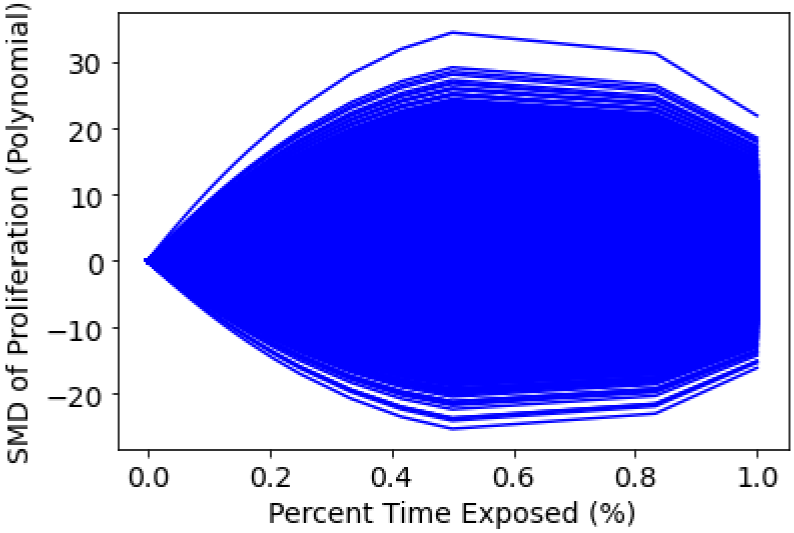


Distribution of a values Proliferation, percent (Polynomial)

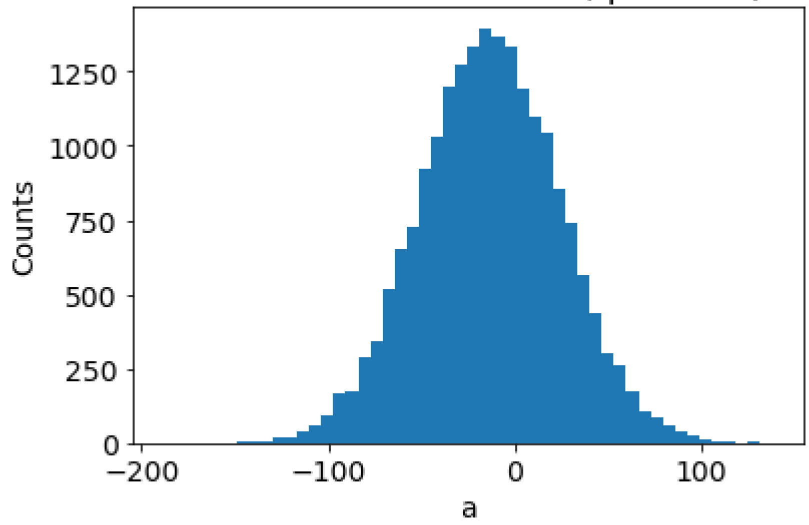


Distribution of b values Proliferation, percent (Polynomial)

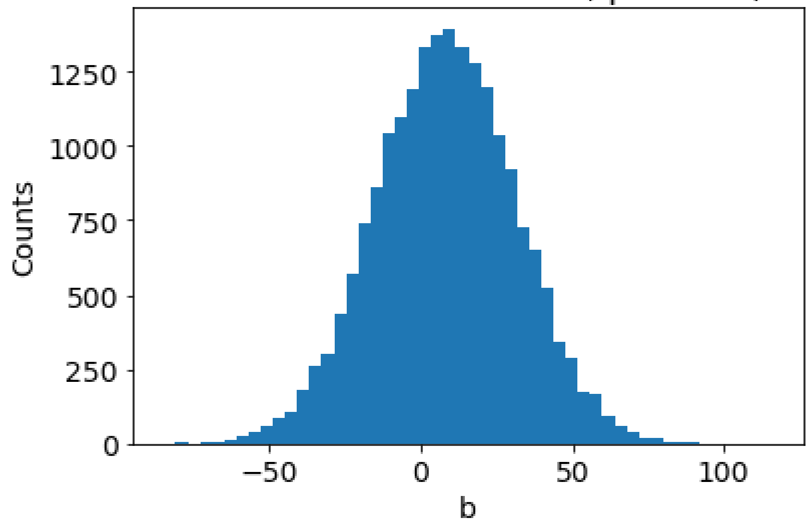

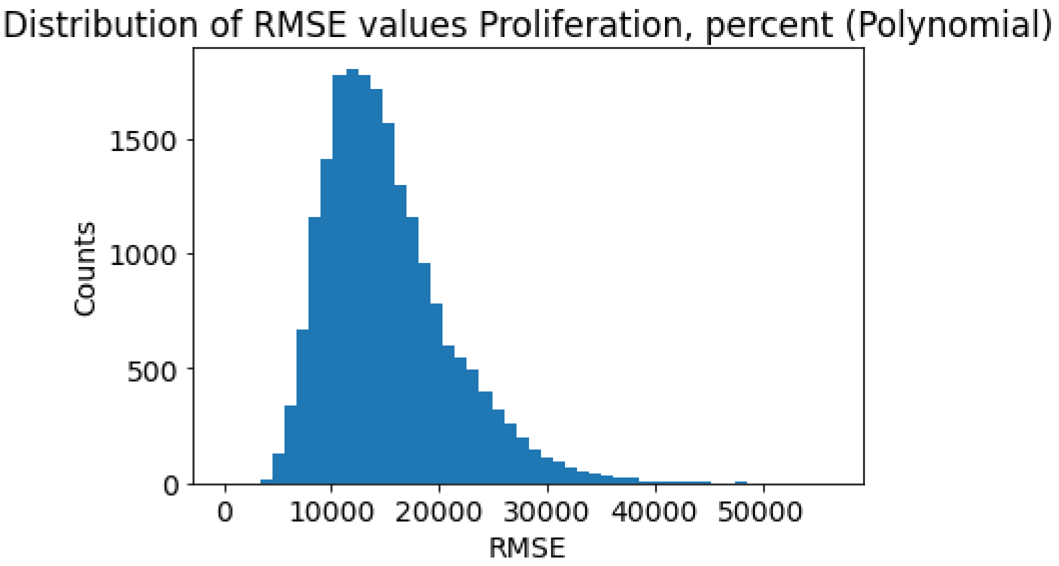
Distribution of RMSE values Proliferation, percent (Polynomial)
Counts
1500
1000
500
0
0
10000
20000
30000
40000
50000
RMSE

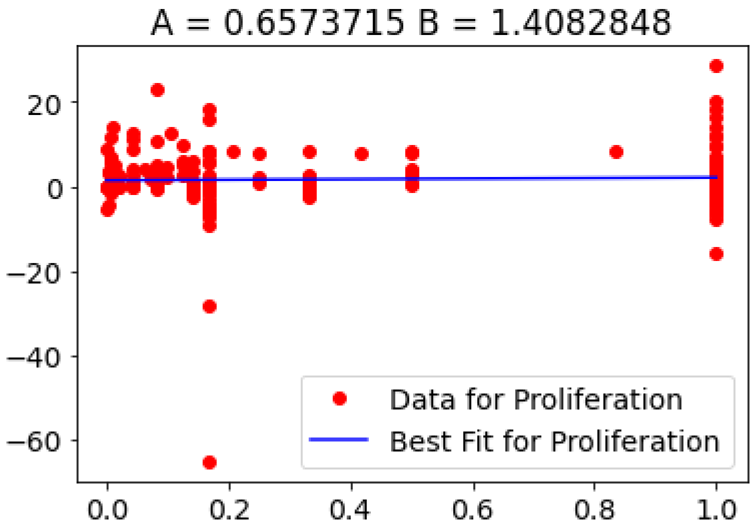
A = 0.6573715 B = 1.4082848
20
0
−20
−40
−60
0.0
0.2
0.4
0.6
0.8
1.0
Data for Proliferation
Best Fit for Proliferation

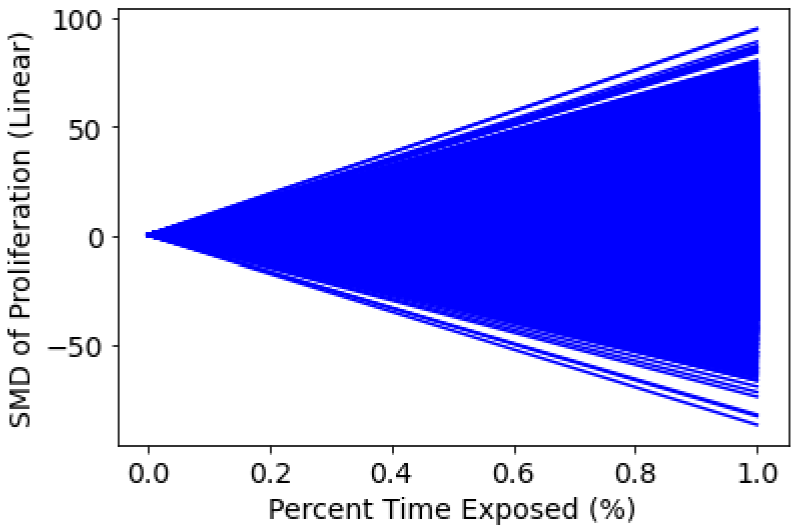
SMD of Proliferation (Linear)
100
50
0
−50
0.0
0.2
0.4
0.6
0.8
1.0
Percent Time Exposed (%)

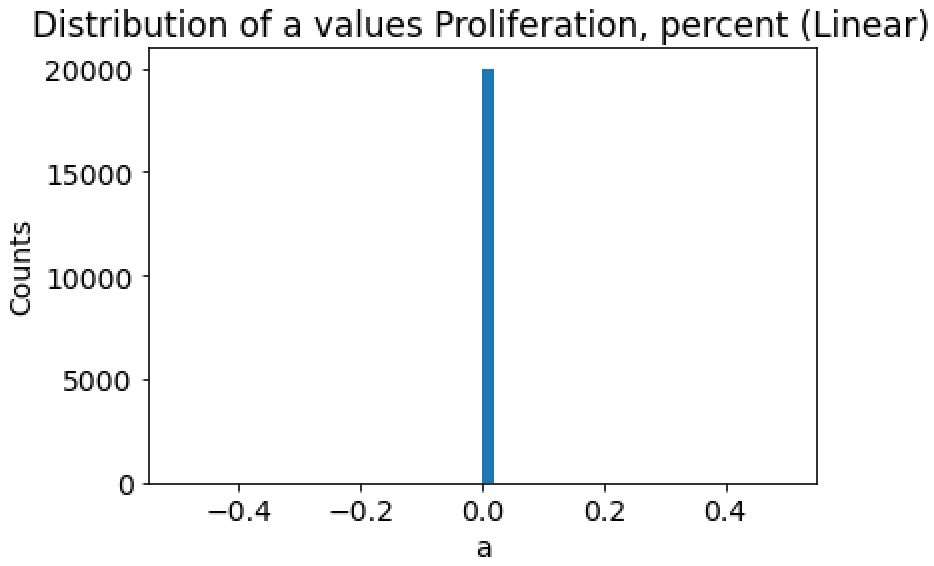


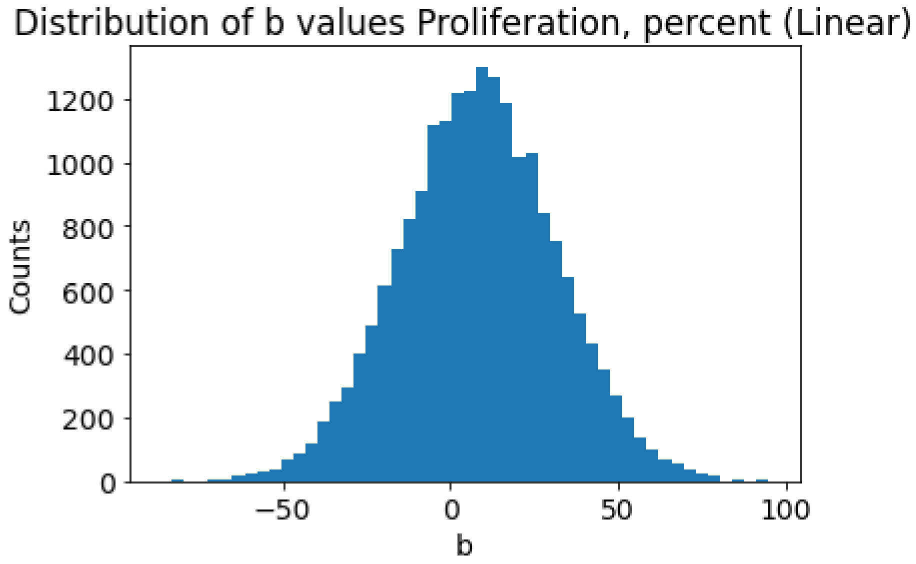


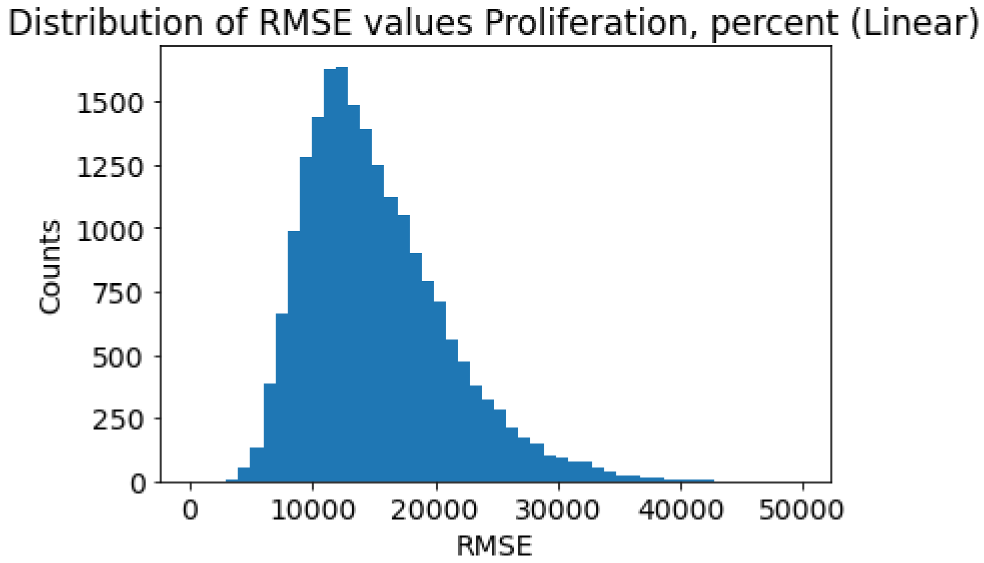


# Call functions appropriately (p-value calculation)

# Iterate through all of the a and b values from the simulations to generate an average value and probability of being 0

In [8]:

```
# filepath
filepath_a= 'Final_data/A_matrix/'
filepath_b= 'Final_data/B_matrix/'

#make starting list for collecting all significant data
significant = [['Analysis', "Choice", 'A/B', 'Fit', "Probability", "Value"]]

#A_matrix properties
for filename in os.listdir(filepath_a):
    if 'csv' in filename:

        #extraction of names
        f = os.path.join(filepath_a, filename)

        #extract type of analysis--A
        x = f.find("_matrix")
        if 'Linear' in f:
            y = f.find('_Linear')
            fit = "Linear"
        else:
            y = f.find('_Polynomial')
            fit = 'Polynomial'

        title = f[x + 8 : y]

        a= pd.read_csv(f, index_col = None)

        f_b = filepath_b + filename

        b= pd.read_csv(f_b, index_col = None)

        #actually run the function
        result = calc_p_value(a, b, title, fit)

        #more labeling for the final spreadsheet
        if 'magnitude' in f:
            ch = 'magnitude'
        elif 'frequency' in f:
            ch = 'frequency'
        elif 'percent' in f:
            ch = 'percent'
        else:
            ch = 'total time'

        # if the results are significant, add it to the final spreadsheet
        if result[0] < 0.05:
            significant.append([title] + [ch] + ["A"] + [fit] + [result[0]] + [result[2
        if result[1] < 0.05:
            significant.append([title] + [ch] + ["B"]+ [fit] + [result[1]] + [result[3]

#write the accumulated file
with open("Significant_values.csv", "w", newline='') as f:
    writer = csv.writer(f,delimiter=',')
    writer.writerows(significant)
```

```
Astrocyte_frequency
The average value for the a parameter is 0.0000.
The average value for the b parameter is -48.6247.
The p-value for the a parameter is 1.0000.
The p-value for the b parameter is 0.4951.
Astrocyte_frequency
The average value for the a parameter is 0.5303.
The average value for the b parameter is -13.2845.
The p-value for the a parameter is 0.4978.
The p-value for the b parameter is 0.4978.
Astrocyte_magnitude
The average value for the a parameter is 0.0000.
The average value for the b parameter is -0.0009.
The p-value for the a parameter is 1.0000.
The p-value for the b parameter is 0.4966.
Astrocyte_magnitude
The average value for the a parameter is 0.0000.
The average value for the b parameter is -0.0019.
The p-value for the a parameter is 0.4959.
The p-value for the b parameter is 0.4957.
Astrocyte_percent
The average value for the a parameter is 0.0000.
The average value for the b parameter is -12.7800.
The p-value for the a parameter is 1.0000.
The p-value for the b parameter is 0.4954.
Astrocyte_percent
The average value for the a parameter is 20.9884.
The average value for the b parameter is -13.9488.
The p-value for the a parameter is 0.4937.
The p-value for the b parameter is 0.4926.
Astrocyte_total_time
The average value for the a parameter is 0.0000.
The average value for the b parameter is -0.0105.
The p-value for the a parameter is 1.0000.
The p-value for the b parameter is 0.4967.
Astrocyte_total_time
The average value for the a parameter is -0.0000.
The average value for the b parameter is 0.0032.
The p-value for the a parameter is 0.5009.
The p-value for the b parameter is 0.5009.
Differentiation_frequency
The average value for the a parameter is 0.0000.
The average value for the b parameter is 0.0880.
The p-value for the a parameter is 1.0000.
The p-value for the b parameter is 0.4607.
Differentiation_frequency
The average value for the a parameter is -0.0010.
The average value for the b parameter is 0.0951.
The p-value for the a parameter is 0.4607.
The p-value for the b parameter is 0.4607.
Differentiation_magnitude
The average value for the a parameter is 0.0000.
The average value for the b parameter is 0.0021.
The p-value for the a parameter is 1.0000.
The p-value for the b parameter is 0.4545.
Differentiation_magnitude
The average value for the a parameter is -0.0000.
The average value for the b parameter is 0.0024.
The p-value for the a parameter is 0.4576.
The p-value for the b parameter is 0.4586.
Differentiation_total_time
The average value for the a parameter is 0.0000.
The average value for the b parameter is 0.0334.
The p-value for the a parameter is 1.0000.
The p-value for the b parameter is 0.4650.
```

```
Differentiation_total_time
The average value for the a parameter is -0.0001.
The average value for the b parameter is 0.0344.
The p-value for the a parameter is 0.4604.
The p-value for the b parameter is 0.4604.
Growth_frequency
The average value for the a parameter is 0.0000.
The average value for the b parameter is 0.1800.
The p-value for the a parameter is 1.0000.
The p-value for the b parameter is 0.0001.
Growth_frequency
The average value for the a parameter is -0.0018.
The average value for the b parameter is 0.1801.
The p-value for the a parameter is 0.0000.
The p-value for the b parameter is 0.0001.
Growth_magnitude
The average value for the a parameter is 0.0000.
The average value for the b parameter is 2.0190.
The p-value for the a parameter is 1.0000.
The p-value for the b parameter is 0.0001.
Growth_magnitude
The average value for the a parameter is -0.0133.
The average value for the b parameter is 2.0190.
The p-value for the a parameter is 0.0000.
The p-value for the b parameter is 0.0001.
Growth_percent
The average value for the a parameter is 0.0000.
The average value for the b parameter is 37.9929.
The p-value for the a parameter is 1.0000.
The p-value for the b parameter is 0.0001.
Growth_percent
The average value for the a parameter is -64.6605.
The average value for the b parameter is 38.0263.
The p-value for the a parameter is 0.0000.
The p-value for the b parameter is 0.0001.
Growth_total_time
The average value for the a parameter is 0.0000.
The average value for the b parameter is 0.1493.
The p-value for the a parameter is 1.0000.
The p-value for the b parameter is 0.0001.
Growth_total_time
The average value for the a parameter is -0.0011.
The average value for the b parameter is 0.1496.
The p-value for the a parameter is 0.0000.
The p-value for the b parameter is 0.0001.
Neuron_frequency
The average value for the a parameter is 0.0000.
The average value for the b parameter is 0.1385.
The p-value for the a parameter is 1.0000.
The p-value for the b parameter is 0.0001.
Neuron_frequency
The average value for the a parameter is -0.0014.
The average value for the b parameter is 0.1386.
The p-value for the a parameter is 0.0000.
The p-value for the b parameter is 0.0001.
Neuron_magnitude
The average value for the a parameter is 0.0000.
The average value for the b parameter is 0.0034.
The p-value for the a parameter is 1.0000.
The p-value for the b parameter is 0.0001.
Neuron_magnitude
The average value for the a parameter is -0.0000.
The average value for the b parameter is 0.0035.
The p-value for the a parameter is 0.0000.
The p-value for the b parameter is 0.0001.
```

```
Neuron_total_time
The average value for the a parameter is 0.0000.
The average value for the b parameter is 0.0538.
The p-value for the a parameter is 1.0000.
The p-value for the b parameter is 0.0001.
Neuron_total_time
The average value for the a parameter is -0.0002.
The average value for the b parameter is 0.0538.
The p-value for the a parameter is 0.0000.
The p-value for the b parameter is 0.0001.
Oligodendrocyte_frequency
The average value for the a parameter is 0.0000.
The average value for the b parameter is -2.7262.
The p-value for the a parameter is 1.0000.
The p-value for the b parameter is 0.0000.
Oligodendrocyte_frequency
The average value for the a parameter is -0.0016.
The average value for the b parameter is 0.0596.
The p-value for the a parameter is 0.1356.
The p-value for the b parameter is 0.0001.
Oligodendrocyte_magnitude
The average value for the a parameter is 0.0000.
The average value for the b parameter is 0.0137.
The p-value for the a parameter is 1.0000.
The p-value for the b parameter is 0.2864.
Oligodendrocyte_magnitude
The average value for the a parameter is -0.0001.
The average value for the b parameter is 0.0134.
The p-value for the a parameter is 0.2929.
The p-value for the b parameter is 0.2930.
Oligodendrocyte_total_time
The average value for the a parameter is 0.0000.
The average value for the b parameter is 0.0208.
The p-value for the a parameter is 1.0000.
The p-value for the b parameter is 0.2858.
Oligodendrocyte_total_time
The average value for the a parameter is -0.0001.
The average value for the b parameter is 0.0207.
The p-value for the a parameter is 0.2890.
The p-value for the b parameter is 0.2891.
Proliferation_frequency
The average value for the a parameter is 0.0000.
The average value for the b parameter is 0.0442.
The p-value for the a parameter is 1.0000.
The p-value for the b parameter is 0.3582.
Proliferation_frequency
The average value for the a parameter is -0.0004.
The average value for the b parameter is 0.0422.
The p-value for the a parameter is 0.3639.
The p-value for the b parameter is 0.3640.
Proliferation_magnitude
The average value for the a parameter is 0.0000.
The average value for the b parameter is 0.0033.
The p-value for the a parameter is 1.0000.
The p-value for the b parameter is 0.3607.
Proliferation_magnitude
The average value for the a parameter is -0.0000.
The average value for the b parameter is 0.0033.
The p-value for the a parameter is 0.3644.
The p-value for the b parameter is 0.3650.
Proliferation_percent
The average value for the a parameter is 0.0000.
The average value for the b parameter is 8.3114.
The p-value for the a parameter is 1.0000.
The p-value for the b parameter is 0.3587.
```

```
Proliferation_percent
The average value for the a parameter is -13.2103.
The average value for the b parameter is 8.1430.
The p-value for the a parameter is 0.3620.
The p-value for the b parameter is 0.3621.
Proliferation_total_time
The average value for the a parameter is 0.0000.
The average value for the b parameter is 0.0167.
The p-value for the a parameter is 1.0000.
The p-value for the b parameter is 0.3631.
Proliferation_total_time
The average value for the a parameter is -0.0001.
The average value for the b parameter is 0.0170.
The p-value for the a parameter is 0.3638.
The p-value for the b parameter is 0.3638.
Viability_frequency
The average value for the a parameter is 0.0000.
The average value for the b parameter is 0.1110.
The p-value for the a parameter is 1.0000.
The p-value for the b parameter is 0.0001.
Viability_frequency
The average value for the a parameter is -0.0011.
The average value for the b parameter is 0.1109.
The p-value for the a parameter is 0.0000.
The p-value for the b parameter is 0.0001.
Viability_magnitude
The average value for the a parameter is 0.0000.
The average value for the b parameter is 0.0079.
The p-value for the a parameter is 1.0000.
The p-value for the b parameter is 0.0001.
Viability_magnitude
The average value for the a parameter is -0.0000.
The average value for the b parameter is 0.0079.
The p-value for the a parameter is 0.0000.
The p-value for the b parameter is 0.0001.
Viability_percent
The average value for the a parameter is 0.0000.
The average value for the b parameter is 21.6271.
The p-value for the a parameter is 1.0000.
The p-value for the b parameter is 0.0001.
Viability_percent
The average value for the a parameter is -35.4247.
The average value for the b parameter is 21.6272.
The p-value for the a parameter is 0.0000.
The p-value for the b parameter is 0.0001.
Viability_total_time
The average value for the a parameter is 0.0000.
The average value for the b parameter is 0.0574.
The p-value for the a parameter is 1.0000.
The p-value for the b parameter is 0.0001.
Viability_total_time
The average value for the a parameter is -0.0003.
The average value for the b parameter is 0.0574.
The p-value for the a parameter is 0.0000.
The p-value for the b parameter is 0.0001.
```

# Raw data extraction

## inputs:

## new_path-- Original data matrices

### outputs:

### SMD-- difference between mean and a value

### SDPool--variance from the mean and a value

### This section helps to reextract data from the original data matrices

In [9]:

```
def raw_data(new_path):
    #set up data for usage in the ODE, unique for every file

    #index
    global index
    index = len(pd.read_csv(new_path))

    #open file, extract data
    #get magnitude or frequency data
    #also extract SMD, SMD_pool
    dat = pd.read_csv(new_path, index_col = None)
    dataMat = pd.DataFrame(dat, columns= ['Title', 'CellCategory', 'SpecificOutcomeVari

    #data_of_interest
    #SMD
    #SD_pool
    global steps
    if 'magnitude' in new_path:
        dataMat = dataMat.sort_values('Magnitudge')
        steps = dataMat.Magnitudge
    elif 'frequency' in new_path:
        dataMat = dataMat.sort_values('Freq')
        steps = dataMat.Freq
    elif 'percent' in new_path:
        dataMat = dataMat.sort_values('pertimeExposed')
        steps = dataMat.pertimeExposed
    else:
        dataMat = dataMat.sort_values('TotalTimeExposed')
        steps = dataMat.TotalTimeExposed

    global SMD
    SMD = dataMat.SMD
    global SDpool
    SDpool = dataMat.Sdpool
```

# ExpDataGen

## Generate sample data based on the parameters from before for each simulation

In [10]:

```
def ExpDataGen():
    nVal = 100
    # Generates ExpData vector--used in ODE solver
    global ExpData
```

```
    ExpData = np.arange(0,index,1)
    # Sets iteration markers
    m = 0
    for x in np.arange(0,index,1):
        # Generates a normal distribution based on the SMD and SD from that specific da
        RandGen = np.random.normal(SMD[m], SDpool[m], nVal)
        # Randomly pulls a value from that normal distirbution to input into model
        randCho = random.choice(RandGen)
        # Assigns random value into experimental data
        global SMD_0
        SMD_0 = randCho
        ExpData[m] = randCho
        # Iterates m
        m = m + 1
```

# RMSE_vs_a_b

### Input: a, b (based on the simulations)

### Outputs: 3D plot of RMSE versus varied a and b values, RMSE versus varied a, and RMSE versus varied b

### shape of changes of RMSE due to a and b noted (polynomial, linear)

In [11]:

```
def RMSE_vs_a_b(a, b):
    # Creates vectors of possible a and b values based on above simulations
    a_poss = np.linspace(a * 0.1, a * 2,10)
    b_poss = np.linspace(b * 0.1, b * 2, 10)

    #extract data file
    ng = group.find('_')
    if group[ng + 1] == 'd':
        new_group = group[: ng + 5]
    elif group[ng + 1] == 'u':
        new_group = group[: ng + 3]
    else:
        new_group = group[: ng]
    new_path = 'Data/' + new_group + '.csv'

    #get the parsed data
    raw_data(new_path)

    ExpDataGen()

    #need to get the aliasing correct on the graphs in order to save them
    # Calculates RMSE for each possible a and b value
    fig = plt.figure(figsize=(9, 9))
    plt.rcParams.update({'font.size': 14})
    ax = plt.axes(projection='3d')
    ax.set_xlabel('A Value')
    ax.set_ylabel("B Value")
    ax.set_zlabel("RMSE")
    plt.title("3D plot of " + group + " (" + choice+ ")")
```

```
    #set colors for the different datasets to see the contouring over time
    colors = ['red', 'orangered', 'orange', 'yellow', 'greenyellow', 'green', 'blue', '

    #reset index every time
    ind = 0
    for a in a_poss:
        fill = colors[ind]
        ind += 1
        for b in b_poss:
            RMSE_val = RMSE([a,b])
            ax.plot3D(a,b, -RMSE_val, 'co', color = fill)
    fig.savefig('Final_data/Final_data_sensitivity/3D_Plots/' + group + "_" + choice +

    ind = 0
    # Generates a 2D figure for a versus RMSE to be plotted for easier visualization
    fig2, ax2 = plt.subplots(figsize=(9, 9))
    plt.rcParams.update({'font.size': 14})
    for a in a_poss:
        fill = colors[ind]
        ind += 1
        for b in b_poss:
            RMSE_val = RMSE([a,b])
            plt.plot(a, RMSE_val, 'co', color = fill)
    ax2.set_xlabel('A Value')
    ax2.set_ylabel("RMSE")
    plt.title("2D plot (Constant A) of " + group + " (" + choice+ ")")


    plt.xlabel("Possible a values")
    plt.ylabel("RMSE")
    fig2.savefig('Final_data/Final_data_sensitivity/A_Variation/' + group + "_" + choic

    ind = 0
    # Generates a 2D figure for b versus RMSE to be plotted for easier visualization
    fig3, ax3 = plt.subplots(figsize=(9, 9))
    plt.rcParams.update({'font.size': 14})
    for a in a_poss:
        fill = colors[ind]
        ind += 1
        for b in b_poss:
            RMSE_val = RMSE([a,b])
            plt.plot(b, RMSE_val, 'co', color = fill)
    ax3.set_xlabel('B Value')
    ax3.set_ylabel("RMSE")
    plt.title("2D plot (Constant B) of " + group + " (" + choice + ")")
    fig3.savefig('Final_data/Final_data_sensitivity/B_Variation/' + group + "_" + choic
```

# sensitivity_a_b

## Input: a_value, b_value (based on the simulations)

## Output: Graph of the SMD versus changes in a and b

## Change in SMD<2 over a 20% increase in a and b noted as a stable model

In [12]:

```
def sensitivity_a_b(a_value, b_value):
    SMD_i0 = sum(SMD) / len(SMD)

    #plot
    fig4, ax4 = plt.subplots(figsize=(9, 9))

    #get starting value
    SMD_0 = odeint(dSMDdFq, SMD_i0, steps, args = (a_value, b_value))

    colors = ['red', 'green', 'blue', 'purple']
    percentages = [1.05, 1.1, 1.15, 1.2]
    labels = ['5%', '10%', '15%', '20%']

    for w in range(len(percentages)):
        # Calculates SMD if a is altered by a percentage
        SMD_a = odeint(dSMDdFq, SMD_i0, steps, args = (a_value*percentages[w] ,b_value)

        SMD_a0 = SMD_a - SMD_0

        # plot the a data
        plt.plot(steps, SMD_a0, 'm:', label = "a (" + labels[w] + ")", color = colors[w

        # Calculates SMD if b is altered
        SMD_b = odeint(dSMDdFq, SMD_i0, steps, args = (a_value, b_value*percentages[w])
        # Determined the perturbation in SMD based on error caused by a being 5% change

        SMD_b0 = SMD_b - SMD_0
        plt.plot(steps, SMD_b0, label = "b (" + labels[w] + ")",  color = colors[w]);

        if w == (len(percentages) - 1):
            ret = [SMD_a0[-1], SMD_b0[-1]]


    # Labels the plot
    plt.rcParams.update({'font.size': 14})
    if 'magnitude' in group:
        additional = 'Magnitude (mT)'
    elif 'frequency' in group:
        additional = 'Frequency (Hz)'
    elif 'percent' in group:
        additional = 'Percent Time Exposed (%)'
    else:
        additional = 'Total Time Exposed (Hours)'
    ax4.set_xlabel(additional)
    ax4.set_ylabel("Sensitivity of SMD")
    ax4.legend(loc='lower left')
    plt.title("Sensitivity Analysis of A/B Values for " + group + " (" + choice+ ")")
    fig4.savefig('Final_data/Final_data_sensitivity/Sensitivity_A_B/' + group + "_" + c

    return ret
```

# Call functions appropriately for sensitivity analyses

# RMSE_vs_a_b(), sensitivity_a_b()

# Also writes out the final value at the 20% increase in a/b for sensitivity analysis purpsoed

In [14]:

```
filepath = 'Final_data/p_values/'

#items of interest
#fill in
#lst = ['viability_Neural_magnitude', 'regeneration_Neural_frequency', 'proliferation_N

#add back in 'Linear' to choice
lst = ['Growth_frequency',\
       'Growth_magnitude', \
       'Growth_total_time',\
       'Growth_percent', \
       'Neuron_magnitude',\
       'Neuron_total_time',\
       'Viability_magnitude',\
       'Viability_percent',\
       'Viability_total_time']

sen = [['Group', 'Linear/Polynomial', 'a value at 20%', 'b value at 20%']]

for group in lst:
    for choice in ['Linear','Polynomial']:
        file = filepath + group + "_" + choice + '.csv'
        print(file)
        with open(file) as csv_file:
            csv_reader = csv.reader(csv_file, delimiter=',')
            rows = list(csv_reader)
        a_value = float(rows[1][1])
        b_value = float(rows[2][1])
        RMSE_vs_a_b(a_value, b_value)
        r = sensitivity_a_b(a_value, b_value)
        sen.append([group] + [choice] + [r[0]] + [r[1]])


#write the accumulated file
with open("Last_value.csv", "w", newline='') as f:
    writer = csv.writer(f,delimiter=',')
    writer.writerows(sen)
```

```
Final_data/p_values/Growth_frequency_Linear.csv
Final_data/p_values/Growth_frequency_Polynomial.csv
Final_data/p_values/Growth_magnitude_Linear.csv
Final_data/p_values/Growth_magnitude_Polynomial.csv
Final_data/p_values/Growth_total_time_Linear.csv
Final_data/p_values/Growth_total_time_Polynomial.csv
<ipython-input-11-0b5ddc6eb400>:26: RuntimeWarning: More than 20 figures have been opene
d. Figures created through the pyplot interface (`matplotlib.pyplot.figure`) are retaine
d until explicitly closed and may consume too much memory. (To control this warning, see
the rcParam `figure.max_open_warning`).
  fig = plt.figure(figsize=(9, 9))
Final_data/p_values/Growth_percent_Linear.csv
Final_data/p_values/Growth_percent_Polynomial.csv
Final_data/p_values/Neuron_magnitude_Linear.csv
Final_data/p_values/Neuron_magnitude_Polynomial.csv
Final_data/p_values/Neuron_total_time_Linear.csv
```

```
Final_data/p_values/Neuron_total_time_Polynomial.csv
Final_data/p_values/Viability_magnitude_Linear.csv
Final_data/p_values/Viability_magnitude_Polynomial.csv
Final_data/p_values/Viability_percent_Linear.csv
Final_data/p_values/Viability_percent_Polynomial.csv
Final_data/p_values/Viability_total_time_Linear.csv
Final_data/p_values/Viability_total_time_Polynomial.csv
```

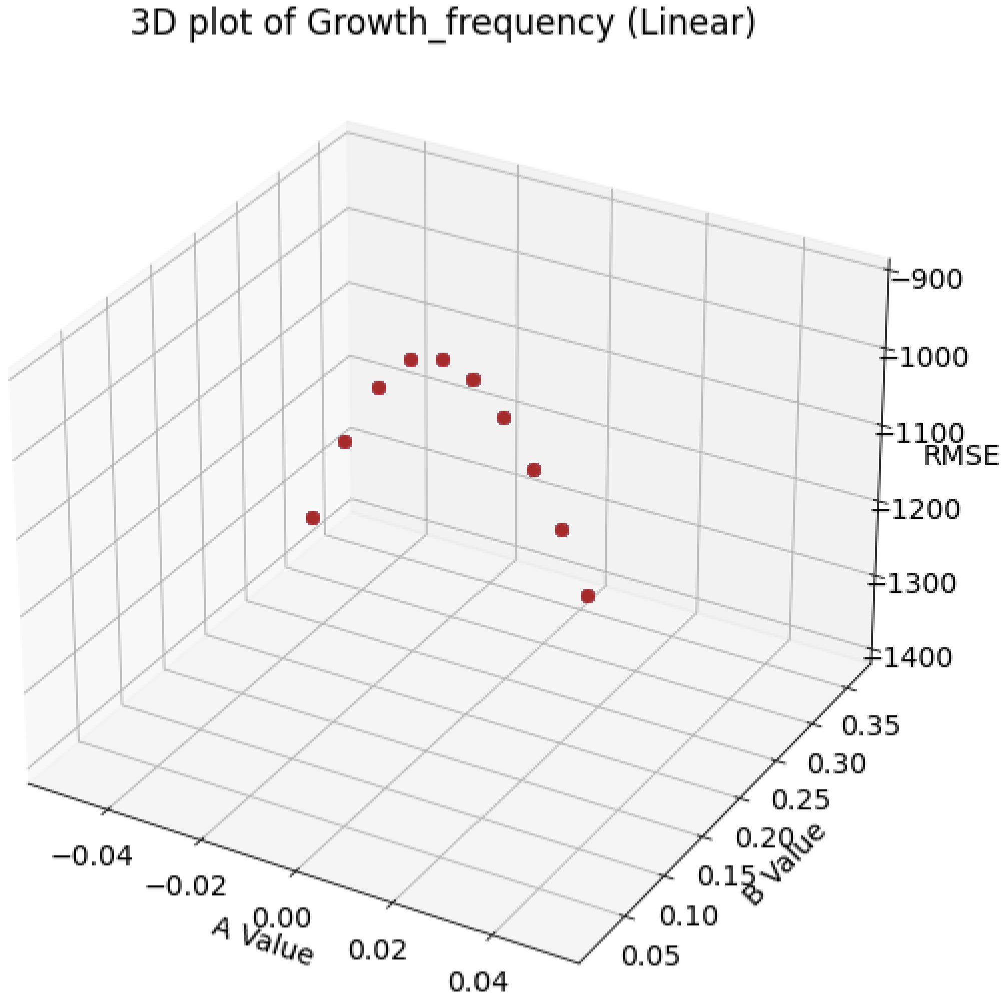

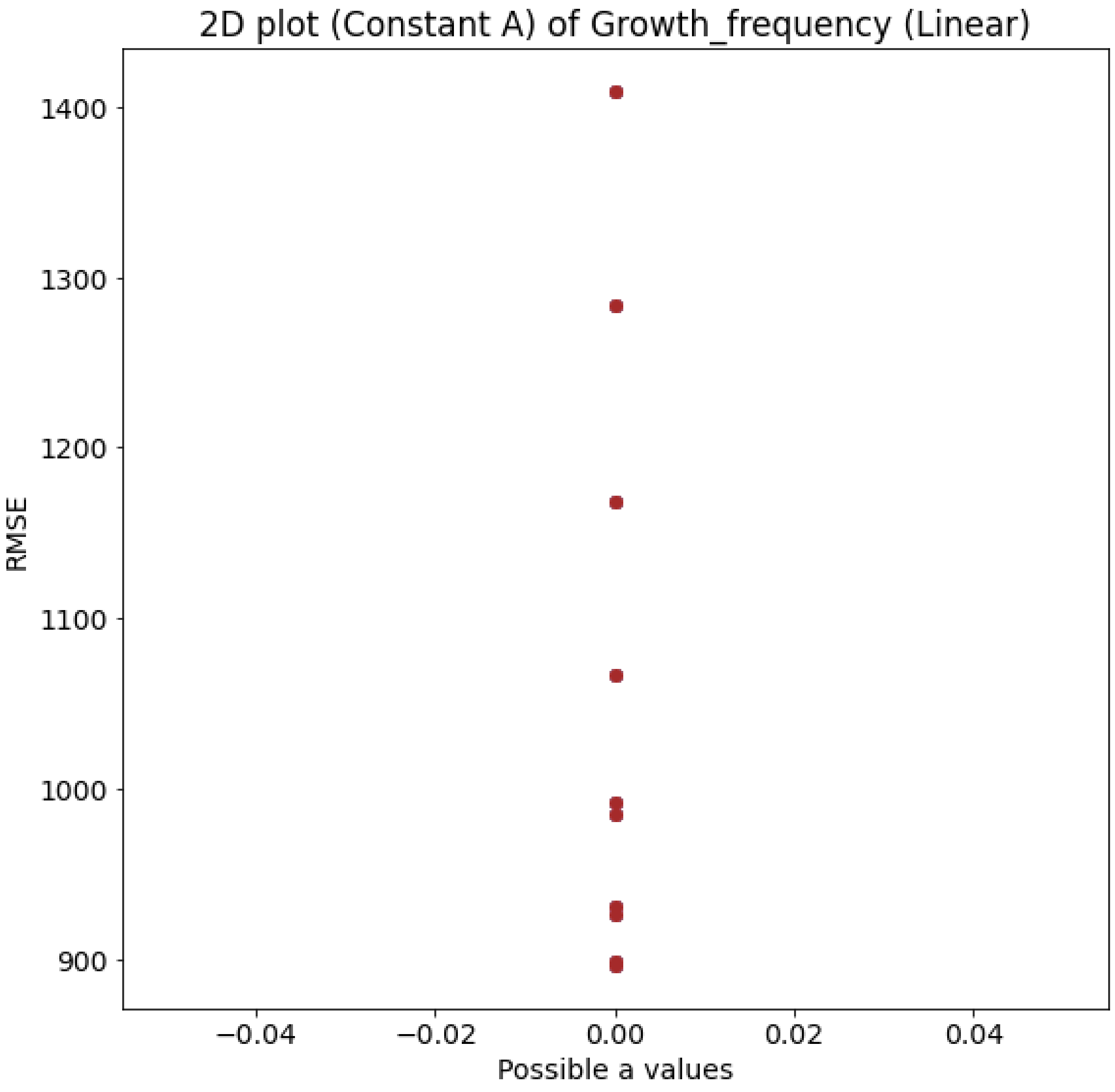
2D plot (Constant A) of Growth_frequency (Linear)
RMSE
1400
1300
1200
1100
1000
900
−0.04
−0.02
0.00
0.02
0.04
Possible a values

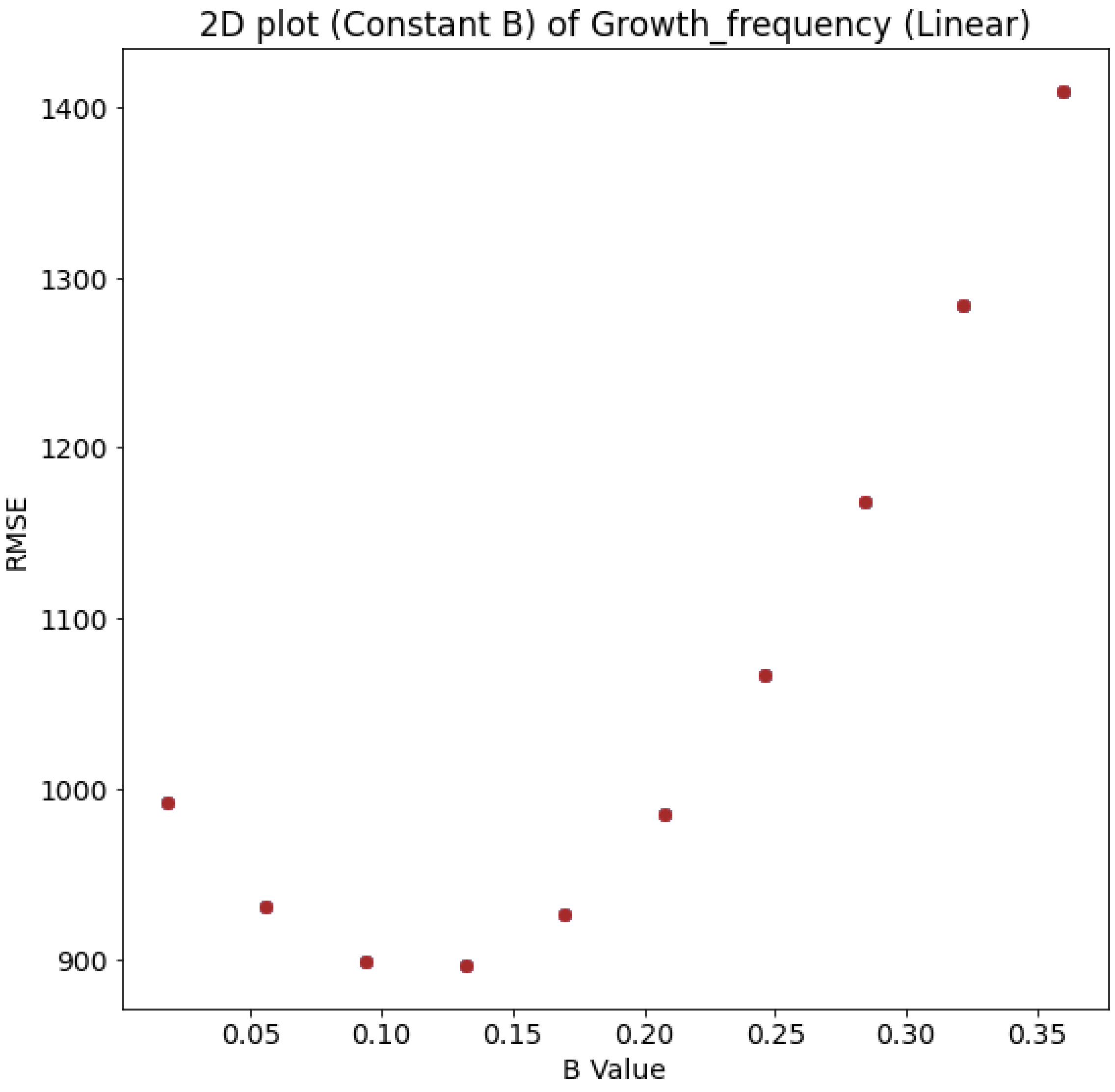
2D plot (Constant B) of Growth_frequency (Linear)
RMSE
1400
1300
1200
1100
1000
900
0.05
0.10
0.15
0.20
0.25
0.30
0.35
B Value

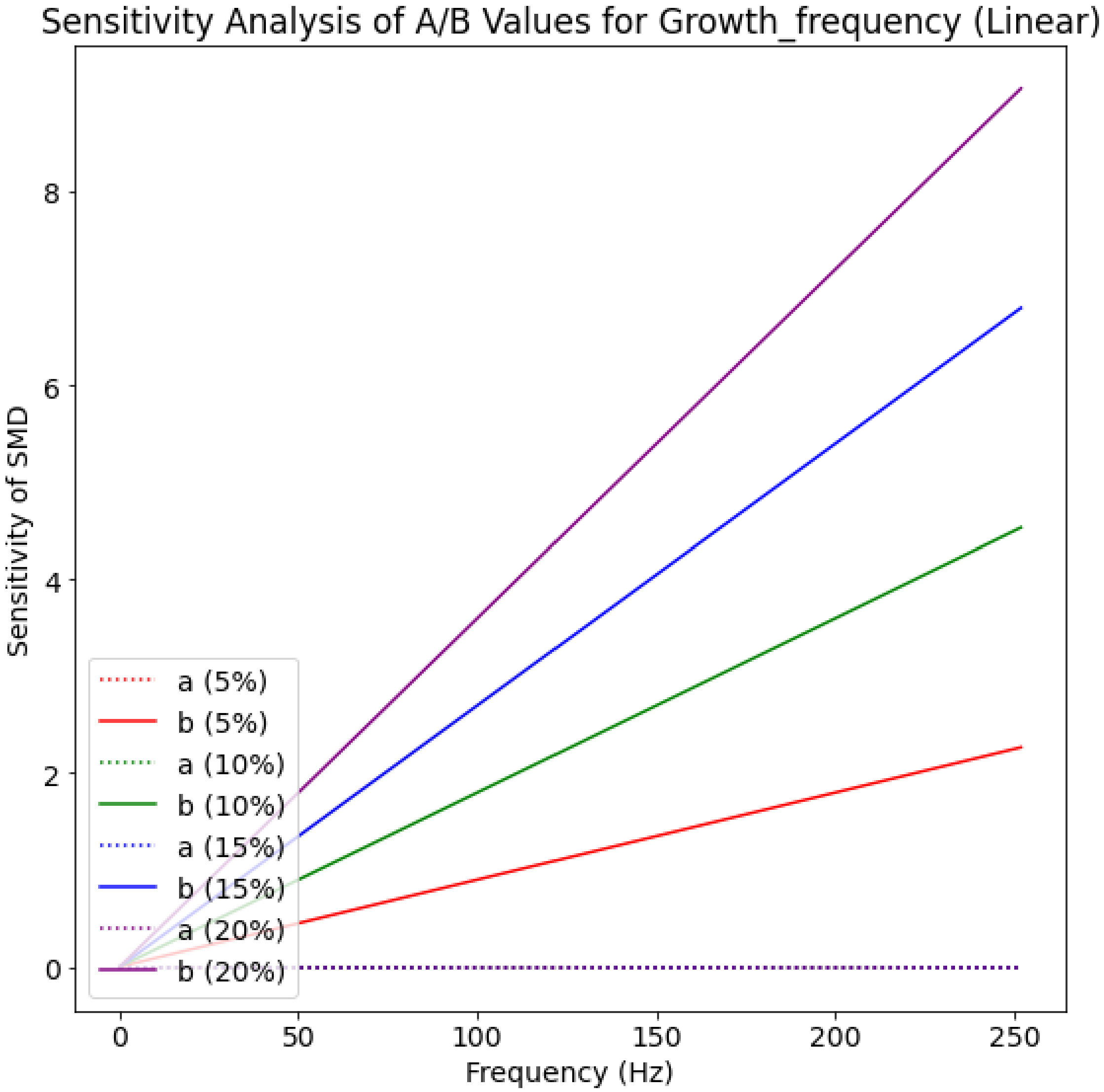
Sensitivity Analysis of A/B Values for Growth_frequency (Linear)
Sensitivity of SMD
Frequency (Hz)
0
2
4
6
8
0
50
100
150
200
250
a (5%)
b (5%)
a (10%)
b (10%)
a (15%)
b (15%)
a (20%)
b (20%)

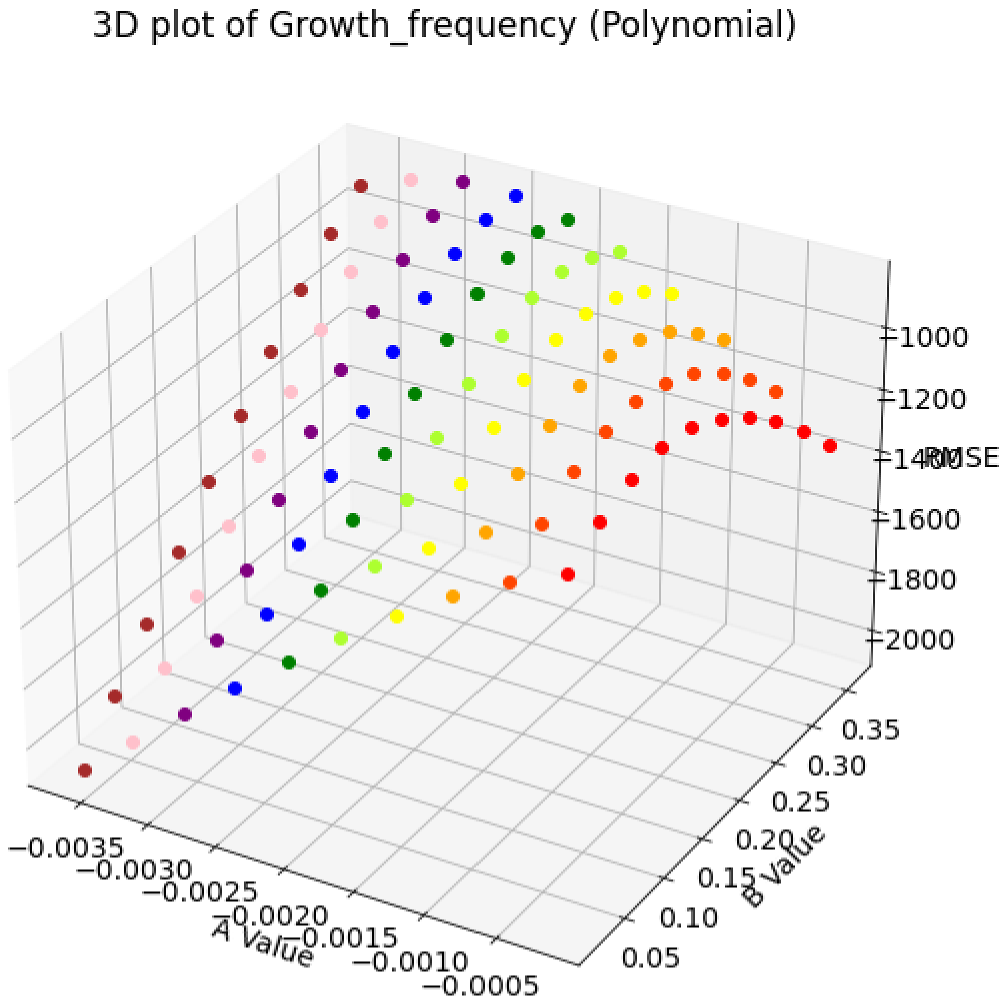
3D plot of Growth_frequency (Polynomial)
RMSE
1000
1200
1400
1600
1800
2000
A Value
−0.0035
−0.0030
−0.0025
−0.0020
−0.0015
−0.0010
−0.0005
B Value
0.05
0.10
0.15
0.20
0.25
0.30
0.35

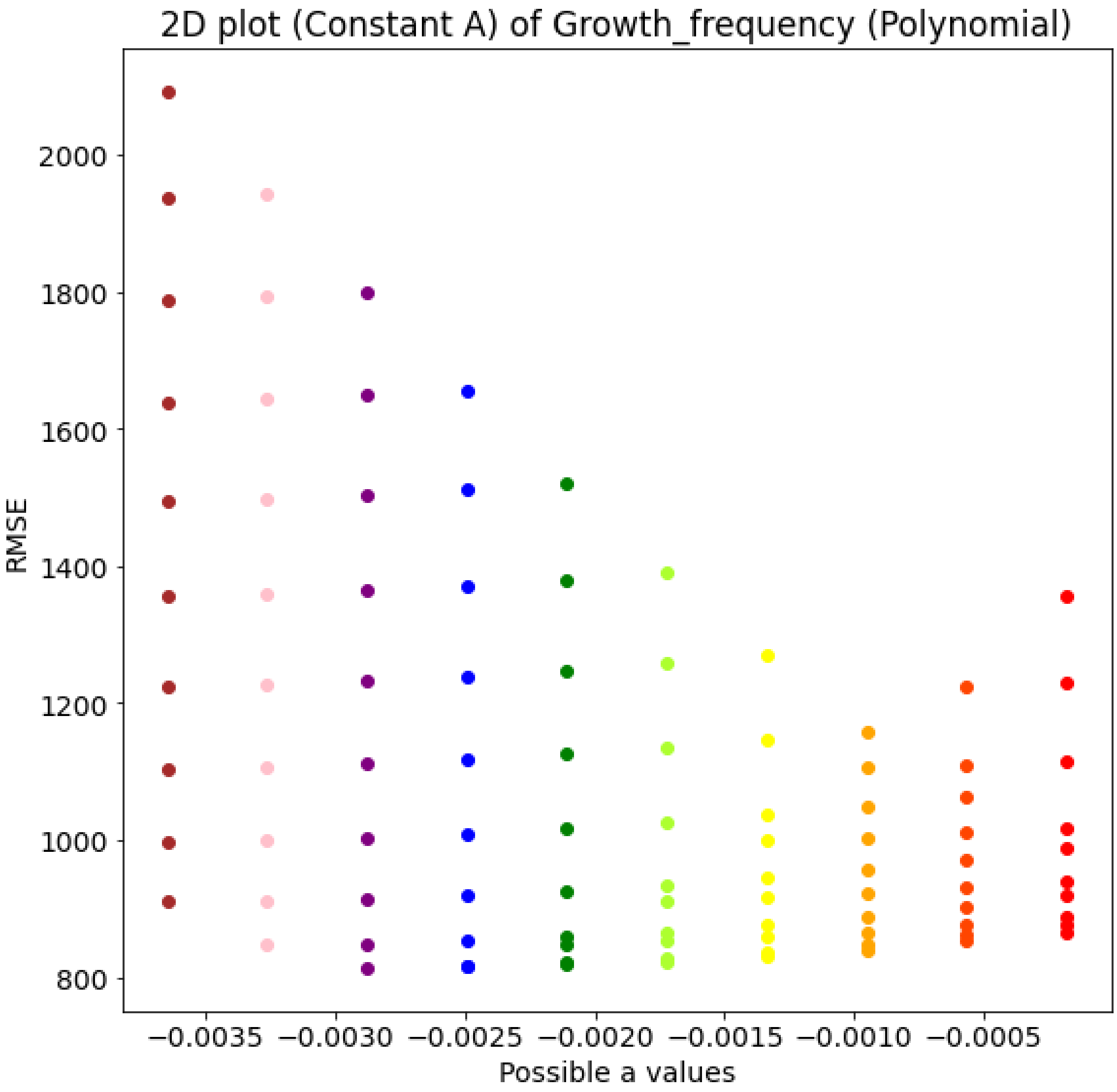
2D plot (Constant A) of Growth_frequency (Polynomial)
RMSE
2000
1800
1600
1400
1200
1000
800
−0.0035
−0.0030
−0.0025
−0.0020
−0.0015
−0.0010
−0.0005
Possible a values

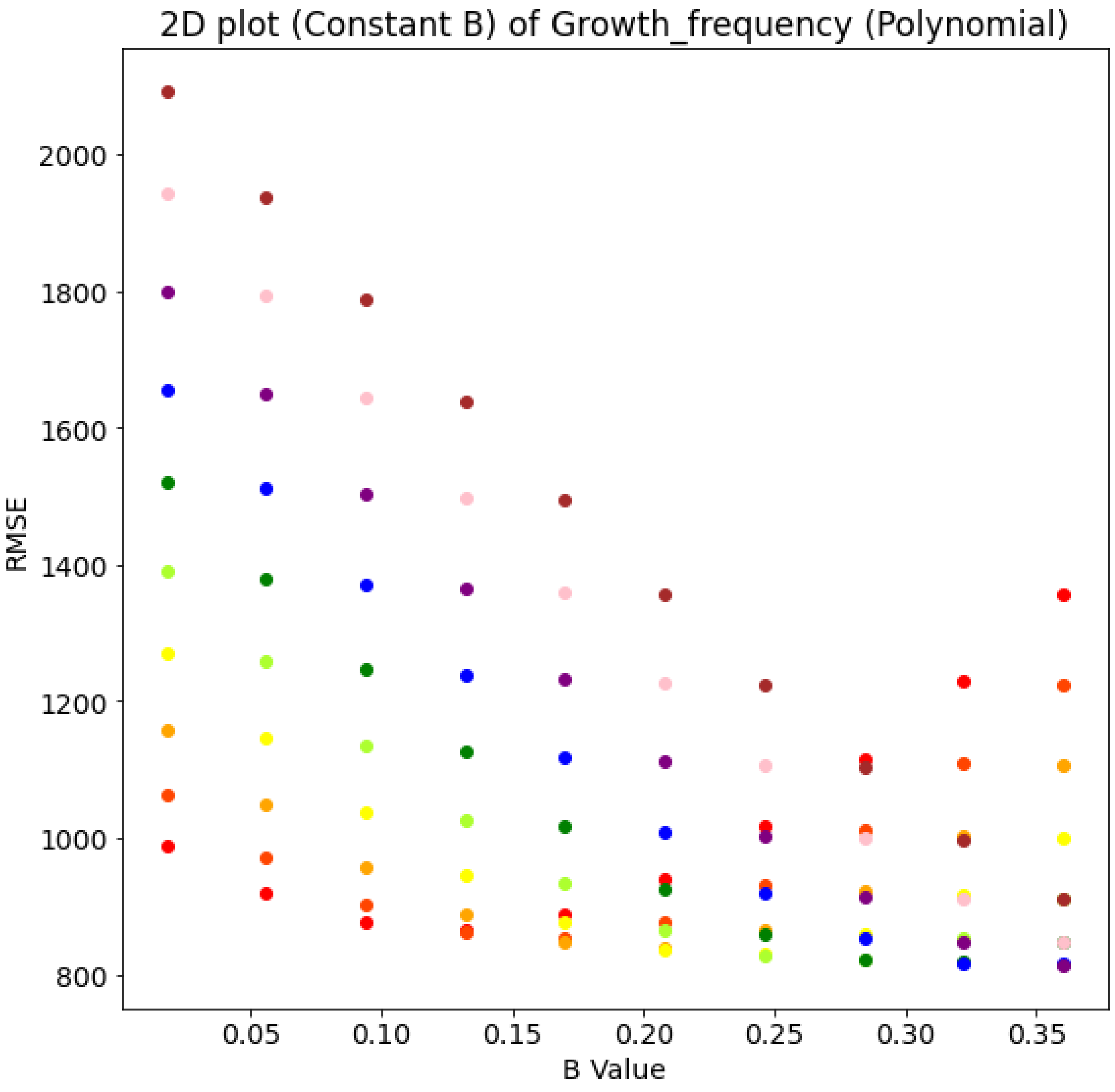
2D plot (Constant B) of Growth_frequency (Polynomial)
RMSE
2000
1800
1600
1400
1200
1000
800
0.05
0.10
0.15
0.20
0.25
0.30
0.35
B Value

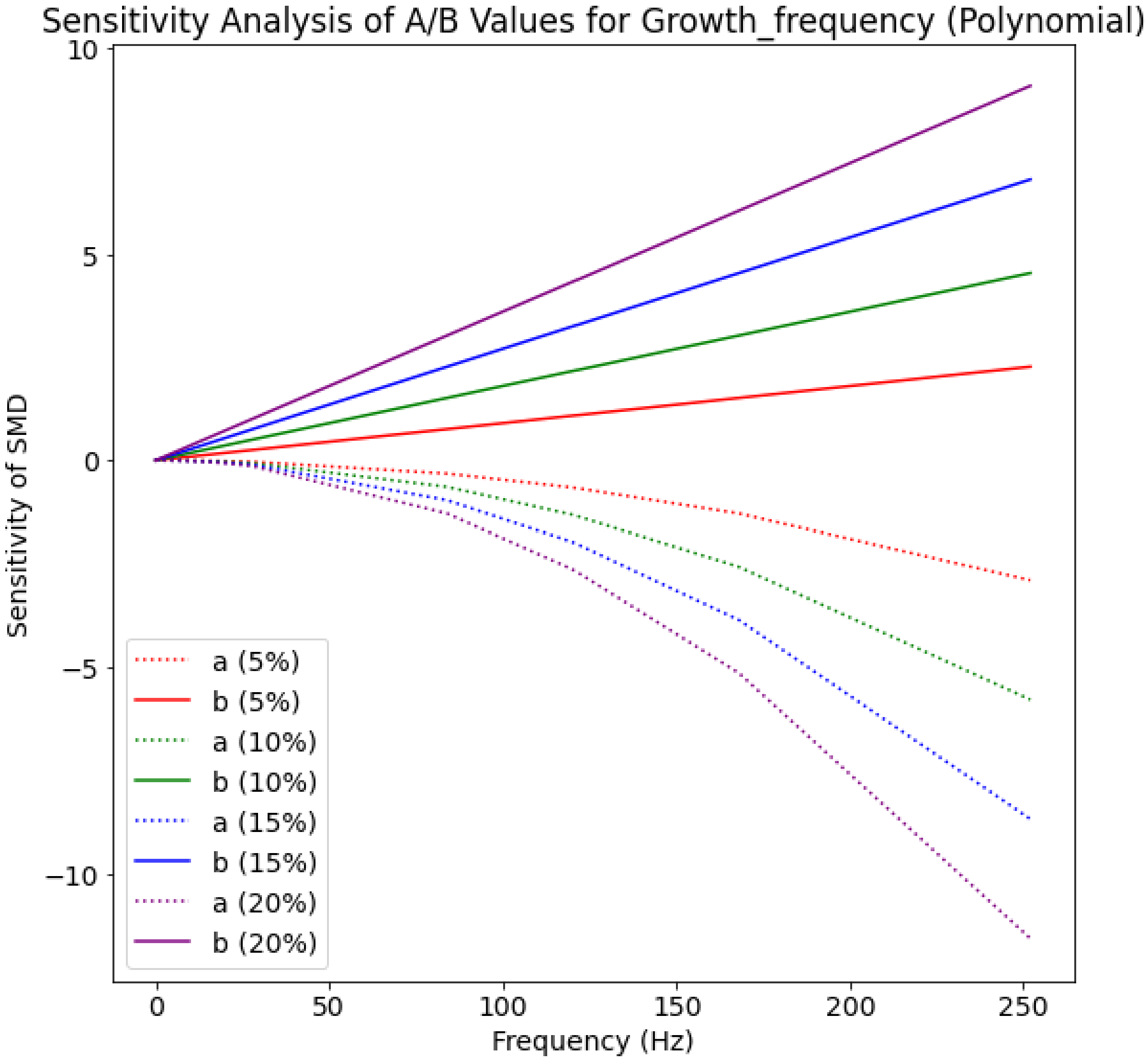
Sensitivity Analysis of A/B Values for Growth_frequency (Polynomial)
Sensitivity of SMD
Frequency (Hz)
10
5
0
−5
−10
0
50
100
150
200
250
a (5%)
b (5%)
a (10%)
b (10%)
a (15%)
b (15%)
a (20%)
b (20%)

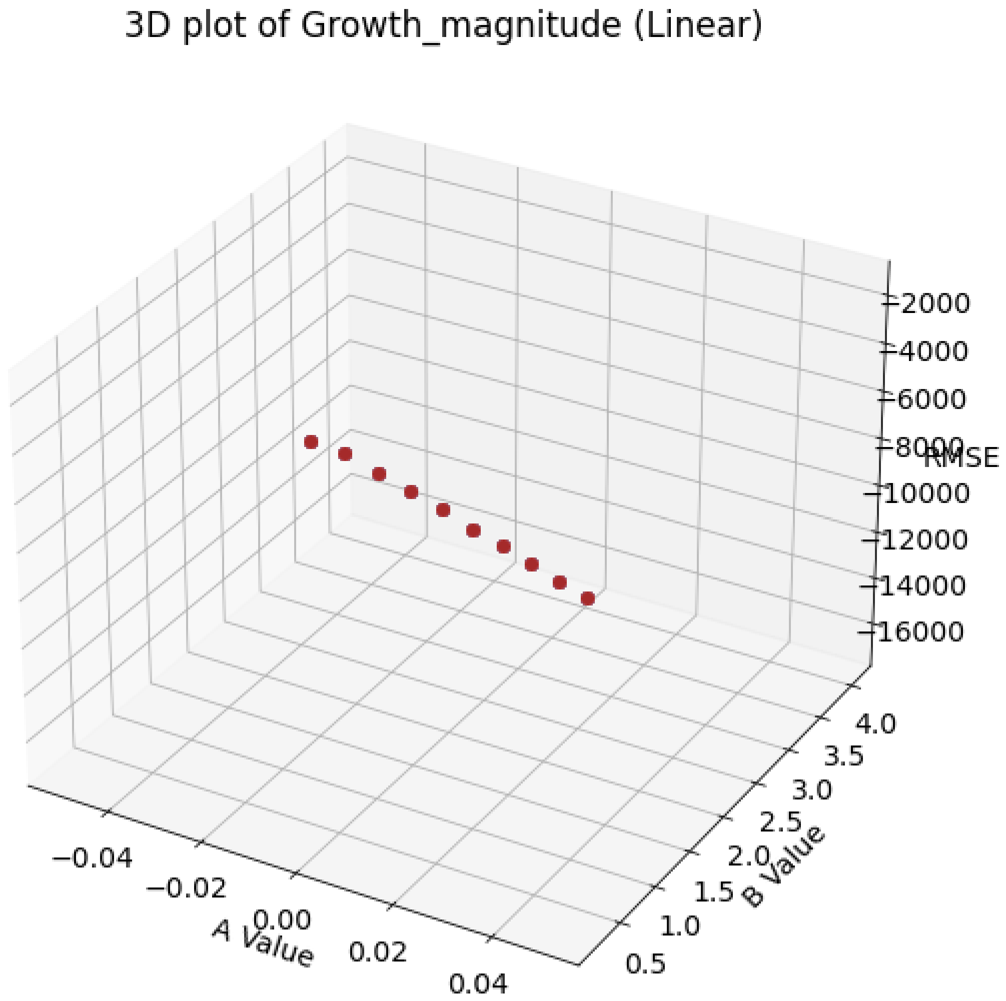
3D plot of Growth_magnitude (Linear)
RMSE
2000
4000
6000
8000
10000
12000
14000
16000
A Value
−0.04
−0.02
0.00
0.02
0.04
B Value
0.5
1.0
1.5
2.0
2.5
3.0
3.5
4.0

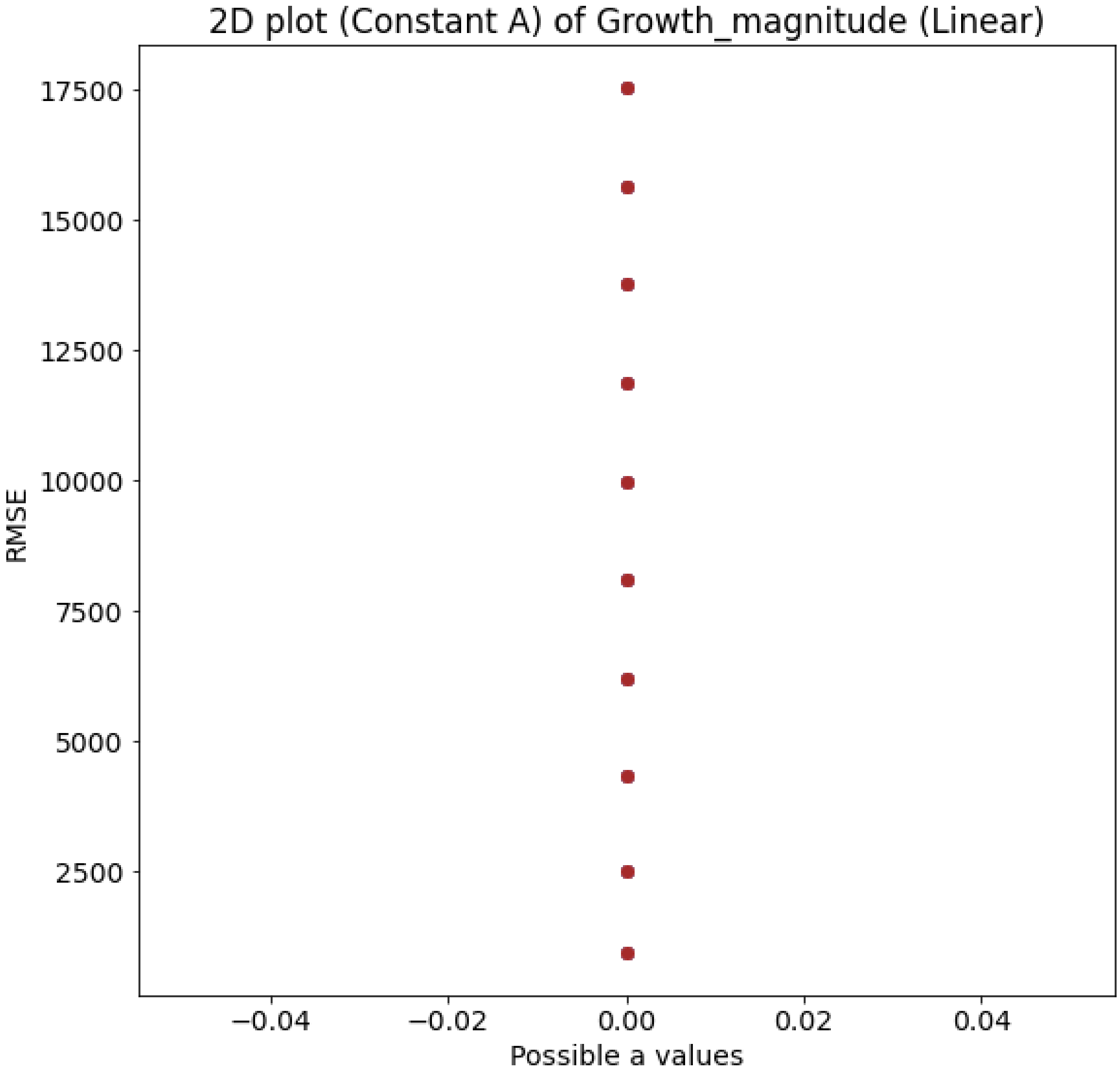
2D plot (Constant A) of Growth_magnitude (Linear)
RMSE
17500
15000
12500
10000
7500
5000
2500
−0.04
−0.02
0.00
0.02
0.04
Possible a values

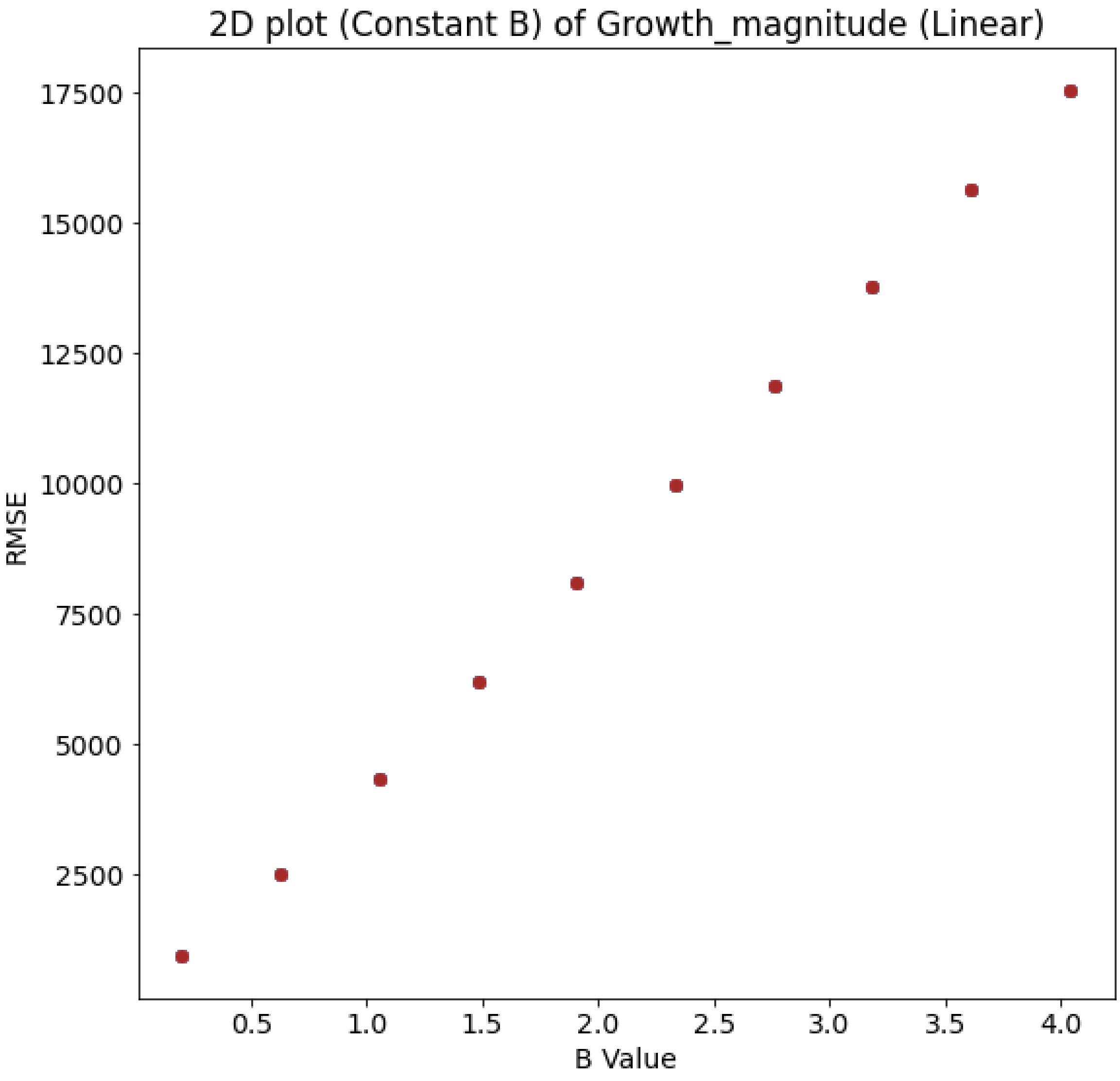
2D plot (Constant B) of Growth_magnitude (Linear)
RMSE
B Value
17500
15000
12500
10000
7500
5000
2500
0.5
1.0
1.5
2.0
2.5
3.0
3.5
4.0

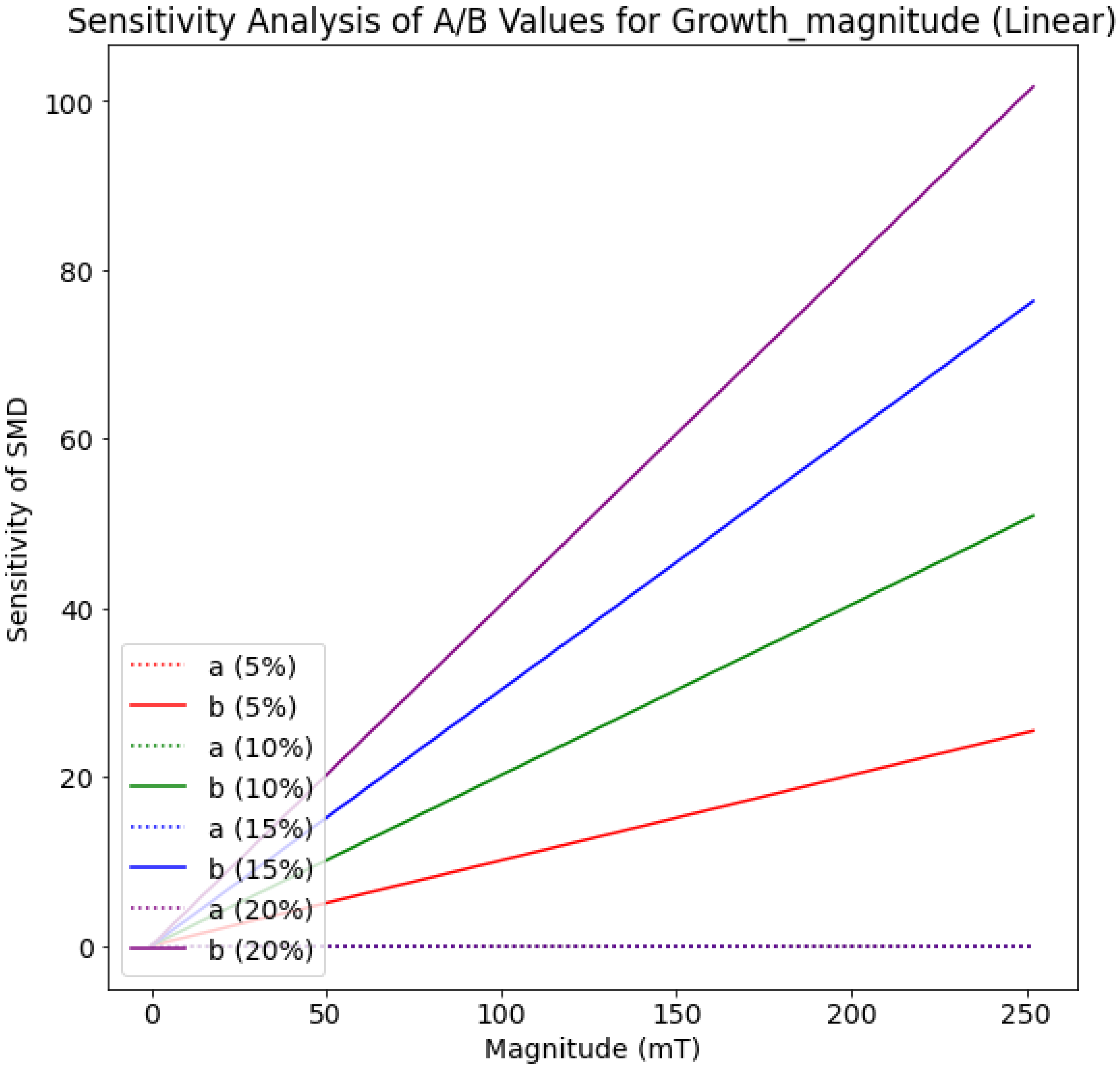
Sensitivity Analysis of A/B Values for Growth_magnitude (Linear)
Sensitivity of SMD
Magnitude (mT)
100
80
60
40
20
0
0
50
100
150
200
250
a (5%)
b (5%)
a (10%)
b (10%)
a (15%)
b (15%)
a (20%)
b (20%)

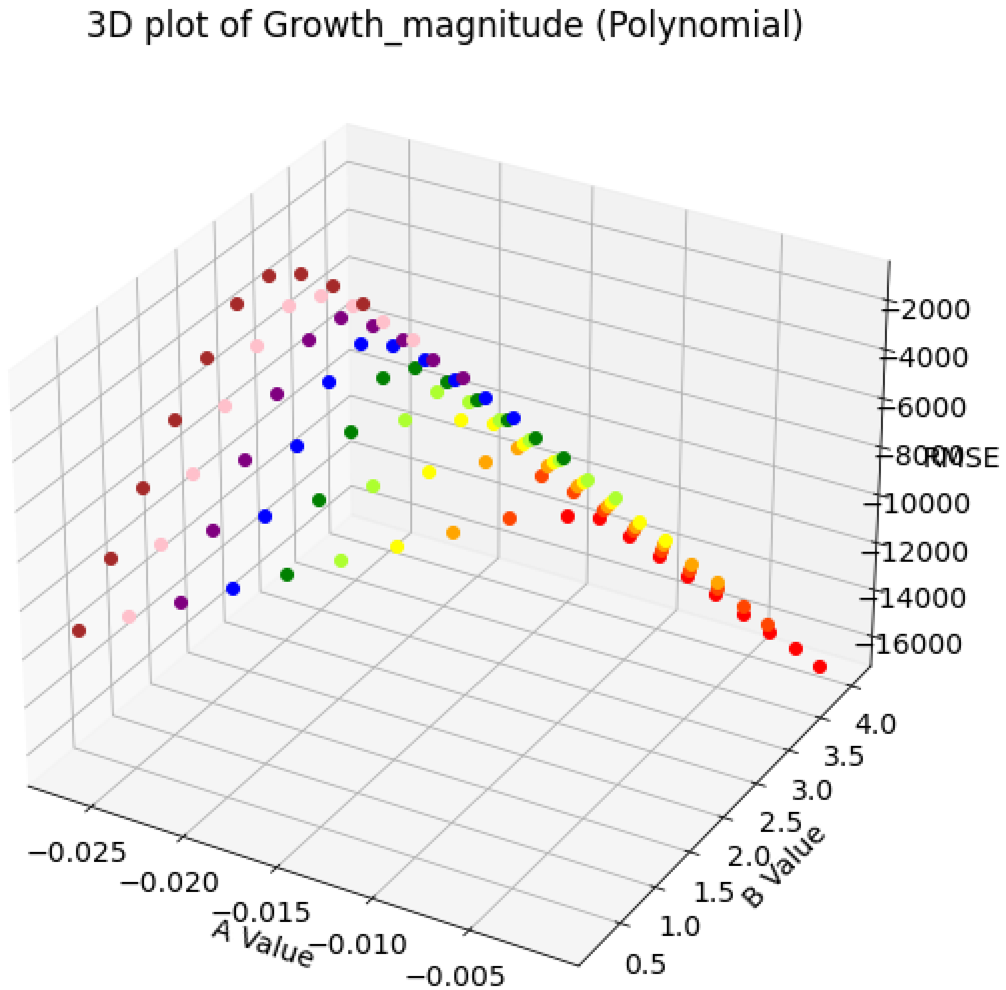
3D plot of Growth_magnitude (Polynomial)
A Value
B Value
RMSE
−0.025
−0.020
−0.015
−0.010
−0.005
0.5
1.0
1.5
2.0
2.5
3.0
3.5
4.0
2000
4000
6000
8000
10000
12000
14000
16000

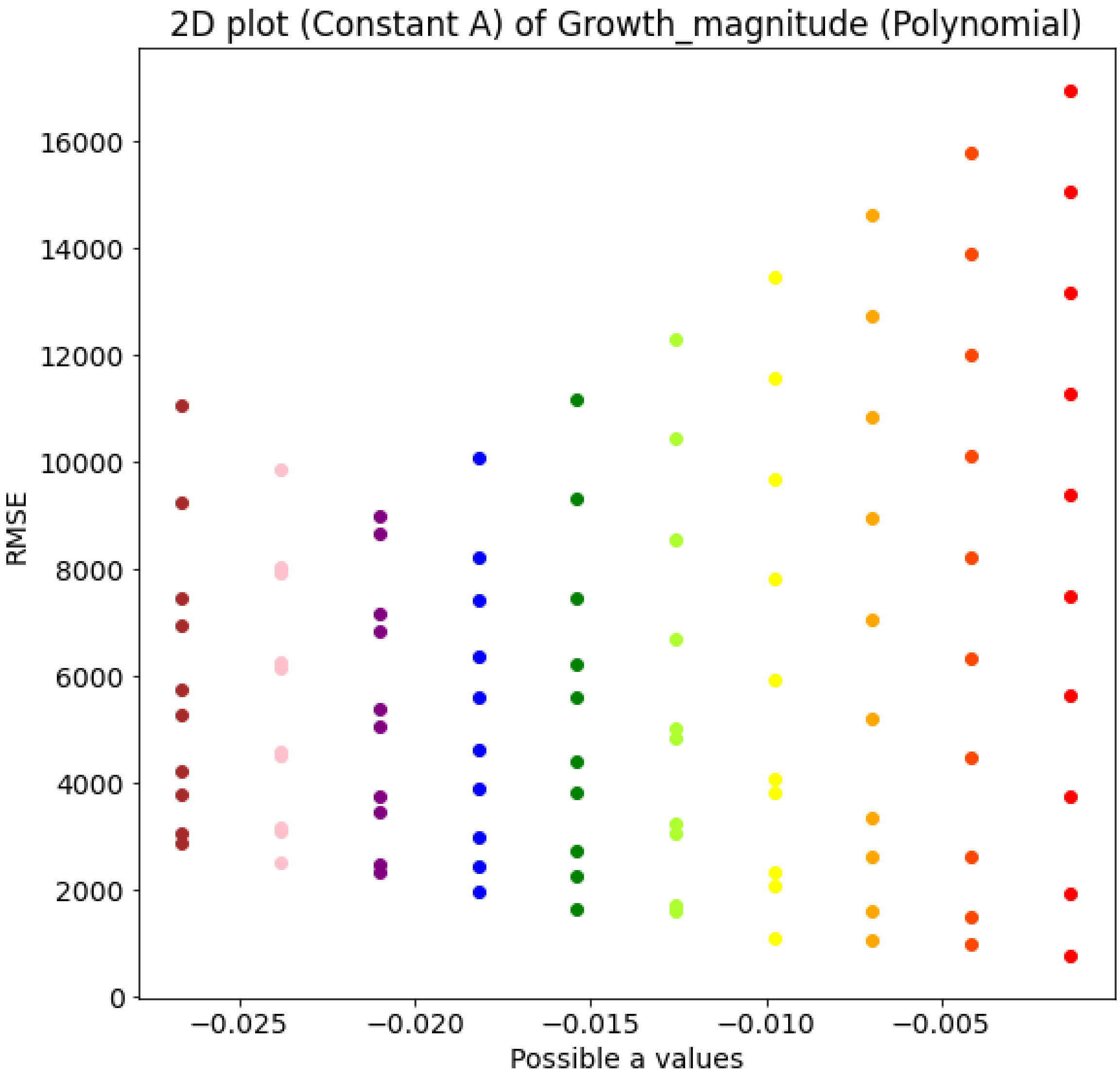
2D plot (Constant A) of Growth_magnitude (Polynomial)
RMSE
Possible a values
16000
14000
12000
10000
8000
6000
4000
2000
0
−0.025
−0.020
−0.015
−0.010
−0.005

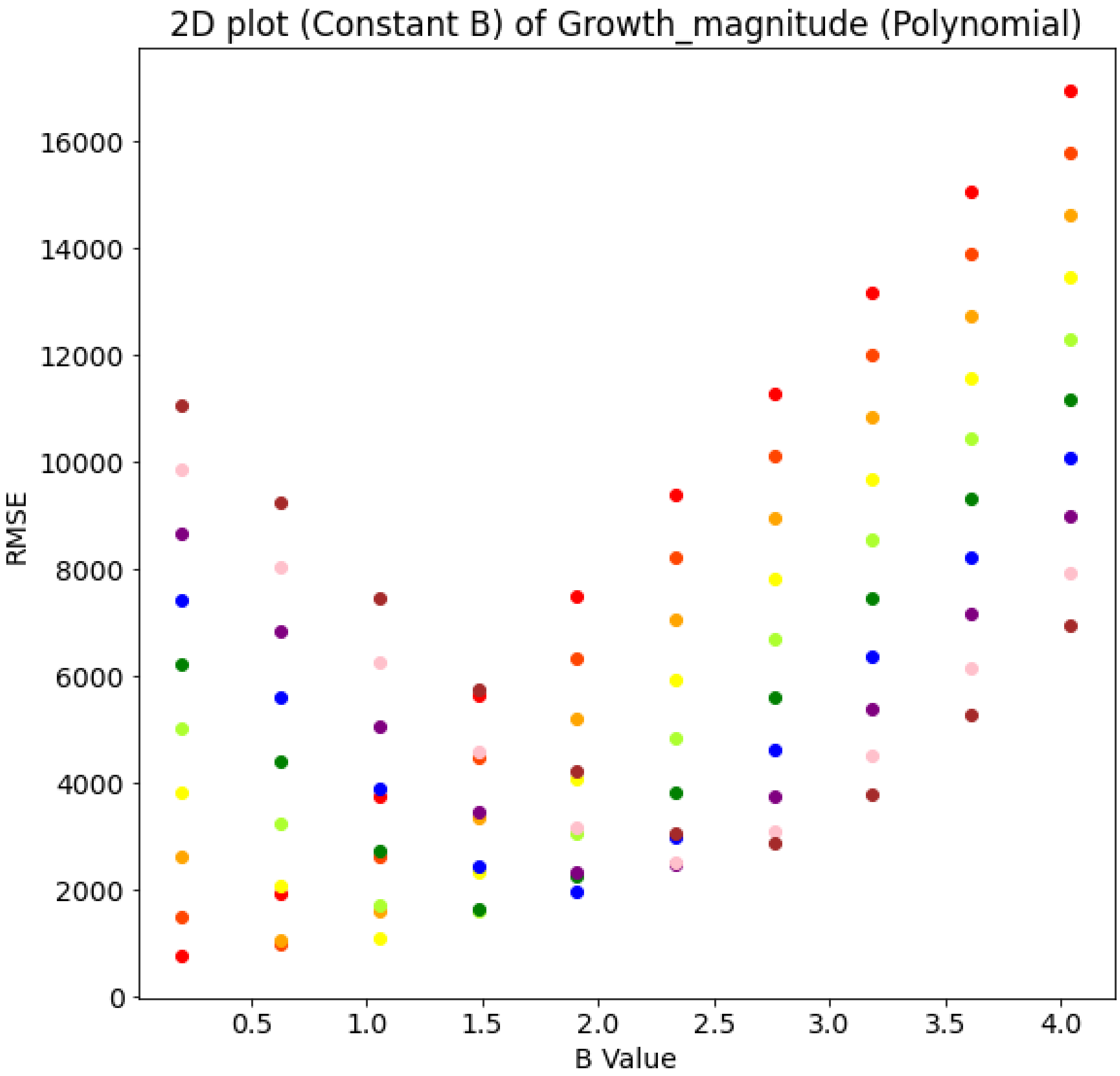
2D plot (Constant B) of Growth_magnitude (Polynomial)
RMSE
B Value
0
2000
4000
6000
8000
10000
12000
14000
16000
0.5
1.0
1.5
2.0
2.5
3.0
3.5
4.0

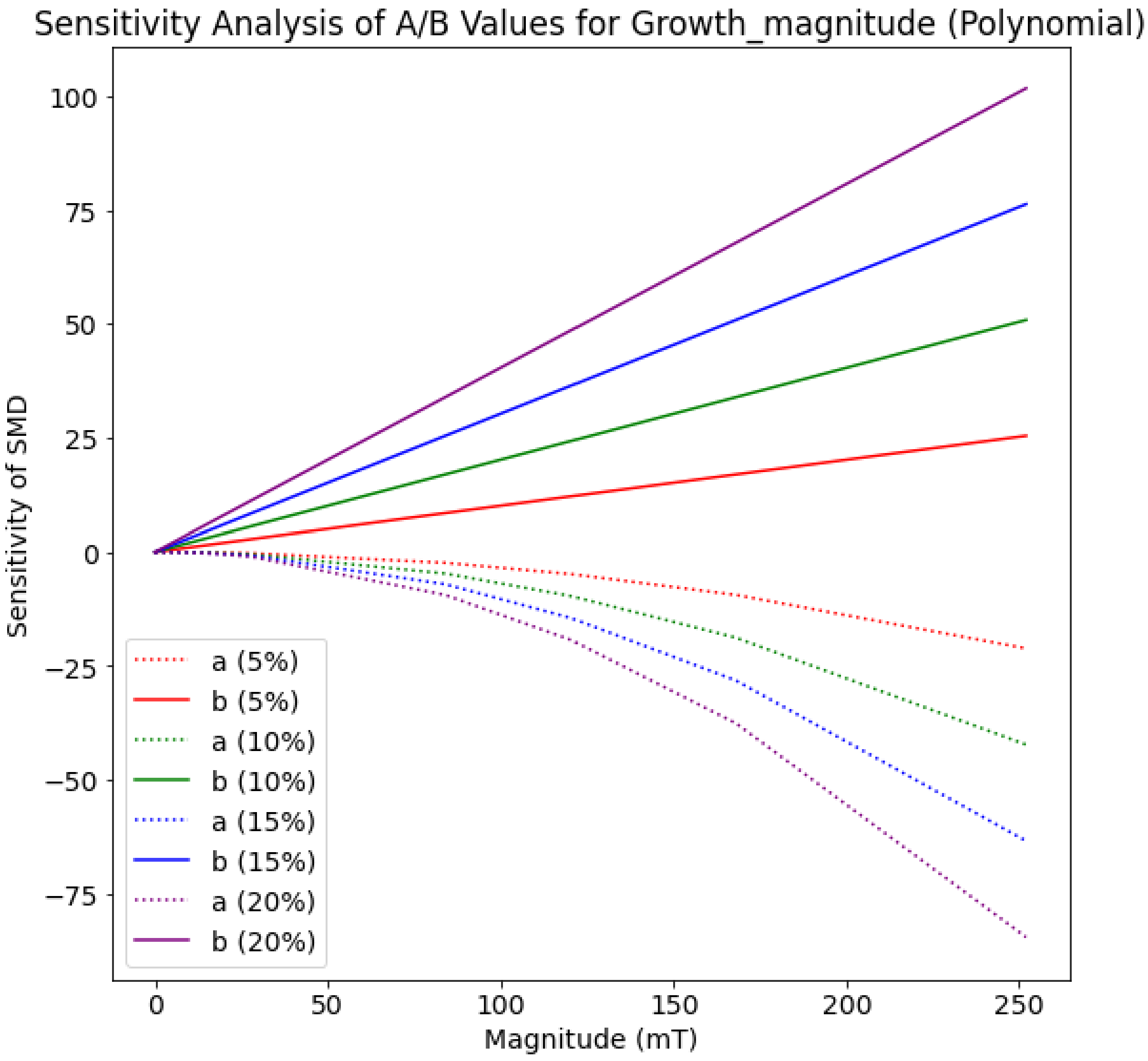
Sensitivity Analysis of A/B Values for Growth_magnitude (Polynomial)
Sensitivity of SMD
Magnitude (mT)
100
75
50
25
0
−25
−50
−75
0
50
100
150
200
250
a (5%)
b (5%)
a (10%)
b (10%)
a (15%)
b (15%)
a (20%)
b (20%)

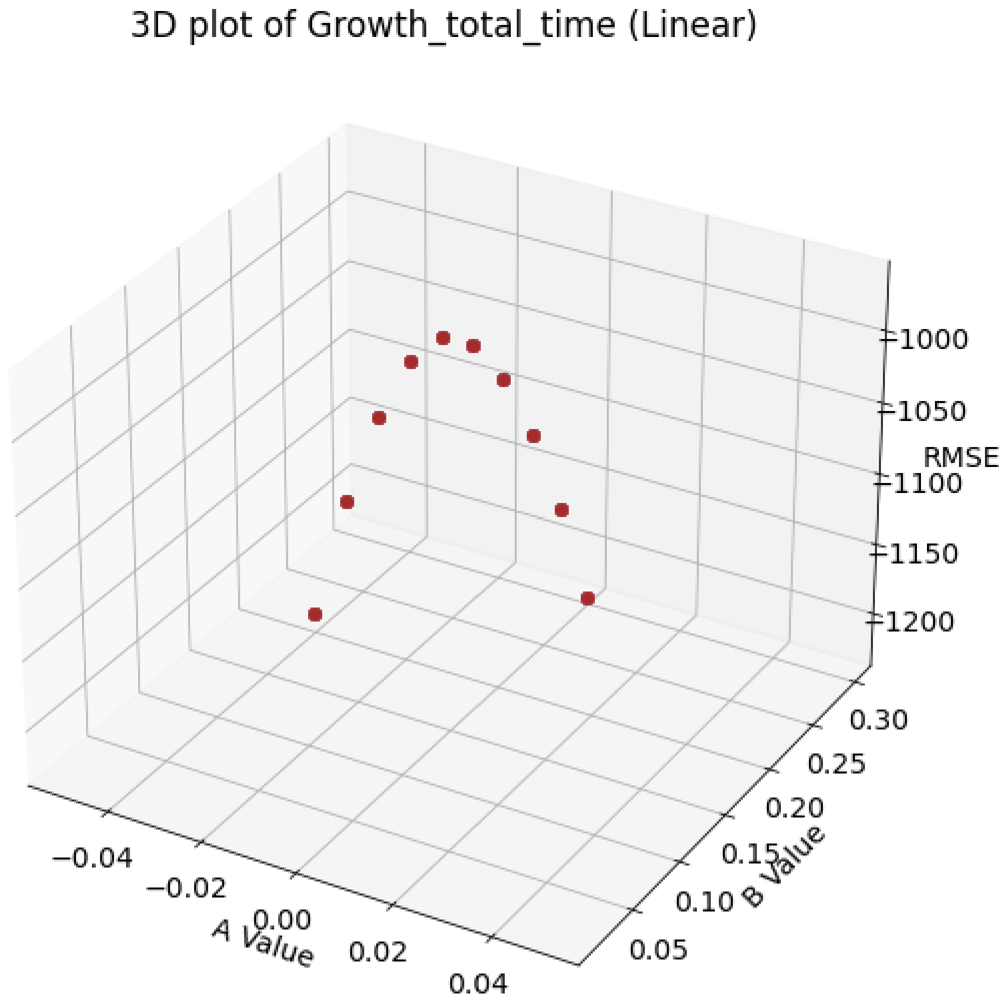
3D plot of Growth_total_time (Linear)
RMSE
1000
1050
1100
1150
1200
0.30
0.25
0.20
0.15
0.10
0.05
B Value
−0.04
−0.02
0.00
0.02
0.04
A Value

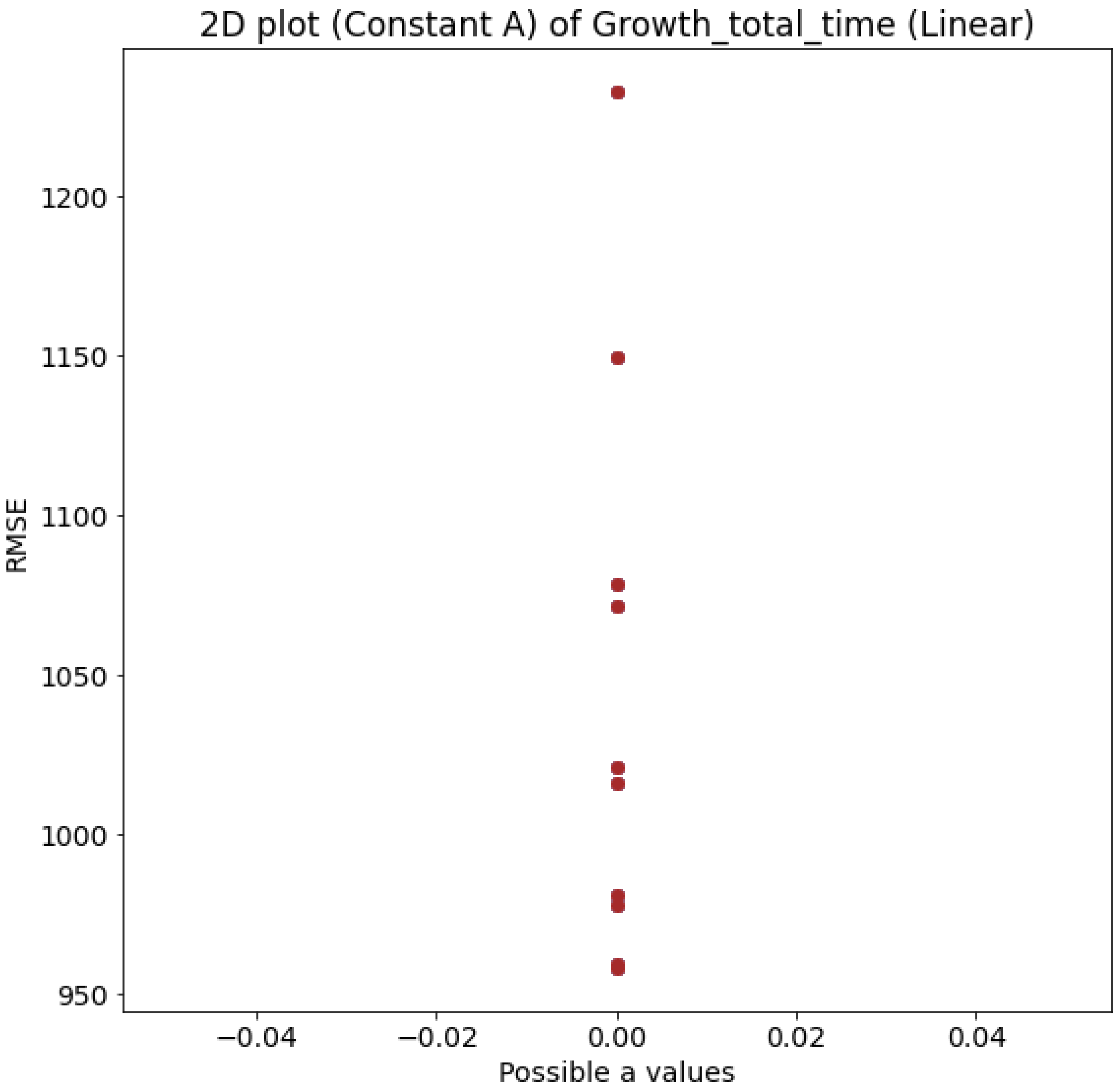
2D plot (Constant A) of Growth_total_time (Linear)
RMSE
1200
1150
1100
1050
1000
950
−0.04
−0.02
0.00
0.02
0.04
Possible a values

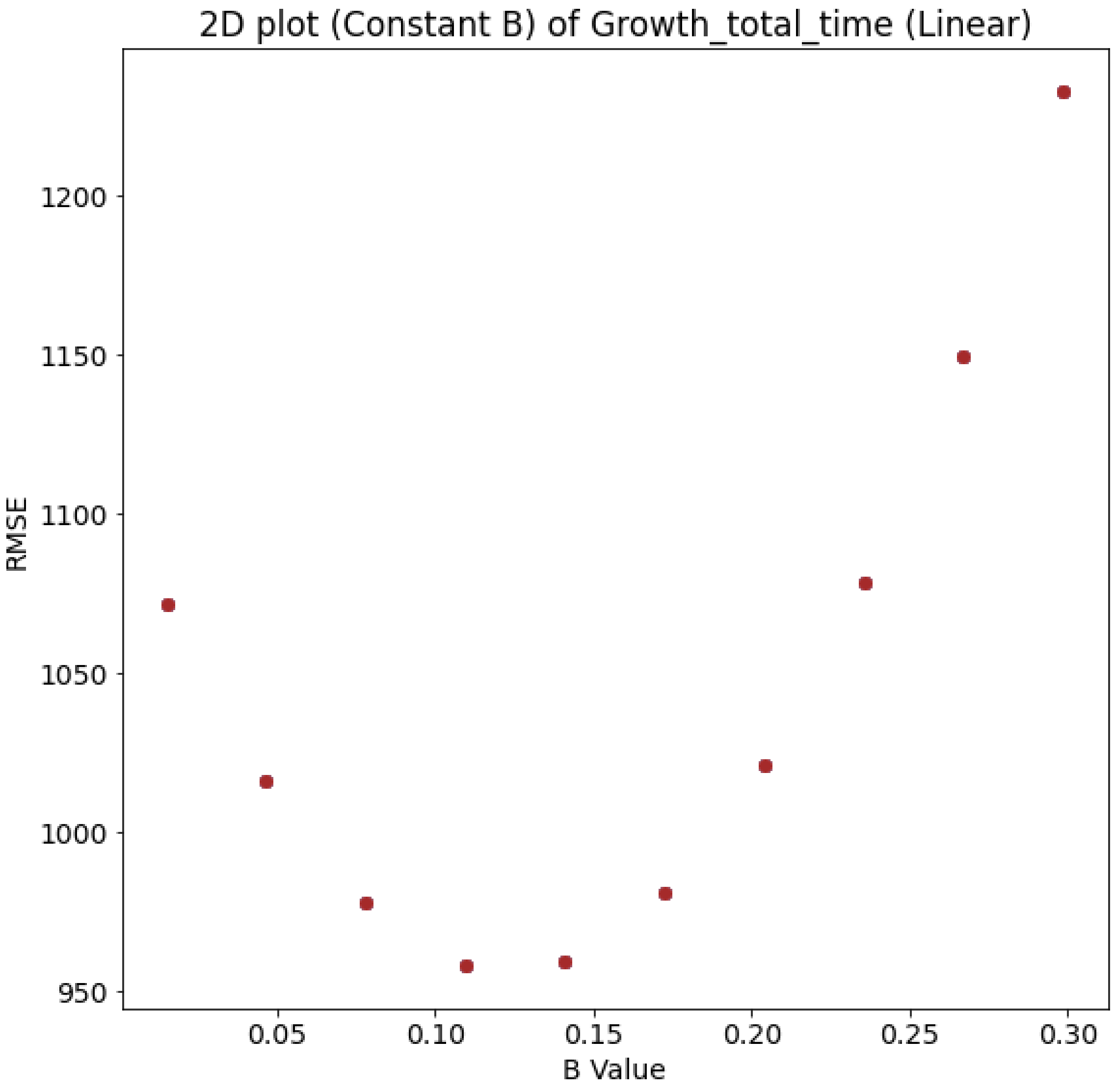
2D plot (Constant B) of Growth_total_time (Linear)
RMSE
B Value
1200
1150
1100
1050
1000
950
0.05
0.10
0.15
0.20
0.25
0.30

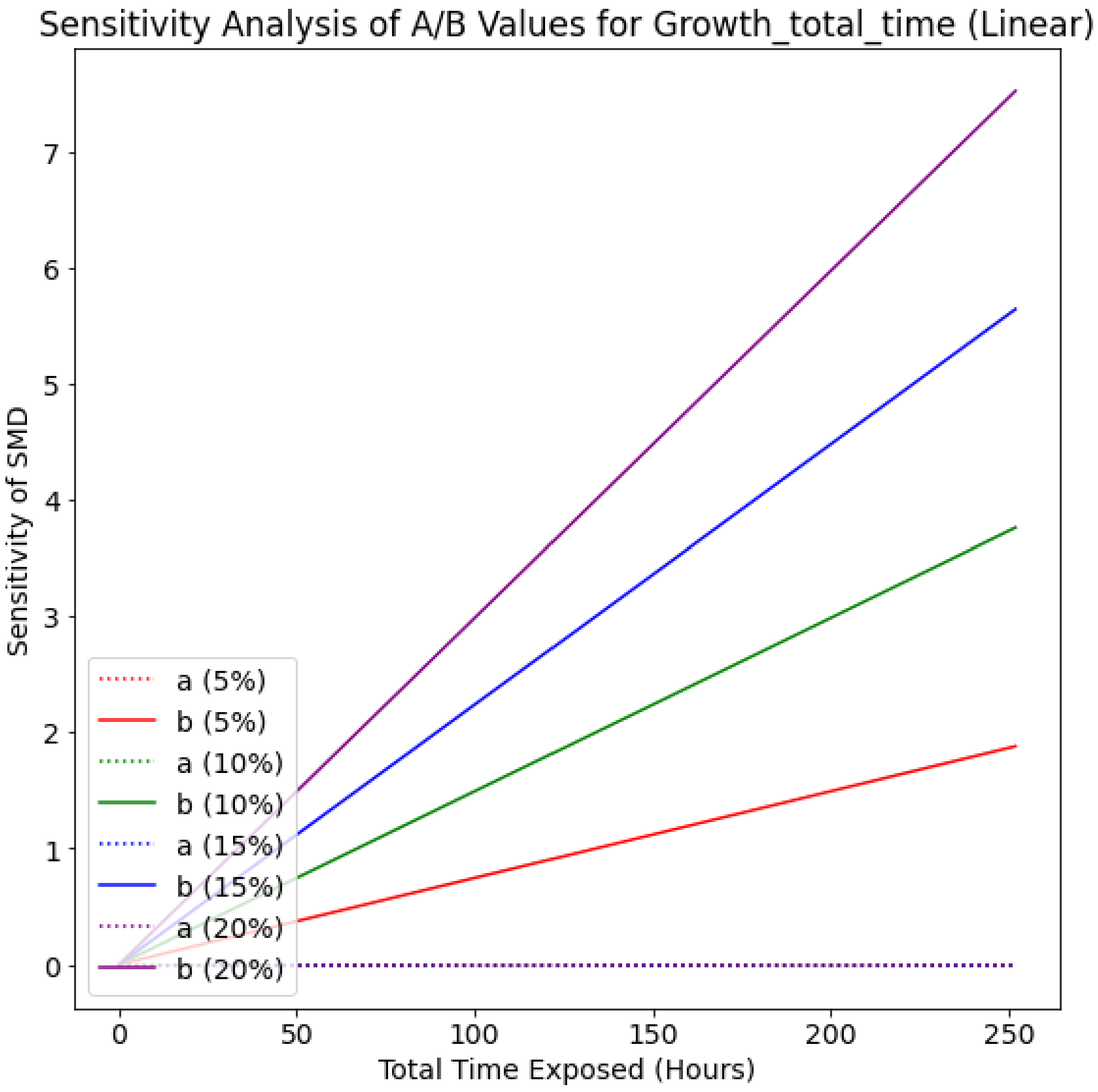
Sensitivity Analysis of A/B Values for Growth_total_time (Linear)
Sensitivity of SMD
Total Time Exposed (Hours)
0
1
2
3
4
5
6
7
0
50
100
150
200
250
a (5%)
b (5%)
a (10%)
b (10%)
a (15%)
b (15%)
a (20%)
b (20%)

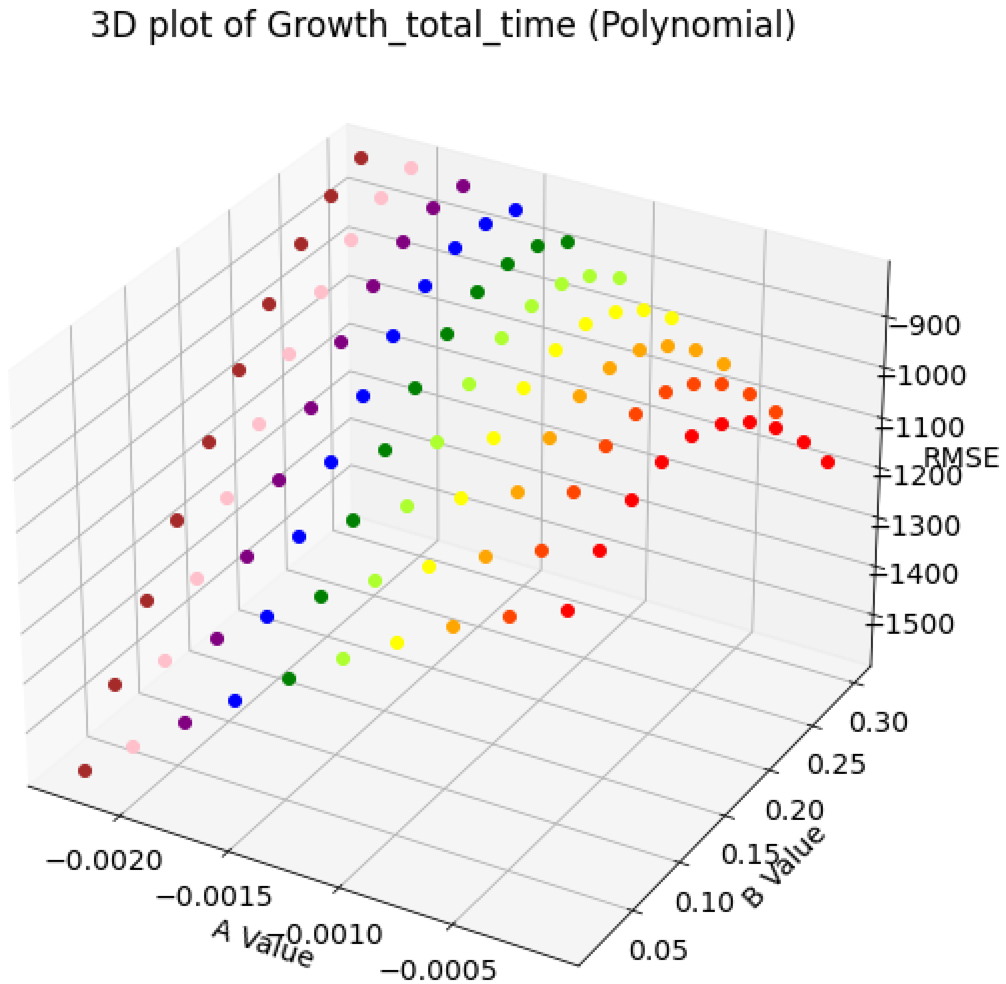
3D plot of Growth_total_time (Polynomial)
−900
−1000
−1100
−1200
−1300
−1400
−1500
RMSE
0.30
0.25
0.20
0.15
0.10
0.05
B Value
−0.0020
−0.0015
−0.0010
−0.0005
A Value

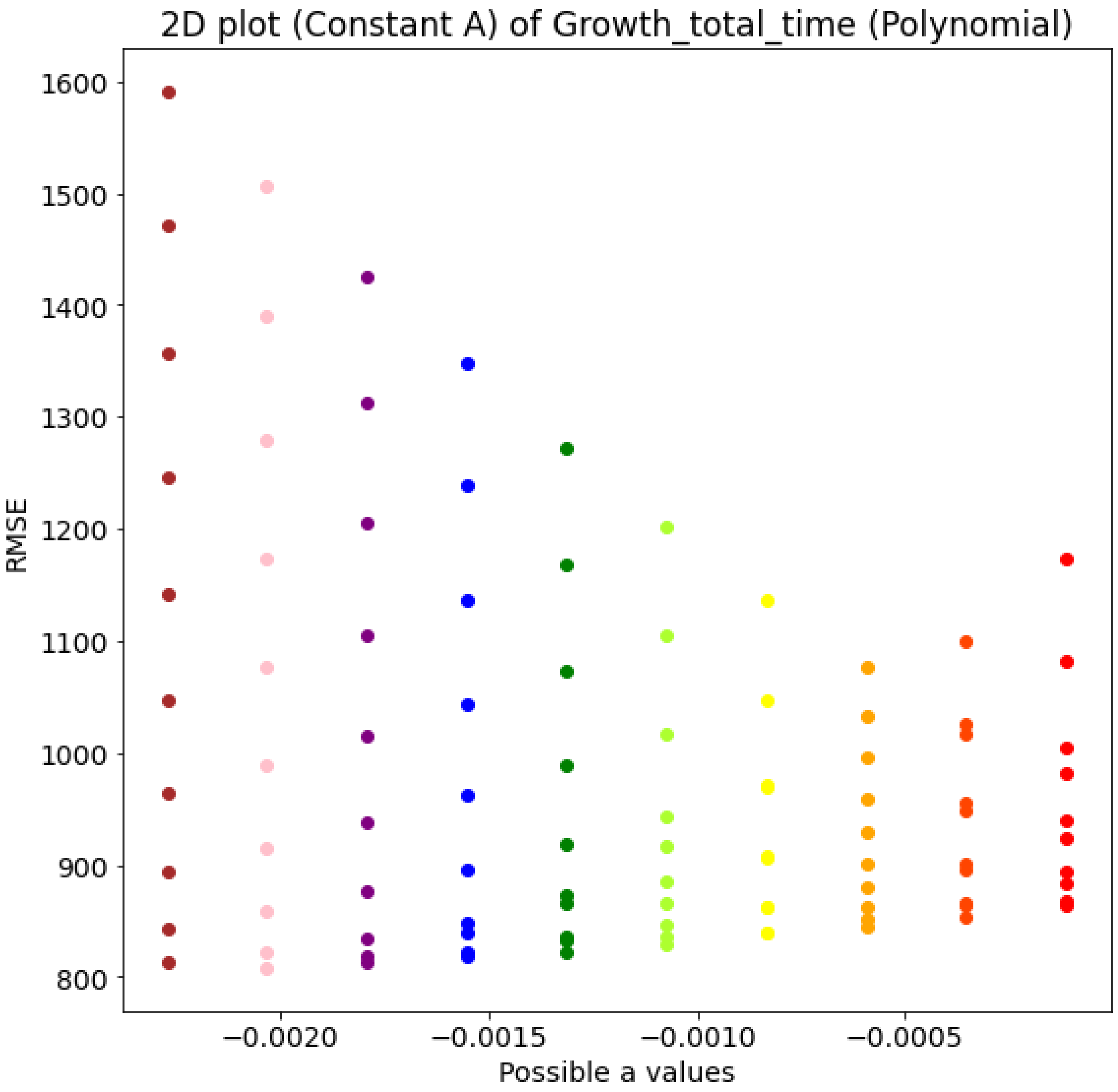
2D plot (Constant A) of Growth_total_time (Polynomial)
RMSE
Possible a values
1600
1500
1400
1300
1200
1100
1000
900
800
−0.0020
−0.0015
−0.0010
−0.0005

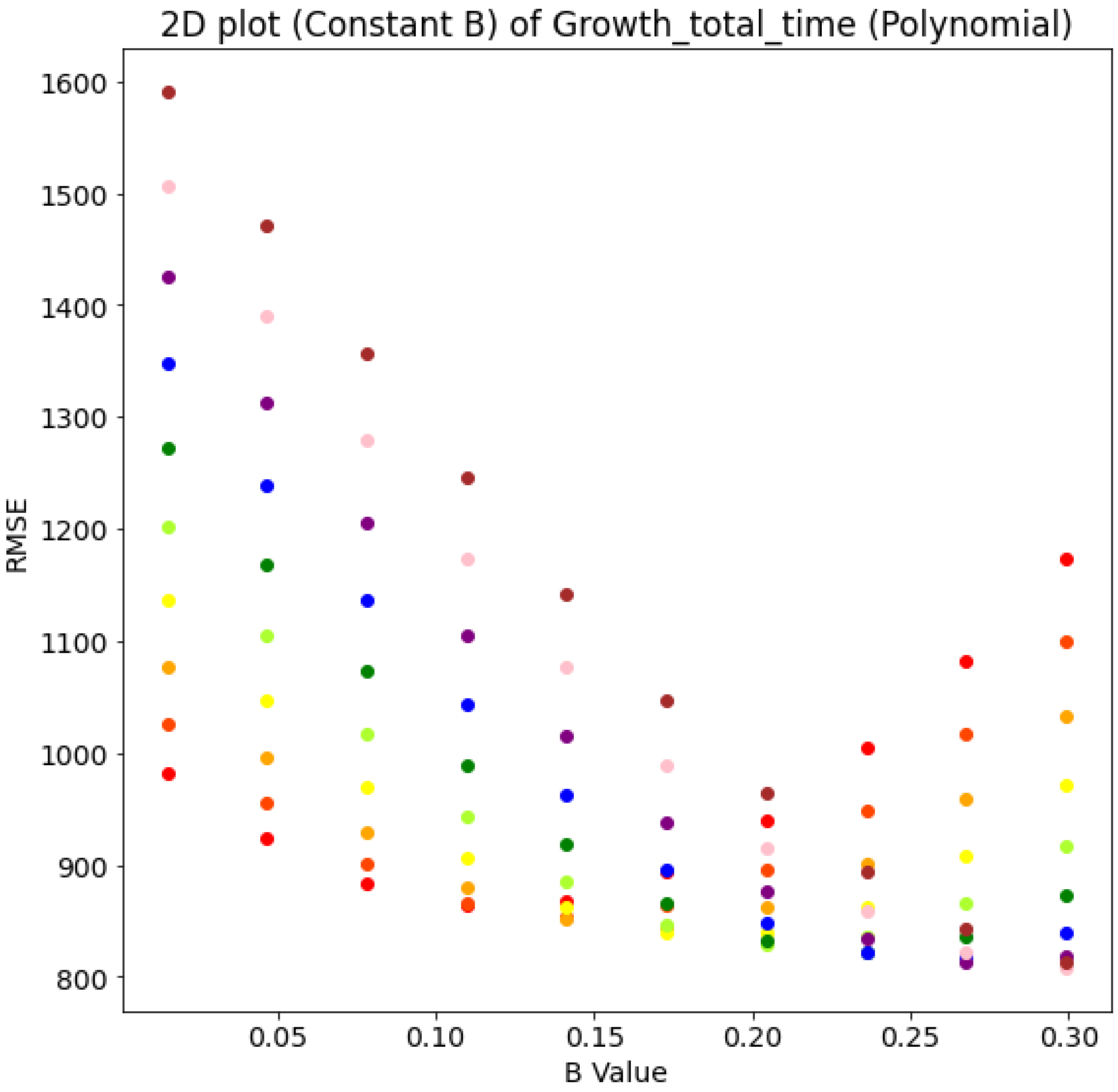
2D plot (Constant B) of Growth_total_time (Polynomial)
RMSE
B Value
1600
1500
1400
1300
1200
1100
1000
900
800
0.05
0.10
0.15
0.20
0.25
0.30

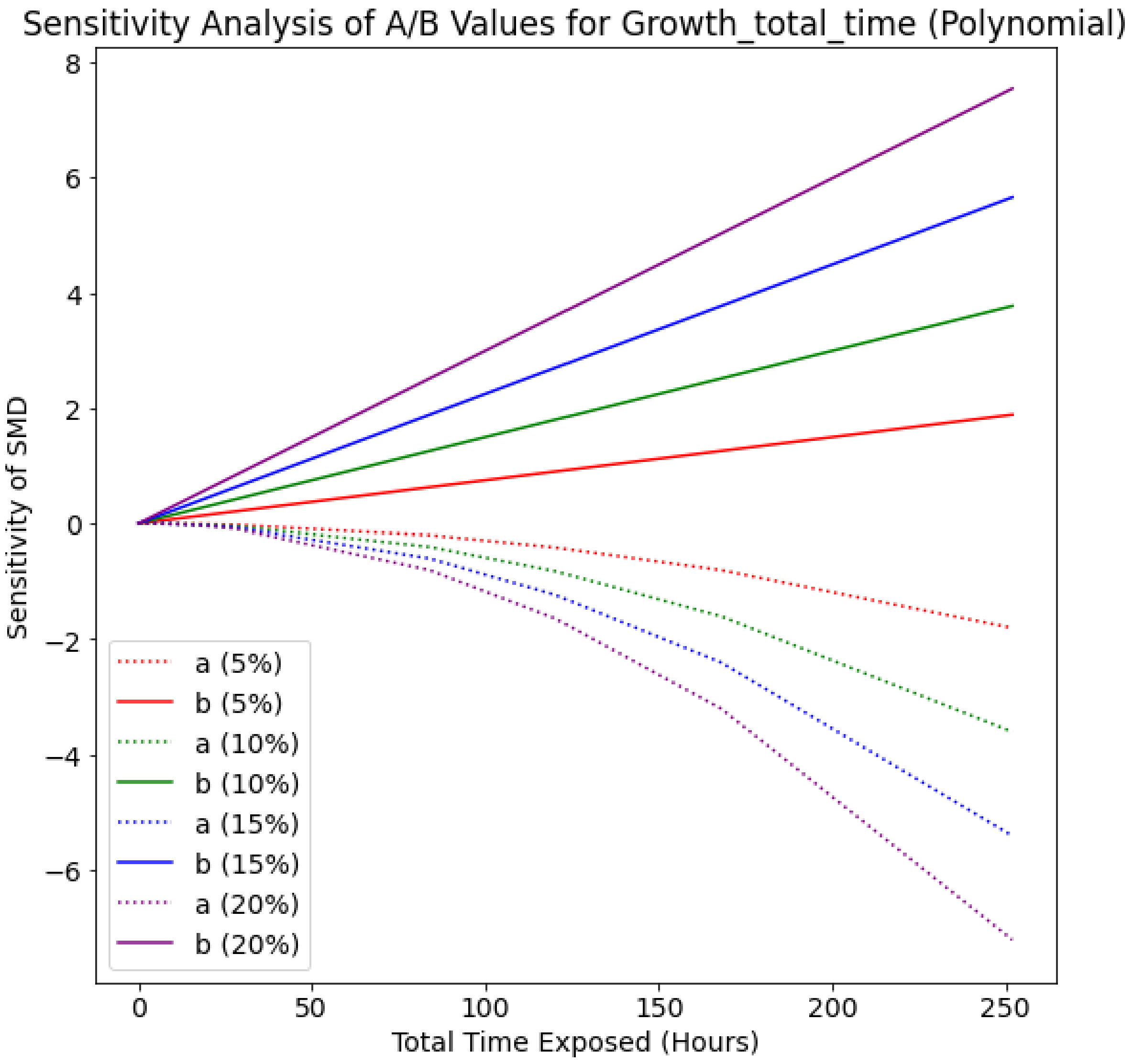
Sensitivity Analysis of A/B Values for Growth_total_time (Polynomial)
Sensitivity of SMD
Total Time Exposed (Hours)
8
6
4
2
0
−2
−4
−6
0
50
100
150
200
250
a (5%)
b (5%)
a (10%)
b (10%)
a (15%)
b (15%)
a (20%)
b (20%)

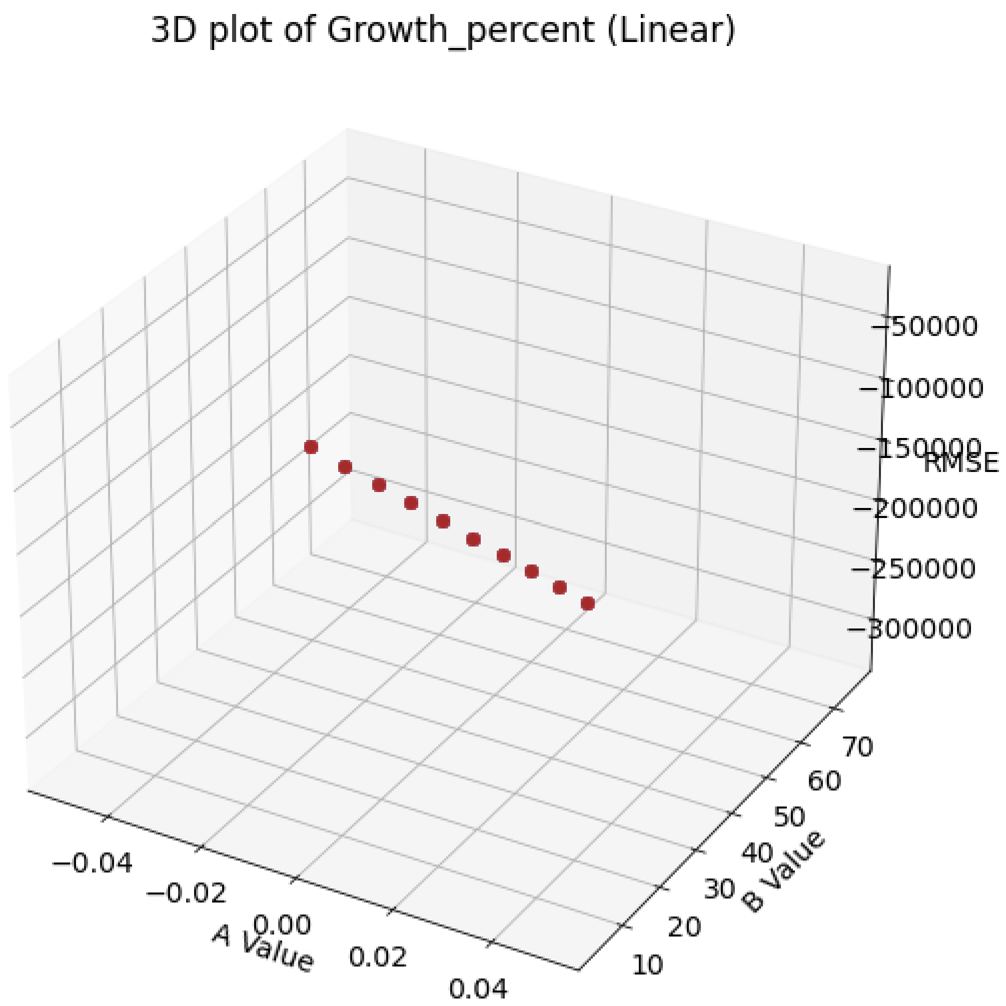
3D plot of Growth_percent (Linear)
−50000
−100000
−150000
RMSE
−200000
−250000
−300000
70
60
50
40
30
20
10
B Value
−0.04
−0.02
0.00
0.02
0.04
A Value

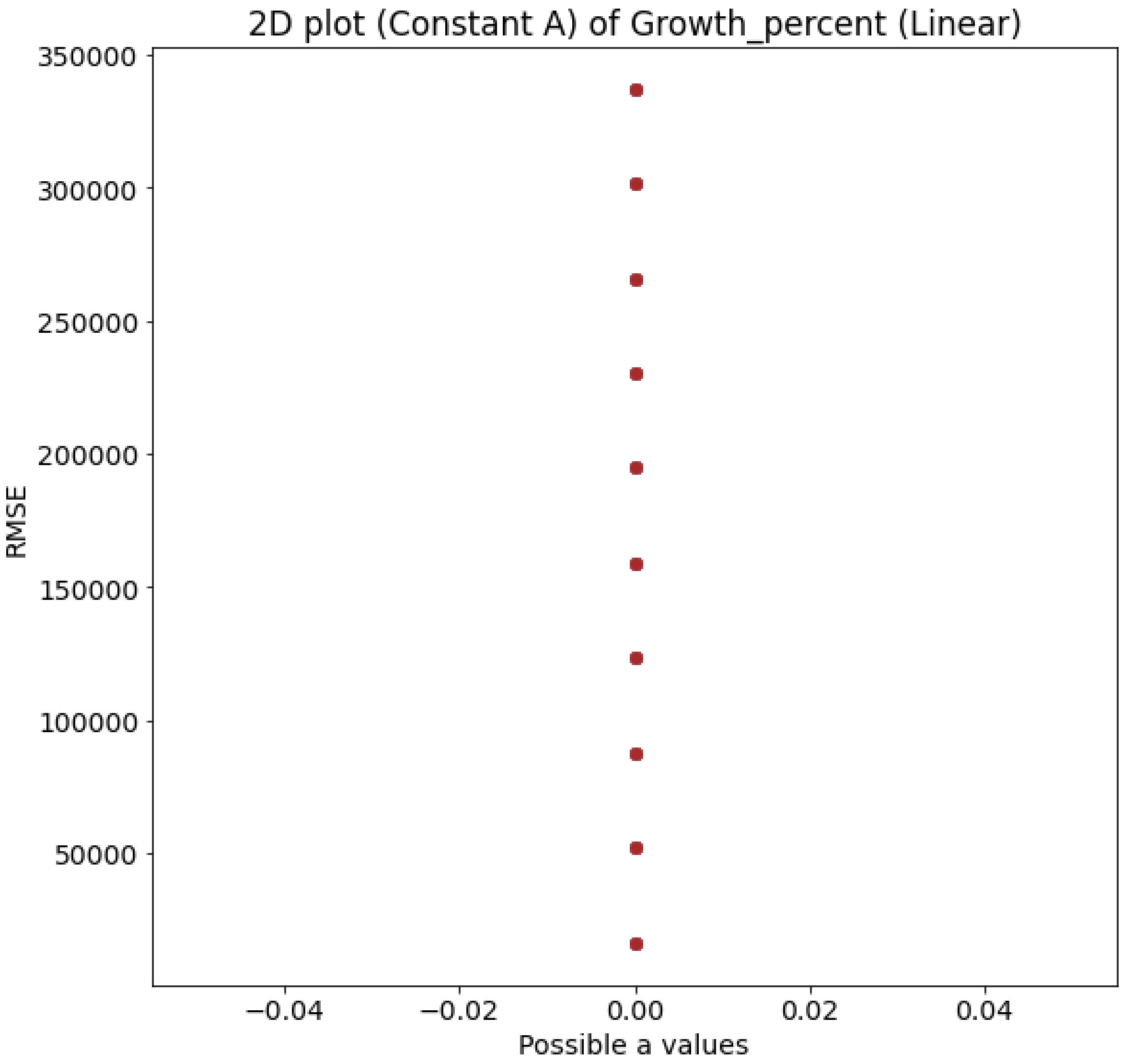
2D plot (Constant A) of Growth_percent (Linear)
RMSE
350000
300000
250000
200000
150000
100000
50000
−0.04
−0.02
0.00
0.02
0.04
Possible a values

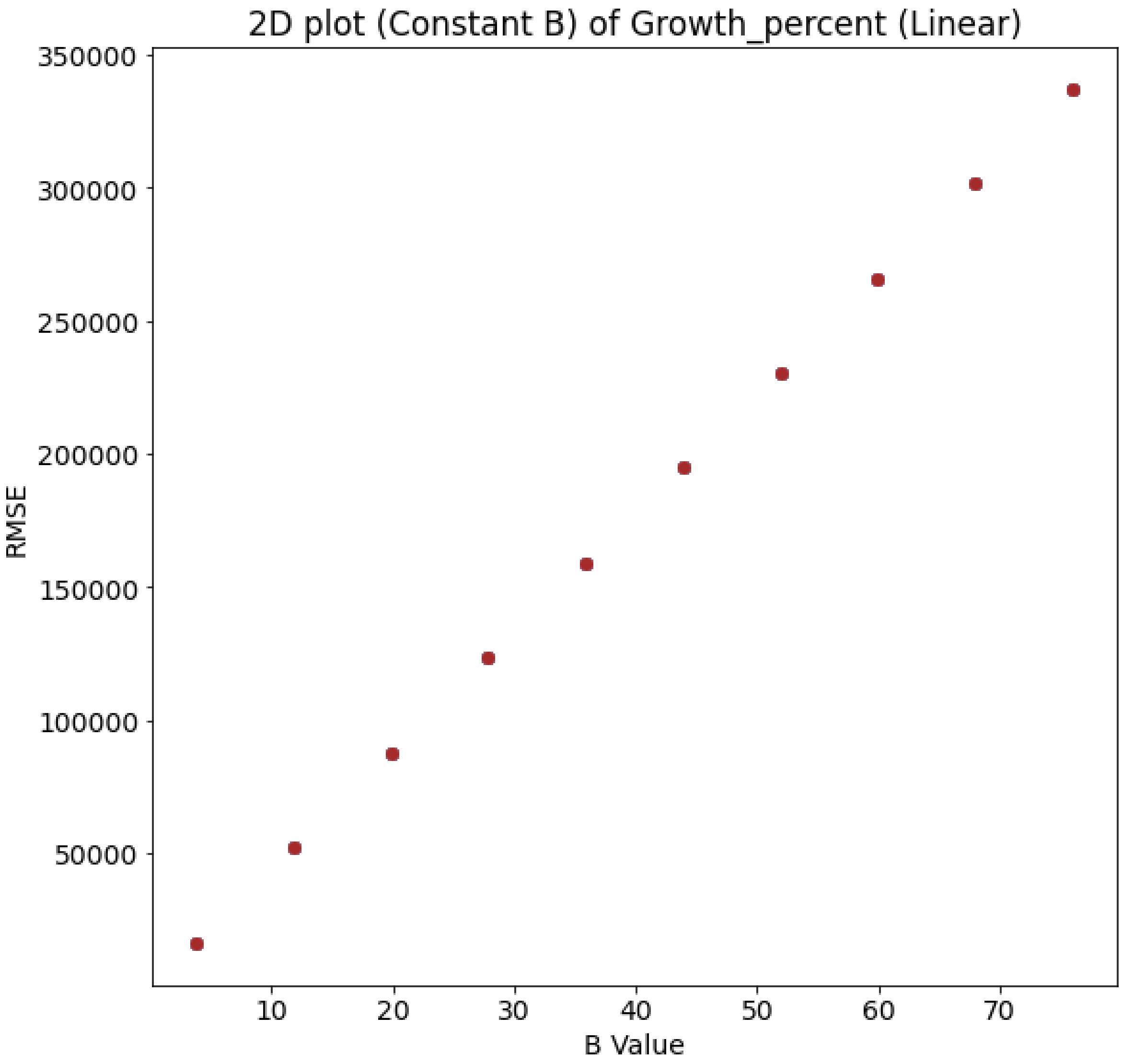
2D plot (Constant B) of Growth_percent (Linear)
RMSE
350000
300000
250000
200000
150000
100000
50000
10
20
30
40
50
60
70
B Value

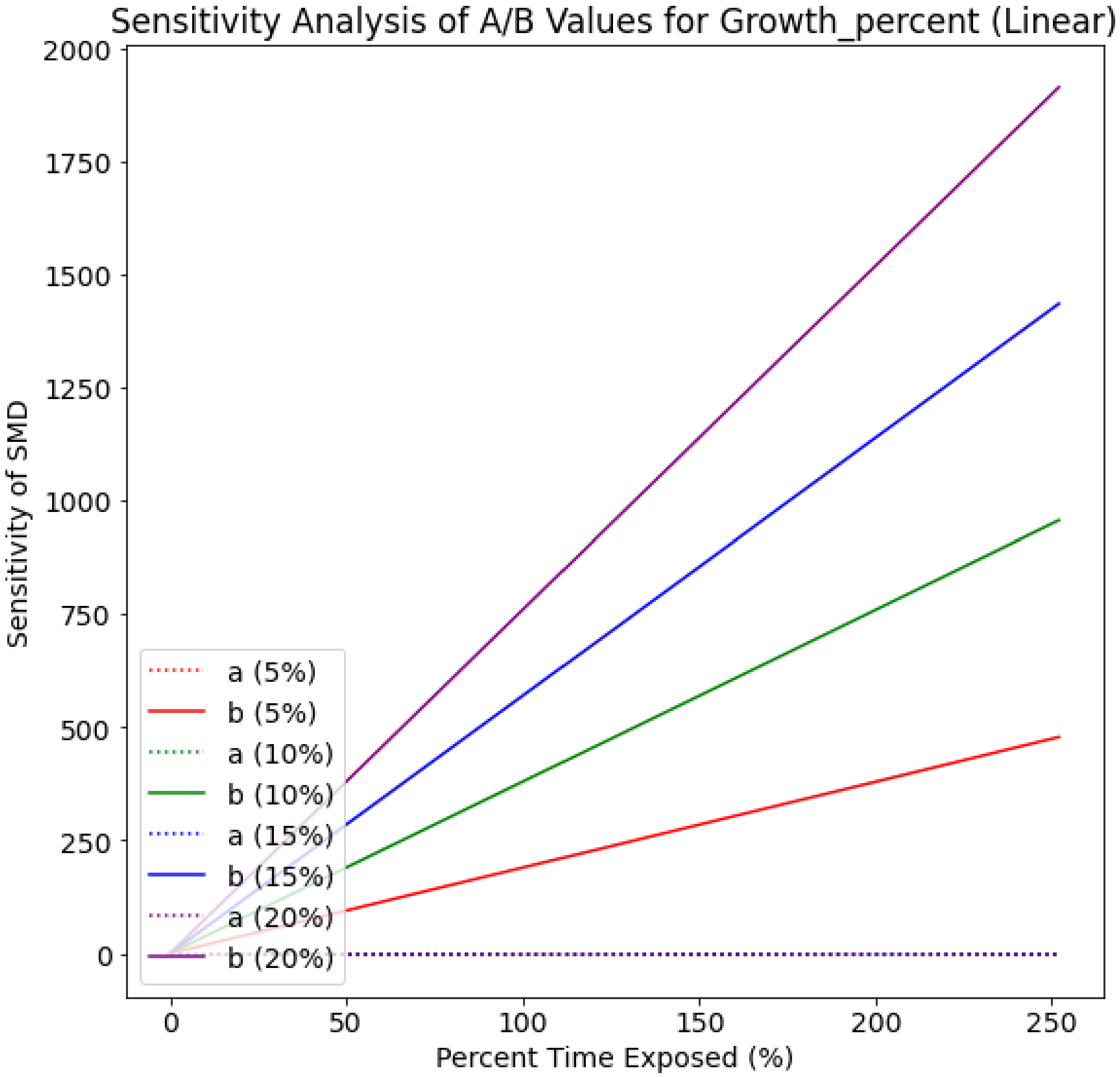
Sensitivity Analysis of A/B Values for Growth_percent (Linear)
Sensitivity of SMD
Percent Time Exposed (%)
2000
1750
1500
1250
1000
750
500
250
0
0
50
100
150
200
250
a (5%)
b (5%)
a (10%)
b (10%)
a (15%)
b (15%)
a (20%)
b (20%)

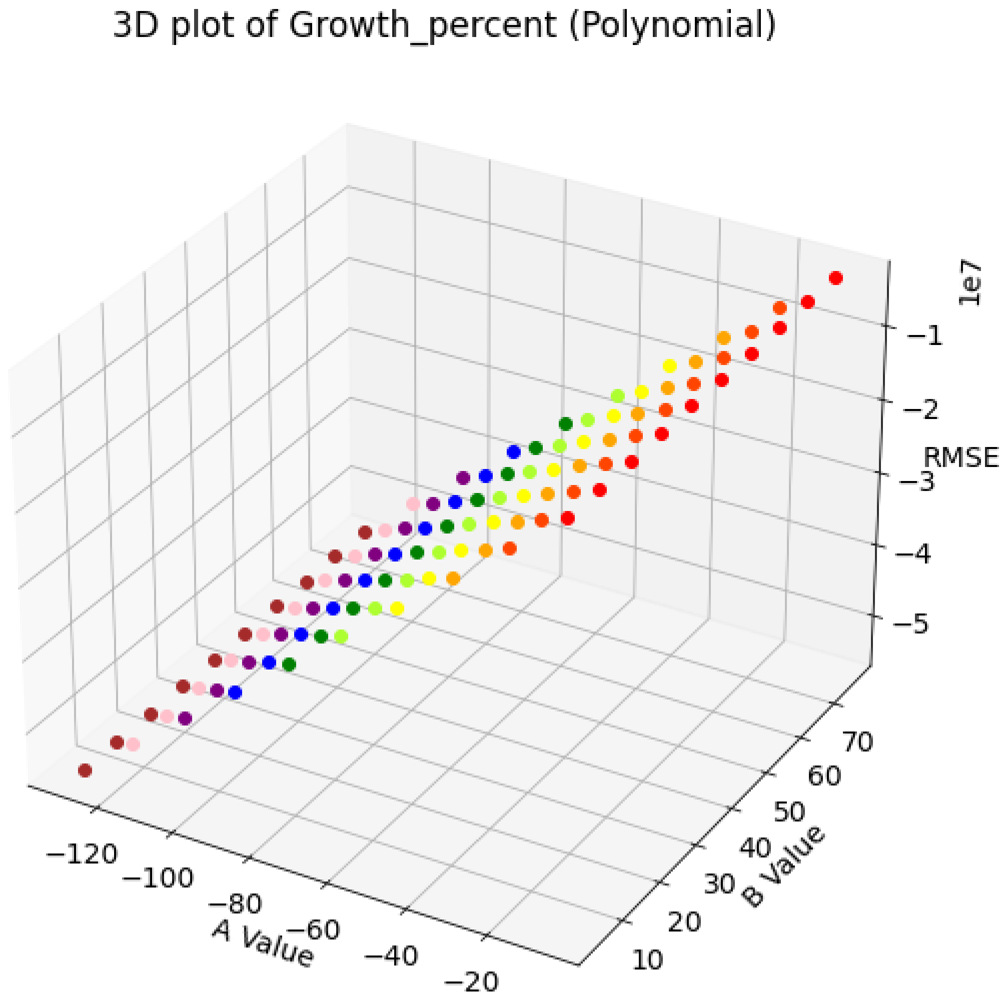
3D plot of Growth_percent (Polynomial)
1e7
−1
−2
RMSE
−3
−4
−5
70
60
50
40
30
B Value
20
10
−120
−100
−80
A Value
−60
−40
−20

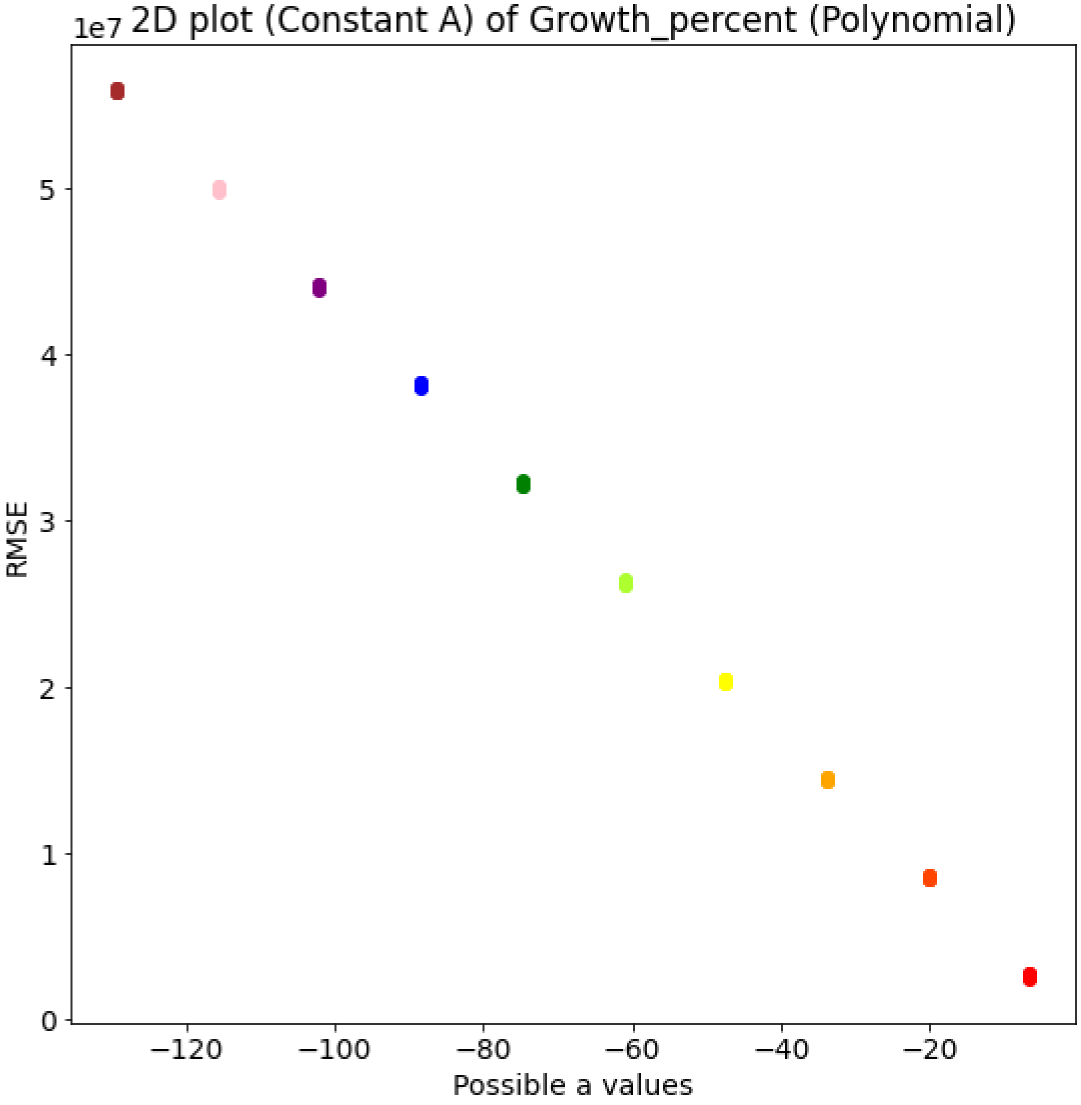

2D plot (Constant A) of Growth_percent (Polynomial)
1e7
RMSE
0
1
2
3
4
5
−120
−100
−80
−60
−40
−20
Possible a values

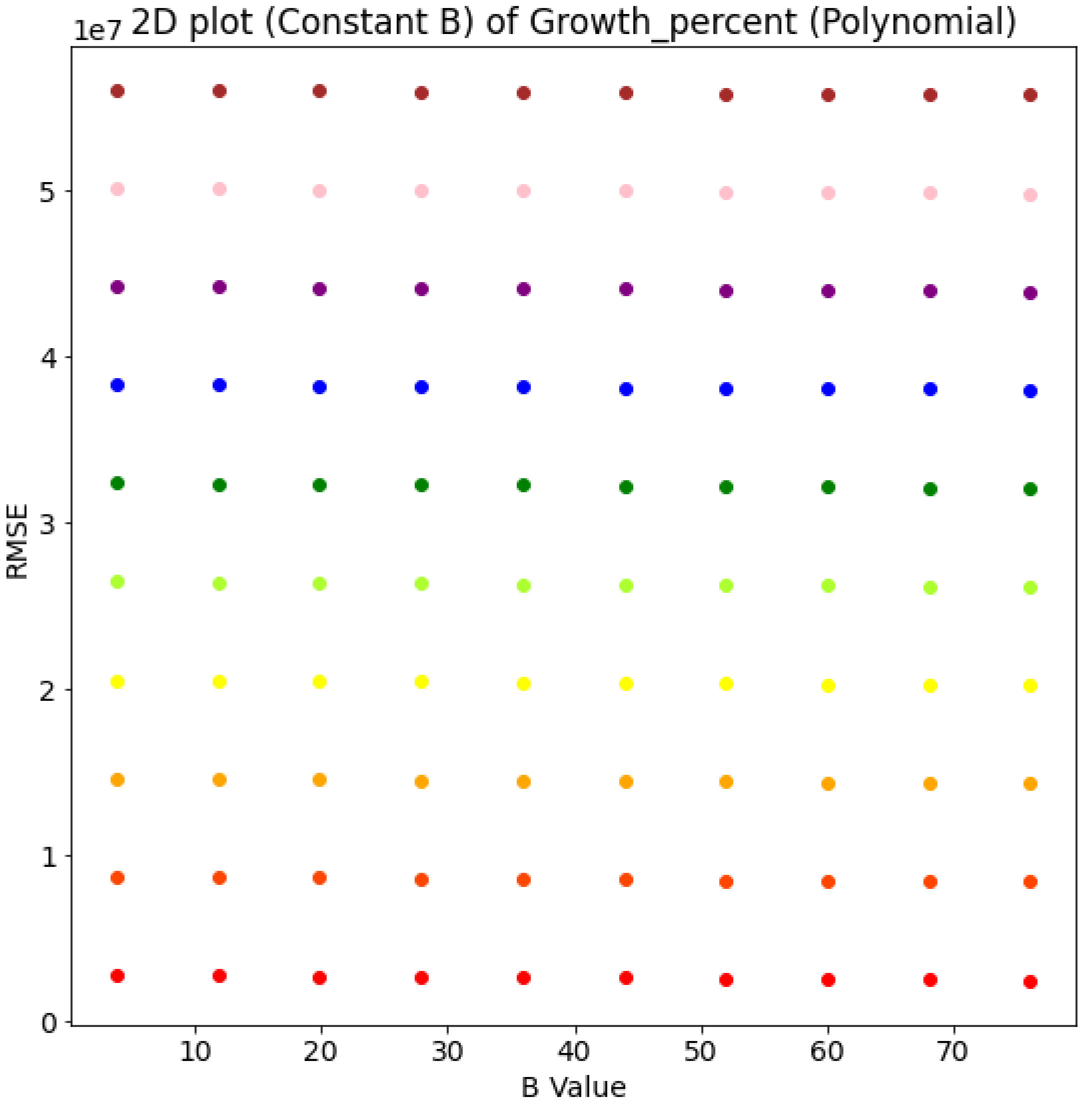
2D plot (Constant B) of Growth_percent (Polynomial)
1e7
RMSE
B Value
0
1
2
3
4
5
10
20
30
40
50
60
70

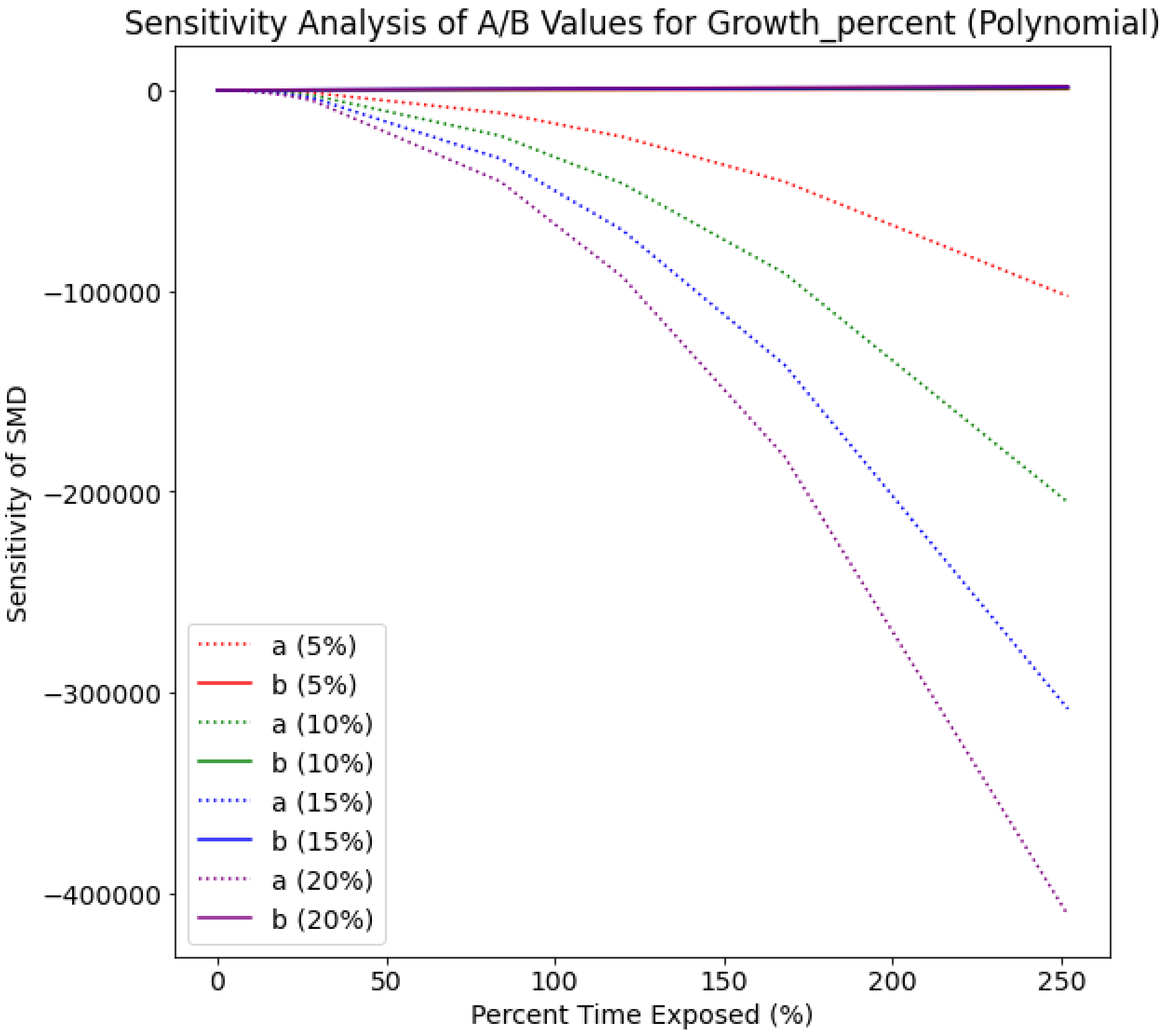
Sensitivity Analysis of A/B Values for Growth_percent (Polynomial)
Sensitivity of SMD
0
−100000
−200000
−300000
−400000
0
50
100
150
200
250
Percent Time Exposed (%)
a (5%)
b (5%)
a (10%)
b (10%)
a (15%)
b (15%)
a (20%)
b (20%)

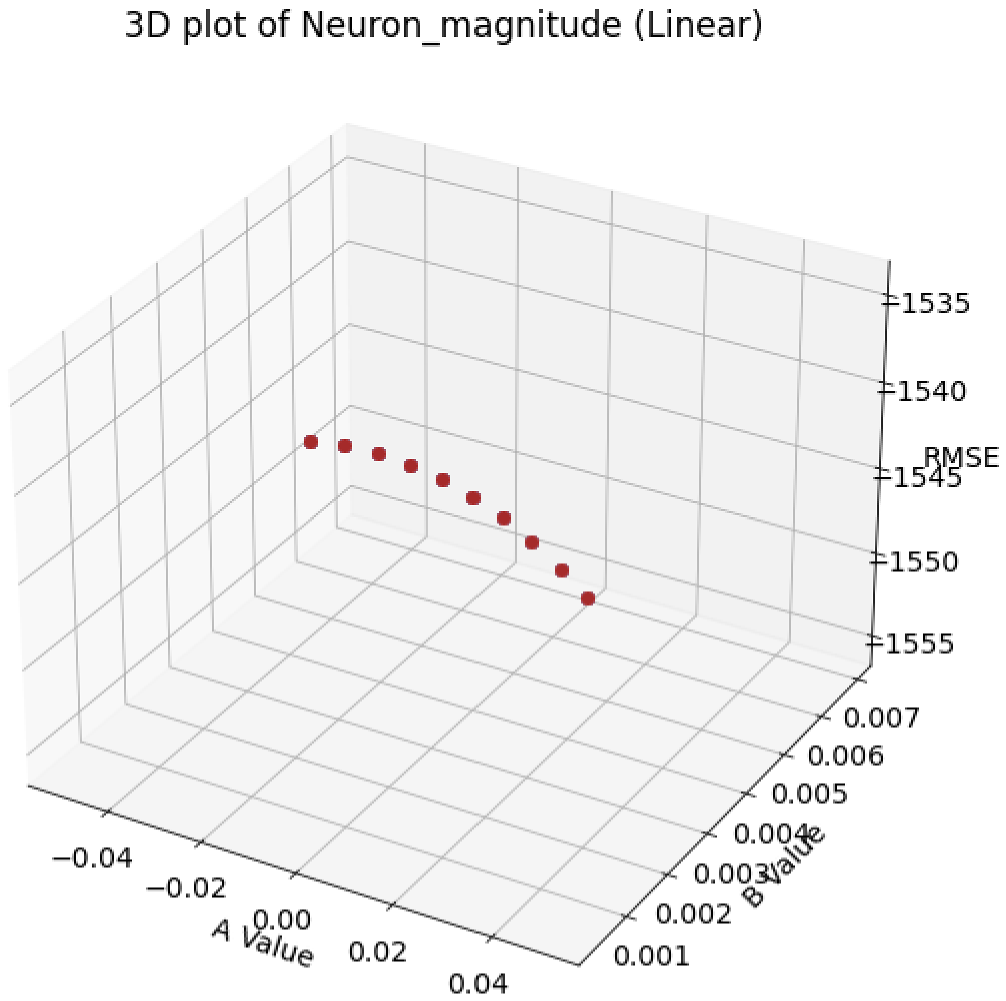
3D plot of Neuron_magnitude (Linear)
RMSE
1535
1540
1545
1550
1555
A Value
−0.04
−0.02
0.00
0.02
0.04
B Value
0.001
0.002
0.003
0.004
0.005
0.006
0.007

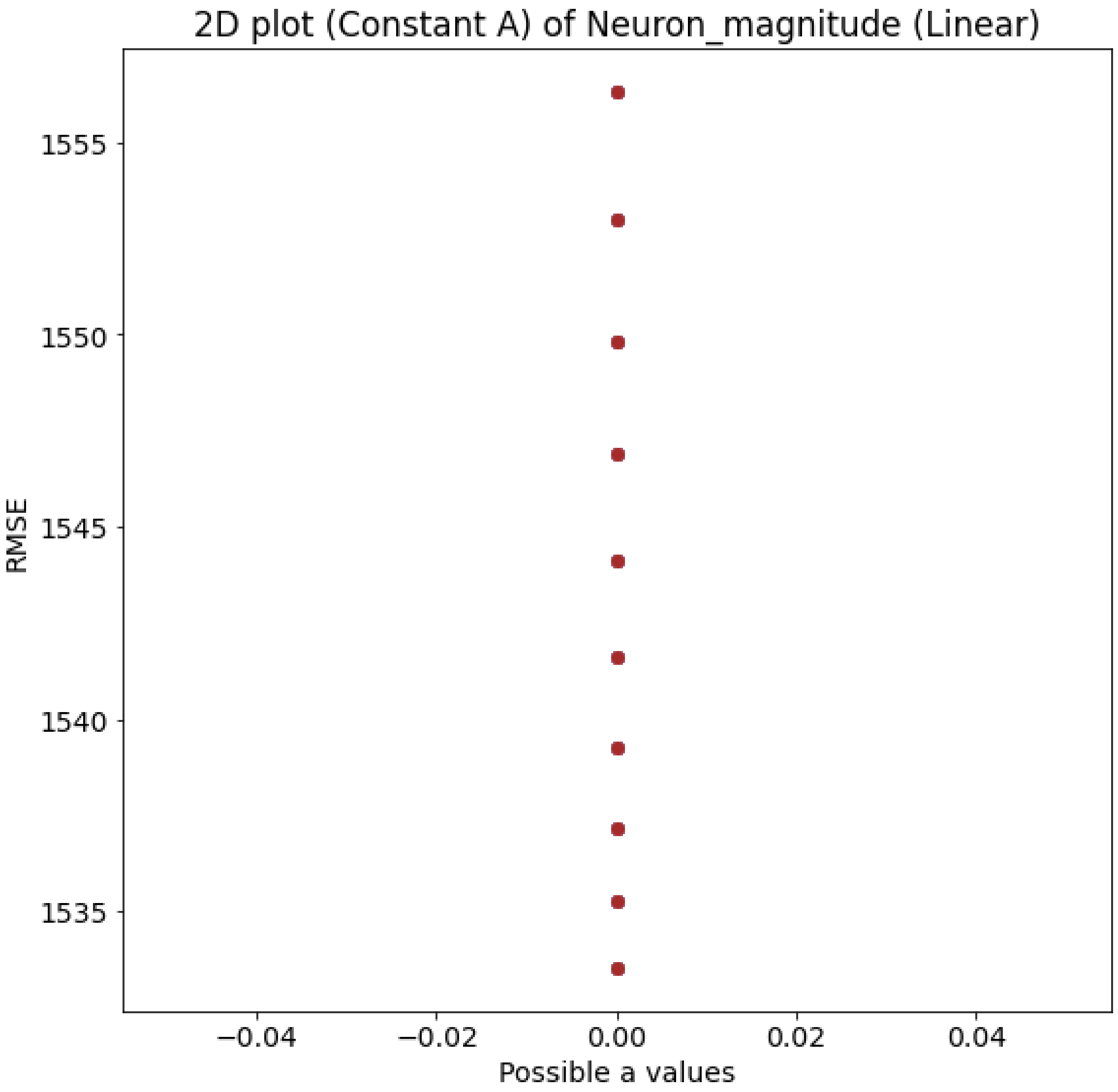
2D plot (Constant A) of Neuron_magnitude (Linear)
RMSE
1555
1550
1545
1540
1535
−0.04
−0.02
0.00
0.02
0.04
Possible a values

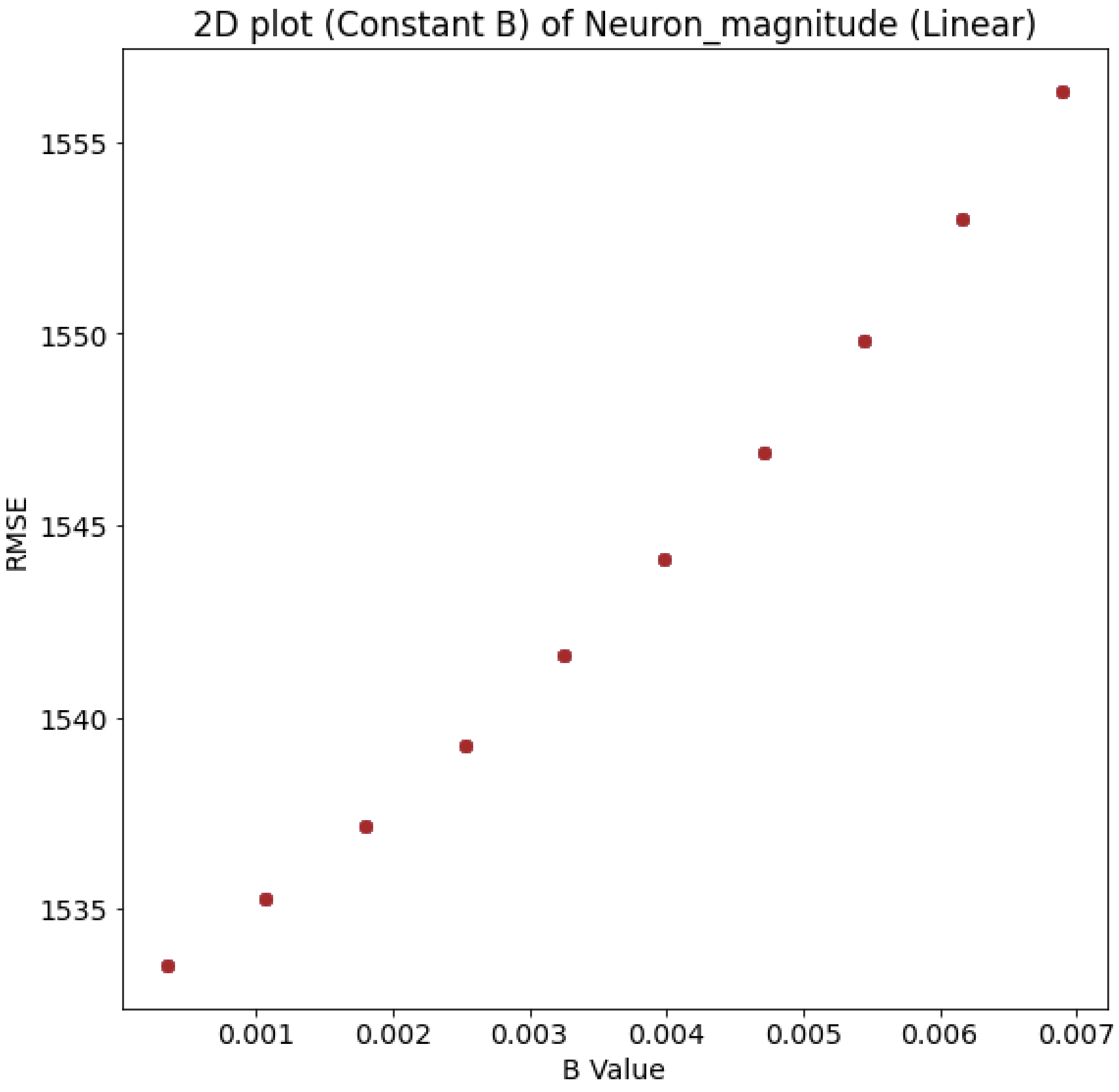
2D plot (Constant B) of Neuron_magnitude (Linear)
RMSE
1535
1540
1545
1550
1555
0.001
0.002
0.003
0.004
0.005
0.006
0.007
B Value

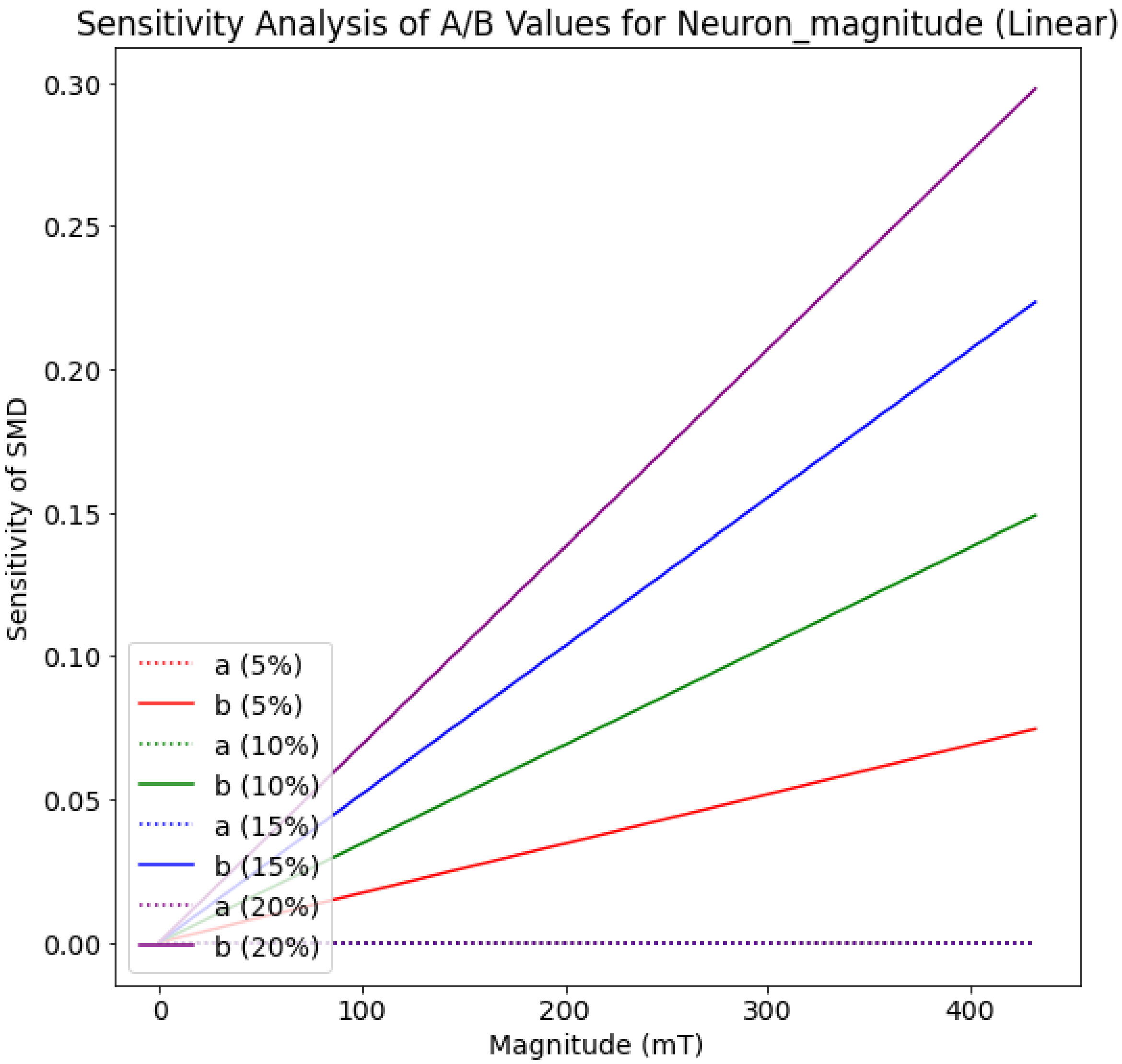
Sensitivity Analysis of A/B Values for Neuron_magnitude (Linear)
Sensitivity of SMD
Magnitude (mT)
0.00
0.05
0.10
0.15
0.20
0.25
0.30
0
100
200
300
400
a (5%)
b (5%)
a (10%)
b (10%)
a (15%)
b (15%)
a (20%)
b (20%)

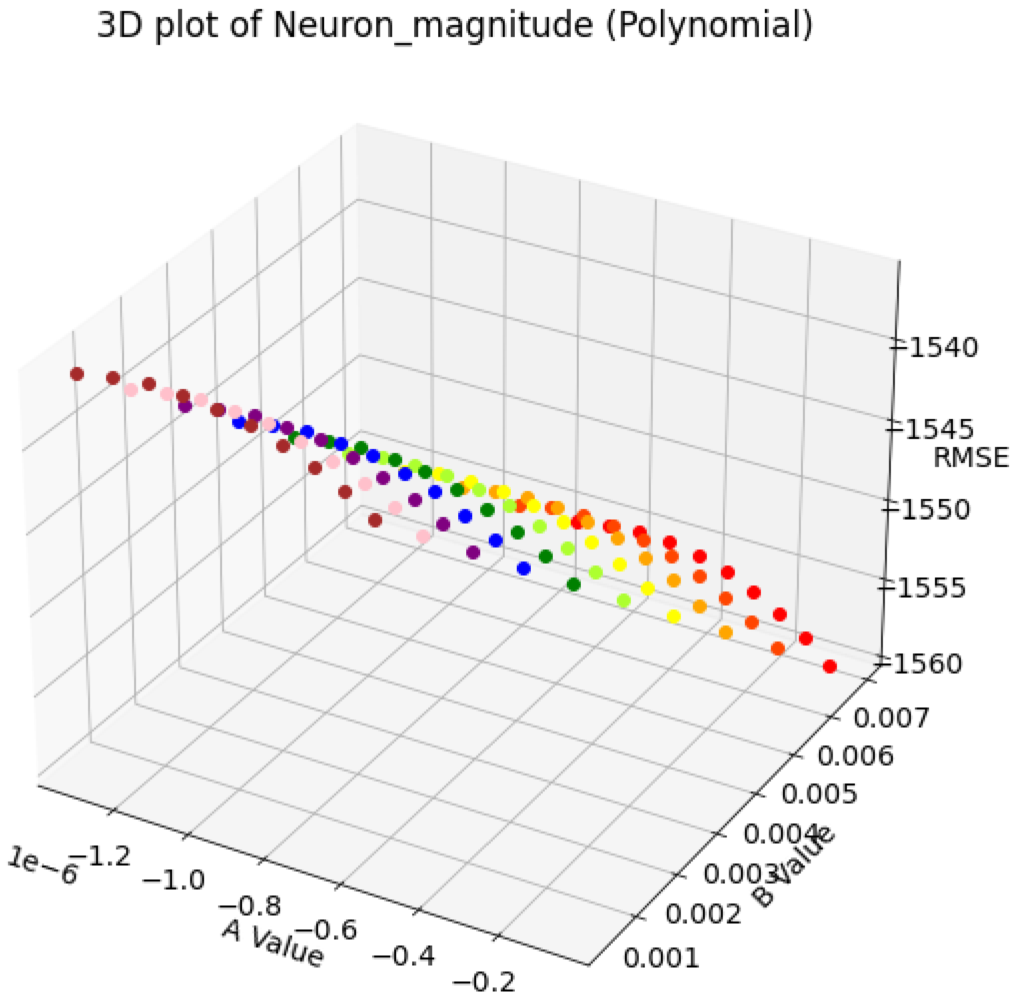
3D plot of Neuron_magnitude (Polynomial)
1540
1545
1550
1555
1560
RMSE
0.007
0.006
0.005
0.004
0.003
0.002
0.001
B Value
1e−6
−1.2
−1.0
−0.8
−0.6
−0.4
−0.2
A Value

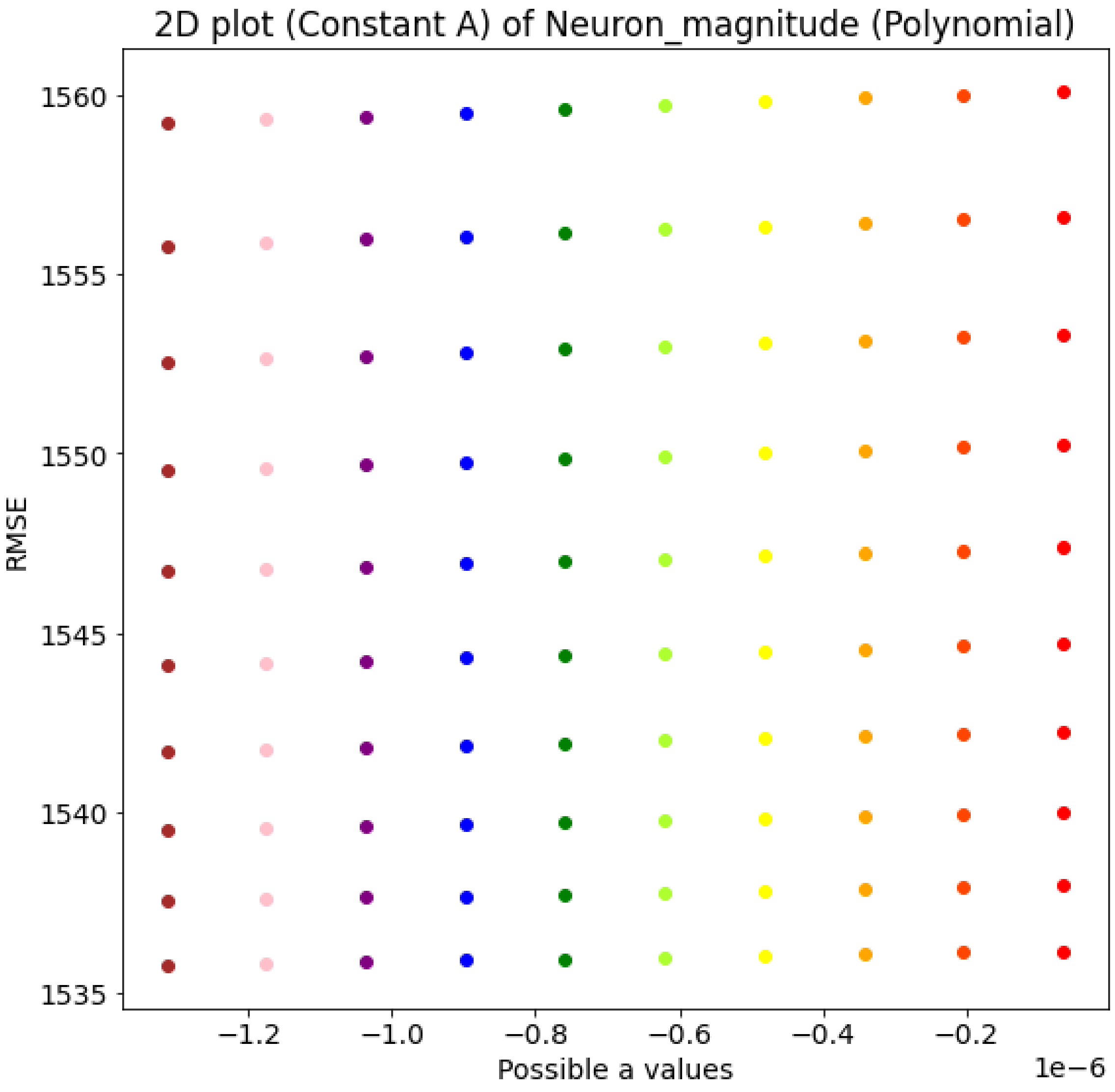
2D plot (Constant A) of Neuron_magnitude (Polynomial)
RMSE
1535
1540
1545
1550
1555
1560
−1.2
−1.0
−0.8
−0.6
−0.4
−0.2
1e−6
Possible a values

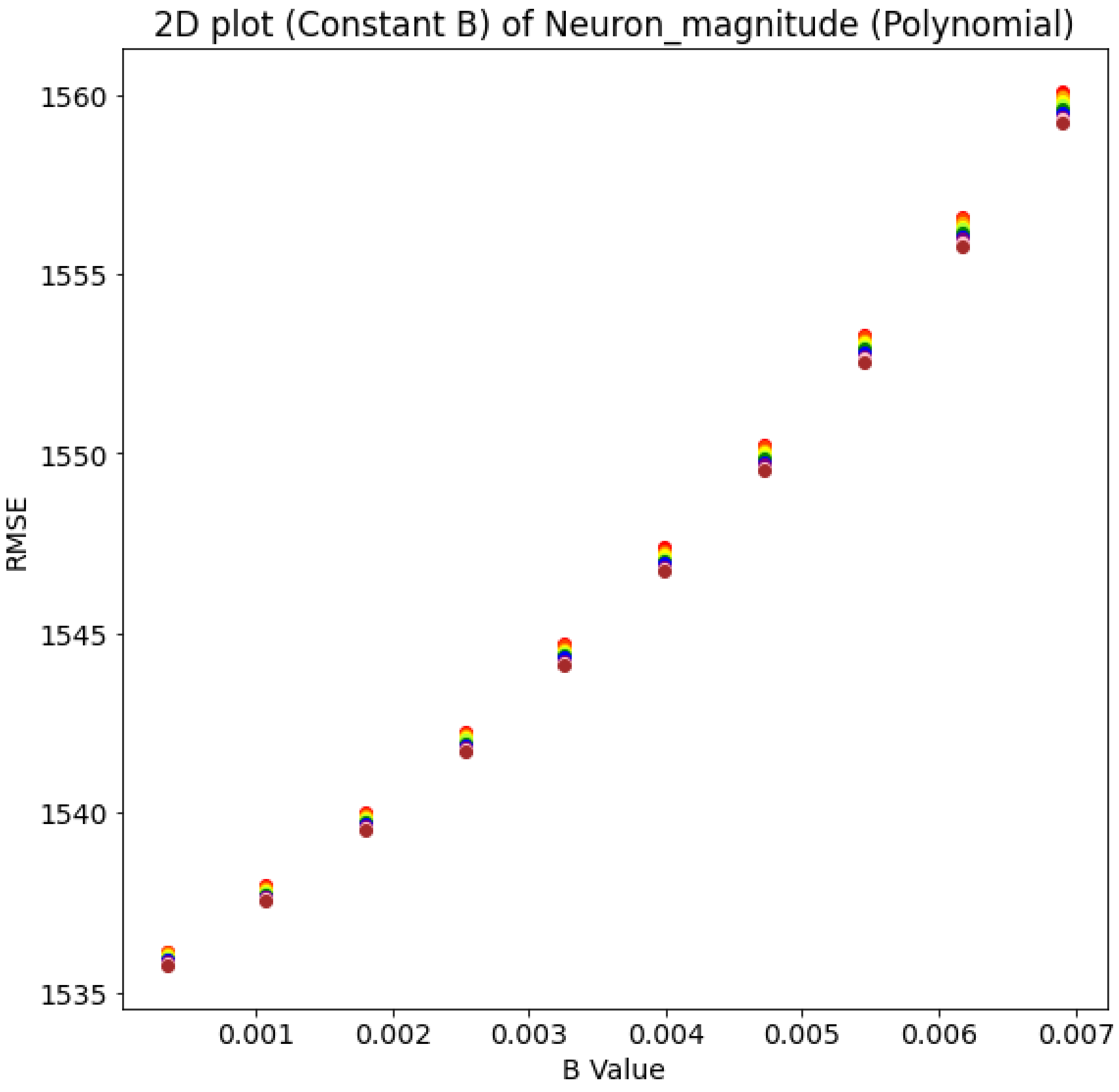
2D plot (Constant B) of Neuron_magnitude (Polynomial)
1560
1555
1550
1545
1540
1535
RMSE
0.001
0.002
0.003
0.004
0.005
0.006
0.007
B Value

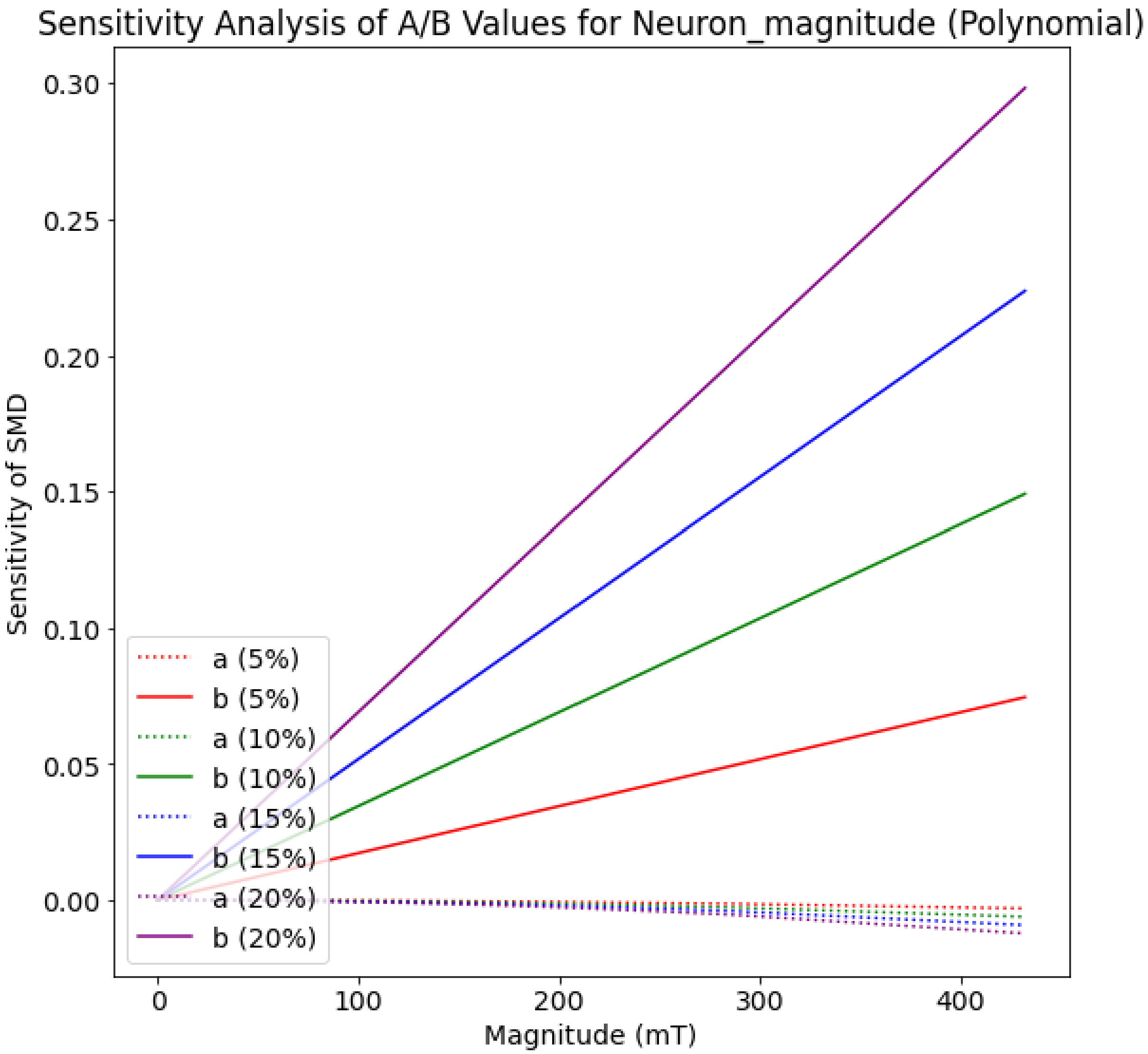
Sensitivity Analysis of A/B Values for Neuron_magnitude (Polynomial)
Sensitivity of SMD
Magnitude (mT)
0.30
0.25
0.20
0.15
0.10
0.05
0.00
0
100
200
300
400
a (5%)
b (5%)
a (10%)
b (10%)
a (15%)
b (15%)
a (20%)
b (20%)

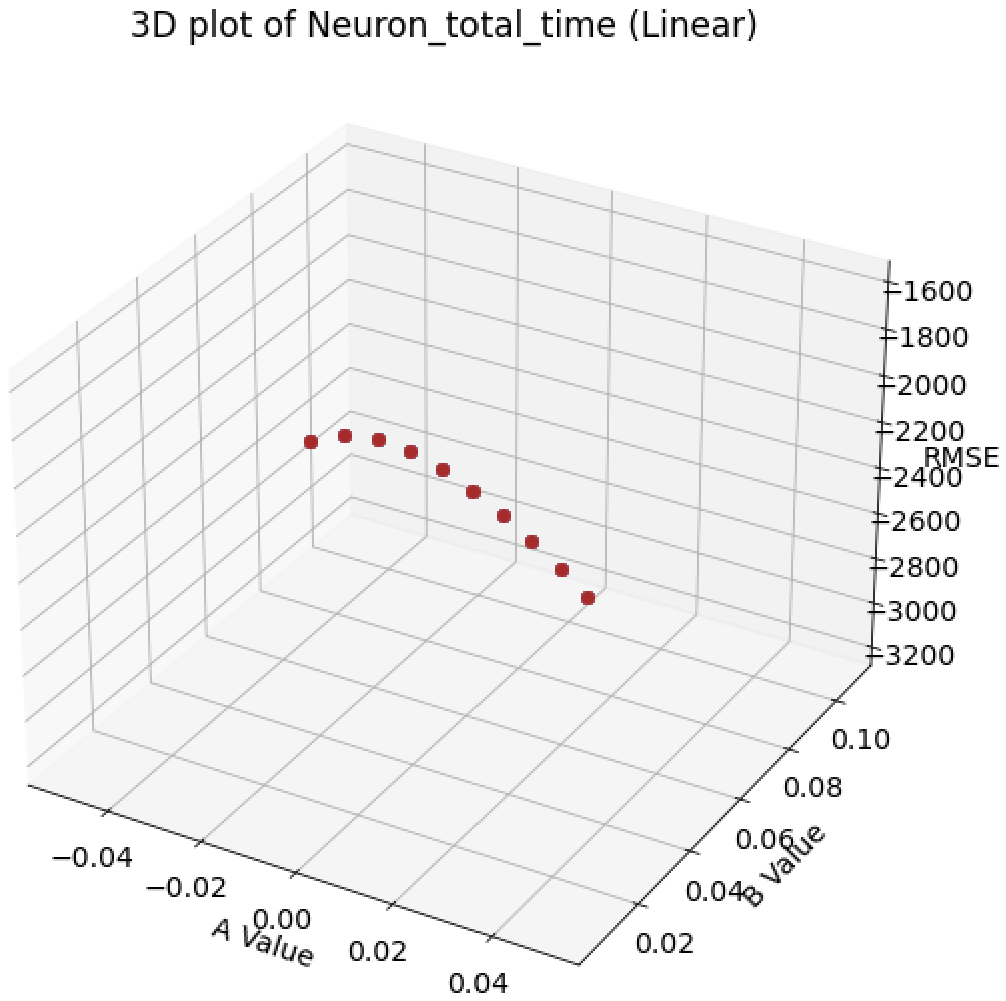
3D plot of Neuron_total_time (Linear)
1600
1800
2000
2200
2400
2600
2800
3000
3200
RMSE
0.10
0.08
0.06
0.04
0.02
B Value
−0.04
−0.02
0.00
0.02
0.04
A Value

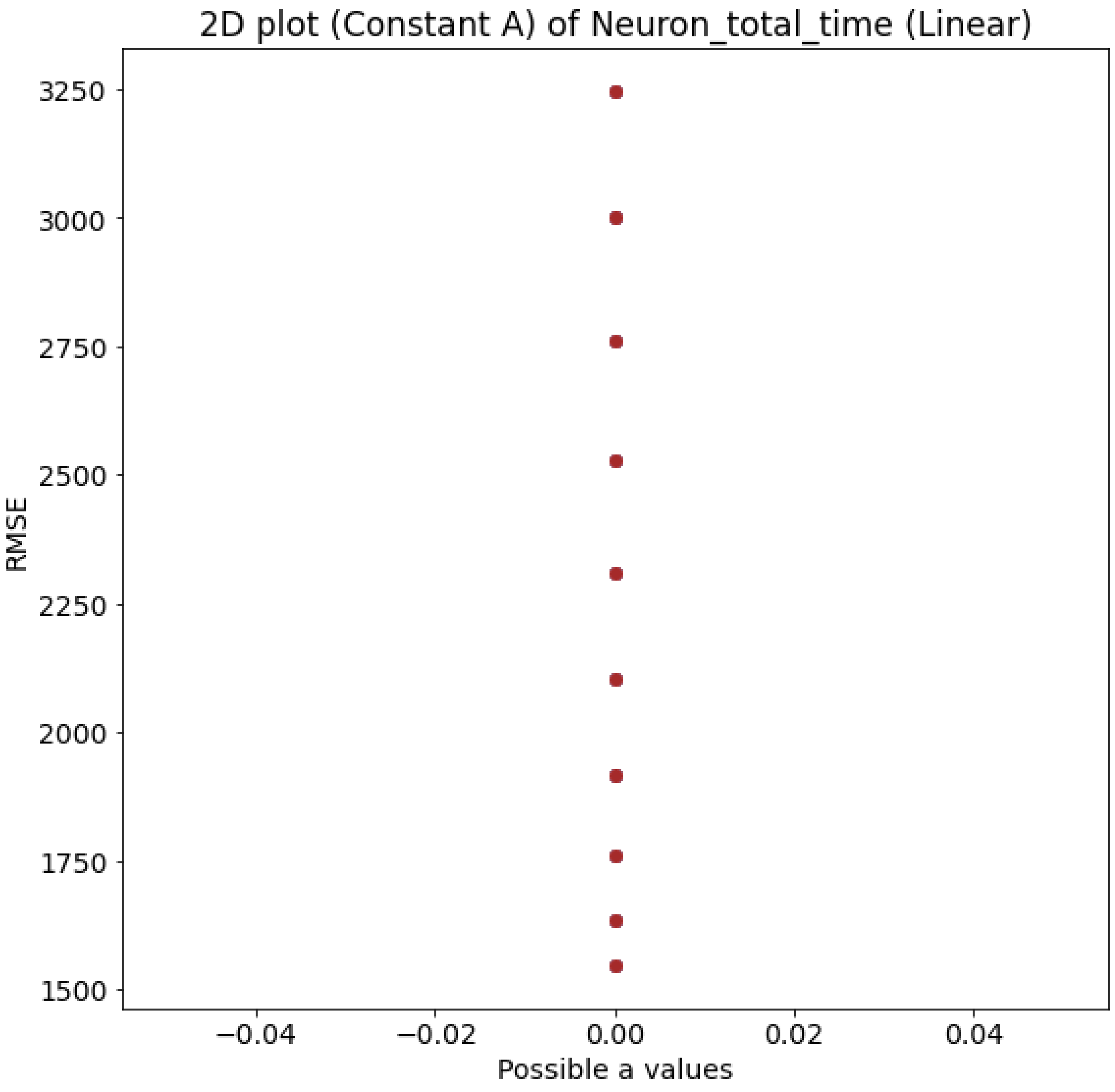
2D plot (Constant A) of Neuron_total_time (Linear)
RMSE
3250
3000
2750
2500
2250
2000
1750
1500
−0.04
−0.02
0.00
0.02
0.04
Possible a values

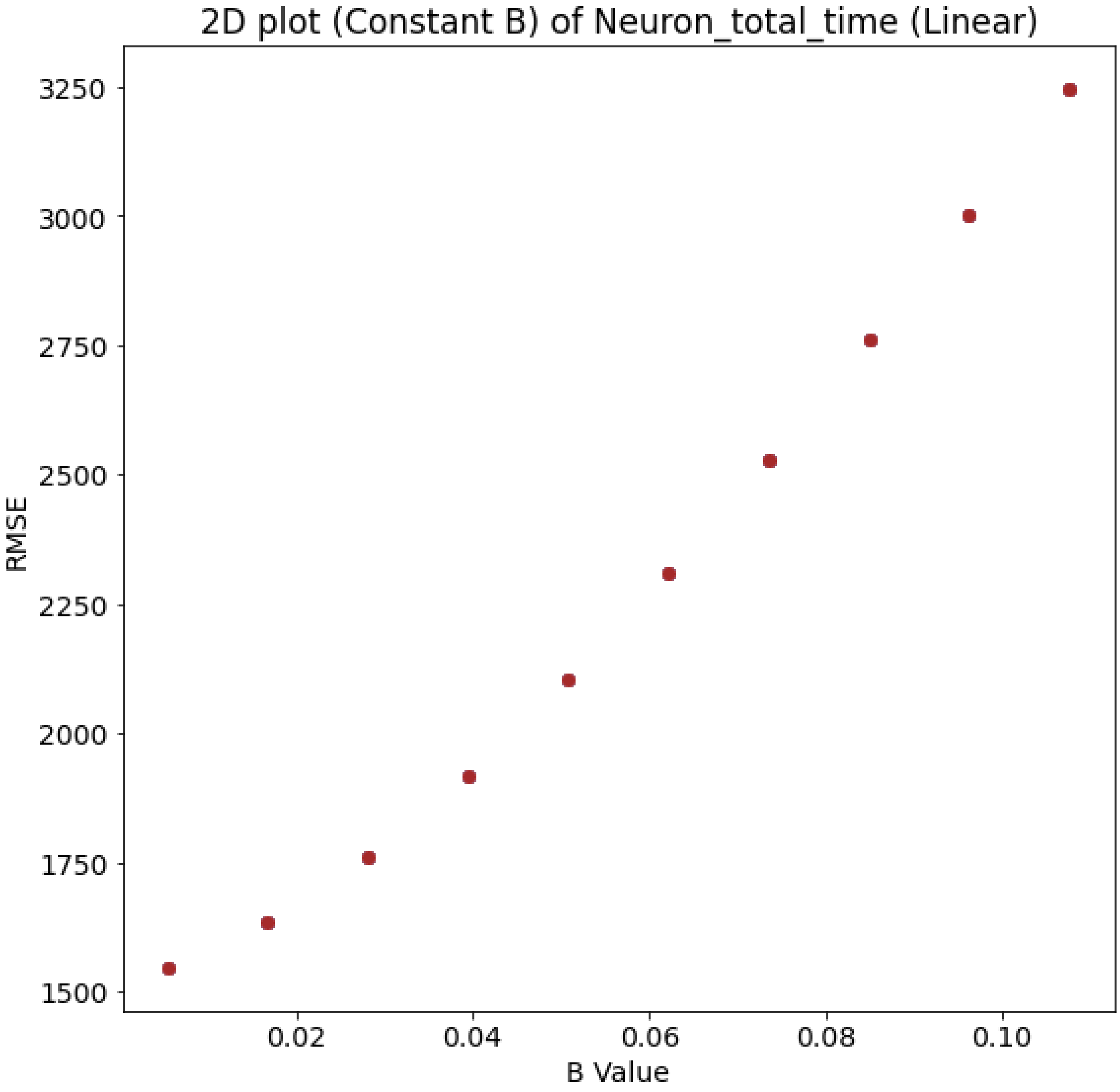
2D plot (Constant B) of Neuron_total_time (Linear)
RMSE
B Value
3250
3000
2750
2500
2250
2000
1750
1500
0.02
0.04
0.06
0.08
0.10

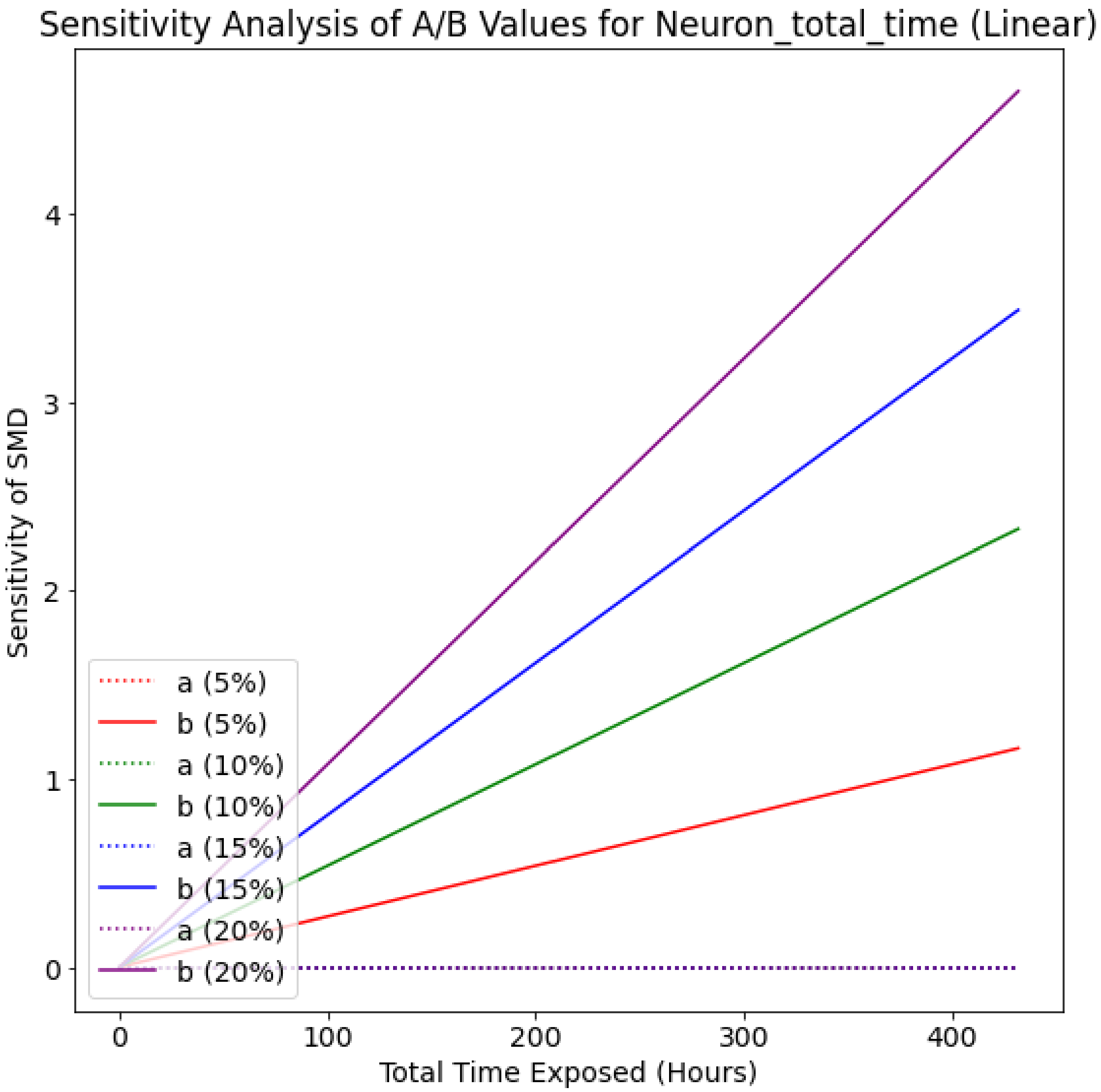
Sensitivity Analysis of A/B Values for Neuron_total_time (Linear)
Sensitivity of SMD
Total Time Exposed (Hours)
0
1
2
3
4
0
100
200
300
400
a (5%)
b (5%)
a (10%)
b (10%)
a (15%)
b (15%)
a (20%)
b (20%)

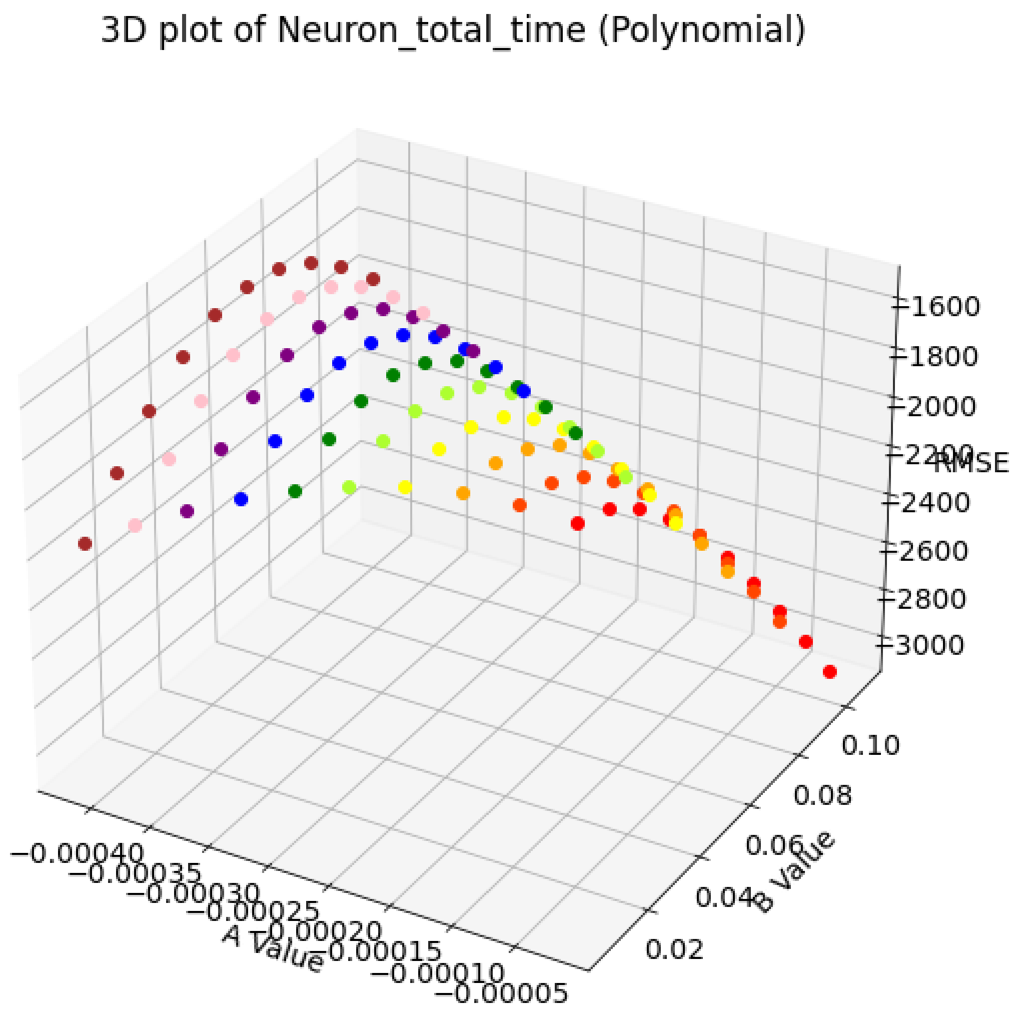
3D plot of Neuron_total_time (Polynomial)
RMSE
1600
1800
2000
2200
2400
2600
2800
3000
B Value
0.02
0.04
0.06
0.08
0.10
A Value
−0.00040
−0.00035
−0.00030
−0.00025
−0.00020
−0.00015
−0.00010
−0.00005

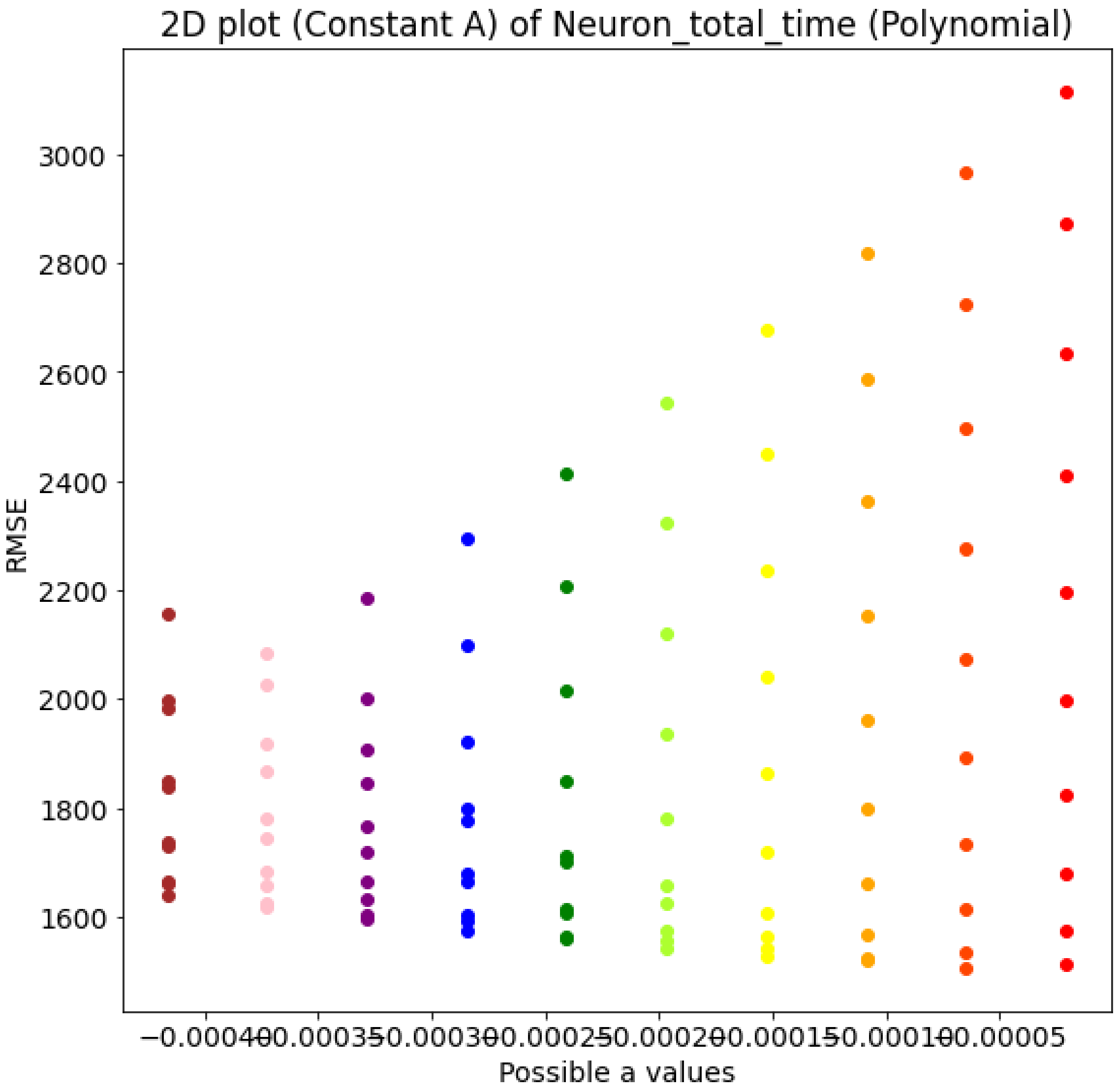
2D plot (Constant A) of Neuron_total_time (Polynomial)
RMSE
3000
2800
2600
2400
2200
2000
1800
1600
−0.00040
−0.00035
−0.00030
−0.00025
−0.00020
−0.00015
−0.00010
−0.00005
Possible a values

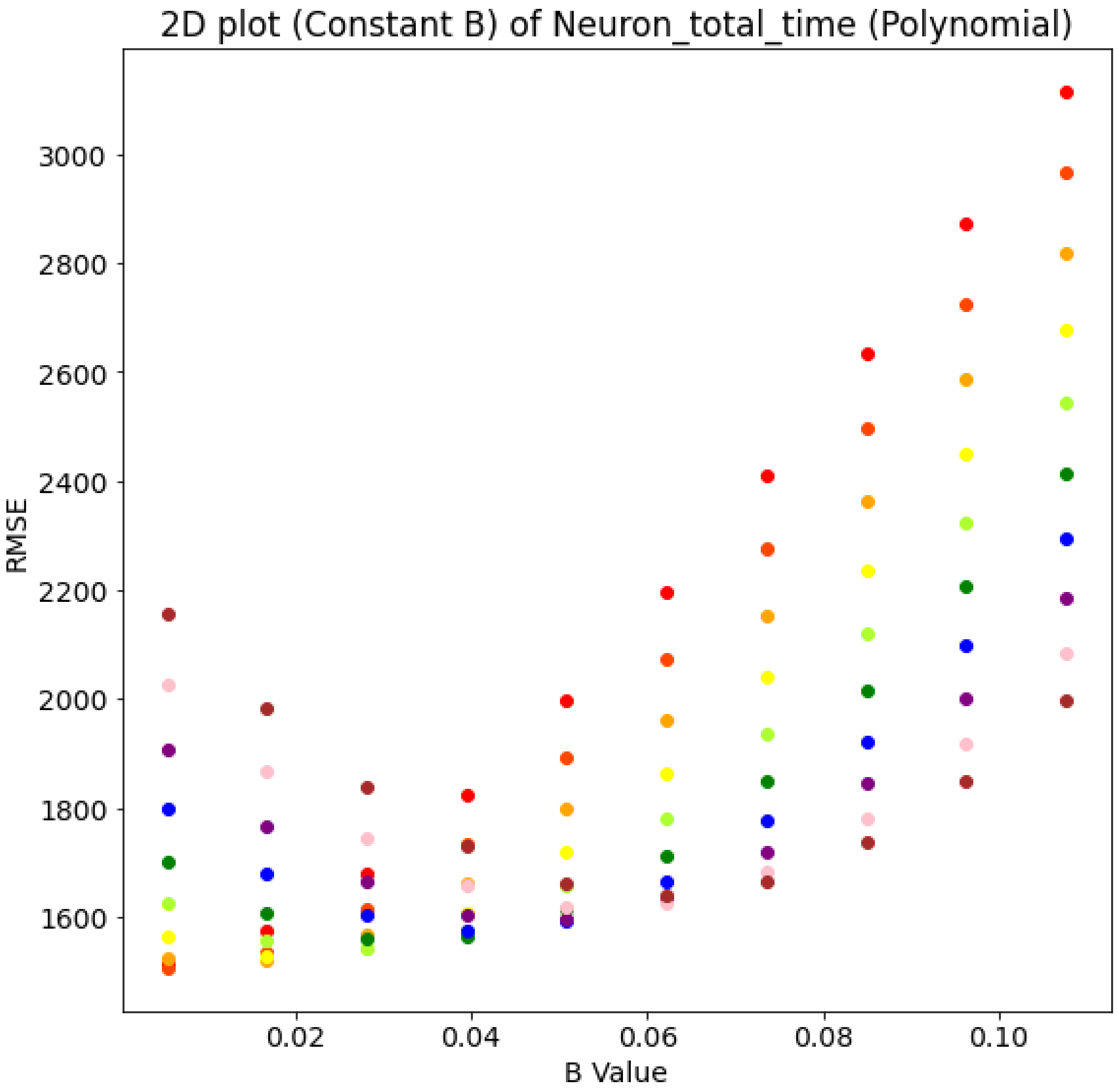
2D plot (Constant B) of Neuron_total_time (Polynomial)
RMSE
3000
2800
2600
2400
2200
2000
1800
1600
0.02
0.04
0.06
0.08
0.10
B Value

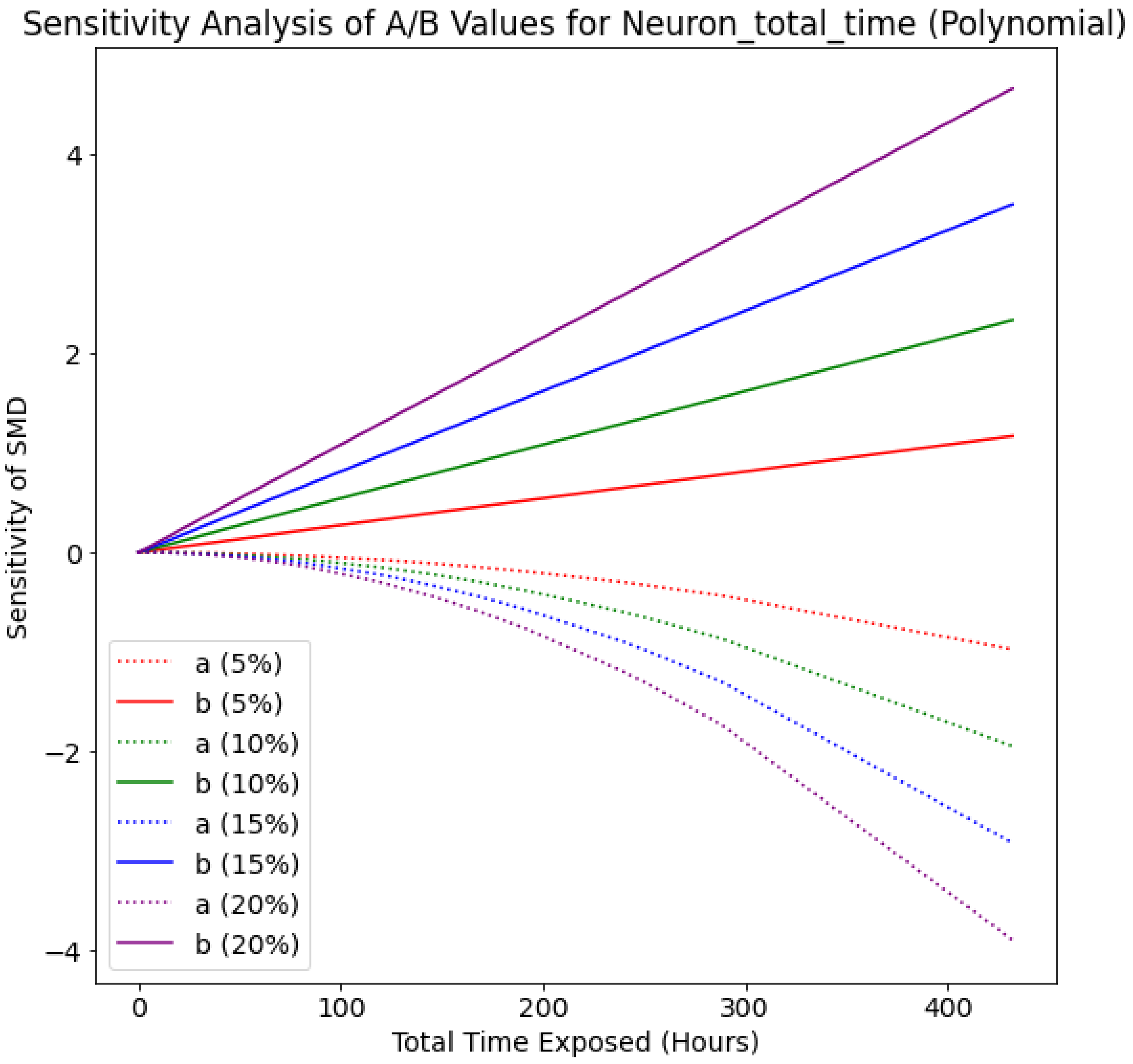
Sensitivity Analysis of A/B Values for Neuron_total_time (Polynomial)
Sensitivity of SMD
Total Time Exposed (Hours)
4
2
0
−2
−4
0
100
200
300
400
a (5%)
b (5%)
a (10%)
b (10%)
a (15%)
b (15%)
a (20%)
b (20%)

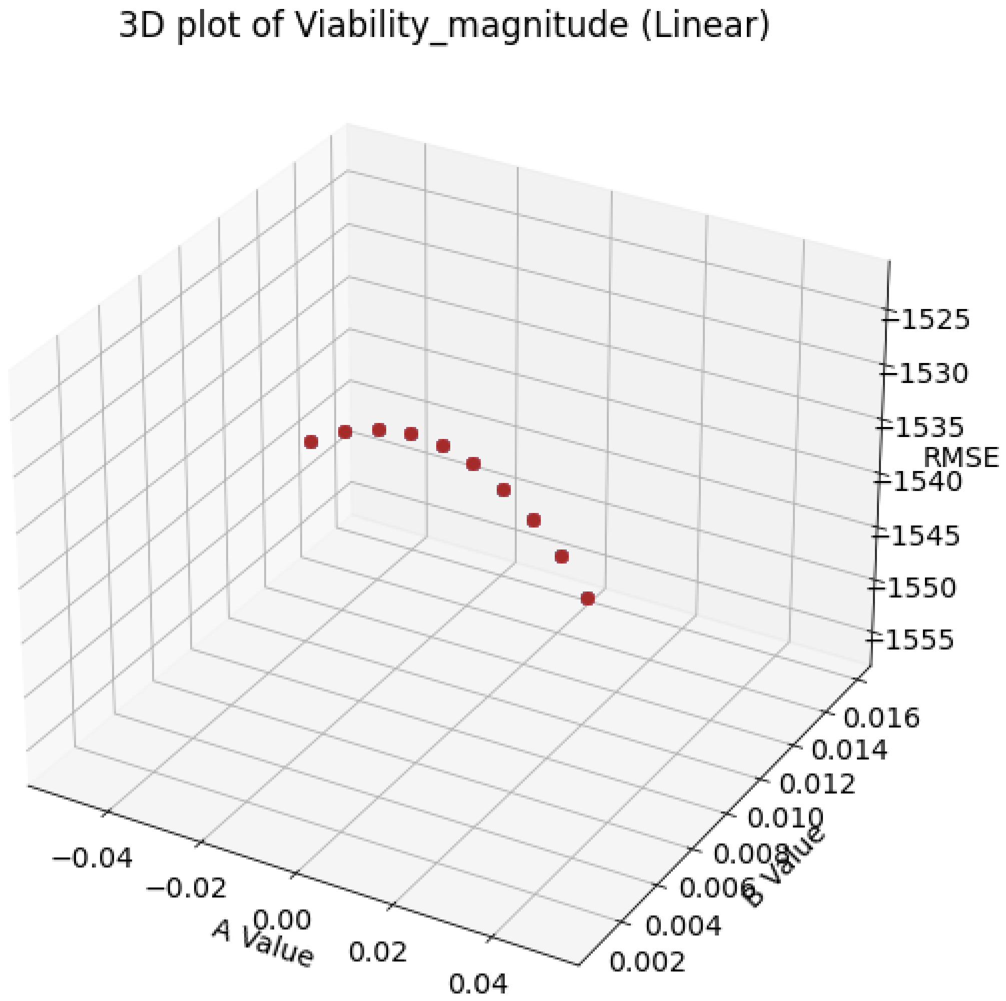
3D plot of Viability_magnitude (Linear)
RMSE
1525
1530
1535
1540
1545
1550
1555
A Value
−0.04
−0.02
0.00
0.02
0.04
B Value
0.002
0.004
0.006
0.008
0.010
0.012
0.014
0.016

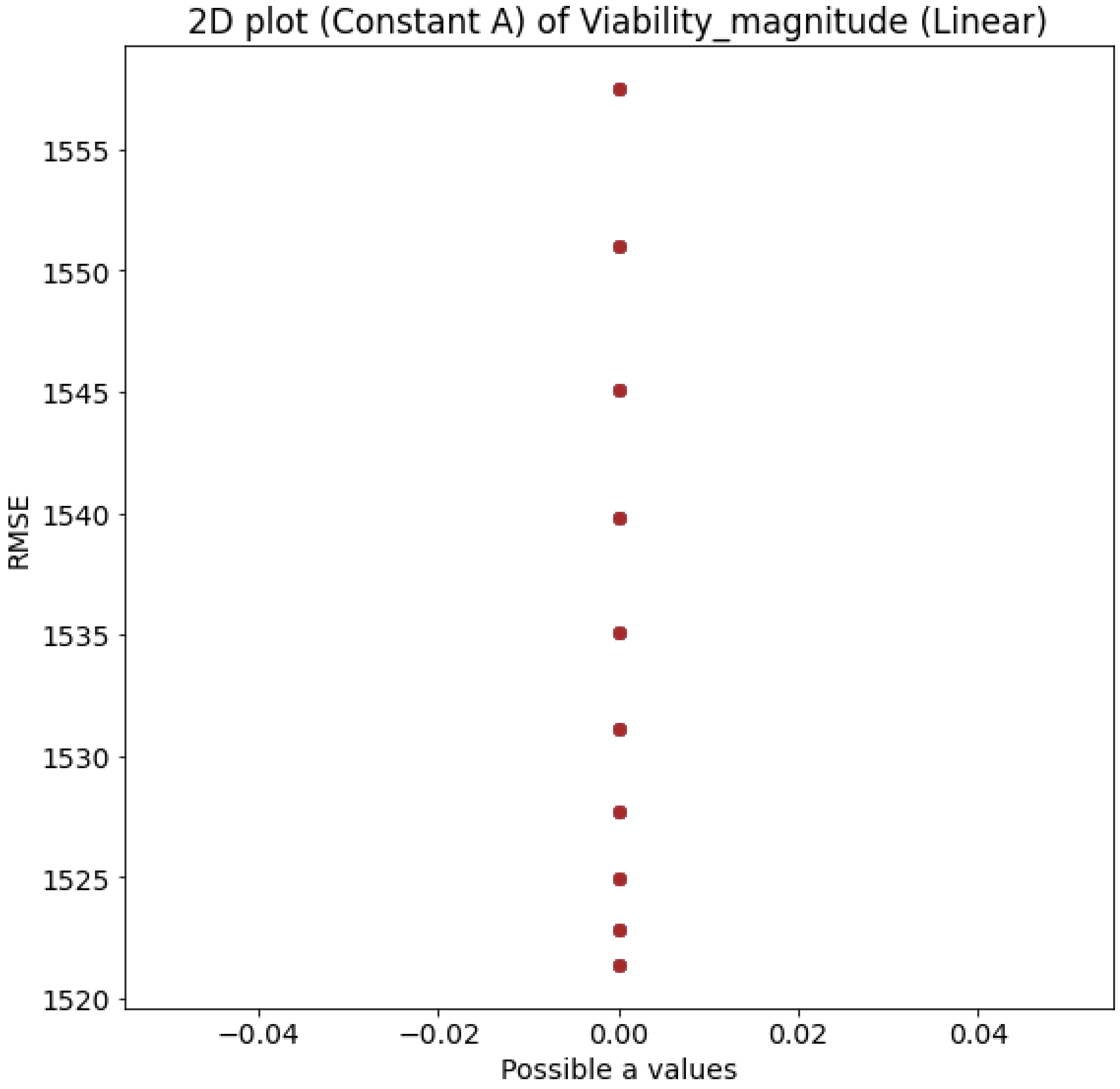
2D plot (Constant A) of Viability_magnitude (Linear)
RMSE
1555
1550
1545
1540
1535
1530
1525
1520
−0.04
−0.02
0.00
0.02
0.04
Possible a values

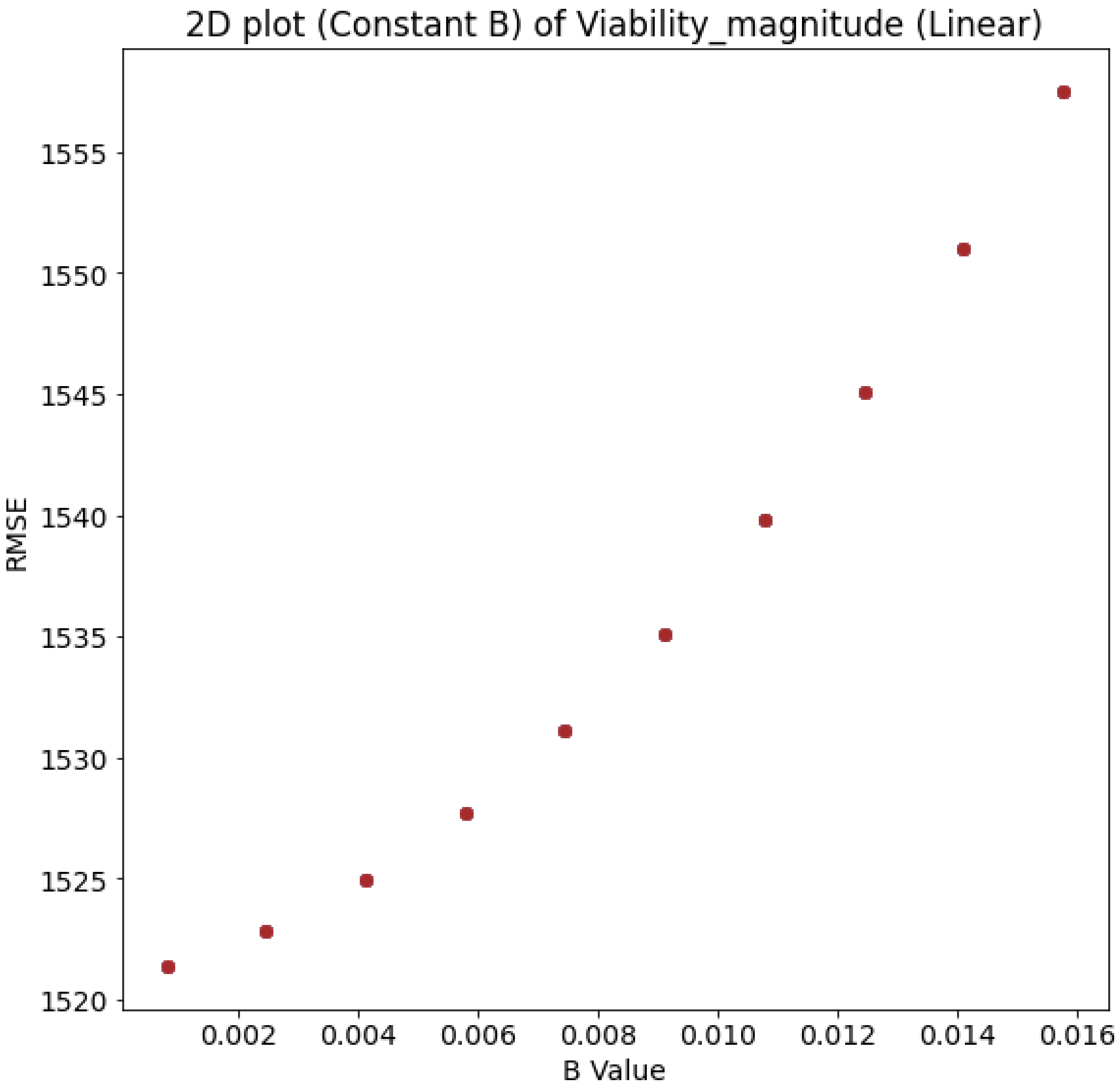
2D plot (Constant B) of Viability_magnitude (Linear)
RMSE
B Value
1520
1525
1530
1535
1540
1545
1550
1555
0.002
0.004
0.006
0.008
0.010
0.012
0.014
0.016

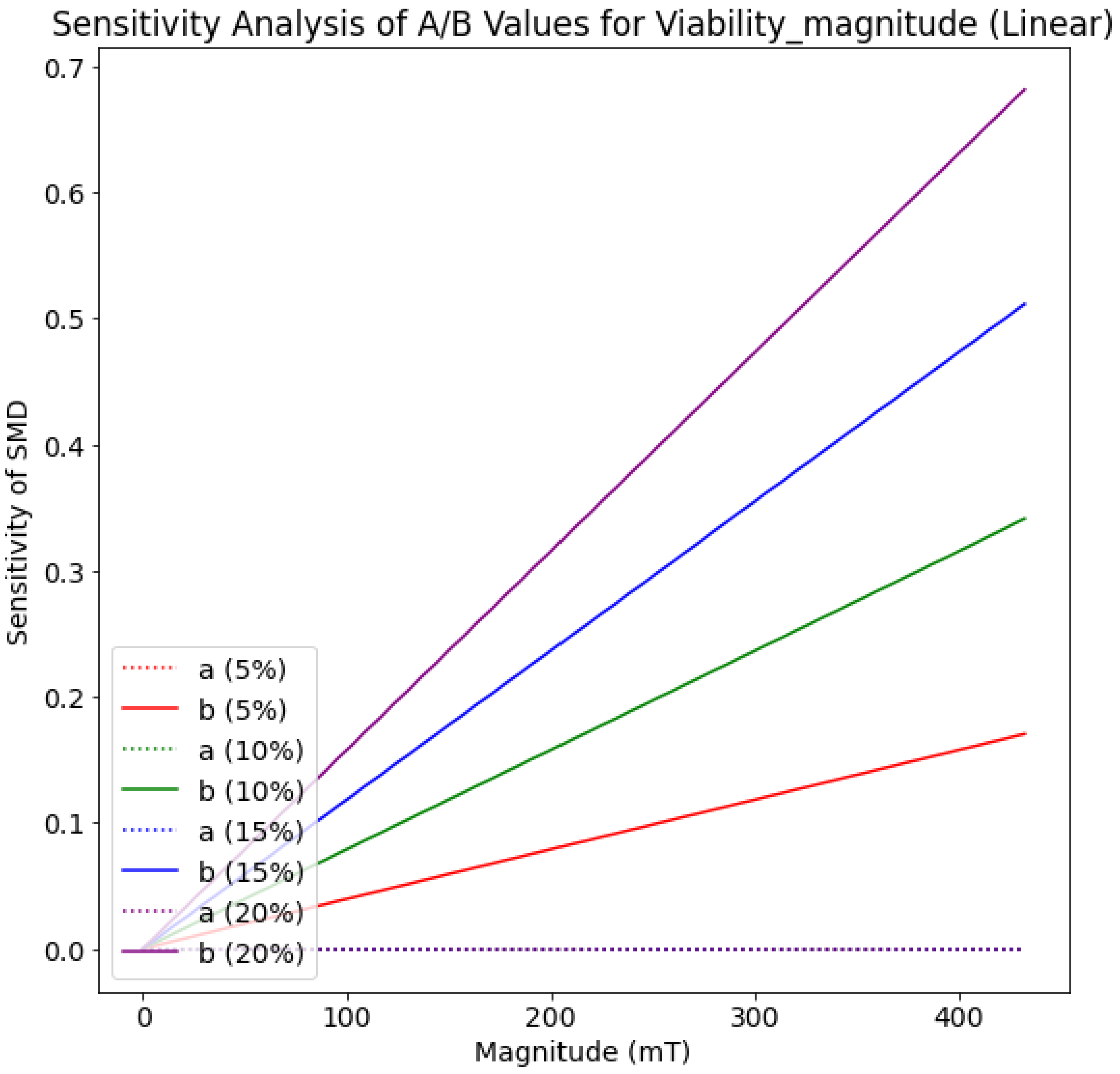
Sensitivity Analysis of A/B Values for Viability_magnitude (Linear)
Sensitivity of SMD
Magnitude (mT)
0.0
0.1
0.2
0.3
0.4
0.5
0.6
0.7
0
100
200
300
400
a (5%)
b (5%)
a (10%)
b (10%)
a (15%)
b (15%)
a (20%)
b (20%)

3D plot of Viability_magnitude (Polynomial)

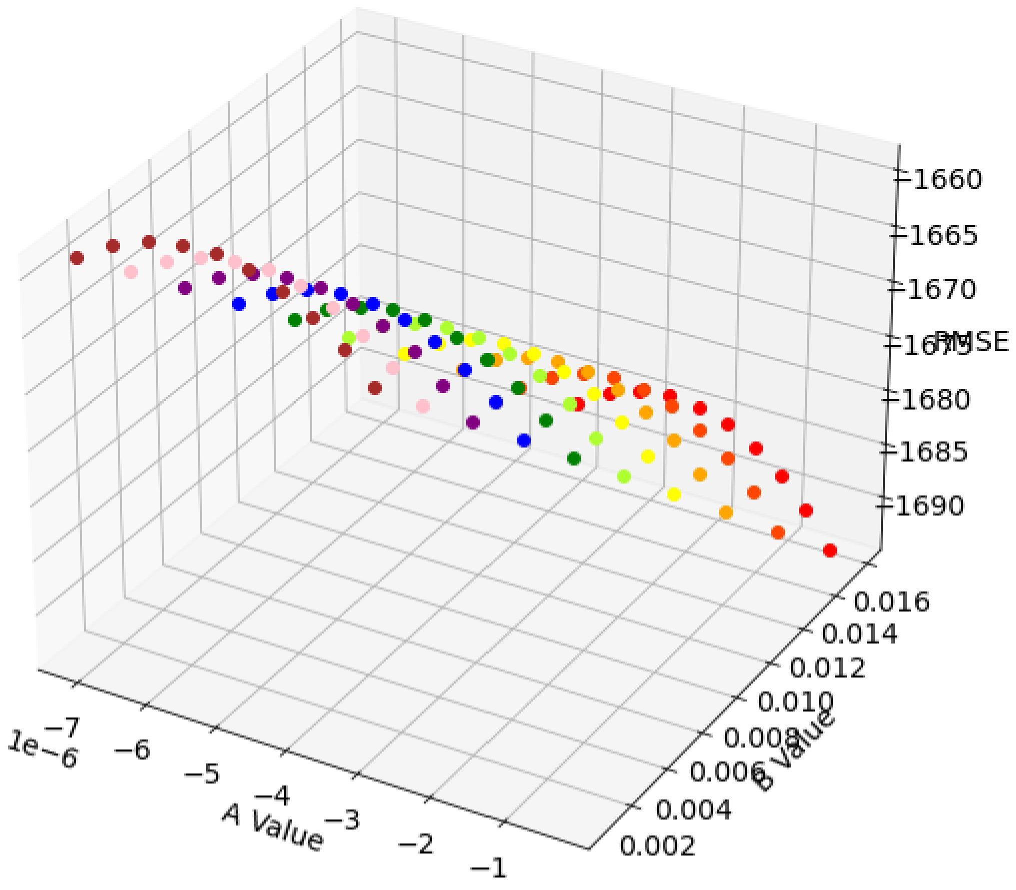

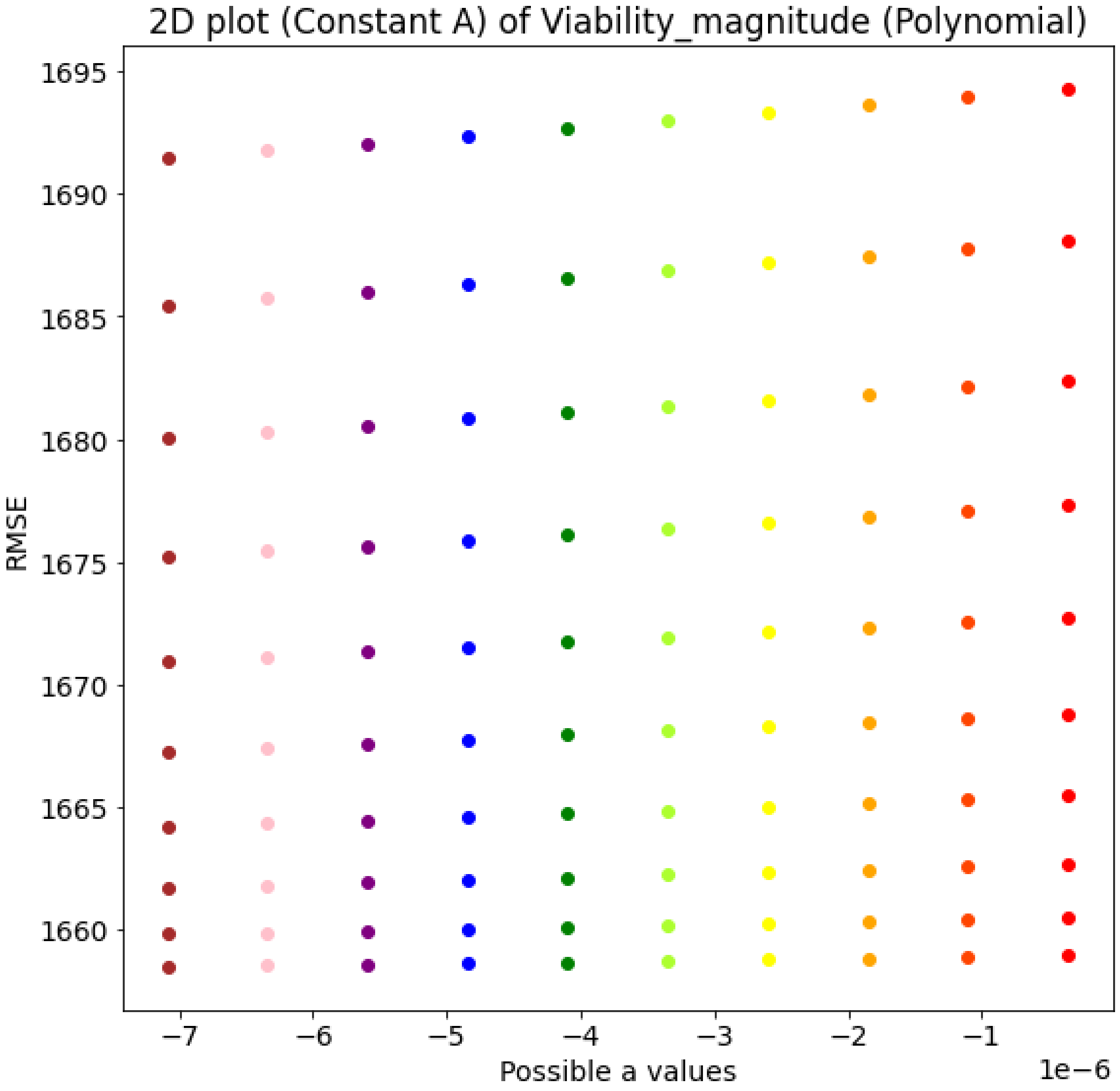
2D plot (Constant A) of Viability_magnitude (Polynomial)
1695
1690
1685
1680
1675
1670
1665
1660
RMSE
−7
−6
−5
−4
−3
−2
−1
Possible a values
1e−6

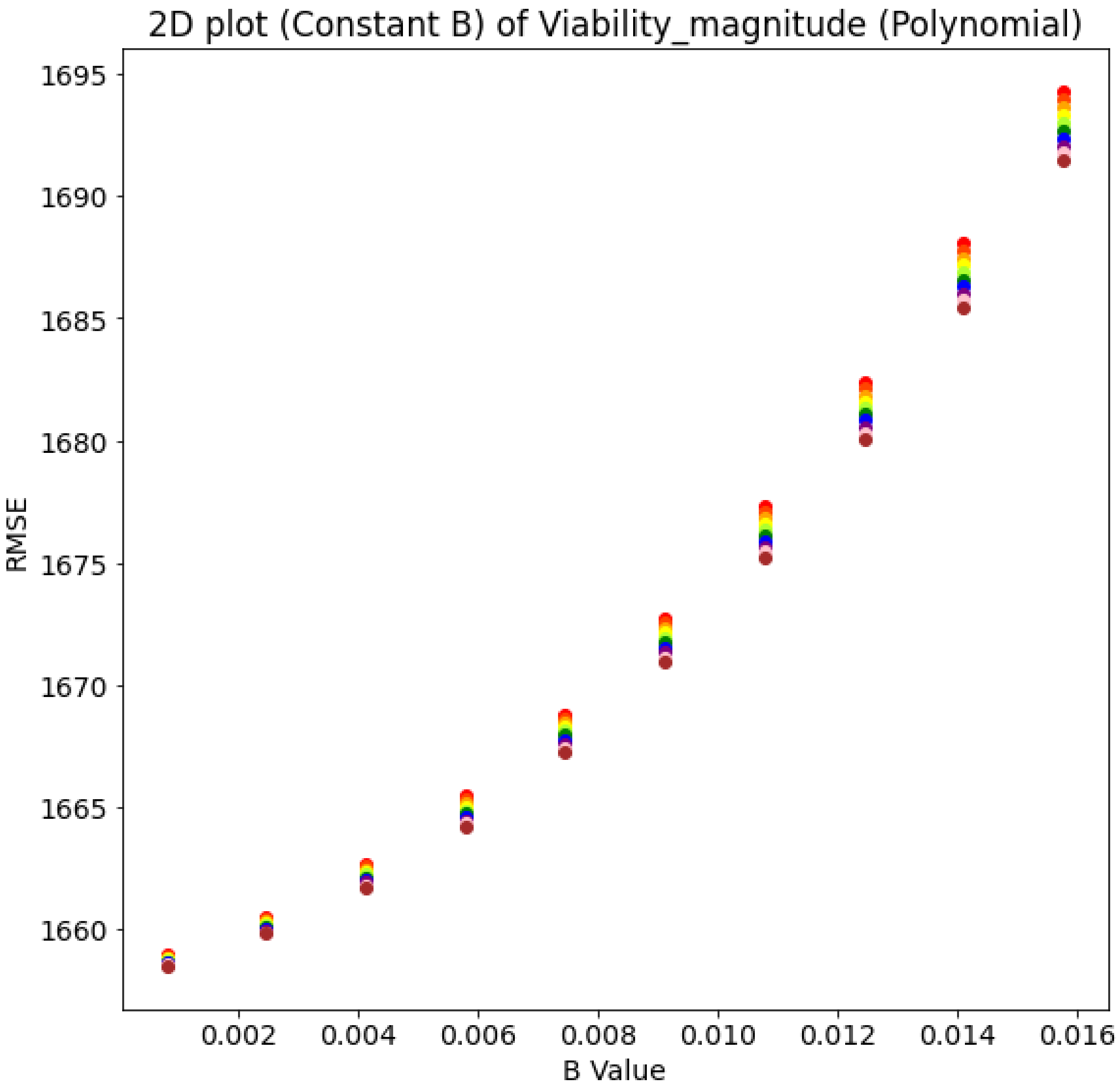
2D plot (Constant B) of Viability_magnitude (Polynomial)
RMSE
B Value
1695
1690
1685
1680
1675
1670
1665
1660
0.002
0.004
0.006
0.008
0.010
0.012
0.014
0.016

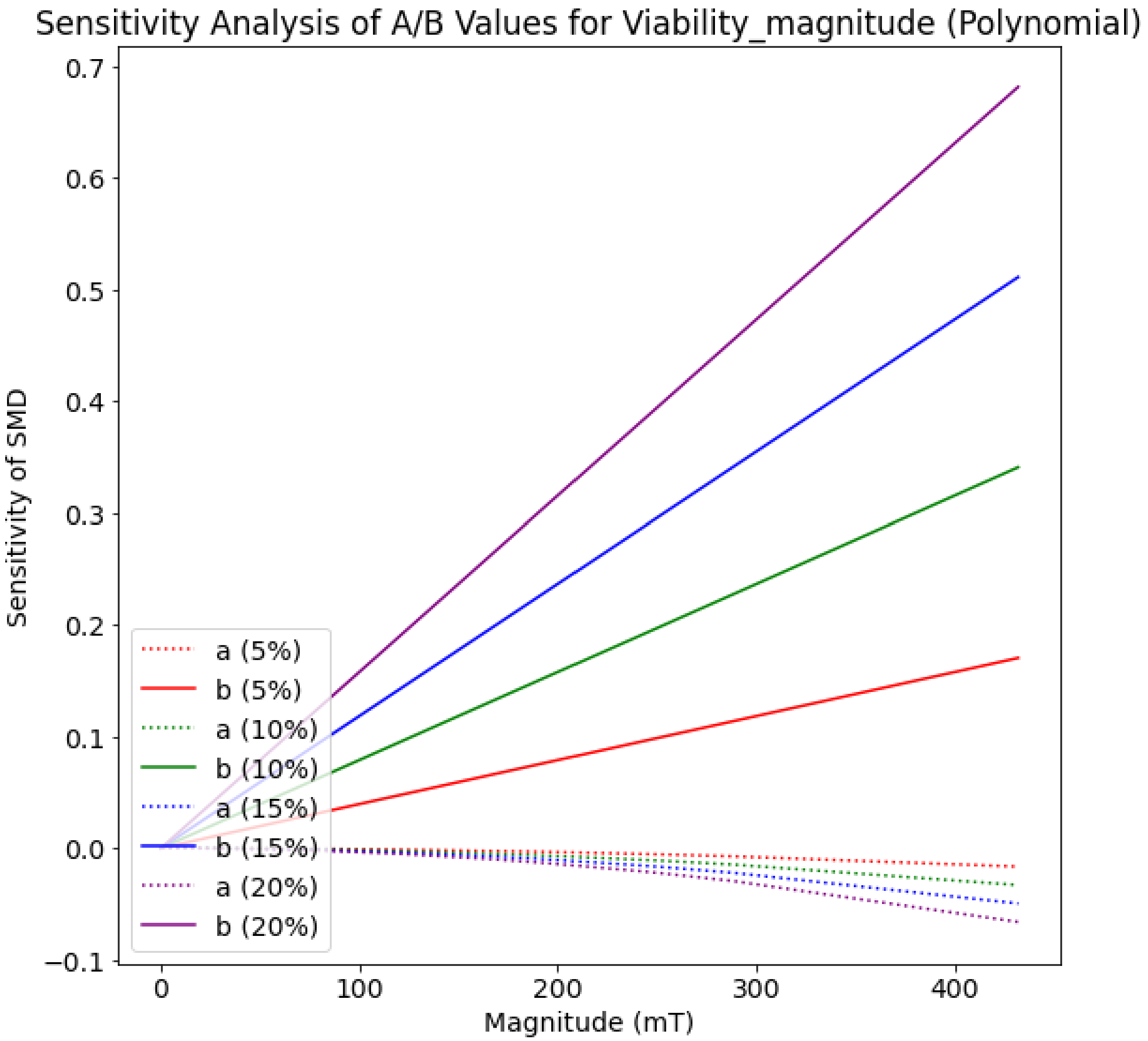
Sensitivity Analysis of A/B Values for Viability_magnitude (Polynomial)
Sensitivity of SMD
Magnitude (mT)
0.7
0.6
0.5
0.4
0.3
0.2
0.1
0.0
−0.1
0
100
200
300
400
a (5%)
b (5%)
a (10%)
b (10%)
a (15%)
b (15%)
a (20%)
b (20%)

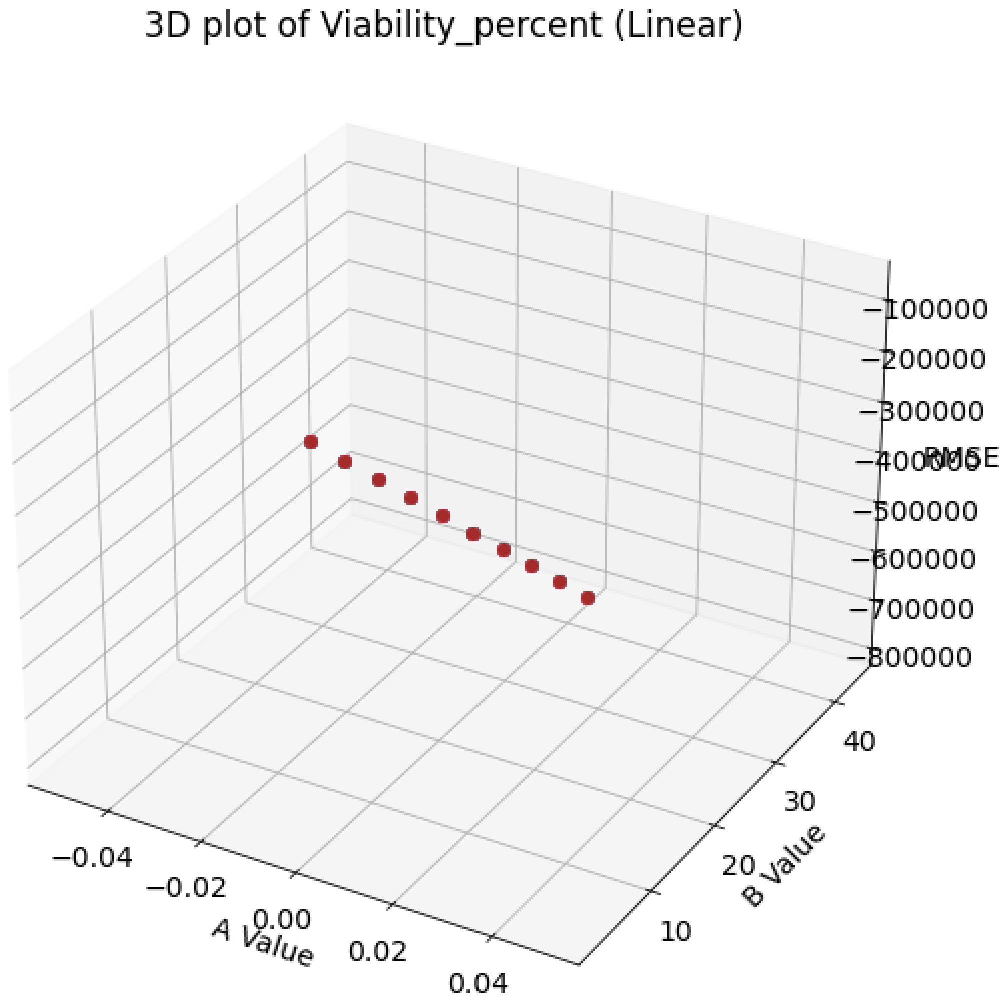
3D plot of Viability_percent (Linear)
−100000
−200000
−300000
−400000
−500000
−600000
−700000
−800000
RMSE
40
30
20
10
B Value
−0.04
−0.02
0.00
0.02
0.04
A Value

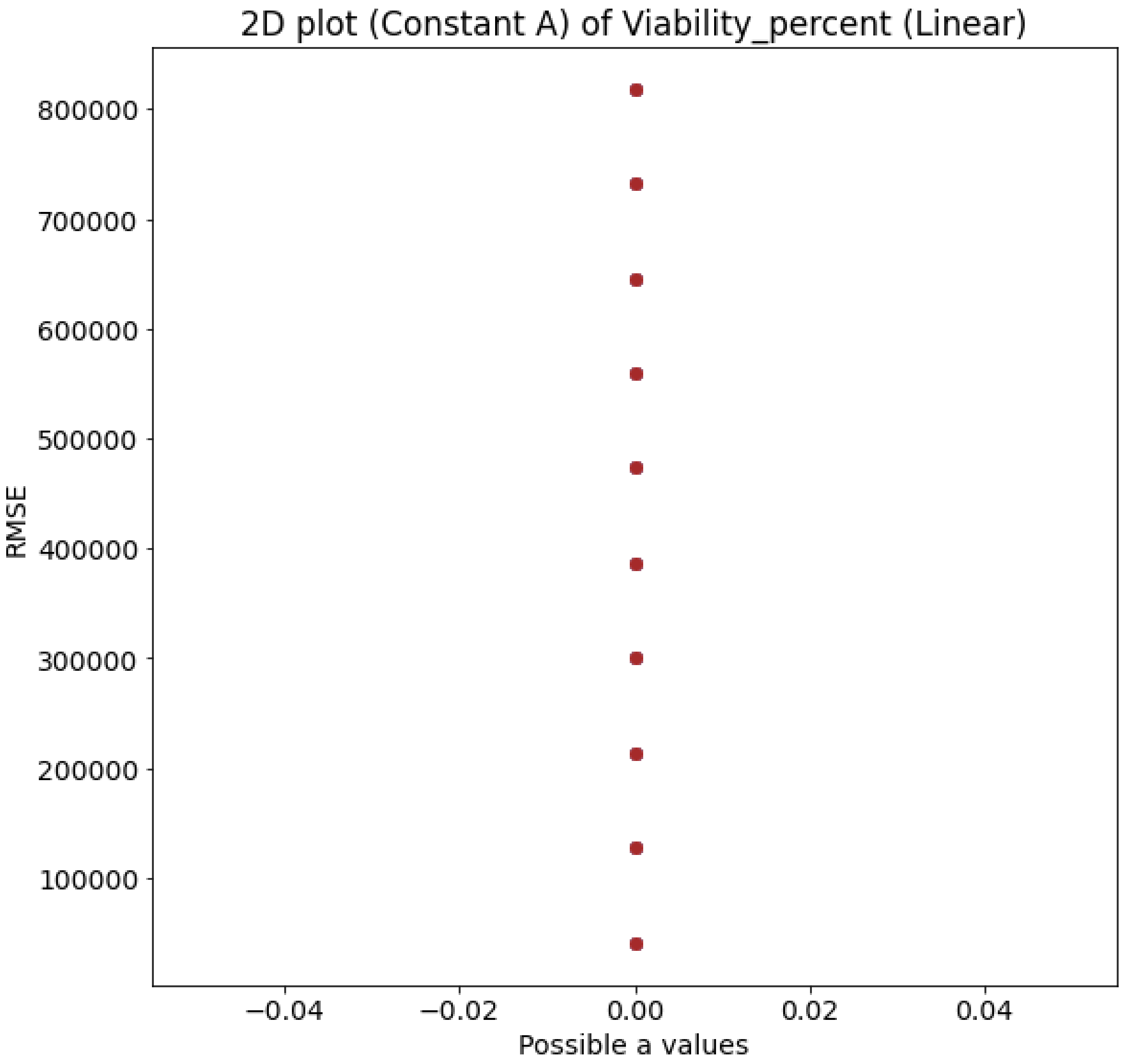
2D plot (Constant A) of Viability_percent (Linear)
RMSE
800000
700000
600000
500000
400000
300000
200000
100000
−0.04
−0.02
0.00
0.02
0.04
Possible a values

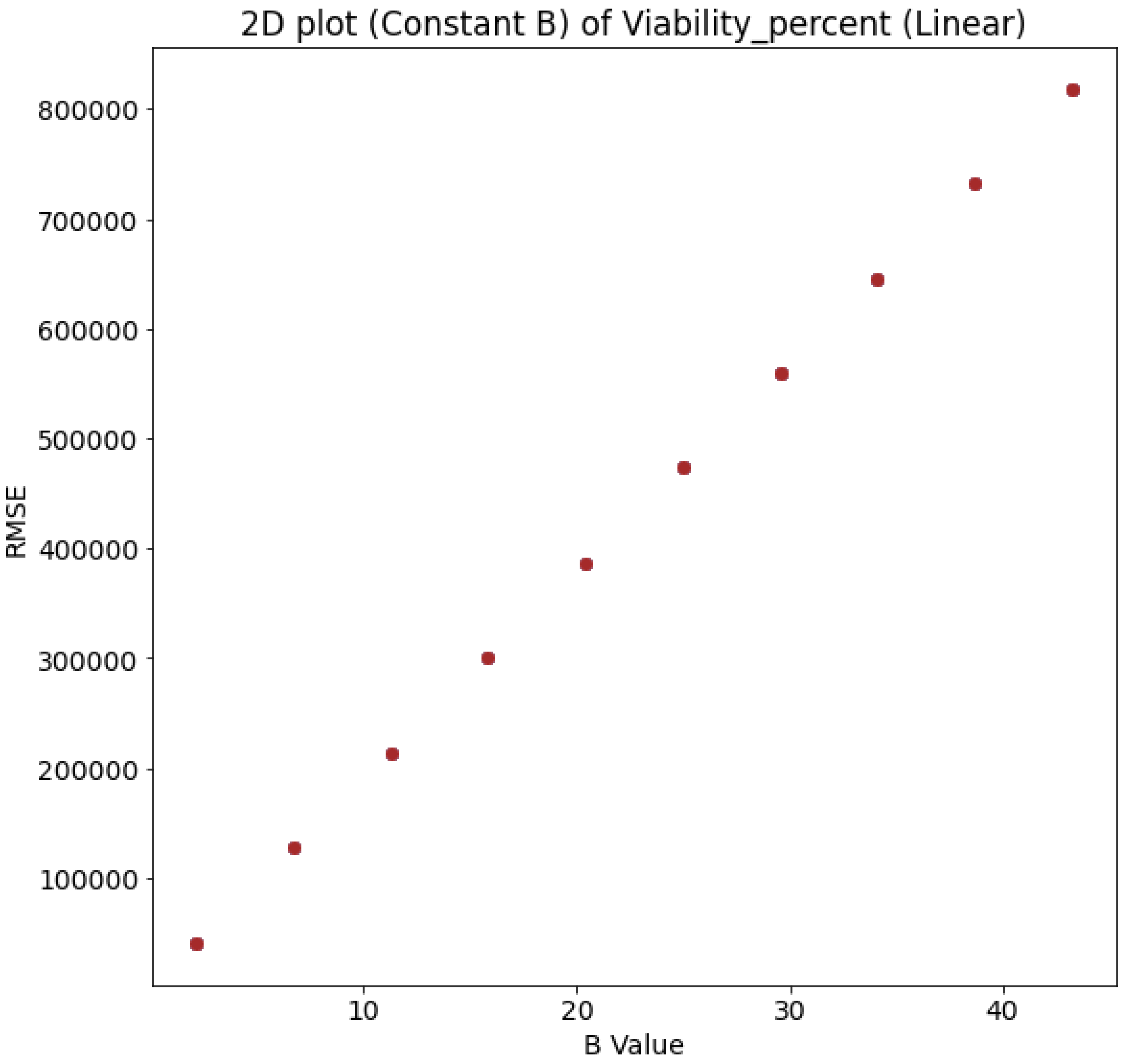
2D plot (Constant B) of Viability_percent (Linear)
RMSE
800000
700000
600000
500000
400000
300000
200000
100000
10
20
30
40
B Value

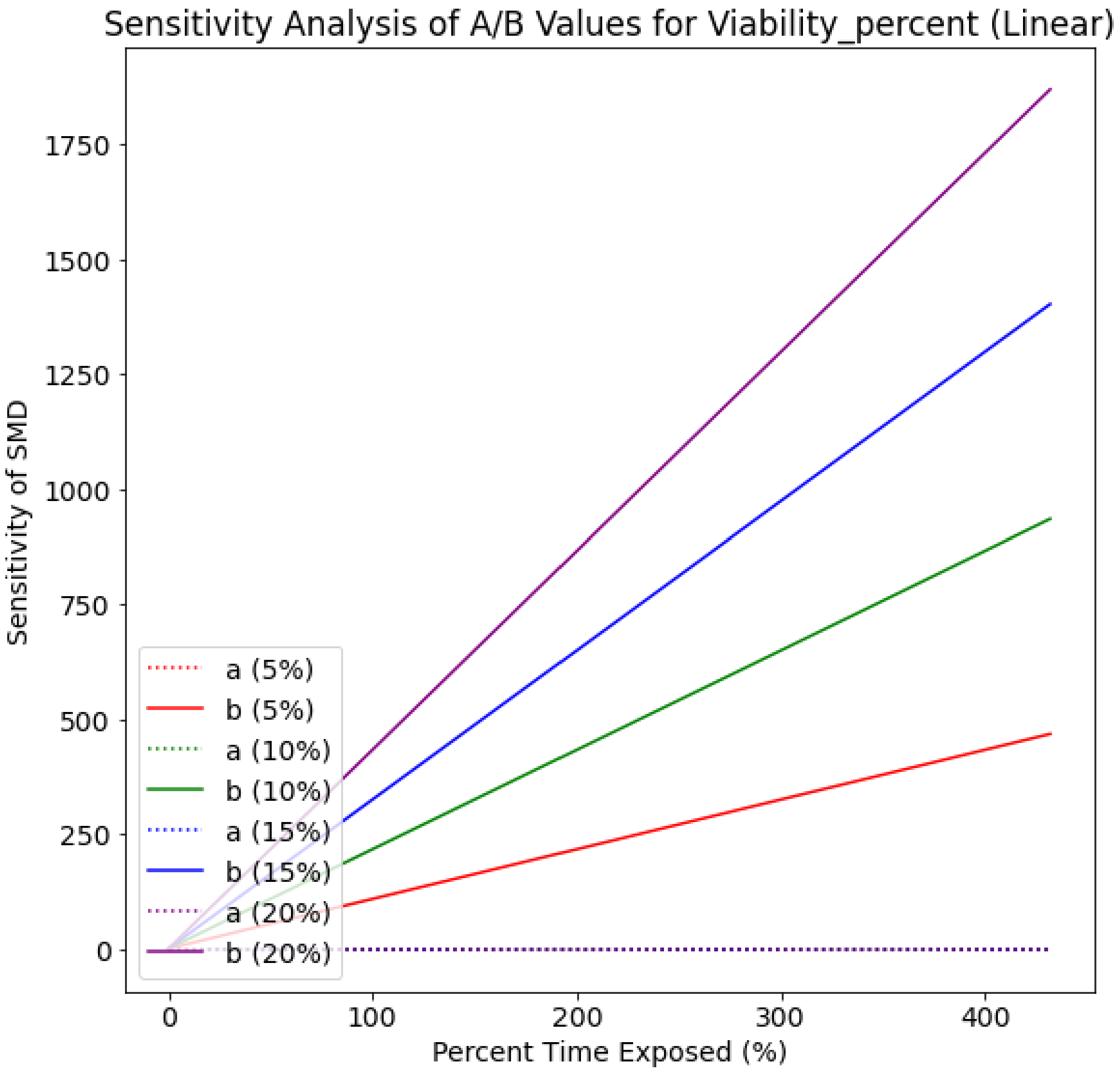
Sensitivity Analysis of A/B Values for Viability_percent (Linear)
Sensitivity of SMD
Percent Time Exposed (%)
0
250
500
750
1000
1250
1500
1750
0
100
200
300
400
a (5%)
b (5%)
a (10%)
b (10%)
a (15%)
b (15%)
a (20%)
b (20%)

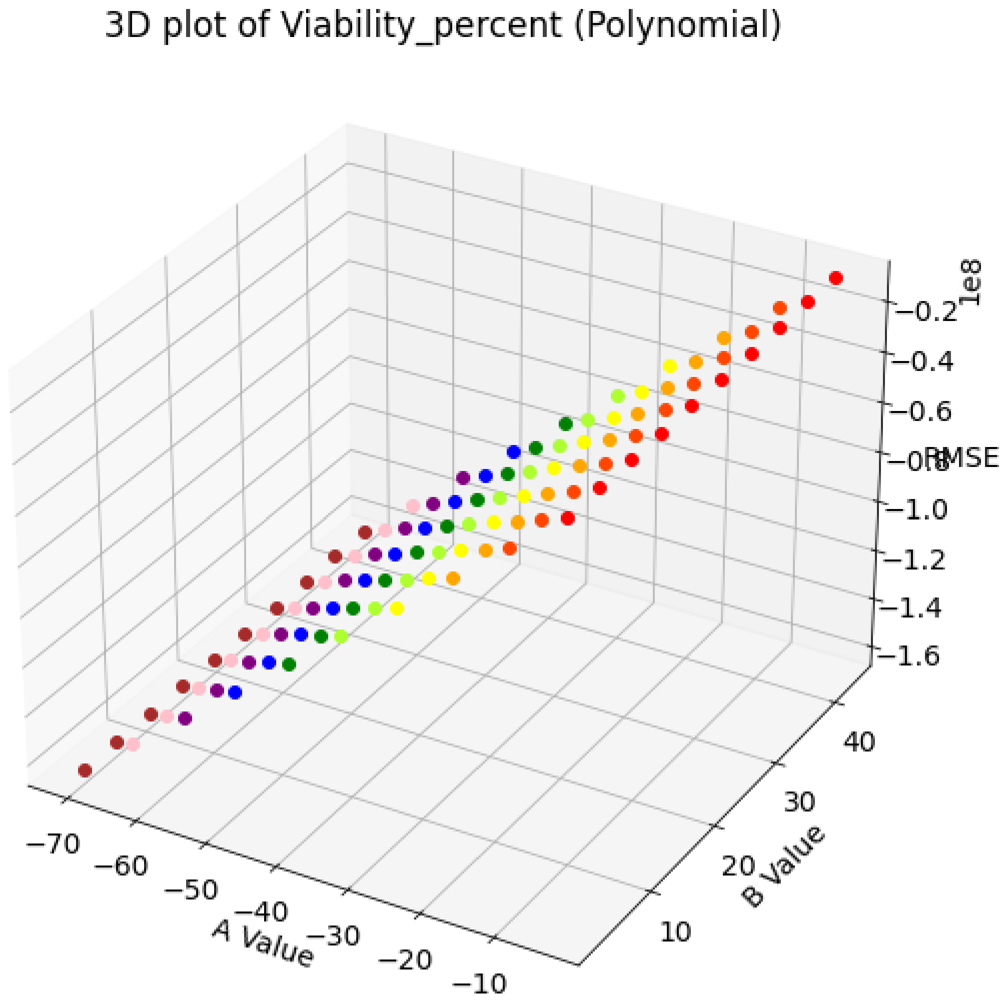
3D plot of Viability_percent (Polynomial)
1e8
−0.2
−0.4
−0.6
−0.8
−1.0
−1.2
−1.4
−1.6
RMSE
40
30
20
10
B Value
−70
−60
−50
−40
−30
−20
−10
A Value

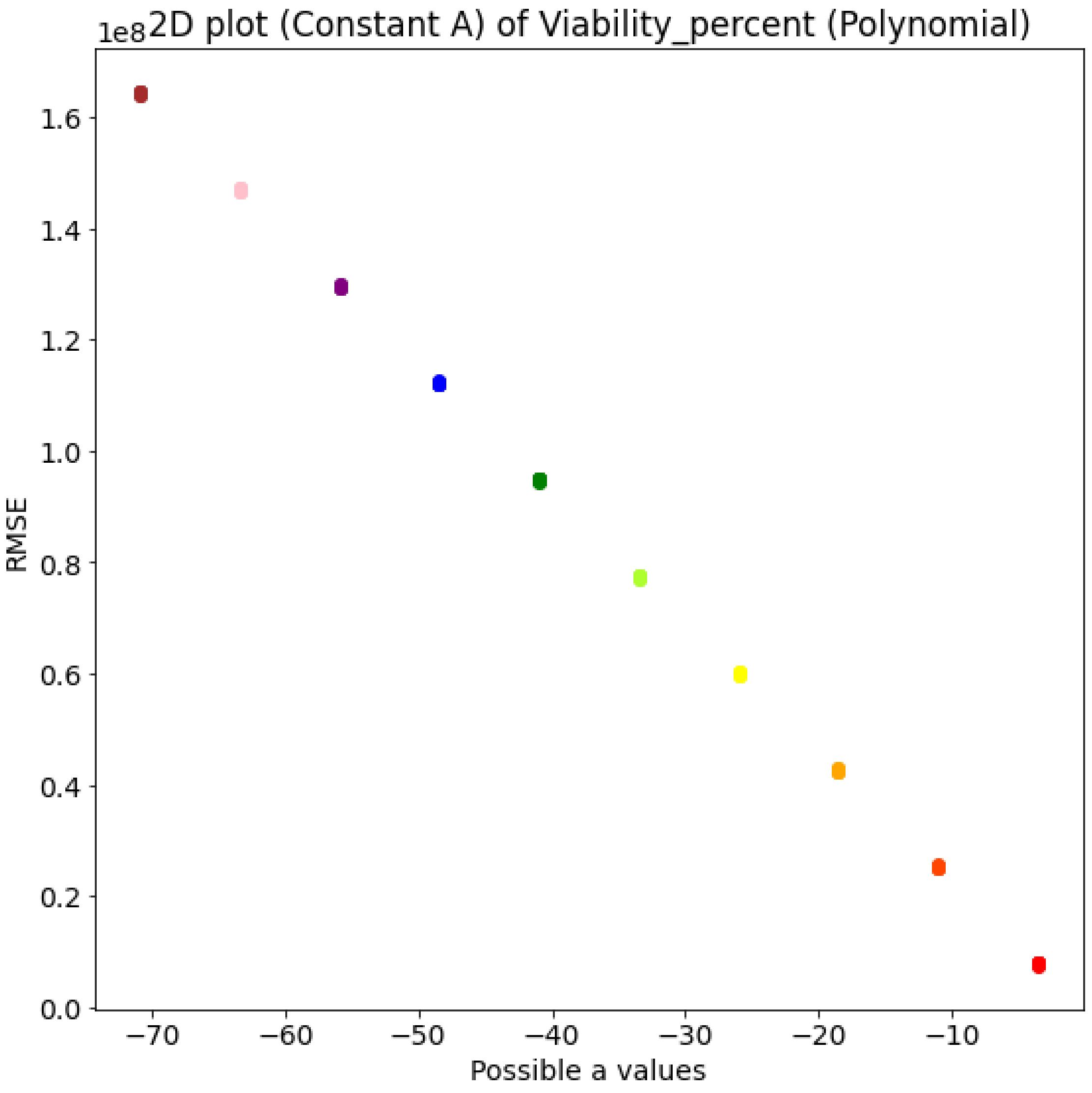
1e8
2D plot (Constant A) of Viability_percent (Polynomial)
RMSE
1.6
1.4
1.2
1.0
0.8
0.6
0.4
0.2
0.0
−70
−60
−50
−40
−30
−20
−10
Possible a values

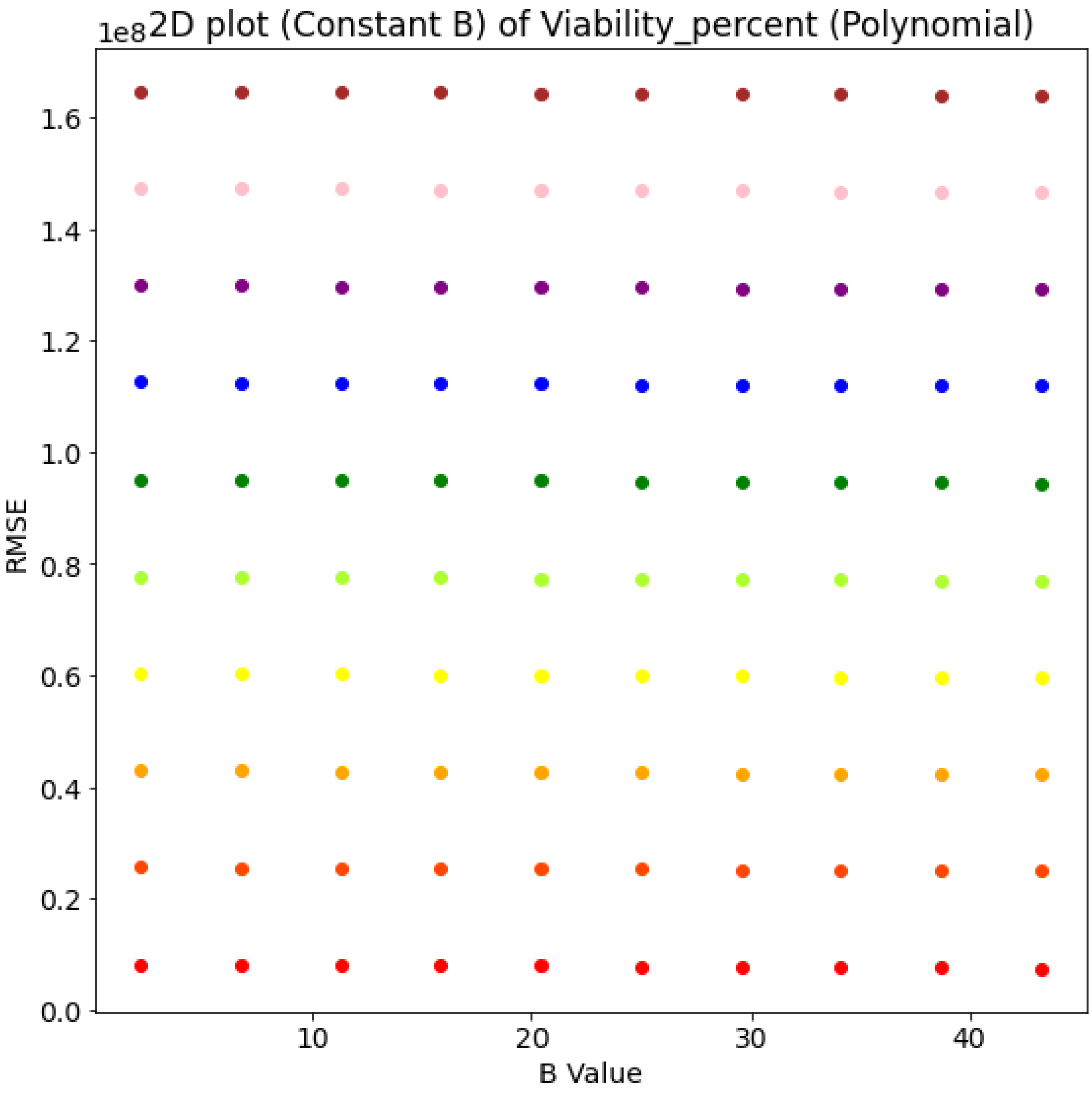
1e8
2D plot (Constant B) of Viability_percent (Polynomial)
RMSE
0.0
0.2
0.4
0.6
0.8
1.0
1.2
1.4
1.6
10
20
30
40
B Value

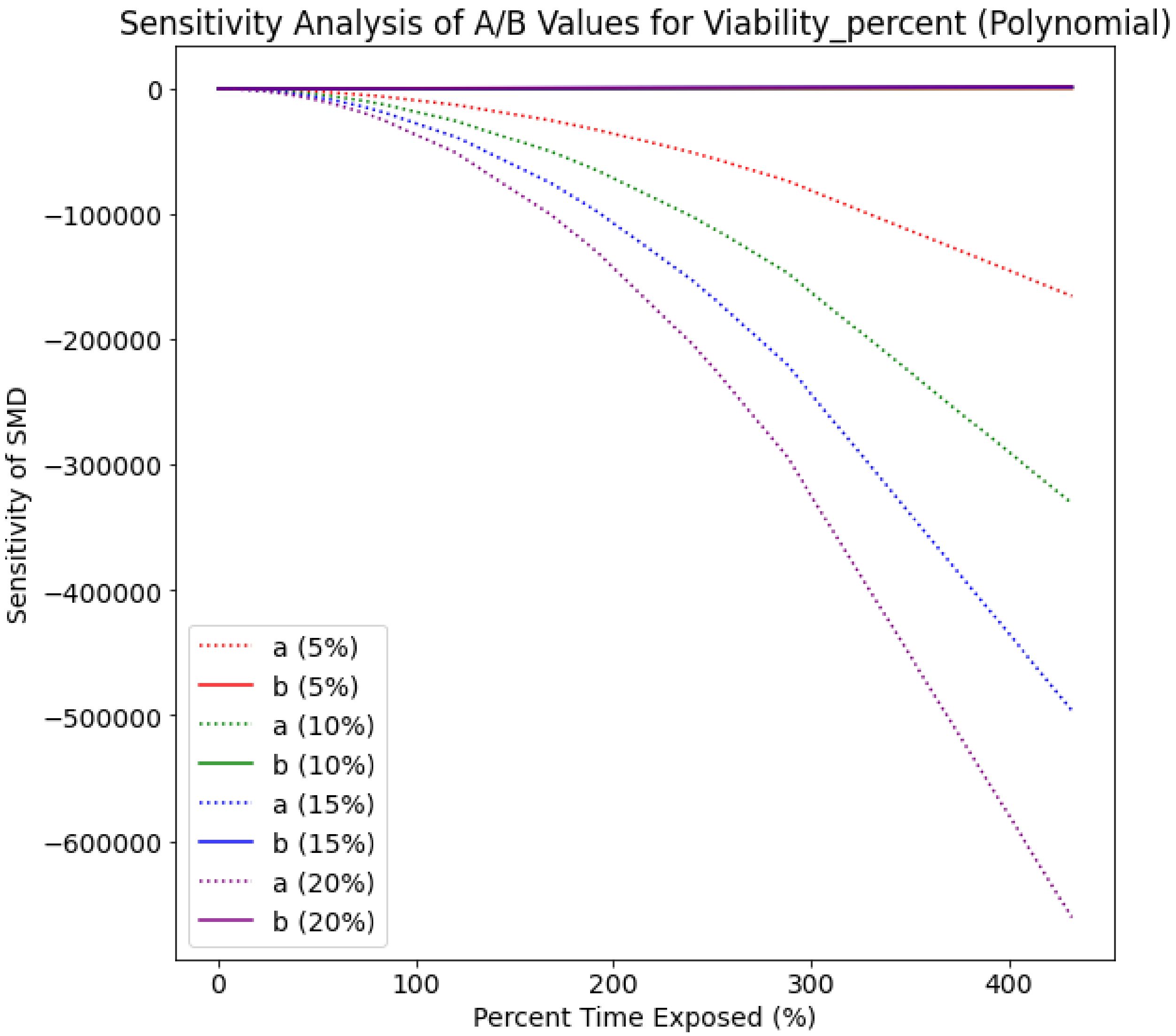
Sensitivity Analysis of A/B Values for Viability_percent (Polynomial)
Sensitivity of SMD
Percent Time Exposed (%)
0
−100000
−200000
−300000
−400000
−500000
−600000
0
100
200
300
400
a (5%)
b (5%)
a (10%)
b (10%)
a (15%)
b (15%)
a (20%)
b (20%)

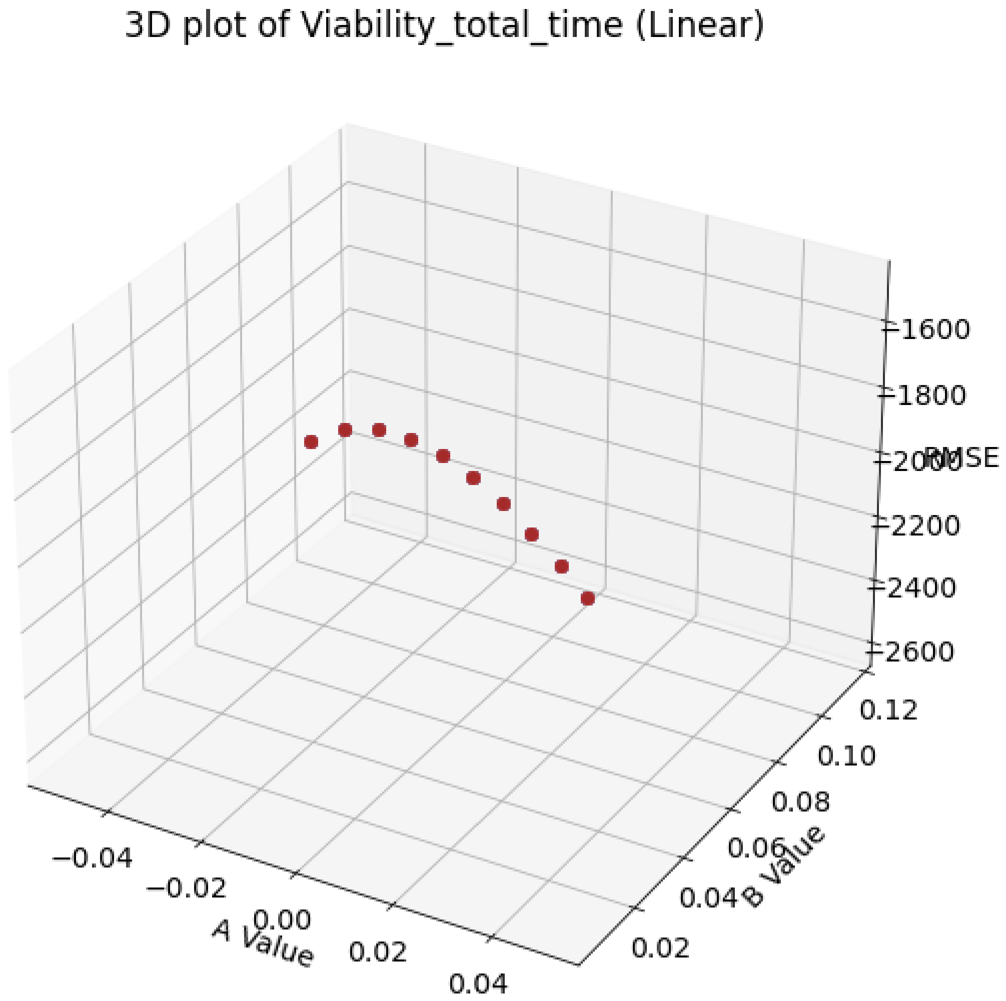
3D plot of Viability_total_time (Linear)
1600
1800
2000
2200
2400
2600
RMSE
0.12
0.10
0.08
0.06
0.04
0.02
B Value
−0.04
−0.02
0.00
0.02
0.04
A Value

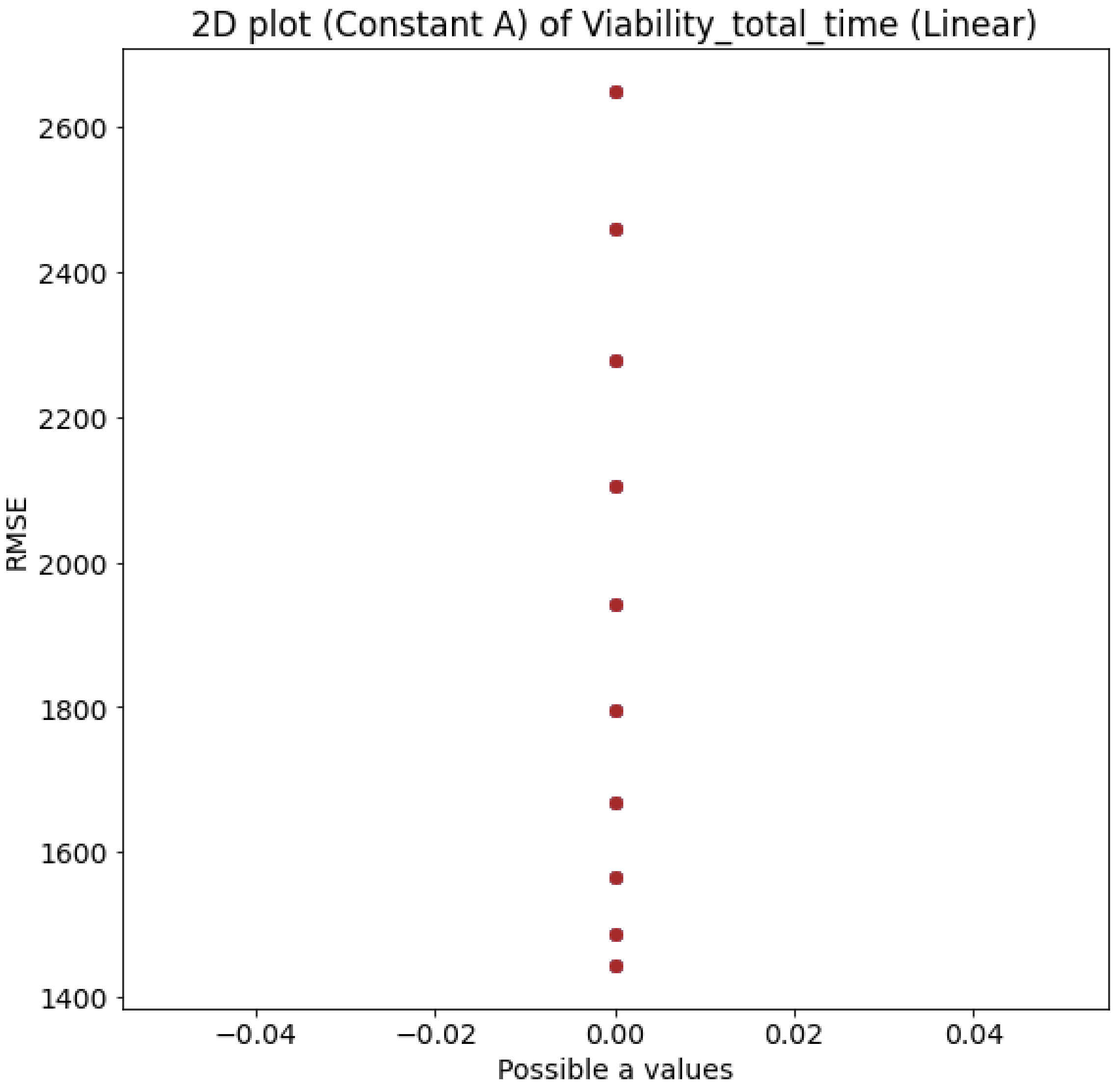
2D plot (Constant A) of Viability_total_time (Linear)
RMSE
2600
2400
2200
2000
1800
1600
1400
−0.04
−0.02
0.00
0.02
0.04
Possible a values

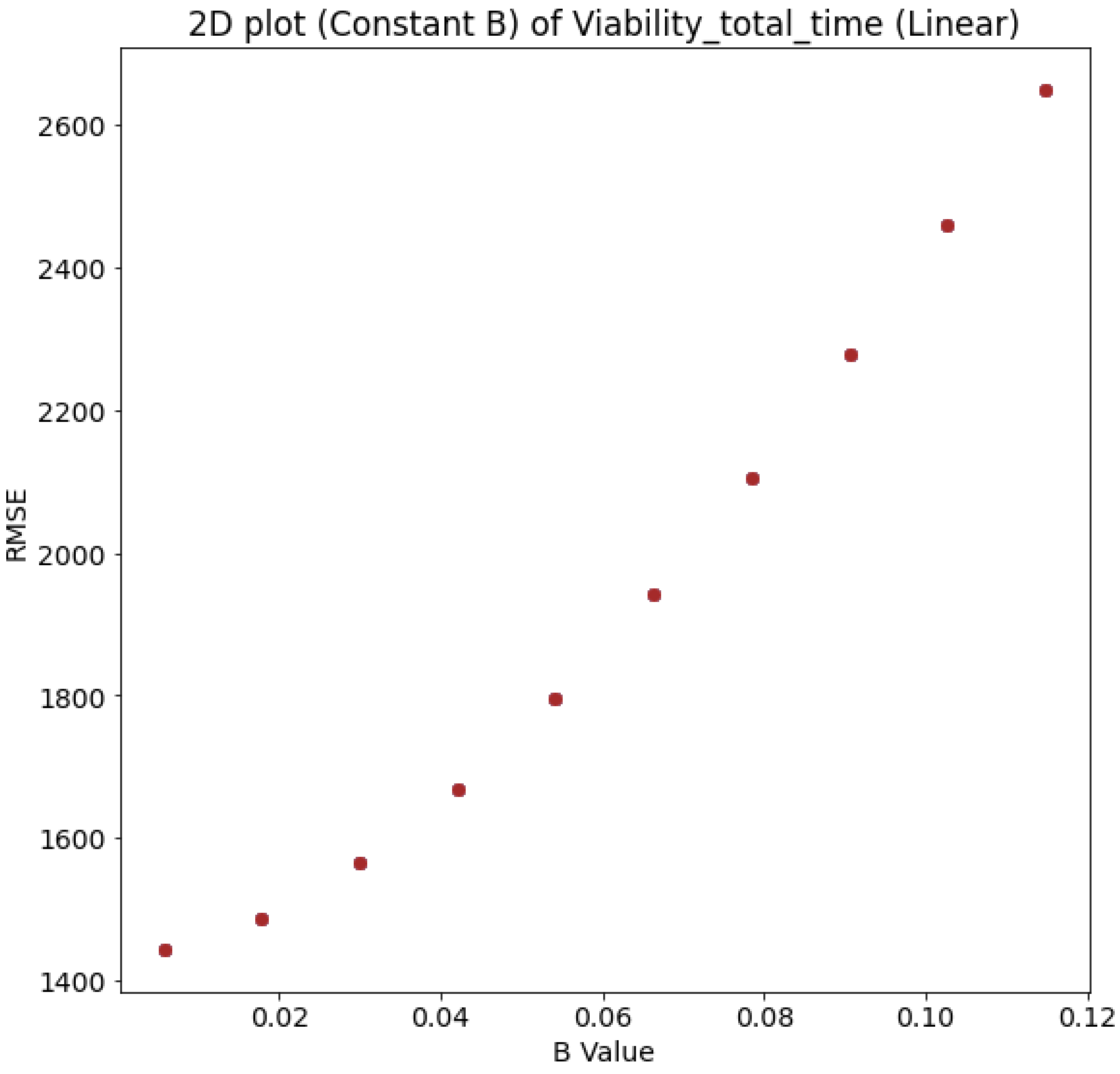
2D plot (Constant B) of Viability_total_time (Linear)
RMSE
2600
2400
2200
2000
1800
1600
1400
0.02
0.04
0.06
0.08
0.10
0.12
B Value

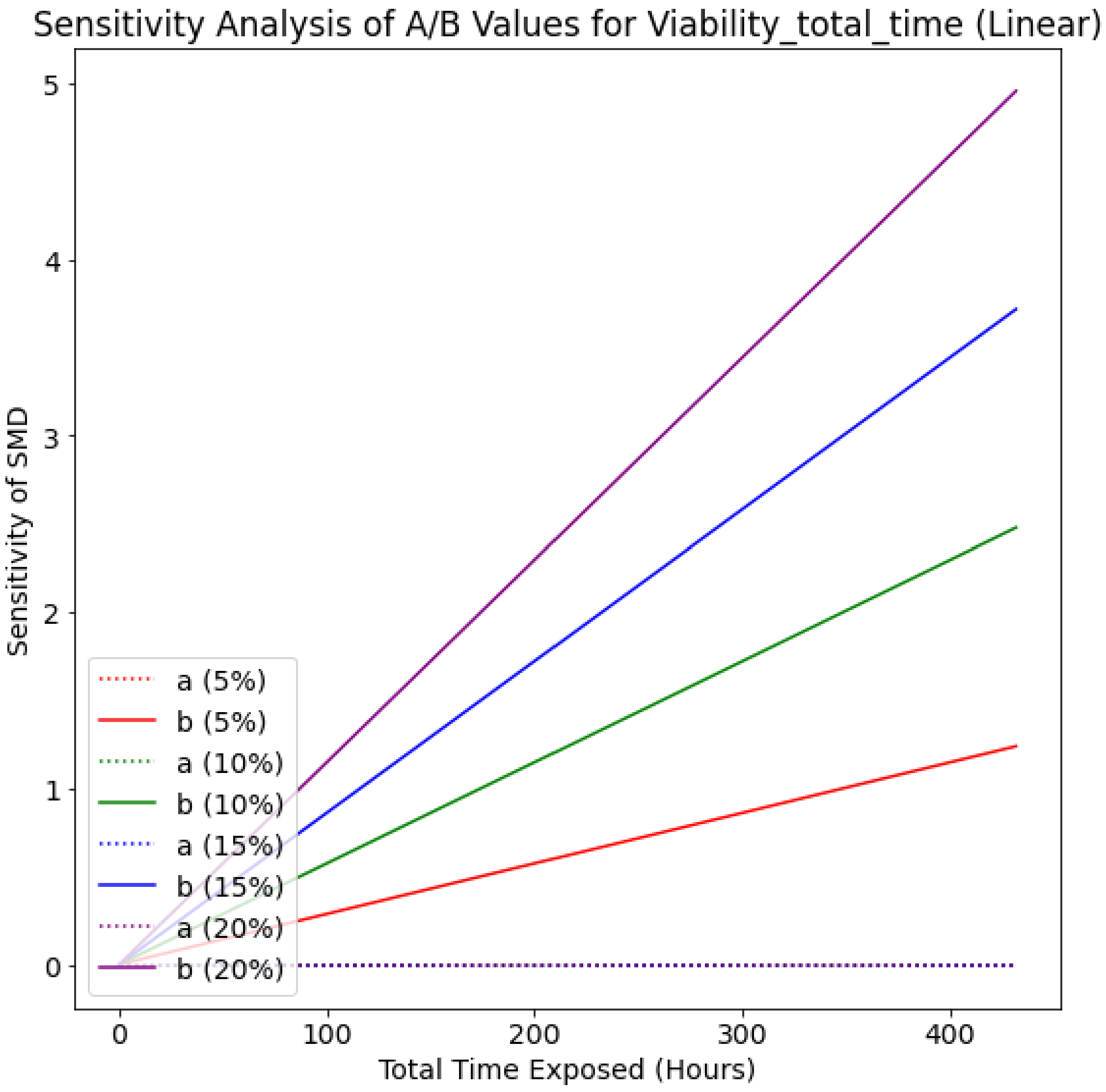
Sensitivity Analysis of A/B Values for Viability_total_time (Linear)
Sensitivity of SMD
Total Time Exposed (Hours)
0
1
2
3
4
5
0
100
200
300
400
a (5%)
b (5%)
a (10%)
b (10%)
a (15%)
b (15%)
a (20%)
b (20%)

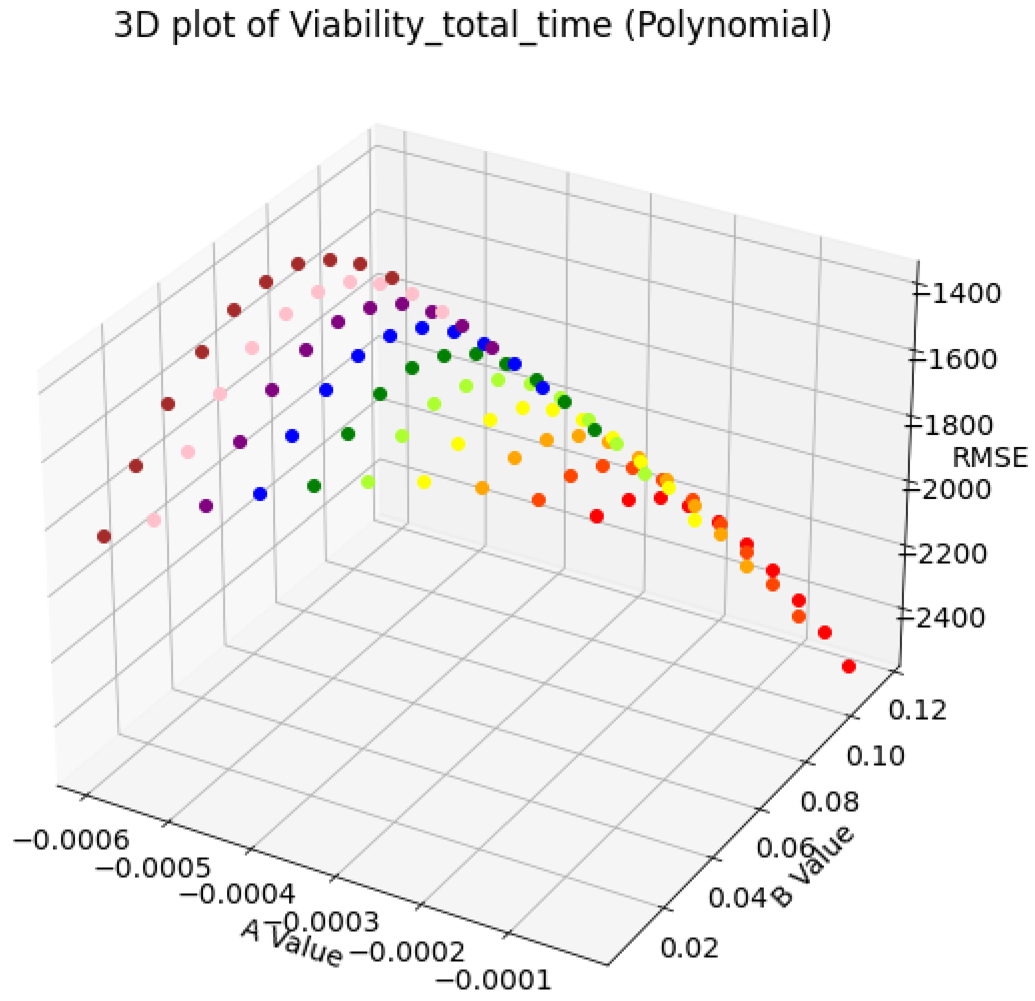
3D plot of Viability_total_time (Polynomial)
RMSE
1400
1600
1800
2000
2200
2400
B Value
0.02
0.04
0.06
0.08
0.10
0.12
A Value
−0.0006
−0.0005
−0.0004
−0.0003
−0.0002
−0.0001

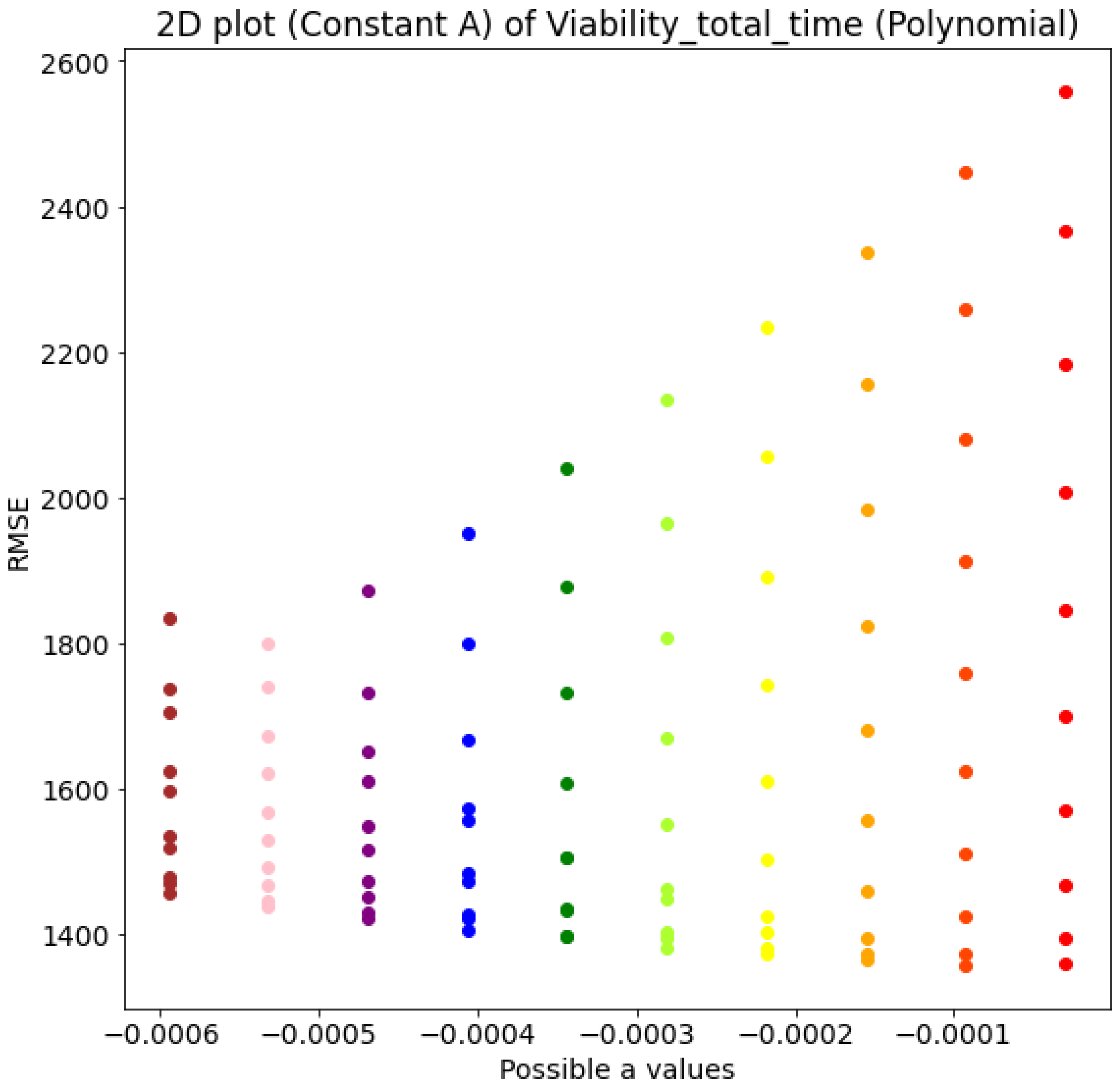
2D plot (Constant A) of Viability_total_time (Polynomial)
RMSE
2600
2400
2200
2000
1800
1600
1400
−0.0006
−0.0005
−0.0004
−0.0003
−0.0002
−0.0001
Possible a values

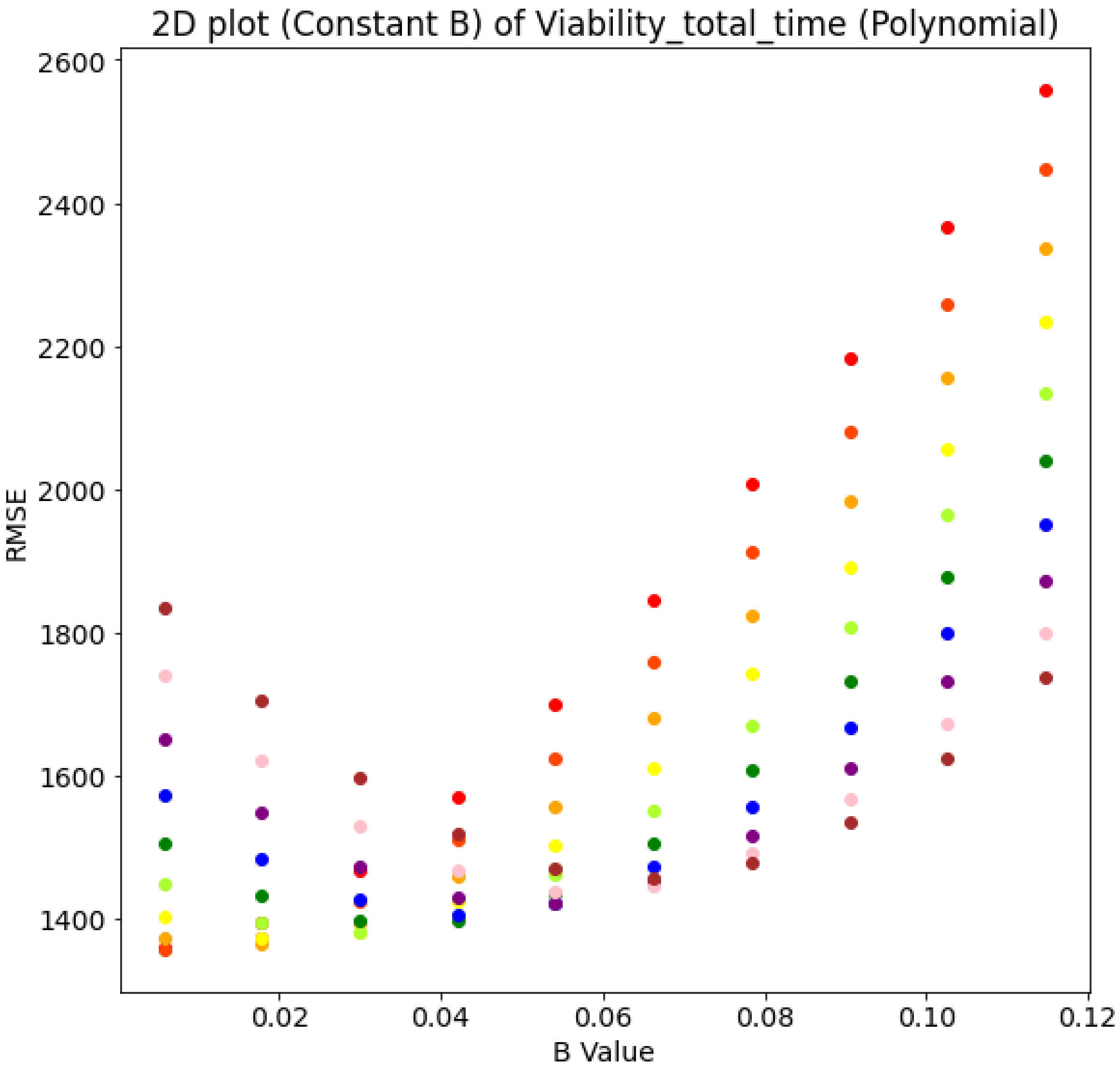
2D plot (Constant B) of Viability_total_time (Polynomial)
RMSE
B Value
2600
2400
2200
2000
1800
1600
1400
0.02
0.04
0.06
0.08
0.10
0.12

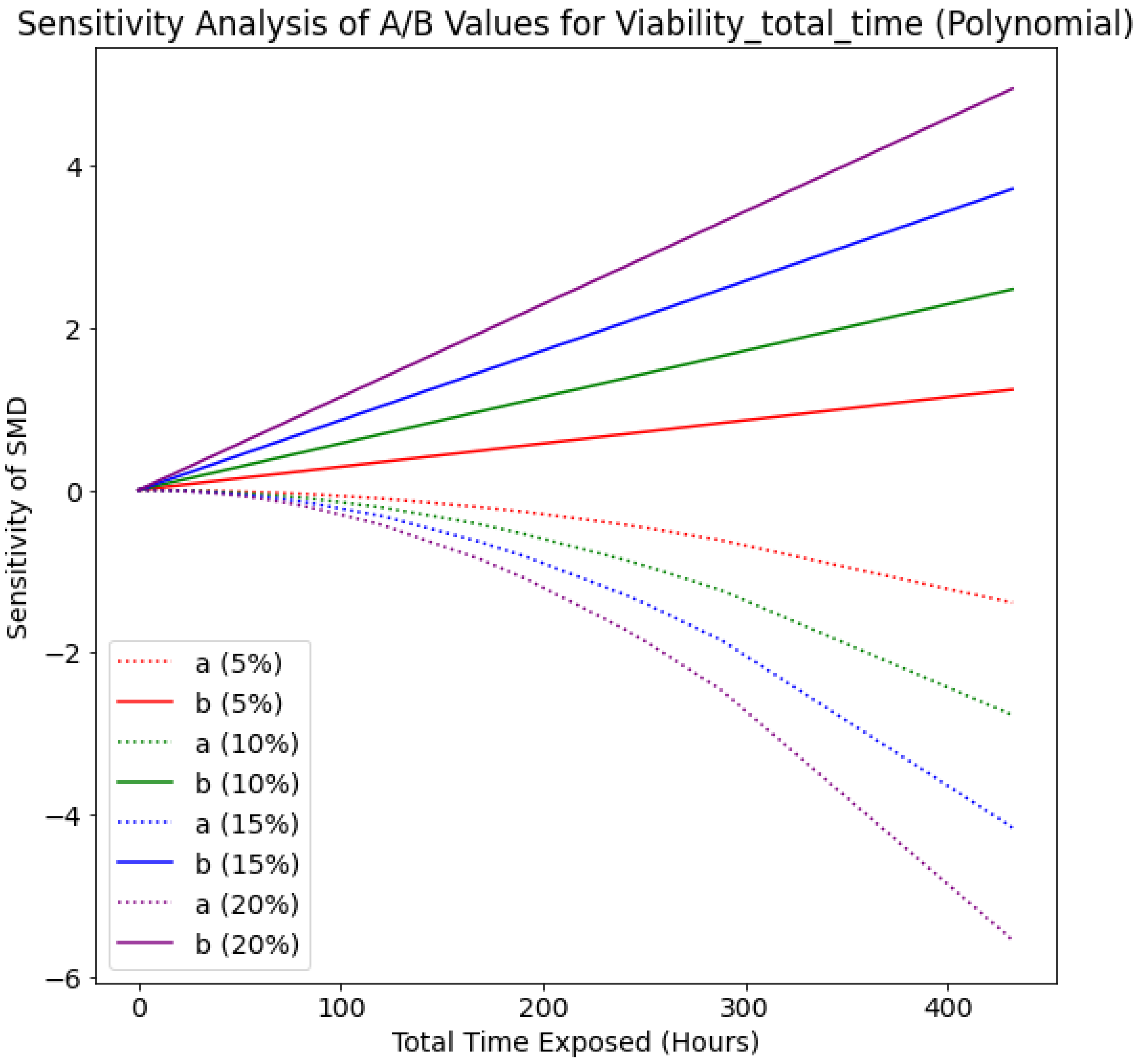
Sensitivity Analysis of A/B Values for Viability_total_time (Polynomial)
Sensitivity of SMD
Total Time Exposed (Hours)
a (5%)
b (5%)
a (10%)
b (10%)
a (15%)
b (15%)
a (20%)
b (20%)
4
2
0
−2
−4
−6
0
100
200
300
400

In [ ]:
