# Supplementary material for: Mapping the Landscape of Magnetic Field Effects on Neural Regeneration and Repair: A Combined Systematic Review, Mathematical Model, and Meta-Analysis
Source: J Tissue Eng Regen Med. 2023 Sep 21;2023:5038317. doi: 10.1155/2023/5038317 (PMC11918650; doi:10.1155/2023/5038317)
Supplement: Supplementary Materials — Supplementary Material S1: PRISMA checklist. Supplementary Material S2: MOOSE Checklist. Supplementary Material S3: original code used for simulations. Figure S1: mathematical modeling and sensitivity analyses reveal NPC viability has a robust relationship with magnitude of magnetic field exposure. Raw SMD data of viability versus magnitude. These data are used in Figure 4(c). Table S1: PECO inclusion criteria and search terms. List of population, exposure, comparison, and outcomes used to define our inclusion criteria and the exact search terms used to identify articles for screening. Table S2: full-text screening results and reason for exclusion. At the full-text screening level when articles were excluded, the reason was documented and reported here. The most common reason for exclusion was full animal exposure. Table S3: descriptions of included text outcome variables: study title (manuscript title), cell type, magnitude (mT), field type, frequency (Hz), and outcome variable. Table S4: ARRIVE Guideline Scoring on a per article basis. X-axis: the numbers correspond to the ARRIVE guideline ranking on a scale from 0 to 2. Since there were two reviewers, if the ARRIVE guideline ranking varied by only 1, the rankings were averaged. If different by 2 (i.e., one reviewer chose 0 and one chose 2), the article was discussed until a consensus was reached. Table S5: article counts used for simulations. X-axis: number of manuscripts, number of datapoints, average number of datapoints per manuscript (rounded to nearest integer), standard deviation (rounded to nearest integer), median, number of unique independent variable (e.g., if the dataset is 0, 0, 1, 2, there are 3 unique independent variable points). Y-axis: maturation, proliferation, viability, neuro/astrocyte/oligodendrocyte differentiation. Table S6: variable definition for simulations. X-axis: class (regenerative dependent variables), up/downregulate (an increase in a cellular marker result in an increase of a rege [file 5038317.f1.zip › tableS1_july13_2023.pdf]

Table S1: PECO inclusion criteria and search terms

List of PECO inclusion criteria and search terms

**PECO**	**Inclusion criteria**
P (Patient)	Human or mammal neural cells
E (Exposure)	Magnetic field
C (Comparison)	Human or mammal neural cells that are not exposed to magnetic field
O (Outcome)	Regenerative processes (differentiation, proliferation, growth, viability)
Study design	Original research article published in peer reviewed publication
Excluded	Whole organism/animal/human included, article not written in English, MRI, bacteria, plant cells, tissues that are not muscle or nerve tissue
Search Terms	"magnetic field" AND ("tissue" OR "cells") AND ("human" OR "animal") AND ("muscle" OR "brain" OR "neuron") AND ("proliferation" OR "migration" OR "differentiation" OR" regeneration") NOT ("MRI")
