# Supplementary material for: Mapping the Landscape of Magnetic Field Effects on Neural Regeneration and Repair: A Combined Systematic Review, Mathematical Model, and Meta-Analysis
Source: J Tissue Eng Regen Med. 2023 Sep 21;2023:5038317. doi: 10.1155/2023/5038317 (PMC11918650; doi:10.1155/2023/5038317)
Supplement: Supplementary Materials — Supplementary Material S1: PRISMA checklist. Supplementary Material S2: MOOSE Checklist. Supplementary Material S3: original code used for simulations. Figure S1: mathematical modeling and sensitivity analyses reveal NPC viability has a robust relationship with magnitude of magnetic field exposure. Raw SMD data of viability versus magnitude. These data are used in Figure 4(c). Table S1: PECO inclusion criteria and search terms. List of population, exposure, comparison, and outcomes used to define our inclusion criteria and the exact search terms used to identify articles for screening. Table S2: full-text screening results and reason for exclusion. At the full-text screening level when articles were excluded, the reason was documented and reported here. The most common reason for exclusion was full animal exposure. Table S3: descriptions of included text outcome variables: study title (manuscript title), cell type, magnitude (mT), field type, frequency (Hz), and outcome variable. Table S4: ARRIVE Guideline Scoring on a per article basis. X-axis: the numbers correspond to the ARRIVE guideline ranking on a scale from 0 to 2. Since there were two reviewers, if the ARRIVE guideline ranking varied by only 1, the rankings were averaged. If different by 2 (i.e., one reviewer chose 0 and one chose 2), the article was discussed until a consensus was reached. Table S5: article counts used for simulations. X-axis: number of manuscripts, number of datapoints, average number of datapoints per manuscript (rounded to nearest integer), standard deviation (rounded to nearest integer), median, number of unique independent variable (e.g., if the dataset is 0, 0, 1, 2, there are 3 unique independent variable points). Y-axis: maturation, proliferation, viability, neuro/astrocyte/oligodendrocyte differentiation. Table S6: variable definition for simulations. X-axis: class (regenerative dependent variables), up/downregulate (an increase in a cellular marker result in an increase of a rege [file 5038317.f1.zip › tables2_july13_2023.pdf]

Table S2: Full text screening results and reason for exclusion

Column titles: Title (name of manuscript), Not accessible online, Include = 1 / Exclude = 0, Not correct tissue (not neural tissue), Not magnetic field application (no magnetic fields applied), No control, Not studying regenerative process (defined as differentiation, proliferation, viability, maturation), Not original research article, Not peer reviewed, Not written in English, Whole animal/organism exposed (usually related to excluding *in vivo* studies)

Title	Include = 1 /Exclude = 0	Not correct tissue	Not magnetic field application	No control	Not studying regenerative process	Not original research article	Not peer reviewed	Not written in English	Whole animal/ organism exposed
The influence of spatial pulsed magnetic field application on neuropathic pain after tibial nerve transection in rat	0								1
Neuronal outgrowth of PC-12 cells after combined treatment with nerve growth factor and a magnetic field: Influence of the induced electric field strength	0	1							
Use of a static magnetic field to promote recovery after peripheral nerve injury	0								1
Variable spatial magnetic field influences peripheral nerves regeneration in rats	0								1
Does Pulsed Magnetic Field Therapy Influence Nerve Regeneration in the Median Nerve Model of the Rat?	0								1
Influence of static magnetic fields on nerve regeneration in vitro	0	1							
Cellular Magnetic Fields: Fundamental and Applied Measurements on Nerve Axons, Peripheral Nerve Bundles and Skeletal Muscle	0					1			
Study on Neural Regeneration Effect of Rat by Using Pulsed Functional Magnetic Stimulation	0								1
Effect of magnetic stimulation on the gene expression profile of in vitro cultured neural cells	1				1				

Gene expression profile analysis in cultured human neuronal cells after static magnetic stimulation	0	1							
Enhancement of functional recovery following a crush lesion to the rat sciatic nerve by exposure to pulsed electromagnetic fields	0								1
Stimulation of regeneration of the rat sciatic nerve by 50 Hz sinusoidal magnetic fields	0								1
Effects of static magnetic field exposure on antioxidative enzymes activity and DNA in rat brain	0				1				
Effect of pulsed magnetic field on regenerating rat sciatic nerve: An in-vitro electrophysiologic study	0								1
The stimulatory effect of magnetic fields on regeneration of the rat sciatic nerve is frequency dependent	0								1
Effect of pulsed electromagnetic stimulation on facial nerve regeneration	0								1
Effects of electromagnetic field (PEMF) exposure at different frequency and duration on the peripheral nerve regeneration: in vitro and in vivo study	1								
Co-treatment effect of pulsed electromagnetic field (PEMF) with human dental pulp stromal cells and FK506 on the regeneration of crush injured rat sciatic nerve	0	1							
Advances in electrical and magnetic stimulation on nerve regeneration	0					1			
Low intensity repetitive transcranial magnetic stimulation does not induce cell survival or regeneration in a mouse optic nerve crush model	0								1
Magnetic Nanoparticles for Efficient Delivery of Growth Factors: Stimulation of Peripheral Nerve Regeneration	0		1						

PEMF fails to enhance nerve regeneration after sciatic nerve crush lesion	0								1
Pulsed electromagnetic fields induce peripheral nerve regeneration and endplate enzymatic changes	0								1
Magnetic fields stimulate peripheral nerve regeneration in hypophysectiomized rats	0								1
Repetitive low intensity magnetic field stimulation in a neuronal cell line: A metabolomics study	0	1							
Pulsed magnetic field promotes proliferation and neurotrophic genes expression in Schwann cells in vitro	1								
Regenerative effects of pulsed magnetic field on injured peripheral nerves	0								1
The effects of pulsed electromagnetic field on experimentally induced sciatic nerve injury in rats	0								1
Partial regeneration of the sciatic nerve in rats enhances motor excitability to magnetic stimulation	0			1					
Circadian rhythm influences the promoting role of pulsed electromagnetic fields on sciatic nerve regeneration in rats	0								1
The effects of high-intensity pulsed electromagnetic field on proliferation and differentiation of neural stem cells of neonatal rats in vitro	1								
Pulsed magnetic fields enhance the rate of recovery of damaged nerve excitability	0								1
The direct influence of electromagnetic fields on nerve- and muscle cells of man within the frequency range of 1 Hz to 30 MHz	0	1							

Low-frequency pulsed electromagnetic field promotes functional recovery, reduces inflammation and oxidative stress, and enhances HSP70 expression following spinal cord injury	0								1
Exposure to 60-Hz magnetic fields and proliferation of human astrocytoma cells in vitro	0	1							
A conditioning lesion promotes in vivo nerve regeneration in the contralateral sciatic nerve of rats	0								1
Exposure to Pulsed Magnetic Fields Enhances Motor Recovery in Cats after Spinal Cord Injury	0								1
Low frequency pulsed electromagnetic field promotes the recovery of neurological function after spinal cord injury in rats	0								1
Comparison of effects of high- and low-frequency electromagnetic fields on proliferation and differentiation of neural stem cells	1								
An evaluation of genotoxicity in human neuronal-type cells subjected to oxidative stress under an extremely low frequency pulsed magnetic field	0	1							
Therapeutic effects of acrobatic exercise and magnetic field exposure on functional recovery after spinal cord injury in mice	0								1
Neurophysiological Effects Induced in the Nervous Tissue by Low-Frequency, Pulsed Magnetic Fields	1				1				
Effect of weak, interrupted sinusoidal low frequency magnetic field on neural regeneration in rats: Functional evaluation	0								1
Effects of high frequency repetitive transcranial magnetic stimulation on	0								1

KCC2 expression in rats with spasticity following spinal cord injury									
Effect of extremely low frequency magnetic field in prevention of spinal cord injury-induced osteoporosis	0								1
Effects of acute exposure to static magnetic field on ionic composition of rat spinal cord	0								1
Effect of Low Intensity Magnetic Field Stimulation on Calcium-Mediated Cytotoxicity After Mild Spinal Cord Contusion Injury in Rats	0								1
Effects of repetitive magnetic stimulation on motor function and GAP43 and 5-HT expression in rats with spinal cord injury	0								1
The Regenerative Effect of Trans-spinal Magnetic Stimulation After Spinal Cord Injury: Mechanisms and Pathways Underlying the Effect	0								1
Spinal cord injury-induced astrocyte migration and glial scar formation: Effects of magnetic stimulation frequency	0								1
Partial Recovery of Silver Nanoparticle-Induced Neural Cytotoxicity through the Application of a Static Magnetic Field	0				1				
Biomaterials and Magnetic Stem Cell Delivery in the Treatment of Spinal Cord Injury	0								1
Mechanism of GABA receptors involved in spasticity inhibition induced by transcranial magnetic stimulation following spinal cord injury	0								1
The response of the neuronal activity in the somatosensory cortex after high-intensity intermediate-frequency magnetic field exposure to the spinal cord in rats under anesthesia and waking states	0								1

Highly efficient magnetic targeting of mesenchymal stem cells in spinal cord injury	0								1
Astrocytes contribute to the neuronal recovery promoted by high-frequency repetitive magnetic stimulation in in vitro models of ischemia	1						-		
Repetitive transcranial magnetic stimulation promotes neural stem cell proliferation via the regulation of mir-25 in a rat model of focal cerebral ischemia	0								1
High-frequency repetitive transcranial magnetic stimulation improves functional recovery by inhibiting neurotoxic polarization of astrocytes in ischemic rats	1								
Repetitive magnetic stimulation promotes neural stem cells proliferation by upregulating MiR-106b in vitro	1								
Repetitive transcranial magnetic stimulation does not influence immunological HL-60 cells and neuronal PC12 cells	0	1							
High-frequency repetitive transcranial magnetic stimulation for treating moderate traumatic brain injury in rats: A pilot study	0								1
Static Magnetic Field Induced Neural Stem/Progenitor Cell Early Differentiation and Promotes Maturation	1								
Optimising repetitive transcranial magnetic stimulation for neural circuit repair following traumatic brain injury	0					1			
Combined effects of 50 Hz magnetic field and magnetic nanoparticles on the proliferation and apoptosis of PC12 cells	0	1							
Effects of acute and chronic low frequency electromagnetic field exposure	0	1							

on PC12 cells during neuronal differentiation									
Changes in neurite outgrowth but not in cell division induced by low EMF exposure: Influence of field strength and culture conditions on responses in rat PC12 pheochromocytoma cells	0	1							
No effect of pulsed electromagnetic fields on PC12 and HL-60 cells	0	1							
Neuronal differentiation of chromaffin cells in vitro, induced by extremely low frequency magnetic fields or nerve growth factor: A histological and ultrastructural comparative study	1								
Extremely low-frequency electromagnetic fields promote in vitro neuronal differentiation and neurite outgrowth of embryonic neural stem cells via up- regulating TRPC1	1								
60 Hz magnetic field exposure induces DNA crosslinks in rat brain cells	0								1
Static Magnetic Field Effect on Cell Alignment, Growth, and Differentiation in Human Cord-Derived Mesenchymal Stem Cells	0	1							
In vitro developmental neurotoxicity following chronic exposure to 50 Hz extremely low-frequency electromagnetic fields in primary rat cortical cultures	0				0				
Extremely low frequency magnetic fields promote neurite varicosity formation and cell excitability in cultured rat chromaffin cells	1								
Influence of pulsed electromagnetic field with different pulse duty cycles on neurite outgrowth in PC12 rat pheochromocytoma cells	0	1							
Effects of repetitive magnetic stimulation on the growth of primarily cultured	1				0				

hippocampus neurons in vitro and their expression of iron-containing enzymes									
Effect of repetitive transcranial magnetic stimulation on auditory function following acoustic trauma	0								1
Stimulation of neurite outgrowth in PC12D cells by ELF magnetic field and suppression by melatonin	0	1							
Magnetic field-induced DNA strand breaks in brain cells of the rat	0								1
Acute neuroprotective effects of extremely low-frequency electromagnetic fields after traumatic brain injury in rats	0								1
Oxidative stress effects on the central nervous system of rats after acute exposure to ultra high frequency electromagnetic fields	0								1
Neurite Outgrowth on Chromaffin Cells Applying Extremely Low Frequency Magnetic Fields by Permanent Magnets	1								
Short Duration Electrical Stimulation to Enhance Neurite Outgrowth and Maturation of Adult Neural Stem Progenitor Cells	0		1						
Combination of Human Mesenchymal Stem Cells and Repetitive Transcranial Magnetic Stimulation Enhances Neurological Recovery of 6-Hydroxydopamine Model of Parkinsonianâ€™s Disease	0	1							
A magnetic evaluation of peripheral nerve regeneration: II. The signal amplitude in the distal segment in relation to functional recovery	0								1
Radiofrequency Energy Loop Primes Cardiac, Neuronal, and Skeletal Muscle Differentiation in Mouse Embryonic Stem Cells: A New Tool for Improving Tissue Regeneration	0	-	1						
Biological effects of low-frequency pulsed magnetic fields on the embryonic	0								1

central nervous system development. a histological and histochemical study									
Recovery of neurophysiological features with time after rat sciatic nerve repair: A magneto-neurographic study	0								1
Acute treatment with pulsed electromagnetic fields and its effect on fast axonal transport in normal and regenerating nerve	0								1
Exposure of Nerve Growth Factor-Treated PC12 Rat Pheochromocytoma Cells to a Modulated Radiofrequency Field at 836.55 MHz: Effects on c-jun and c-fos Expression	0	1							
Effects of 60 Hz electric and magnetic fields on the development of the rat cerebellum	0								1
Effects of 50 Hz Electromagnetic Field Exposure on Apoptosis and Differentiation in a Neuroblastoma Cell Line	0	1							
The effect of magnetic stimulation on differentiation of human induced pluripotent stem cells into neuron	1								
Effects of pulsed magnetic fields on neurite outgrowth from chick embryo dorsal root ganglia	0	1							
Effectsof extremely low frequency magnetic fieldson NGF induced neuronal differentiation of PC12 cells	0	1							
Extremely low-frequency electromagnetic fields enhance the proliferation and differentiation of neural progenitor cells cultured from ischemic brains	1								
Electromagnetic fields affect transcript levels of apoptosis-related genes in embryonic stem cell-derived neural progenitor cells	1								

Magnetic stimulation supports muscle and nerve regeneration after trauma in mice	0								1
Effects of static magnetic field and cadmium on oxidative stress and DNA damage in rat cortex brain and hippocampus	0	1			1				
Enhancement of β-galactosidase gene expression in rat pheochromocytoma cells by exposure to extremely low frequency magnetic fields	0	1			1				
Effect of transcranial magnetic motor cortex stimulation on peripheral nerve regeneration: An experimental study	0				1				
D600, a Ca2+ antagonist, prevents stimulation of nerve regeneration by magnetic fields	0								1
The role of voltage-gated Ca2+ channels in neurite growth of cultured chromaffin cells induced by extremely low frequency (ELF) magnetic field stimulation	0	1							
Comparison between low frequency magnetic field stimulation and nerve growth factor treatment of cultured chromaffin cells, on neurite growth, noradrenaline release, excitable properties, and grafting in nigrostriatal lesioned rats	0	1							
Directed and enhanced neurite growth with pulsed magnetic field stimulation	1								
Action of 50 Hz magnetic fields on neurite outgrowth in pheochromocytoma cells	0	1							
Melatonin prevents magnetic-field-induced neurite outgrowth in a subline of pheochromocytoma cells, PC12D	0	1			1				

Regulation of neurite outgrowth by extracellular Ca2+ for neural cells PC12 and PC12D	0		1						
Exposure to ELF- magnetic field promotes restoration of sensori-motor functions in adult rats with hemisection of thoracic spinal cord	0								1
Acute and chronic effects of exposure to a 1-mT magnetic field on the cytoskeleton, stress proteins, and proliferation of astroglial cells in culture	1								
Pulsed electromagnetic fields potentiate neurite outgrowth in the dopaminergic MN9D cell line	1								
Electromagnetic fields influence NGF activity and levels following sciatic nerve transection	0								1
Pulsed electromagnetic fields accelerate functional recovery of transected sciatic nerve bridged by chitosan conduit: An animal model study	0								1
Effects of radiofrequency electromagnetic field exposure on neuronal differentiation and mitochondrial function in SH-SY5Y cells	0		1						
Repetitive spinal electromagnetic stimulation opens a window of synaptic plasticity in damaged spinal cord: Role of NMDA receptors	0								1
Low frequency pulsed electromagnetic field promotes differentiation of oligodendrocyte precursor cells through upregulation of miR-219-5p in vitro	1								
Static Magnetic Field Stimulation Enhances Oligodendrocyte Differentiation and Secretion of Neurotrophic Factors	1								
Extremely low frequency magnetic field induces human neuronal differentiation through NMDA receptor activation	1								

Egr1 mediated the neuronal differentiation induced by extremely low-frequency electromagnetic fields	0	1							
Extremely low-frequency electromagnetic fields affect transcript levels of neuronal differentiation-related genes in embryonic neural stem cells	1								
Extremely low-frequency electromagnetic fields enhance the survival of newborn neurons in the mouse hippocampus	1								0
The change of HCN1/HCN2 mRNA expression in peripheral nerve after chronic constriction injury induced neuropathy followed by pulsed electromagnetic field therapy	0								1
Effects of static magnetic fields on primary cortical neurons	1								
Possible promotion of neuronal differentiation in fetal rat brain neural progenitor cells after sustained exposure to static magnetism	1								
Static magnetic field controls cell cycle in cultured human glioblastoma cells	0	1							
Effect of long term exposure to 0.5 T static magnetic fields on growth and size of GH3 cells	0	1							
Differentiation of chromaffin cells elicited by ELF MF modifies gene expression pattern	1								
50-Hz magnetic field exposure influences DNA repair and mitochondrial DNA synthesis of distinct cell types in brain and kidney of adult mice	0								1
Magnetic Assembly of a Multifunctional Guidance Conduit for Peripheral Nerve Repair	0		1						
Effects of 50 Hz electromagnetic fields on voltage-gated Ca2+ channels and their	1								

role in modulation of neuroendocrine cell proliferation and death									
A magnetic evaluation of peripheral nerve regeneration: I. The discrepancy between magnetic and histologic data from the proximal segment	0		1						
Enhanced expression of neuronal nitric oxide synthase and phospholipase C-γ1 in regenerating murine neuronal cells by pulsed electromagnetic field	0								1
The expression of pluripotency and neuronal differentiation markers under the influence of electromagnetic field and nitric oxide	0	1							
Extremely low-frequency electromagnetic fields promote in vitro neurogenesis via upregulation of Ca(v)1-channel activity	1								
Non-invasive neuromagnetic monitoring of nerve and muscle injury currents	0		1		1				
Extremely low frequency electromagnetic fields promote cognitive function and hippocampal neurogenesis of rats with cerebral ischemia	0								1
Effects of Extremely Low-Frequency Electromagnetic Fields on Neurogenesis and Cognitive Behavior in an Experimental Model of Hippocampal Injury	0								1
Exposure to extremely low-frequency (50Hz) electromagnetic fields enhances adult hippocampal neurogenesis in C57BL/6 mice	0								1
Epigenetic Modulation of Adult Hippocampal Neurogenesis by Extremely Low-Frequency Electromagnetic Fields	1								
Extremely low-freauency electromagnetic fields promote in vitro neurogenesis via upregulation of Cav1-channel activity	1								
Nerves in a human body exposed to low-frequency electromagnetic fields	0								1

Insights in the biology of extremely low-frequency magnetic fields exposure on human health	0					1			
Guided neurite elongation and schwann cell invasion into magnetically aligned collagen in simulated peripheral nerve regeneration	0		1						
Extremely low frequency electromagnetic field exposure promotes differentiation of pituitary corticotrope-derived AtT20 D16V cells	0	1							
Nerve compound action current (NCAC) measurements and morphometric analysis in the proximal segment after nerve transection and repair in a rabbit model	0		1						
Cell-Based Therapy in TBI: Magnetic Retention of Neural Stem Cells In Vivo	0								1
Effectiveness of magnetically aligned collagen for neural regeneration in vitro and in vivo	0		1						
Magnetic fields at resonant conditions for the hydrogen ion affect neurite outgrowth in PC-12 cells: A test of the ion parametric resonance model	0	1							
Neural circuit repair by low-intensity magnetic stimulation requires cellular magnetoreceptors and specific stimulation patterns	0								1
Long-term exposure to a hypomagnetic field attenuates adult hippocampal neurogenesis and cognition	0								1
Evaluation of the neuroprotective effects of electromagnetic fields and coenzyme Q10 on hippocampal injury in mouse	0								1
Elimination of the geomagnetic field stimulates the proliferation of mouse neural progenitor and stem cells	1								
DNA Synthesis and Cell Proliferation in C6 Glioma and Primary Glial Cells	0		1						

Exposed to a 836.55 MHz Modulated Radiofrequency Field									
Low-Field Magnetic Stimulation Accelerates the Differentiation of Oligodendrocyte Precursor Cells via Non-canonical TGF-Î² Signaling Pathways	1								
Exposure to ELF-pulse modulated X band microwaves increases in vitro human astrocytoma cell proliferation	0	1							
Effect of controlled electromagnetic fields on the differentiation between mesenchymal stem cells and neuron cells inside nutrient media modified with carbon nanostructures: A new growth/differentiation factor	0				1				
Extremely low-frequency electromagnetic fields enhance the proliferation and differentiation of neural progenitor cells cultured from ischemic brains	1								
Magnetic field exposure saves rat cerebellar granule neurons from apoptosis in vitro	1								
Fifty Hertz Extremely Low-Frequency Magnetic Field Exposure Elicits Redox and Trophic Response in Rat-Cortical Neurons	1								
Neuroprotective effect of weak static magnetic fields in primary neuronal cultures	1								
