# Supplementary material for: Mapping the Landscape of Magnetic Field Effects on Neural Regeneration and Repair: A Combined Systematic Review, Mathematical Model, and Meta-Analysis
Source: J Tissue Eng Regen Med. 2023 Sep 21;2023:5038317. doi: 10.1155/2023/5038317 (PMC11918650; doi:10.1155/2023/5038317)
Supplement: Supplementary Materials — Supplementary Material S1: PRISMA checklist. Supplementary Material S2: MOOSE Checklist. Supplementary Material S3: original code used for simulations. Figure S1: mathematical modeling and sensitivity analyses reveal NPC viability has a robust relationship with magnitude of magnetic field exposure. Raw SMD data of viability versus magnitude. These data are used in Figure 4(c). Table S1: PECO inclusion criteria and search terms. List of population, exposure, comparison, and outcomes used to define our inclusion criteria and the exact search terms used to identify articles for screening. Table S2: full-text screening results and reason for exclusion. At the full-text screening level when articles were excluded, the reason was documented and reported here. The most common reason for exclusion was full animal exposure. Table S3: descriptions of included text outcome variables: study title (manuscript title), cell type, magnitude (mT), field type, frequency (Hz), and outcome variable. Table S4: ARRIVE Guideline Scoring on a per article basis. X-axis: the numbers correspond to the ARRIVE guideline ranking on a scale from 0 to 2. Since there were two reviewers, if the ARRIVE guideline ranking varied by only 1, the rankings were averaged. If different by 2 (i.e., one reviewer chose 0 and one chose 2), the article was discussed until a consensus was reached. Table S5: article counts used for simulations. X-axis: number of manuscripts, number of datapoints, average number of datapoints per manuscript (rounded to nearest integer), standard deviation (rounded to nearest integer), median, number of unique independent variable (e.g., if the dataset is 0, 0, 1, 2, there are 3 unique independent variable points). Y-axis: maturation, proliferation, viability, neuro/astrocyte/oligodendrocyte differentiation. Table S6: variable definition for simulations. X-axis: class (regenerative dependent variables), up/downregulate (an increase in a cellular marker result in an increase of a rege [file 5038317.f1.zip › tables3_july13_2023.pdf]

Table S3: Descriptions of included text outcome variables

Study Title (Manuscript title), Cell type, Magnitude (mT), Field Type, Frequency (Hz), Outcome Variable

Study Title	Cell type	Magnitude (mT)	Field Type	Frequency (Hz)	Outcome Variable
The effects of high-intensity pulsed electromagnetic field on proliferation and differentiation of neural stem cells of neonatal rats in vitro	neonatal murine NSCs	500,1000,3K,4K, 5K,6K,8K,10K	Dynamic	0.1	morphology, growth patterns, viabilty
Extremely low-frequency electromagnetic fields promote in vitro neuronal differentiation and neurite outgrowth of embryonic neural stem cells via up-regulating TRPC1	embryonic murine neural stem cells	1	Dynamic	50 Hz (1-3 days, 4 hours/day)	proliferation, differentiation, neurite growth, apoptosis, proneural genes, TRPC1 (diff/neurite marker)
The effect of magnetic stimulation on differentiation of human induced pluripotent stem cells into neuron	human iPSC	2400, 4800	Dynamic	10, 1 (train duration of 0.5s and 200s, respectively, intertrain interval of 6 and 20s, 1200 pulses)	differentiation, proliferation, exitatory/inhibitory signals, synapse formation
Extremely low frequency magnetic field induces human neuronal differentiation through NMDA receptor activation	hNPCs (human neural progenitor cells)	1	dynamic	50	differentiation, migration, morphology
Extremely low-frequency electromagnetic fields affect transcript levels of neuronal differentiation-related genes in embryonic neural stem cells	eNSCs (embryonic neural stem cells)	0.5,1,2	dynamic	50	viability, DNA synthesis, diameter of neuroshperes, cell cycle (related genes), differentiation
Possible promotion of neuronal differentiation in fetal rat brain neural progenitor cells after sustained exposure to static magnetism	NPCs (embryonic murine)	100	static	--	differentiation, gene expression
Extremely low-frequency electromagnetic fields promote in vitro neurogenesis via upregulation of Ca(v)1-channel activity	NSCs (neonatal murine)	1	dynamic	50	differentiation,proliferation, physiology (Ca channels), role of Ca channels on differentation, CREB phosphorylation
Elimination of the geomagnetic field stimulates the proliferation of mouse neural progenitor and stem cells	nPCs/ NSCs from C57BL/76 mice	<0.0002	static	--	growth, self-renewal, multipotency, growth factor supplementation, proliferation

Low-Field Magnetic Stimulation Accelerates the Differentiation of Oligodendrocyte Precursor Cells via Non-canonical TGF-Î² Signaling Pathways	CH4 (murine glial--progenitor) and B104 (neuroblastoma)	??	dynamic	pulses--'Every 2-s pulse was composed of 80 rhythmical trains spiking for 6 ms in 19-ms intervals, which constituted the intermittent gamma burst stimulation at 40 Hz. Each train consisted of six pulses with 130 μs width and 1000-Hz frequency'	proliferation, differentiation, TGF-B1 expression
Effect of magnetic stimulation on the gene expression profile of in vitro cultured neural cells	primary embryonic murine neural cells	133 and 640	Dynamic	biphasic, sinus-shaped pulses of 280 us [~3570 Hz] pulse width'; two simulation patterns--A: 8 impulses w a repetition rate of 70 Hz and sequence interval of 4s; B: 20 impulses w rr of 30 Hz and si of 4s	neural stem cell differentiation status, signal activity, gene expression (qRT-PCR assay and single)
Effects of electromagnetic field (PEMF) exposure at different frequency and duration on the peripheral nerve regeneration: in vitro and in vivo study	immortalized murine Schwann cells (iSCs)	1	Dynamic	50 Hz and 150 Hz (1 hour/day and 12 hour/day for 4 different conditions total)	cell proliferation, mRNA expression (S100 and brain-derived neurotrophic factor)
Pulsed magnetic field promotes proliferation and neurotrophic genes expression in Schwann cells in vitro	primary neonatal murine Schwann cells	0-20	Dynamic	50	survival, morphology, proliferation, gene expression, protein levels
Comparison of effects of high- and low-frequency electromagnetic fields on proliferation and differentiation of neural stem cells	primary neonatal murine neural cells (hippocampal)	5 and 2500	Dynamic	50; (30 and 10 min/day, respectively)	proliferation, differentiation (and markers for it), mRNA expression

Neurophysiological Effects Induced in the Nervous Tissue by Low-Frequency, Pulsed Magnetic Fields	murine hippocampal slices, synaptosomes	15	Dynamic	0.03, 0.07, 0.16, 0.5 (pulsed)	evoked potentials (population spikes), neurotransmitter and calcium accumulation, glutamate uptake, neuronal excitability
Astrocytes contribute to the neuronal recovery promoted by high-frequency repetitive magnetic stimulation in in vitro models of ischemia	primary embryonic murine neural cells	Not mentioned	Dynamic	10 Hz (24 trains of 50 pulses, biphasic waveform of 280us duration)	viability, synaptic and neurite morphology modifications, ERK1/2 and c-Fos expression
High-frequency repetitive transcranial magnetic stimulation improves functional recovery by inhibiting neurotoxic polarization of astrocytes in ischemic rats	primary embryonic murine neurons and primary neonatal murine astrocytes	1900	Dynamic	1, 5, 10 (600 pulses in 10 minutes every day for 2 days)	viability, resistance to alteration and alleviation of neurotoxic effects due to O2/Glucose deprivation, synaptic formation
Repetitive magnetic stimulation promotes neural stem cells proliferation by upregulating MiR-106b in vitro	primary murine neonatal neural cells (hippocampal)	3500	Dynamic	10 (200,400,...1000 pulses/day, stimulation interval time=10s)	proliferation, miRNA expression, gene/protein expression, cyclin-dependent kinase
Static Magnetic Field Induced Neural Stem/Progenitor Cell Early Differentiation and Promotes Maturation	primary murine neonatal neural cells	510 +- 10	Static	-	proliferation, cell cycle (and cell cycle related proteins), differentiation, morphology, electrophysiology
Neuronal differentiation of chromaffin cells in vitro, induced by extremely low frequency magnetic fields or nerve growth factor: A histological and ultrastructural comparative study	primary neonatal murine chromaffin cells	0.7	Static	-- (4hour/day for 7 days)	proliferation, morphology, electron density, cytoskeleton (growth cones, filopodia, neuritic projections, varicosities)
In vitro developmental neurotoxicity following chronic exposure to 50 Hz extremely low-frequency electromagnetic fields in primary rat cortical cultures	primary embryonic murine neural cells	0.001-1	Dynamic	50 Hz (7 days)	viability, calcium concentrations, neurite length, electrical activity
Extremely low frequency magnetic fields promote neurite varicosity formation and cell excitability in cultured rat chromaffin cells	primary murine chromaffin cells	1	Dynamic	60 Hz	morphology, neurite outgrowth, electrical activity, resting membrane potential, action potential

Effects of repetitive magnetic stimulation on the growth of primarily cultured hippocampus neurons in vitro and their expression of iron-containing enzymes	primary neonatal murine neural cells (hippocampal)	1680, 2520, 4200	Dynamic	1 Hz (3 pulse trains, each containing 100 pulses at 1 Hz, 60s interval between sequences)	morphology, survival, catalase/protein kinase A expression, aconitase (metabolic marker)
Neurite Outgrowth on Chromaffin Cells Applying Extremely Low Frequency Magnetic Fields by Permanent Magnets	primary murine chromaffin cells	0-45	Dynamic	4,7,10, and 12 Hz (1,3,5,7 days and 2,4,6,8 hours/day)	morphology, neurite outgrowth
Directed and enhanced neurite growth with pulsed magnetic field stimulation	dorsal root ganglia from murine embryos	??	dynamic	varies with pulse width (5-20us), time between pulses (50-200 us), number of pulses (22-88), repetition rate (15-25Hz), current (15-25A), Magnetic vector potential (5-8.33*10^-6 Weber/m)	neurite outgrowth
Acute and chronic effects of exposure to a 1-mT magnetic field on the cytoskeleton, stress proteins, and proliferation of astroglial cells in culture	primary astroglial cells (cerebral hemispheres of Wistar rats)	1	both	50 Hz	hsp, actin, GFAP, morphology, proliferation
Low frequency pulsed electromagnetic field promotes differentiation of oligodendrocyte precursor cells through upregulation of miR-219-5p in vitro	murine oligodendrocyte precursor cells	1.8	dynamic	50Hz (pulsed square)	differentiation, miR-219-5p and Lingo1 expression
Static Magnetic Field Stimulation Enhances Oligodendrocyte Differentiation and Secretion of Neurotrophic Factors	hiPSC derived oligodenodrocyte precursor cells	300	static	--	gene expression of cell activity, mature oligodendrocyte, secretion of neurotrophic factors. myelination capacity, calcium flux
Effects of static magnetic fields on primary cortical neurons	primary murine neurons (neonatal, cortical)	100,500,750,100, 020,000,000	static	--	phosphorylation response of ERK and JNK, JNK

Differentiation of chromaffin cells elicited by ELF MF modifies gene expression pattern	chromaffin cells (neontal murine)	0.7	dynamic	60	gene expression
Effects of 50 Hz electromagnetic fields on voltage-gated Ca2+ channels and their role in modulation of neuroendocrine cell proliferation and death	rat pituitary GH3 cells (and human neuroblastoma IMR32)	0.005-10	dynamic	1-100	proliferation,apoptosis, current through Ca channels, Ca channel expression
Extremely Low-Frequency Electromagnetic Fields Promote In Vitro Neuronal Differentiation and Neurite Outgrowth of Embryonic Neural Stem Cells via Up-Regulating TRPC1	eNSCs (from murine embryonic telencephalons)	1	dynamic	50	proliferation, maintenance, differentiation, neurite outgrowth, apoptosis, proneural genes, TRPC1 (Calcium channel) gene expression,
Extremely low-frequency electromagnetic fields enhance the proliferation and differentiation of neural progenitor cells cultured from ischemic brains	murine embryonic and ischemic brain neural progenitor cells	0.4	dynamic	50	proliferation, differentiation, phosphorylated protein kinase B
Magnetic field exposure saves rat cerebellar granule neurons from apoptosis in vitro	cerebellar granule neurons (neonatal murine)	300	dynamic	50	apoptosis, induced currents
Fifty Hertz Extremely Low-Frequency Magnetic Field Exposure Elicits Redox and Trophic Response in Rat-Cortical Neurons	murine embryonic cortical neurons	0.1,1	dynamic	50	neuronal viability, redox status/membrane peroxidative damage, neurotrophin and cytokine expressions
NEUROPROTECTIVE EFFECT OF WEAK STATIC MAGNETIC FIELDS IN PRIMARY NEURONAL CULTURES	neonatal murine cortical and hippocampal neurons	5	static	--	apoptosis, MMP collapse, Ca influx, mRNA/protein expression of VGCCs
Extremely low-frequency electromagnetic fields enhance the survival of newborn neurons in the mouse hippocampus	NSCs (neonatal murine hippocampal)	1	dynamic	50	apoptosis
Epigenetic Modulation of Adult Hippocampal Neurogenesis by Extremely Low-Frequency Electromagnetic Fields	NSCs (neonatal murine hippocampal)	1	dynamic	50	proliferation, differentiation, gene promotors
Electromagnetic fields affect transcript levels of apoptosis-related genes in embryonic stem cell-derived neural progenitor cells	murine embryonic stem cells	2	dynamic	50	differentiation markers (mRNA transcripts), cytogenetic effects, proliferation, apoptosis,
