# Supplementary material for: Mapping the Landscape of Magnetic Field Effects on Neural Regeneration and Repair: A Combined Systematic Review, Mathematical Model, and Meta-Analysis
Source: J Tissue Eng Regen Med. 2023 Sep 21;2023:5038317. doi: 10.1155/2023/5038317 (PMC11918650; doi:10.1155/2023/5038317)
Supplement: Supplementary Materials — Supplementary Material S1: PRISMA checklist. Supplementary Material S2: MOOSE Checklist. Supplementary Material S3: original code used for simulations. Figure S1: mathematical modeling and sensitivity analyses reveal NPC viability has a robust relationship with magnitude of magnetic field exposure. Raw SMD data of viability versus magnitude. These data are used in Figure 4(c). Table S1: PECO inclusion criteria and search terms. List of population, exposure, comparison, and outcomes used to define our inclusion criteria and the exact search terms used to identify articles for screening. Table S2: full-text screening results and reason for exclusion. At the full-text screening level when articles were excluded, the reason was documented and reported here. The most common reason for exclusion was full animal exposure. Table S3: descriptions of included text outcome variables: study title (manuscript title), cell type, magnitude (mT), field type, frequency (Hz), and outcome variable. Table S4: ARRIVE Guideline Scoring on a per article basis. X-axis: the numbers correspond to the ARRIVE guideline ranking on a scale from 0 to 2. Since there were two reviewers, if the ARRIVE guideline ranking varied by only 1, the rankings were averaged. If different by 2 (i.e., one reviewer chose 0 and one chose 2), the article was discussed until a consensus was reached. Table S5: article counts used for simulations. X-axis: number of manuscripts, number of datapoints, average number of datapoints per manuscript (rounded to nearest integer), standard deviation (rounded to nearest integer), median, number of unique independent variable (e.g., if the dataset is 0, 0, 1, 2, there are 3 unique independent variable points). Y-axis: maturation, proliferation, viability, neuro/astrocyte/oligodendrocyte differentiation. Table S6: variable definition for simulations. X-axis: class (regenerative dependent variables), up/downregulate (an increase in a cellular marker result in an increase of a rege [file 5038317.f1.zip › tableS4_july13_2023.pdf]

Table S4: ARRIVE Guideline Scoring on a per article basis

X-axis: The numbers correspond to the ARRIVE guideline ranking on a scale from 0 - 2. Since there were two reviewers, if the ARRIVE guideline ranking varied by only 1, the rankings were averaged. If different by 2 (one reviewer chose 0 and one chose 2), the article was discussed and a consensus was reached.

Article Title	1	2	3	4	5	6	7	8	9	10	11	12	13	17	18	19	20	21
Acute and chronic effects of exposure to a 1-mT magnetic field on the cytoskeleton, stress proteins, and proliferation of astroglial cells in culture	2	1	0	0	0	2	0	0	2	2	2	2	2	1	0	0	0	0
Astrocytes contribute to the neuronal recovery promoted by high-frequency repetitive magnetic stimulation in in vitro models of ischemia	2	1.5	0	2	2	2	1	2	2	2	1.5	2	2	1.5	1.5	0	2	2
Comparison of effects of high- and low-frequency electromagnetic fields on proliferation and differentiation of neural stem cells	2	0	0	0	0	1.5	1	1	2	1.5	2	2	2	1.5	1.5	0	0	2
Differentiation of chromaffin cells elicited by ELF MF modifies gene expression pattern	2	0.5	0	0	0	2	0	1.5	2	2	1.5	2	2	1.5	1	0	0	1
Directed and enhanced neurite growth with pulsed magnetic field stimulation	2	1.5	0	2	2	2	1.5	1.5	2	1	2	2	2	2	1	0	0	0
Effect of magnetic stimulation on the gene expression profile of in vitro cultured neural cells	2	0.5	0	2	0	2	0.5	2	2	0	1.5	2	2	2	2	0	0	0
Effects of electromagnetic field (PEMF) exposure at different frequency and duration on the peripheral nerve regeneration: in vitro and in vivo study	1.5	1	0	2	0	2	1.5	2	2	2	2	2	2	1.5	1.5	0	0	2
Effects of repetitive magnetic stimulation on the growth of primarily cultured hippocampus neurons in vitro and their expression of iron-containing enzymes	2	0	0	2	0	1.5	2	1.5	1.5	2	2	1.5	2	2	1.5	0	0	2
Effects of static magnetic fields on primary cortical neurons	2	1	0	0	0	1	0	1.5	1.5	2	2	1	1	0.5	0.5	0	0	1
Elimination of the geomagnetic field stimulates the proliferation of mouse neural progenitor and stem cells	2	0.5	0	2	0	2	1	2	2	2	2	2	2	1.5	1	0	2	2
Extremely low frequency magnetic field induces human neuronal differentiation through NMDA receptor activation	2	1.5	0	0	0	2	1	1.5	1.5	2	2	2	2	1.5	1	0	0	2
Extremely low frequency magnetic fields promote neurite varicosity formation and cell excitability in cultured rat chromaffin cells	2	1	0	2	0	2	0.5	0.5	1.5	1.5	0.5	2	2	1.5	1	0	0	0

Extremely low-frequency electromagnetic fields affect transcript levels of neuronal differentiation-related genes in embryonic neural stem cells	2	1.5	0	2	2	2	1	1.5	2	2	2	2	2	1.5	1.5	0	0	2
Extremely low-frequency electromagnetic fields promote in vitro neurogenesis via upregulation of Ca(v)1-channel activity	1.5	1.5	0.5	2	2	2	1	1.5	2	2	2	2	2	1.5	1.5	0	0	0
Extremely low-frequency electromagnetic fields promote in vitro neuronal differentiation and neurite outgrowth of embryonic neural stem cells via up- regulating TRPC1	2	1.5	0.5	2	2	2	1	1.5	2	2	2	2	2	1.5	1.5	0	2	2
High-frequency repetitive transcranial magnetic stimulation improves functional recovery by inhibiting neurotoxic polarization of astrocytes in ischemic rats	1.5	1	0	2	0	2	1.5	2	2	2	2	2	2	2	2	0	2	0
In vitro developmental neurotoxicity following chronic exposure to 50 Hz extremely low-frequency electromagnetic fields in primary rat cortical cultures	2	1.5	1	0	2	2	2	2	1.5	2	2	2	2	1.5	1	0	2	2
Low frequency pulsed electromagnetic field promotes differentiation of oligodendrocyte precursor cells through upregulation of miR-219-5p in vitro	1.5	1.5	0	2	2	2	1.5	1.5	1.5	1	2	2	2	2	2	0	0	2
Low-Field Magnetic Stimulation Accelerates the Differentiation of Oligodendrocyte Precursor Cells via Non-canonical TGF-Î² Signaling Pathways	2	1	0	0	0	2	1.5	1	1.5	2	2	2	2	2	2	0	0	2
Neurite Outgrowth on Chromaffin Cells Applying Extremely Low Frequency Magnetic Fields by Permanent Magnets	2	1.5	0	2	0	2	1	1.5	2	2	2	2	2	1.5	1.5	0	0	0
Neuronal differentiation of chromaffin cells in vitro, induced by extremely low frequency magnetic fields or nerve growth factor: A histological and ultrastructural comparative study	2	0	0	0	0	2	0	1	2	0	2	2	2	1	0.5	0	0	1
Neurophysiological Effects Induced in the Nervous Tissue by Low-Frequency, Pulsed Magnetic Fields	1	0.5	0	0	0	1	0	2	2	1.5	1.5	1.5	1	1.5	2	0	0	1
Possible promotion of neuronal differentiation in fetal rat brain neural progenitor cells after sustained exposure to static magnetism	2	1	0	2	2	2	1	2	1.5	2	2	2	2	1.5	1	0	0	0
Pulsed magnetic field promotes proliferation and neurotrophic genes expression in Schwann cells in vitro	1.5	1	0	0	0	1.5	1	1	2	2	1.5	2	2	2	1.5	0	0	2
Repetitive magnetic stimulation promotes neural stem cells proliferation by upregulating MiR-106b in vitro	2	0.5	0	2	2	2	1.5	1.5	2	2	2	2	2	1.5	2	0	2	2
Static Magnetic Field Induced Neural Stem/Progenitor Cell Early Differentiation and Promotes Maturation	1.5	0.5	0	0	0	2	1	1.5	1.5	2	1.5	2	2	2	1.5	0	2	2
Static Magnetic Field Stimulation Enhances Oligodendrocyte Differentiation and Secretion of Neurotrophic Factors	2	1	0.5	0	0	2	1	1.5	2	2	2	2	2	1.5	0.5	0	2	2

The effect of magnetic stimulation on differentiation of human induced pluripotent stem cells into neuron	2	0	0	2	0	2	1	1.5	2	2	1.5	2	2	2	1.5	0	2	2
The effects of high-intensity pulsed electromagnetic field on proliferation and differentiation of neural stem cells of neonatal rats in vitro	1.5	0.5	0	0	0	2	1	1	1.5	1.5	2	0.5	2	0.5	1	0	0	0
Effects of 50 Hz electromagnetic fields on voltage-gated Ca2+ channels and their role in modulation of neuroendocrine cell proliferation and death	2	1.5	0	0	2	2	0.5	1	2	2	2	2	2	1.5	1.5	0	0	1
Extremely low-frequency electromagnetic fields enhance the proliferation and differentiation of neural progenitor cells cultured from ischemic brains	2	1.5	0	0	0	2	1	2	2	2	1.5	2	1	1.5	2	0	0	2
Magnetic field exposure saves rat cerebellar granule neurons from apoptosis in vitro	2	1.5	0	1.5	0	2	0	1	2	2	2	1.5	2	0.5	0.5	0	0	1
Fifty Hertz Extremely Low-Frequency Magnetic Field Exposure Elicits Redox and Trophic Response in Rat-Cortical Neurons	2	1.5	0	0	0	2	1.5	1	2	2	1.5	2	2	1.5	2	0	0	1
Neuroprotective effect of weak static magnetic fields in primary neuronal cultures	1.5	2	0	1.5	2	2	1.5	1	2	2	1.5	2	2	1.5	1	0	0	0
Extremely low-frequency electromagnetic fields enhance the survival of newborn neurons in the mouse hippocampus	1.5	1.5	0	0	2	2	2	1.5	2	2	2	2	2	1.5	1.5	0.5	0	1
Epigenetic Modulation of Adult Hippocampal Neurogenesis by Extremely Low-Frequency Electromagnetic Fields	2	1.5	0	2	2	2	1.5	1.5	1.5	2	1.5	2	1	1.5	2	0.5	2	1
Electromagnetic fields affect transcript levels of apoptosis-related genes in embryonic stem cell-derived neural progenitor cells	2	1.5	0	1.5	2	2	1	0.5	2	2	1.5	2	1	1.5	1.5	0	0	1
Extremely Low-Frequency Electromagnetic Fields Promote In Vitro Neuronal Differentiation and Neurite Outgrowth of Embryonic Neural Stem Cells via Up-Regulating TRPC1	1.5	1.5	0	0	0	2	1.5	2	2	2	1.5	2	1	1.5	2	0	2	0
