# Supplementary material for: Mapping the Landscape of Magnetic Field Effects on Neural Regeneration and Repair: A Combined Systematic Review, Mathematical Model, and Meta-Analysis
Source: J Tissue Eng Regen Med. 2023 Sep 21;2023:5038317. doi: 10.1155/2023/5038317 (PMC11918650; doi:10.1155/2023/5038317)
Supplement: Supplementary Materials — Supplementary Material S1: PRISMA checklist. Supplementary Material S2: MOOSE Checklist. Supplementary Material S3: original code used for simulations. Figure S1: mathematical modeling and sensitivity analyses reveal NPC viability has a robust relationship with magnitude of magnetic field exposure. Raw SMD data of viability versus magnitude. These data are used in Figure 4(c). Table S1: PECO inclusion criteria and search terms. List of population, exposure, comparison, and outcomes used to define our inclusion criteria and the exact search terms used to identify articles for screening. Table S2: full-text screening results and reason for exclusion. At the full-text screening level when articles were excluded, the reason was documented and reported here. The most common reason for exclusion was full animal exposure. Table S3: descriptions of included text outcome variables: study title (manuscript title), cell type, magnitude (mT), field type, frequency (Hz), and outcome variable. Table S4: ARRIVE Guideline Scoring on a per article basis. X-axis: the numbers correspond to the ARRIVE guideline ranking on a scale from 0 to 2. Since there were two reviewers, if the ARRIVE guideline ranking varied by only 1, the rankings were averaged. If different by 2 (i.e., one reviewer chose 0 and one chose 2), the article was discussed until a consensus was reached. Table S5: article counts used for simulations. X-axis: number of manuscripts, number of datapoints, average number of datapoints per manuscript (rounded to nearest integer), standard deviation (rounded to nearest integer), median, number of unique independent variable (e.g., if the dataset is 0, 0, 1, 2, there are 3 unique independent variable points). Y-axis: maturation, proliferation, viability, neuro/astrocyte/oligodendrocyte differentiation. Table S6: variable definition for simulations. X-axis: class (regenerative dependent variables), up/downregulate (an increase in a cellular marker result in an increase of a rege [file 5038317.f1.zip › tableS6_july13_2023.pdf]

Table S6: Variable definition for simulations

X – axis: Class (Regenerative dependent variables), up/downregulate (an increase in a cellular marker result in an increase of a regenerative dependent variable), Variable (specific cellular markers/ characteristics), Description (of the specific cellular markers), Applicable Studies (number of manuscripts that utilize a specific variable)

*=characteristic is included in two categories (regenerative dependent variable)

**= characteristic is included in three categories (regenerative dependent variable)

**Class**	**Up/down regulate**	**Variable**	**Description**	**Applicable Studies (put reference in eventually)**	**Variable**	**Description**	**Applicable Studies**
Differentiation	Up	NSE	Neuron specific enolase	1	O4	Oligodendrocyte marker	1
		GFAP	Glial Fibrillary Astrocyte Protein	6	B-III tubulin		2
		Tuj1		6	Nestin		1
		NeuroD		3	Galc		1
		Mash1		3	MBP	Myelin basic protein	1
		Math3		3	Olig1		1
		Ngn1	Neurogenin	3	miR-219-5p		1
		Ngn2		3	CNP	2',3' Cyclic Nucleotide 3' Phosphodiesterase	2
		Hes6		2	SOX10	Sry-type HMG box	1
		Olig2	Oligodendrocyte transcription factor	1	NT-3**	Neurotropin	2
		Map2	Microtubules associated protein	5	NG2*	Neuron-glial antigen	1
		DCX	Doublecortin	1	NGF**	Nerve growth factor	1
		Other		1	TRKa**	Neurotrophic receptor tyrosine kinase	1
		TRKb**		1	GDNF**	Glial cell derived neurotrophic factor	1
		pCREB**	cAMP response element binding protein	1			

	Down	Hes1**		4	s100**		3
		Hes5*		3	Lingo1*	Leucine Rich Repeat and Ig Domain Containing	1
Proliferation	Up	MTT assay*	3 – (4,5 – dimethylthiazole– 2 – yl) – 2,5 – diphenyltetrazolium bromide	3	Cyclin D		1
		EdU	5-Ethynyl-2’-deoxyuridine	4	Cyclin E		1
		Primary NPs	neurospheres	1	Cyclin B		1
		Secondary NPs		1	PCNA	Proliferating cell nuclear antigen	1
		Sox2		3	NG2*		1
		Ki67		1	NGF**		1
		CCK-8 Assay*	Cell Counting Kit	4	TrkA**		1
		Cells in G1	Growth/ gap	2	TrkB**		1
		Cells in G2		2	pCREB**		1
		Cells in S	synthesis	2	Cells in M1	Mitosis	1
		Neurosphere diameter		2	Cells in M2		1
		BrdU	Bromodeoxyuridine	4	Cells in M3		1
		Total neurospheres (>50 um)		1	Covered areas*		1
		Total neurospheres (>100 um)		1			
	Down	Hes1**		4	s100**		3
		Hes5*		3	miR-338		1
		p53		2	miR-138		1

		p21		2	miR-9		1
		GADD45	Growth arrest and DNA Damage-inducible	2			
Viability	Up	MTT Assay*		3	TrkA**		1
		CCK-8 Assay*		4	TrkB**		1
		BDNF**		3	MTS	Mitochondrial Targeting Sequence	1
		NT-3**		2	NGF**		1
		Mitochondrial activity		1	PI/Calcein	Potassium iodide	1
		Cell membrane integrity (CFDA)	Carboxyfluorescein Diacetate Acetoxymethyl Ester	1	GDNF*		1
		BCL2		1			
	Down	Hes1**		4	TUNEL	Terminal deoxynucleotidyl transferase dUTP nick end labeling	1
		s100**		4	DNA fragmentation apoptosis ratio		1
		Flow cytometry apoptosis ratio		1	Bax	Bcl-2 Associated X-protein	1
Growth	Up	Total length of neurite		4	NT-3**		2
		Number of primary neurites		1	Number of cells showing neurite outgrowth		1
		Number of branch points		1	Covered area of flask		1
		BDNF**		3			
	Down	Nude axons		1			

* = characteristic is included in two categories. ** = characteristic is included in three categories
