# Supplementary material for: Mapping the Landscape of Magnetic Field Effects on Neural Regeneration and Repair: A Combined Systematic Review, Mathematical Model, and Meta-Analysis
Source: J Tissue Eng Regen Med. 2023 Sep 21;2023:5038317. doi: 10.1155/2023/5038317 (PMC11918650; doi:10.1155/2023/5038317)
Supplement: Supplementary Materials — Supplementary Material S1: PRISMA checklist. Supplementary Material S2: MOOSE Checklist. Supplementary Material S3: original code used for simulations. Figure S1: mathematical modeling and sensitivity analyses reveal NPC viability has a robust relationship with magnitude of magnetic field exposure. Raw SMD data of viability versus magnitude. These data are used in Figure 4(c). Table S1: PECO inclusion criteria and search terms. List of population, exposure, comparison, and outcomes used to define our inclusion criteria and the exact search terms used to identify articles for screening. Table S2: full-text screening results and reason for exclusion. At the full-text screening level when articles were excluded, the reason was documented and reported here. The most common reason for exclusion was full animal exposure. Table S3: descriptions of included text outcome variables: study title (manuscript title), cell type, magnitude (mT), field type, frequency (Hz), and outcome variable. Table S4: ARRIVE Guideline Scoring on a per article basis. X-axis: the numbers correspond to the ARRIVE guideline ranking on a scale from 0 to 2. Since there were two reviewers, if the ARRIVE guideline ranking varied by only 1, the rankings were averaged. If different by 2 (i.e., one reviewer chose 0 and one chose 2), the article was discussed until a consensus was reached. Table S5: article counts used for simulations. X-axis: number of manuscripts, number of datapoints, average number of datapoints per manuscript (rounded to nearest integer), standard deviation (rounded to nearest integer), median, number of unique independent variable (e.g., if the dataset is 0, 0, 1, 2, there are 3 unique independent variable points). Y-axis: maturation, proliferation, viability, neuro/astrocyte/oligodendrocyte differentiation. Table S6: variable definition for simulations. X-axis: class (regenerative dependent variables), up/downregulate (an increase in a cellular marker result in an increase of a rege [file 5038317.f1.zip › suppMatMethods_aug17_2023.pdf]

Supplementary Materials and Methods

Systematic Review

We performed electronic searches of PubMed, Association for Computing Machinery (ACM) Library, Association for Information Systems eLibrary (AISeL), Institute of Electrical and Electronics Engineers (IEEE) Library, Springer, Web of Science, Scopus, Science Direct, and arXiv on September 15, 2021, with search terms outlined in **Table S1**. A manual search on Google Scholar was also performed. Afterwards, we performed title and abstract screening in ASReview, which is a machine learning algorithm for time-efficient and reproducible screening of titles and abstracts.[1] Full-text screening was performed by two independent reviewers (MM, JB) based on the Cochran Handbook recommendations.[2] Included studies were published in peer-reviewed journals and written in English. Publications were excluded if they studied the whole organism, other organismal tissues (e.g., bacteria, plant), non-neural cell types, or a high static magnetic field (as experienced in a magnetic resonance imaging (MRI) scanner). We then performed a citation search of all included publications using Web of Science.[2] All screening steps were completed in pre-designed spreadsheets, and disagreements between reviewers were adjudicated by a third reviewer (GG). One reviewer (MM) extracted study information from the included publications (title, cell type, outcome variable category, specific outcome variable, sample size, magnitude, frequency, total exposure time, time after exposure).

Rigor and Reproducability Analysis

Two independent reviewers (MM and GG) ranked the ARRIVE guideline categories as 'clearly insufficient' (0), 'unclear if sufficient' (1) or 'clearly sufficient' (2). Any disagreements in ranking that differed by 2 were discussed until a consensus was reached; otherwise, the results

were averaged. Since our analysis was limited to *in vitro* studies, conditions 14-16 ("Ethical Statement", "Housing and Husbandry", "Animal Care and Monitoring") were not included in this assessment. The maximum score was 36, indicating the highest rigor, whereas the lowest score possible was a 0, indicating a complete lack of rigor. Individual categories (e.g., "Randomization") were considered 'sufficiently reported' if they had an average score > 1 and a standard deviation < 0.25, whereas 'insufficiently reported' represented an average score < 1 and a standard deviation < 0.25. An 'unclear if sufficiently reported' was recorded if the category score did not fall into these two categories. To test our hypothesis that articles more recently published would have higher ARRIVE scores, SPSS Statistics for Windows (version 28.0, IBM Corp., Armonk, NY, USA) was used to analyze the Spearman correlation between a study's ARRIVE score and publication year.

Mathematical Modeling

For dependent variables that are inversely correlated with their category, the sign of the SMD value was reversed. For example, while BrdU is positively correlated with proliferation,[3] p53 is inversely correlated with proliferation.[4] In the case of BrdU, an increase in the experimental group compared to the control would yield a positive SMD, while for p53, this would result in a negative SMD. As such, the sign of the p53 SMD would be flipped from negative to positive for the purpose of compiling all metrics together. This distinction prevents the following situation: if there is a variable that is known to be 1:1 positively correlated with the independent variable (y = x), while another variable that is known to be 1:1 negatively correlated with the independent variable (y = -x), upon combination, there will be no correlation (y = 0). By reversing the negatively correlated function, we can simply track changes as related to the

expected behavior of the variable, with positive being in the direction of enhanced activity, and negative being in the direction of attenuated activity. For articles that included multiple exposure settings, we included each condition from that dataset as an independent input into our model.

After we ran the fits, we considered which datasets had significant *a* and/or *b* values and completed a series of sensitivity analyses to test the robustness of these findings. We first perturbed the *a* and/or *b* values from 10 – 200% of their original values and reconverged the RMSE to quantify how the model was affected by changes in *a* and *b*. If the RMSE was affected by changes in both *a* and *b* or just *a*, then the polynomial model was analyzed. If just *b* affected the RMSE, only the linear model was analyzed. We then altered *a* and/or *b* values again (increased 5 – 20%) to observe the effects on predicted SMD and to analyze the robustness of our model as a function of error in our coefficients. We marked a model as robust if a 20% change in *a* and/or *b* resulted in less than an absolute value change of 2 for the SMD.

Supplementary Results

Rigor and reproducability analysis

When looking qualitatively at factors driving our R&R results, we found that most studies listed the sample size but did not report power analyses (n = 33 studies that did not comply). We also found that some, but not all, procedures were randomized (n =19 not randomized) and blinded (n = 15 not blinded). While almost every study reported the statistical tests performed, few cited whether their datasets met the appropriate assumptions (n = 29 did not cite assumptions). Some studies did not clearly articulate the limitations of their studies (n=27) or the clinical translatability (n = 27). No study registered their protocols, and most studies did

not report a means for accessing the data (n = 27). With regards to declaration of interests, many studies did not include grant funding (n = 18). Lastly, no study included descriptions of the passage number of cells, or the age or sex of the animal from which cells were derived.

Supplementary References

1 van de Schoot, R. *et al.* An open source machine learning framework for efficient and transparent systematic reviews. *Nature Machine Intelligence* **3**, 125-133, doi:10.1038/s42256-020-00287-7 (2021).

2 Higgins JPT, T. J., Chandler J, Cumpston M, Li T, Page MJ, Welch VA (editors). *Cochrane Handbook for Systematic Reviews of Interventions*. 6.3 edn, (2022).

3 Rothaeusler, K. & Baumgarth, N. Assessment of cell proliferation by 5-bromodeoxyuridine (BrdU) labeling for multicolor flow cytometry. *Curr Protoc Cytom* **Chapter 7**, Unit7 31, doi:10.1002/0471142956.cy0731s40 (2007).

4 Goh, A. M. *et al.* Mutant p53 accumulates in cycling and proliferating cells in the normal tissues of p53 R172H mutant mice. *Oncotarget* **6**, 17968-17980, doi:10.18632/oncotarget.4956 (2015).
